# Integrating Flux Balance Analysis into Kinetic Models to Decipher the Dynamic Metabolism of *Shewanella oneidensis* MR-1

**DOI:** 10.1371/journal.pcbi.1002376

**Published:** 2012-02-02

**Authors:** Xueyang Feng, You Xu, Yixin Chen, Yinjie J. Tang

**Affiliations:** 1 Department of Energy, Environmental and Chemical Engineering, Washington University, St. Louis, Missouri, United States of America; 2 Department of Computer Science and Engineering, Washington University, St. Louis, Missouri, United States of America; University of Wisconsin-Madison, United States of America

## Abstract

*Shewanella oneidensis* MR-1 sequentially utilizes lactate and its waste products (pyruvate and acetate) during batch culture. To decipher MR-1 metabolism, we integrated genome-scale flux balance analysis (FBA) into a multiple-substrate Monod model to perform the dynamic flux balance analysis (dFBA). The dFBA employed a static optimization approach (SOA) by dividing the batch time into small intervals (i.e., ∼400 mini-FBAs), then the Monod model provided time-dependent inflow/outflow fluxes to constrain the mini-FBAs to profile the pseudo-steady-state fluxes in each time interval. The mini-FBAs used a dual-objective function (a weighted combination of “maximizing growth rate” and “minimizing overall flux”) to capture trade-offs between optimal growth and minimal enzyme usage. By fitting the experimental data, a bi-level optimization of dFBA revealed that the optimal weight in the dual-objective function was time-dependent: the objective function was constant in the early growth stage, while the functional weight of minimal enzyme usage increased significantly when lactate became scarce. The dFBA profiled biologically meaningful dynamic MR-1 metabolisms: 1. the oxidative TCA cycle fluxes increased initially and then decreased in the late growth stage; 2. fluxes in the pentose phosphate pathway and gluconeogenesis were stable in the exponential growth period; and 3. the glyoxylate shunt was up-regulated when acetate became the main carbon source for MR-1 growth.

## Author Summary

This study integrates two modeling approaches, a Monod kinetic model and genome-scale flux balance analysis, to analyze the dynamic metabolism of an environmentally important bacterium (*S. oneidensis* MR-1). The modeling results reveal that MR-1 metabolism is suboptimal for biomass growth, while MR-1 continuously reprograms the intracellular flux distributions in adaption to nutrient conditions. This innovative dFBA framework can be widely used to investigate transient cell metabolisms in response to environmental variations. Furthermore, the dFBA is able to simulate metabolite-labeling dynamics in ^13^C-tracer experiments, and thus can serve as a springboard to advanced ^13^C-assisted dynamic metabolic flux analysis by using labeled proteinogenic amino acids to improve flux results.

## Introduction

Cell metabolisms are highly dependent on environmental conditions, so the metabolic state often shifts during the cultivation period [1], [2], [3]. Characterizing the transience of metabolic fluxes is important for understanding how cells responded to environmental changes. Bioprocess models (e.g., a Monod-based kinetic model) [4] have been widely applied to predict microbial dynamics, but they cannot directly obtain the intracellular flux distributions. On the other hand, flux balance analysis (FBA) profiles the rates of enzymatic reactions based on stoichiometric mass balance, knowledge of reaction constraints, and measurements of inflow/outflow fluxes [5], [6]. As an underdetermined model, FBA requires an objective function (e.g., “maximizing growth rate”) for flux calculation. However, since cells may show suboptimal metabolism and reprogram their metabolic fluxes under different environmental conditions, the commonly used objective function is insufficient to describe cell physiologies [7], [8], [9]. Furthermore, FBA assumes steady-state metabolic conditions, and thus is unable to directly analyze the transience of cell metabolism [10], [11], [12].

**Figure 1:**
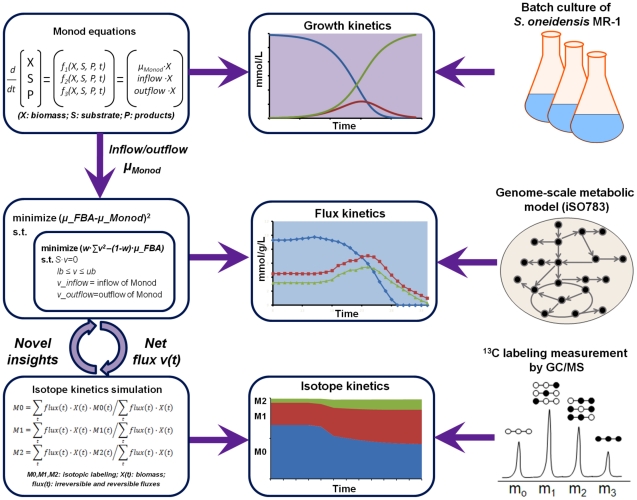
Flowchart of dFBA to decipher the dynamic metabolism of *S. oneidensis* MR-1.

This study developed an FBA framework that integrates Monod kinetics and FBA to decipher the dynamic metabolism of MR-1 (Figure 1). MR-1 is a facultative anaerobic bacterium, which not only plays an important ecological role in carbon cycling and metal reduction, but also has been widely used for *in situ* bioremediation and microbial fuel cell applications [13], [14], [15]. MR-1 has a diverse carbon utilization capability and shifts its metabolism during batch cultivation [16]. MR-1 uses lactate for initial growth and produces acetate and pyruvate. In the late growth stage, MR-1 metabolizes less energy-favorable pyruvate and acetate. To describe such kinetic behavior, we used unsegregated Monod equations to simulate cell growth, lactate utilization, and metabolite secretion and reuse. The standard Monod model was incorporated into a genome-scale FBA model, iSO783 [17], to formulate the dynamic FBA (dFBA) framework [11], which enabled quantitative predictions of the MR-1 metabolism.

## Results

### Monod model

**Figure 2:**
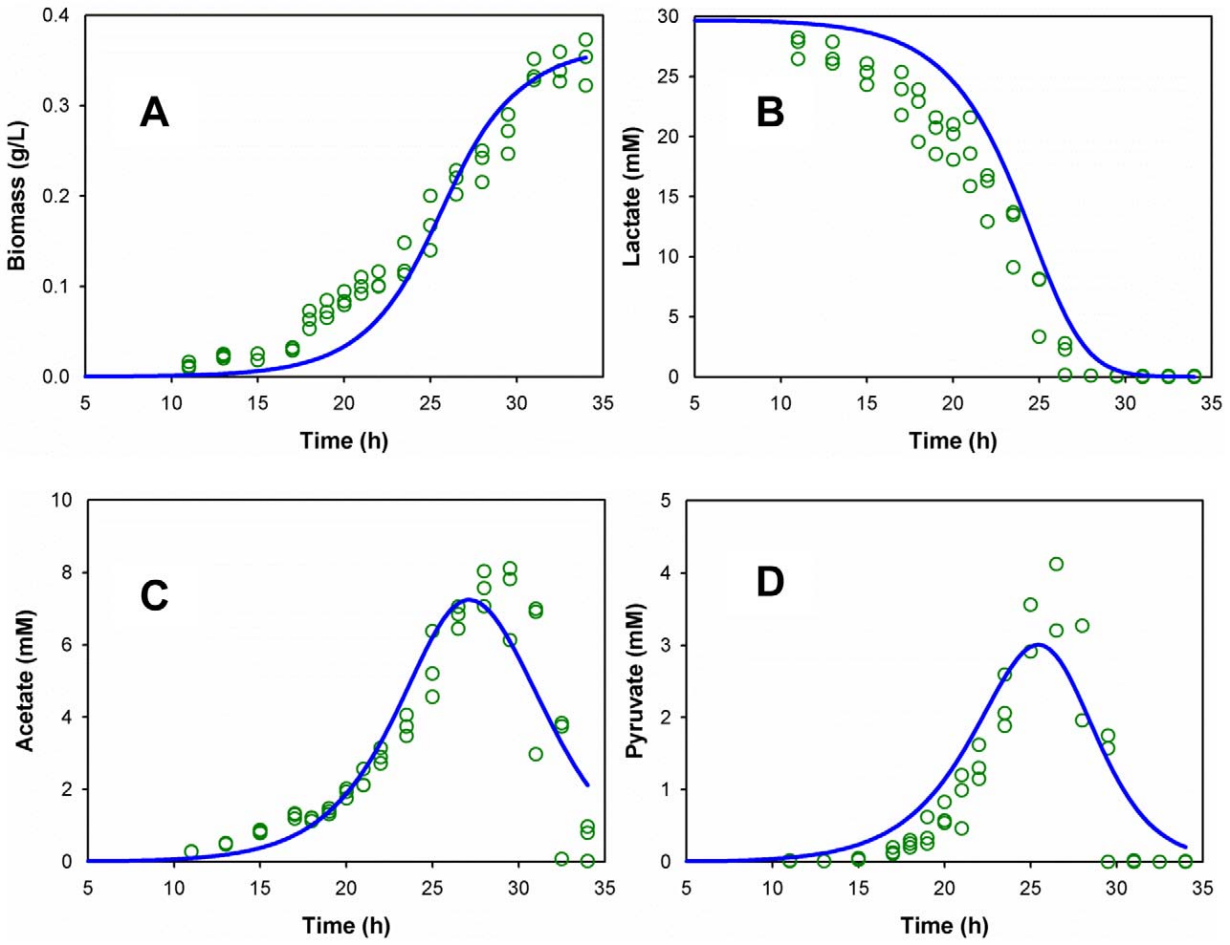
Monod model for growth kinetics. The green dots are the measurements, and the blue lines are the simulated growth by the empirical Monod model.

MR-1 growth displayed an apparent lag phase (∼7.1 h) in 30 mM lactate medium (0.1% inoculation). By incorporating a time delay function for the lag growth phase, a standard Monod model consisting of four ordinary differential equations was built to describe the extracellular metabolite curves and growth kinetics (Figure 2). The parameters of the Monod model were estimated by fitting the experimental data. Table 1 indicates that the lactate-based biomass yield was higher than that for either pyruvate or acetate, confirming the preferential utilization of lactate as an energy-favorable carbon substrate for MR-1. Similarly, the lactate-based growth rate (μ_max_) was much higher than that for either pyruvate or acetate, indicating that lactate was the major carbon substrate for biomass growth at the early growth stage. Table 1 lists the rate coefficients (*k_pl_*, *k_ap_* and *k_al_*) for waste products (pyruvate and acetate) synthesis and reuse, which indicates that MR-1 quickly consumed lactate, producing significant metabolic overflows to the waste products. Such a strategy illustrates an advantageous ecological niche for MR-1 in competing for favorable carbon sources. Finally, although our standard Monod model reasonably well described MR-1 growth data, its results showed some lack-of-fit with statistical analysis (Table S1). Such a discrepancy was possibly due to the model's simplification and to measurement noises. In this study, the kinetic model represents a compromise between complexity and practical simplicity.

**Table 1:** Parameters estimated in the empirical Monod model.

Symbols	Notation	Unit	Value
μ_max,L_	Maximum specific growth rate using lactate	h^−1^	0.57±0.11
μ_max,P_	Maximum specific growth rate using pyruvate	h^−1^	0.14±0.02
μ_max,A_	Maximum specific growth rate using acetate	h^−1^	0.13±0.02
*Y*_X/L_	Apparent biomass yield coefficient from lactate	g DCW/mol lactate	17.0±1.3
*Y*_X/P_	Apparent biomass yield coefficient from pyruvate	g DCW/mol pyruvate	16.7±1.3
*Y*_X/A_	Apparent biomass yield coefficient from acetate	g DCW/mol actate	11.1±4.7
*K*_s,l_	Monod lactate saturation constant	mM	19.4±7.9
*K*_s,p_	Monod pyruvate saturation constant	mM	19.4±8.1
*K*_s,a_	Monod acetate saturation constant	mM	10.1±2.2
*k*_al_	Acetate production coefficient from lactate	L• (h•g DCW)^−1^	0.71±0.06
*k*_pl_	Pyruvate production coefficient from lactate	L• (h•g DCW)^−1^	0.45±0.04
*k*_ap_	Acetate production coefficient from pyruvate	L• (h•g DCW)^−1^	0.94±0.08
*k*_e_	Endogenous metabolism rate constant	h^−1^	0.013±0.016
*t_L_*	Lag time in growth	h	7.10±0.01

### Link kinetic model to FBA

**Figure 3:**
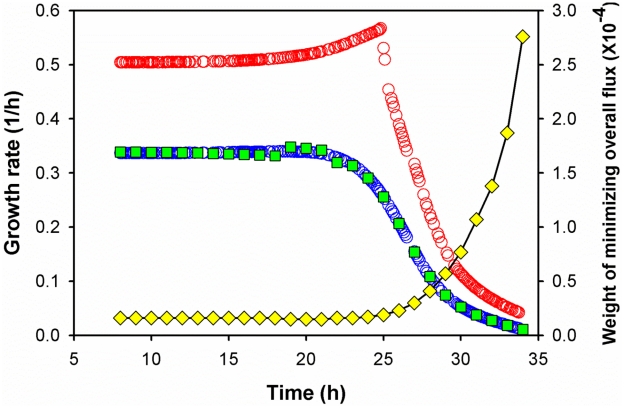
Prediction of growth rates (h^−1^). Blue ○: growth rate determined by the Monod model. Red ○: dFBA prediction using the objective function (maximization of growth rate). Green □: dFBA prediction using dual-objective functions (maximization of growth rate and minimization of overall flux). Yellow ◊: the weight of the dual-objective functions that predicted the measured growth rates. Note: the summation of the square of fluxes (∑*v^2^*) was a very large number (total 774 fluxes), so the magnitude of weight *w* was small.

To resolve the flux dynamics, the static optimization approach (SOA) divided the cultivation phase into numerous pseudo-steady states so that a conventional genome-scale MR-1 framework (iSO783, containing 774 reactions and 634 metabolites) was able to calculate the flux distributions [17] in each five-minute time interval. Such dFBA model consisted of ∼400 mini-FBAs. To avoid repeated and tedious measurements of biomass and metabolite concentrations for each mini-FBA, we used the Monod model to determine the inflow/outflow fluxes of lactate, acetate, and pyruvate in each time interval. The mini-FBAs could be resolved by an objective function of “maximizing growth rate”, but this function severely overestimated the actual biomass growth (Figure 3). To account for the suboptimal metabolic features [7], [8], [9], we used a dual-objective function in dFBA: a combination of “maximize growth rate” and “minimize overall flux”. By appropriately weighing both objectives, we explored the trade-offs between optimal cell growth and minimal enzyme usage. Specifically, the Monod model determined the transient growth rate for each time interval, which tuned the weights in the dual-objective functions for the mini-FBAs so that the biomass growth curve simulated by dFBA was in agreement with experimental observations. Figure 3 showed that the optimal dual-objective function in mini-FBAs was time-dependent. In general, these dual-objective functions were invariable before the carbon substrate switched from lactate to acetate/pyruvate. When lactate became scarce, the weight of “minimizing overall flux” in the objective function increased significantly, indicating an intracellular reduction of enzyme synthesis and minimization of intracellular fluxes.

### Dynamic flux distributions in MR-1

**Figure 4:**
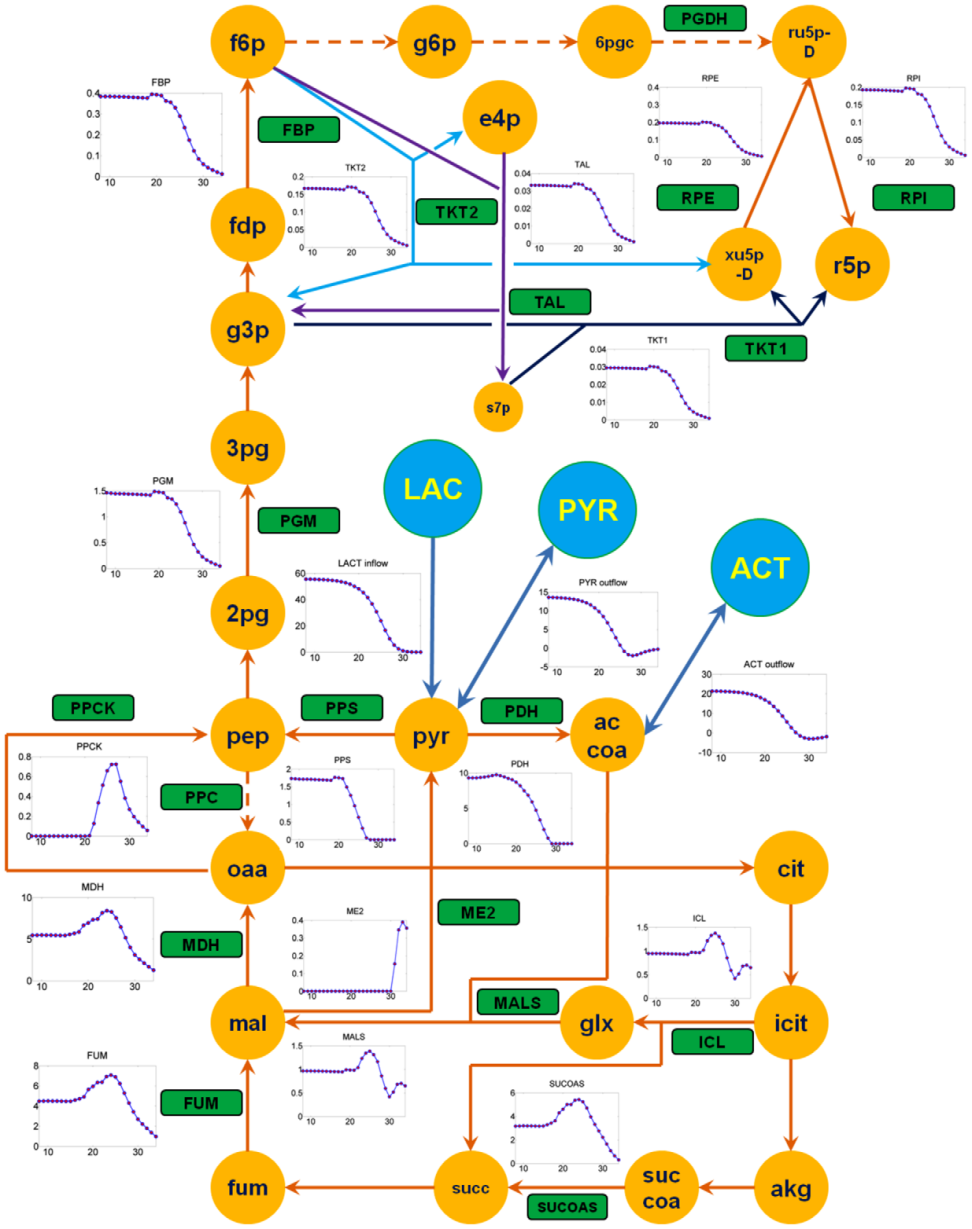
Dynamic flux distributions (unit: mmol/g DCW/h) in central metabolic pathways. The yellow filled cycles are intracellular metabolites; the blue filled cycles are substrates and extracellular metabolites (LAC: extracellular lactate, PYR: extracellular pyruvate, ACT: extracellular acetate); the dashed lines indicate inactive pathways; the green filled boxes are reactions listed in iSO783. All the abbreviations refer to iSO783 [7].

The dynamic flux distributions in MR-1 were calculated using the bi-level optimization (Figure 4). The carbon flows to extracellular acetate and pyruvate were high when lactate was sufficient (∼33% and ∼25% of the lactate uptake flux before the carbon substrate switch, respectively). Fluxes into the gluconeogenesis pathway, reductive PP pathway, and ED pathway were mainly for biomass synthesis, and remained approximately constant during the exponential growth phase. In the middle log phase (22∼25 hours), when the growth rate reached the maximum, fluxes in the oxidative TCA cycle reached a peak (e.g., ∼6 mmol/g DCW/h for succinyl-CoA synthetase) to generate energy and building blocks. When lactate became scarce (25∼30 hrs), MR-1 had to utilize its waste metabolites (acetate and pyruvate). During this metabolic shift, most intracellular fluxes started to decrease. In the late log growth phase (30∼34 hrs), it was also observed that the glyoxylate shunt was up-regulated compared to TCA cycle fluxes after acetate became the main carbon source for MR-1 growth. The glyoxylate shunt reduced the oxidation of carbon substrate for CO_2_ production by diverting the carbon flow into a shorter metabolic route. The glyoxylate shunt activity was further confirmed by *in vitro* enzyme assays at both the mid-log phase (malate synthase activity was 0.18±0.11 µmol/g DCW/min) and the late-log phase (malate synthase activity was 0.37±0.17 µmol/g DCW/min).

### Simulation of dynamic ^13^C-labeling in proteinogenic amino acids

**Figure 5:**
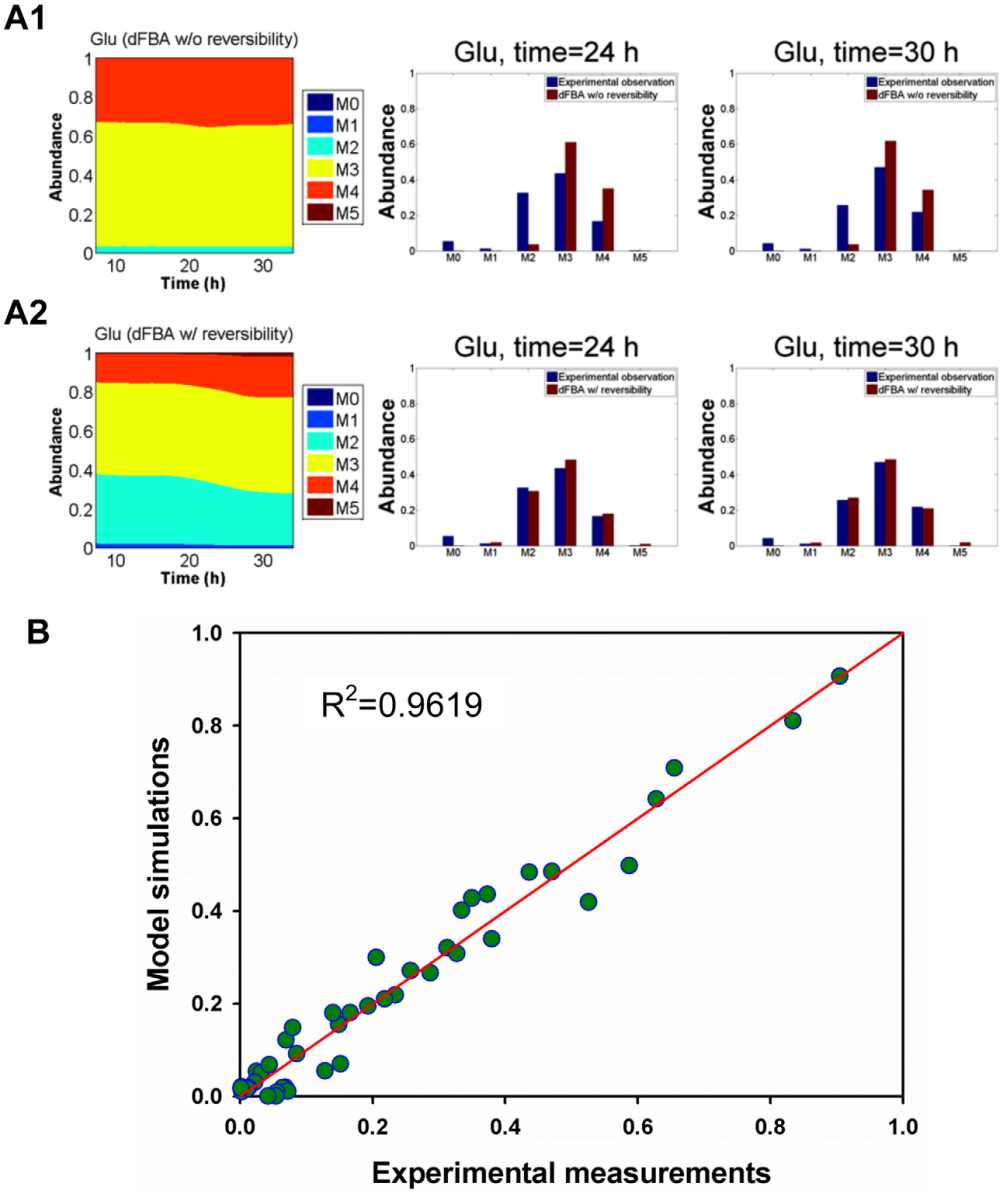
Experimentally observed and simulated isotopomer labeling patterns [M-57]^+^ in proteinogenic amino acids. The standard error for GC-MS measurement was below 0.02. **A1**: dynamic isotopomer simulation for glutamate from dFBA without considering reaction reversibility (dFBA w/o reversibility). **A2**: dynamic isotopomer simulation for glutamate from dFBA considering reaction reversibility (dFBA w/ reversibility). Bar plot: comparison of experimentally observed isotopomer labeling to simulated isotopomer labeling patterns of glutamate (**A1**: without considering reaction reversibility; **A2**: considering reaction reversibility). **B**: The model fitting of the isotopomer labeling data of five key amino acids (Ala, Gly, Ser, Asp, and Glu) at t = 24 and 30 h.

In ^13^C-labeled tracer experiments, dFBA can be used to predict the isotopomer dynamics in slow turnover metabolites, such as proteinogenic amino acids. During MR-1 growth with [3-^13^C] lactate, the dynamic metabolism led to variations of labeling patterns in intracellular metabolites (biomass precursors) so that the isotopic labeling in proteinogenic amino acids was continuously changing during cell growth [18]. Here, we simulated the time-integrative labeling patterns in proteinogenic amino acids based on fluxes from dFBA. The predicted isotopomer labeling patterns of five proteinogenic amino acids (Ala, Ser, Glu, Asp, and Gly, at t = 24 and 30 h) are illustrated in Figure 5A and Figure S1. Compared with the experimental measurements, the labeling patterns predicted by dFBA are consistent with the measured labeling patterns, but some lack-of-fit still exists. One of the limitations of the FBA model is that the intracellular pathway is treated as unidirectional, so the effect of exchange fluxes on isotopomer data is neglected. Considering that some *in vivo* reactions could be bi-directional, we implemented exchange coefficients for four pathways (e.g., the anaplerotic pathway: pyruvate→malate) in the model to improve the simulation of ^13^C-labeling (Table 2). After introduction of the exchange coefficients, the measured and the simulated isotopomer data for proteinogenic amino acids matched (R^2^ = 0.9619, Figure 5B).

**Table 2:** Exchange coefficients for key metabolic pathways of MR-1.

Pathways	Abbreviation	Exchange coefficients	Confidence intervals
Malate ↔ CO_2_+Pyruvate	ME2	0.862	[0.803 0.921]
Serine ↔ Glycine+C1 unit	GHMT	0.270	[0.062 0.477]
Glycine ↔ C1 unit+CO_2_	GLYCL	0.109	[0.061 0.157]
Succinate ↔ Succinyl-CoA	SUCOAS	0.944	[0.906 0.983]

## Discussion

dFBA models have been developed to describe the dynamic metabolism of *E.coli* [10], *Saccharomyces cerevisiae* [19], *Lactococcus lactis* [12], and even for a more complicated coculture system of *E.coli* and *Saccharomyces cerevisiae* [20]. In this study, we developed dFBA for analyzing metabolic states of *S. oneidensis* MR-1. The time-dependent inflow/outflow fluxes for dFBA can be constrained by either a Monod model or other empirical models (such as polynomial-fitting to the measurement data [21]). The Monod model is suitable to uncover kinetic properties of a scale-up bioprocess and empowers the dFBA to correlate the bioprocess parameters (such as nutrient concentrations and inhibition coefficients) with intracellular metabolism analysis. The integration of the Monod model and dFBA can decipher and predict cell metabolisms in response to batch fermentation conditions.

To describe biological realities, a physiologically reasonable objective function is important for FBA. For *E.coli* metabolism, 11 objective functions have been systemically investigated under different cultivation conditions [7]. It turns out that no single objective function can describe metabolic states accurately for all conditions. A recent study of MR-1 indicated that futile cycles may be operational, in which less energetically efficient enzymes are expressed at higher levels than their counterparts and decrease biomass yield [17]. Such suboptimal metabolic features in MR-1 make the conventional objective functions difficult to use in predicting actual cell physiology. To bridge the gap between the *in silico* simulations and experimental observations, we assigned dual-objective functions to resolve mini-FBAs. Using dual-objective functions, dFBA accurately predicted the elevated flux ratio of the glyoxylate shunt (represented by malate synthase activity) to the oxidative TCA cycle (represented by fumarase activity) when acetate started to be used as the main carbon substrate (Figure S2). The up-regulation of the glyoxylate shunt and down-regulation of the oxidative TCA pathways were consistent with a previous ^13^C-metabolic flux analysis of MR-1 [18]. In comparison, this transient metabolic shift in the glyoxylate shunt could not be captured by a single objective function, such as maximal biomass growth. Moreover, our dFBA results showed the weight of the two objective functions remained relatively constant when lactate was sufficient. At the early stage of MR-1 growth, such a pseudo-steady state has been experimentally verified by previous isotopomer-based analysis [18]. Under nutrient scarcity conditions, MR-1 metabolism may reduce synthesis and usage of enzymes to achieve a compromise between minimization of general physiological activities and maintenance of essential cellular functions [22].

The dFBA model can also simulate time-dependent isotopomer enrichment in proteinogenic amino acids. In turn, the isotopomer results (Figure 5) can be used to validate and improve the dFBA model predictions. For example, our dFBA model predicted low fluxes through malic enzyme during the exponential growth because these pathways may reduce biomass production, while the genetic analysis indicates a high functionality of malic enzyme [17]. In the dynamic isotopomer simulations, we found that the fitting of isotopomer labeling patterns was significantly improved by introducing the bi-directional fluxes through the pathway Malate ↔ CO_2_+Pyruvate, while keeping the net flux minimal. Such reversible reactions suggest metabolic flexibility. The activity of malic enzyme was also confirmed by *in vitro* enzyme assays at both the mid-log phase (malic enzyme activity was 0.90±0.18 µmol/g DCW/min) and late-log phase (malic enzyme activity was 1.73±0.81 µmol/g DCW/min).

Proteinogenic amino acids are abundant in biomass and can easily be measured by GC-MS. Complementing this instrumental data, ^13^C-metabolic flux analysis (MFA) offer analytic insight into the cell metabolisms in fermentation processes [3], [23], [24]. However, the turnover rate of protein is much slower than that of intracellular metabolites, so ^13^C-MFA is useful only for analyzing the steady-state central metabolism. To perform ^13^C-MFA of dynamic flux distributions, the fast turnover metabolites have to be extracted and analyzed at multiple time points [1], [3], which requires significant sampling efforts and high-sensitivity analytical measurement of low-abundance/unstable metabolites. Moreover, the calculation of dynamic fluxes with isotopomer data formulates an inverse nonlinear optimization problem, which is computationally challenging. Due to insufficient methods for analyzing low abundance metabolites [25], as well as limitations in computational algorithms, dynamic ^13^C-MFA cannot resolve the flux distributions in a large-scale metabolic network. To overcome these difficulties, this study illustrates a proof-of-concept approach that exploits the synergy between proteinogenic-amino-acid-based ^13^C-MFA and genome-scale dynamic flux balance analysis.

In our dFBA, the Monod model is solved first independently of the FBA. As an alternative approach, we also tested to integrate the kinetic models with FBA (integrative Flux Balance Analysis, iFBA). iFBA simultaneously optimizes the kinetic model parameters and solves the dynamic cell metabolism in MR-1 (Supplementary Text S1). We found that iFBA also requires a dual objective function, a weighted combination of “maximizing growth rate” and “minimizing overall flux”, to improve the model accuracy, similar to the dFBA approach. Such results indicate that it is difficult to use a single objective to describe the flux states under all growth conditions, while the time-dependent trade-off objective functions are effective for analyzing the dynamic suboptimal metabolism.

In conclusion, as in other FBA studies, the dFBA framework proposed in this study links macroscopic bioprocess variables (such as nutrient concentrations) to microscopic intracellular metabolism analysis. It predicts metabolic responses under dynamic culture conditions, and reveals the impact of the kinetic parameters (such as μ_max_) on intracellular flux distributions. Furthermore, dFBA can identify the objective functions that are possibly used by microorganisms in adaption to environmental variations. Finally, by simulating and comparing the isotopomer labeling patterns of different metabolites, the proposed dFBA framework can potentially improve dynamic flux resolutions by incorporating the isotopomer data from labeled proteinogenic amino acids.

## Materials and Methods

### Culture conditions, analytical methods, and isotopomer analysis

*S. oneidensis* MR-1 (ATCC 70050) was first grown in LB medium in shake flasks overnight. A 0.1% inoculum volume was then cultured into modified MR-1 defined medium [26] in shake flasks (100 mL culture for each of triplicate experiments, shaken at 150 rpm) at 30°C. The initial carbon source was ∼30 mM lactate. The growth curve was monitored by dried biomass weight. The concentrations of lactate and acetate in the medium were measured using enzyme kits (r-Biopharm, Darmstadt, Germany). The concentration of pyruvate in the medium was measured with the enzyme assay developed by Marbach and Weli [27].

To analyze the activity of malate synthase and malic enzyme, samples were taken at early middle log phase (biomass of MR-1 ∼0.08 g/L) and late log phase (biomass of MR-1 ∼0.23 g/L). The harvested cells were centrifuged and re-suspended in 100 mM Tris buffer. The samples were then ultra-sonicated for 5 min to release the enzymes. Malate synthase activity was gauged based on the reaction of CoASH with DTNB (Acetyl-CoA+glyoxylate→Malate+CoASH; CoASH+DTNB→CoA-TNB+TNB), as described by Dixon and Kornberg [28]. In general, 20 µL acetyl-CoA (5 mM), 10 µL DTNB (10 mM), 50 µL cell extract, and 500 µL of a solution containing 0.1 M potassium phosphate and 10 mM MgCl_2_ were mixed with water. The mixture was then added with 20 µL 100 mM glyoxylate. The difference in absorbance at OD_412_ before and after glyoxylate addition reflected the activity of malate synthase, in which one unit ΔOD_412_ corresponded to 70.6 nmol of TNB produced (in a 1 mL reaction solution). Furthermore, the activity of malic enzyme was detected based on increased absorbance at 340 nm due to the reduction of NAD^+^ to NADH [29]. In brief, 400 µL 250 mM Tris HCl, 20 µL 50 mM MnCl_2_, 25 µL 40 mM NH_4_Cl, 100 µL 1 M KCl, 50 µL 20 mM NAD^+^, 100 µL 100 mM malate, and 50 µL cell extract were mixed with water (1 mL reaction solution). The change in absorbance at OD_340_ reflected the activity of malic enzyme.

In the labeling experiment, MR-1 was first grown overnight in the LB medium in shake flasks. A 0.1% inoculum volume was then cultured into 100 mL of modified MR-1 defined medium at 30°C, with the initial carbon source as 30 mM [3-^13^C] lactate (purity>98%) purchased from Cambridge Isotope Laboratories, Inc. (Andover, MA). The biomass was harvested at ∼24 h (before lactate was depleted) and ∼30 h (after the substrate had switched from lactate to waste products). To analyze the labeling pattern of proteinogenic amino acids, we hydrolyzed the biomass with 6 M HCl at 100°C. The isotopic analysis of proteinogenic amino acids was performed using a GC-MS based TBDMS method, as previously described [30], [31], [32]. Ions of [M-57]^+^ (unfragmented amino acid) were used for the ^13^C-simulations [33].

### Monod model development

A multiple-substrate Monod model was developed to describe the cell growth, lactate consumption and secretion, and reuse of waste products (acetate and pyruvate).
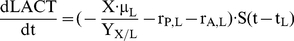
(1)

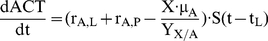
(2)

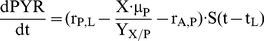
(3)

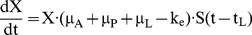
(4)where *X* is biomass (g DCW/L); *LACT*, *ACT*, and *PYR* are lactate, acetate, and pyruvate concentrations (mmol/L), respectively; *μ_L_*, *μ_A_*, and *μ_P_* are the specific growth rates (h^−1^) on lactate, acetate, and pyruvate, respectively; *k_e_* is the endogenous metabolism rate constant (h^−1^); *Y_X/L_*, *Y_X/A_*, and *Y_X/P_* are the biomass yield coefficients (g DCW/mol substrate) of lactate, acetate, and pyruvate respectively; *r_P,L_* and *r_A,L_* are the production rates (mmol/L/h) of acetate and pyruvate from lactate, respectively. *r_A,P_* is the production rates (mmol/L/h) of acetate from pyruvate. *S(t−t_L_)* is the dimensionless unit-step time delay function (S = 0 when t<t_L_; S = 1 when t = t_L_) which described the lag phase after inoculation.

The specific cell growth rate was described by Monod equations:
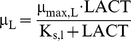
(5)

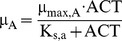
(6)

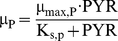
(7)where *μ_max,L_*, *μ_max,A_*, and *μ_max,P_* are the maximal growth rates (h^−1^) for fully aerobic growth on lactate, acetate, and pyruvate, respectively; and *K_s,l_*, *K_s,a_*, and *K_s,p_* are Monod constants (mmol/L) for lactate, acetate, and pyruvate, respectively. The acetate and pyruvate production rates are assumed to be proportional to the biomass [16], as expressed below:
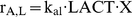
(8)

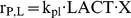
(9)

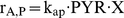
(10)where *k_al_* and *k_pl_* are rate constants of acetate and pyruvate production, respectively (L• (h•g DCW)^−1^). *k_ap_* is the rate constant of acetate production from pyruvate (L• (h•g DCW)^−1^).

The kinetic model (Equations 1∼10) contained 14 kinetic parameters. To estimate the model parameters, an ordinary least squares (OLS) method [34] was applied to solve the inverse problem. OLS aimed to minimize the residual sum of the squares (*R*) between model simulations and experimental measurements, expressed as
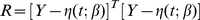
(11)where *η* represents four dependent variables simulated by the kinetic model; *β* represents the vector of the parameters to be estimated; and *Y* is the vector of the measured value of the dependent variables. Since the scales of the dependent variables were different (e.g., the scale of the biomass measurement was <1 g/L, while the lactate measurement >10 mmol/L), the direct application of OLS would overemphasize the fitting of dependent variables with large scales. Therefore, we normalized dependent variables by the corresponding maximum concentrations measured in the experiments.

The “ode23” command in MATLAB (R2009a, Mathworks) was used to solve ODEs, and the “fmincon” command was used to find suitable settings of the parameters. Figure S3 is the histogram of normalized model residuals. The standard deviations of the parameters were derived from a bootstrap analysis, in which the experimental measurements were randomly re-sampled 1000 times and the corresponding parameters were simulated with the same parameter estimation approach. The 1000 re-sampling was found to be adequate since the variation of parameters converged to within a desired tolerance of 0.1%. The MATLAB code of parameter estimation in the Monod model was attached in Dataset S1.

### Bi-level dFBA study

The growth phase was divided into 408 pseudo-steady-state intervals with instantaneous transitions between the two adjacent intervals [11]. At each pseudo-steady state (∼five minutes) [35], a mini-FBA was formulated by a dual-objective function comprised of “maximizing the growth rate” and “minimizing overall flux”; and inflow/outflow fluxes (for lactate, acetate, and pyruvate) derived from the Monod model. The inflow/outflow fluxes were calculated from:
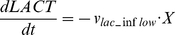
(12)

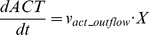
(13)

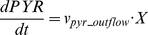
(14)where *v_lac_inflow_*, *v_act_outflow_*, and *v_pyr_outflow_* are transient lactate inflow flux, acetate outflow flux, and pyruvate outflow flux, respectively.

At each pseudo steady state, the mini-FBA followed a bi-level optimization framework similar to ObjFind [36]. The internal optimization was an FBA with a combined objective function in which the weighting factor of “minimizing overall flux” ranged from zero to one. The difference between the transient growth rate simulated from the FBA and that derived from the Monod model was minimized in the external optimization, by tuning the weighting factor in the combined objective function. The bi-level optimization determined a trade-off between maximizing growth rate and maximizing enzyme efficiency under the specified growth conditions. The bi-level optimization was formulated as:
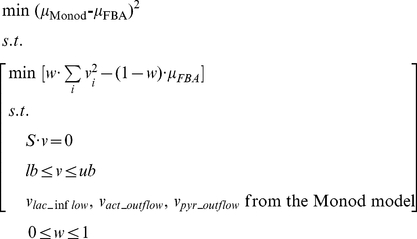
(15)where *μ_monod_* and *μ_FBA_* are transient growth rates derived from the Monod model and the dFBA study, respectively; *w* is the weight of “minimizing overall flux” in the combined objective function; *v* is the vector of the intracellular fluxes; *S* is the stoichiometry matrix; *lb* and *ub* are the lower and upper boundaries for intracellular flux.

The internal optimization was a typical quadratic programming (QP) problem and was solved using the CPLEX solver in the TOMLAB optimization toolbox (TOMLAB Optimization Inc, Seattle, WA) within MATLAB (R2009a). The external optimization problem (i.e., search for weight) was solved by a grid search. Since the QP problem in this study was convex, the locally searched results were also the global solutions [37]. The MATLAB code of bi-level dFBA was attached in Dataset S1.

### Simulation of dynamic ^13^C-labeling in proteinogenic amino acids

Our previous ^13^C-MFA study of MR-1 showed that the labeling patterns in its proteinogenic amino acids changed during the late-stage of batch growth [18]. Using the dynamic flux distributions from dFBA, we could now simulate dynamics of isotopomer labeling patterns in proteinogenic amino acids using the algorithm below.
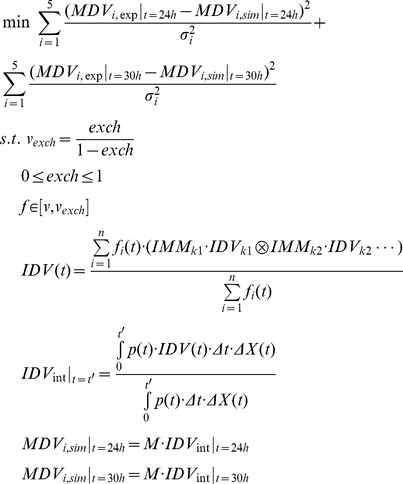
(16)


Since FBA neglects flux reversibility, we implemented exchange coefficients *exch* to account for the reversibility of four key metabolic pathways (ME2, GHMT, GLYCL, and SUCOAS in iSO783, Table 2). In Equation 16, *v_exch_* is the vector of exchanged fluxes in the reversible reactions; *v* is the vector of the transient fluxes at the *t′* interval, simulated from the mini-FBA; *p(t)* are fluxes to proteinogenic amino acids at each time interval; *Δt* is the scale of a pseudo steady state (5 min); *ΔX(t)* is the biomass produced at each time interval; *IMM* is the isotopomer mapping matrices describing the carbon atoms transitions from reactants to products in a reaction; *IDV(t)* are the isotopomer distribution vectors of transient intracellular metabolites at each time interval, which is calculated based on the different labeling patterns of precursors from *n* pathways; *IDV_int_*|*_t = t′_* are the isotopomer distribution vectors in proteinogenic amino acids at the end of the *t′* time interval; *MDV_i,sim_* and *MDV_i,exp_* are the mass distribution vectors for each of the five proteinogenic amino acids, as simulated and as measured by GC-MS, respectively; *M* is the matrix for converting *IDV* to *MDV*; σ_i_ is the standard deviation of the GC-MS measurement (error ∼2%), which is assumed to be constant in this study. The carbon transitions in the reactions involved were given in Supplementary Text S2, and the SBML file for the metabolic model of *Shewanella oneidensis* MR-1 was provided in Supplementary Text S3. The “fmincon” command in MATLAB was used to find the exchange coefficients, and the “nlparci” command in MATLAB (Dataset S1) was used to find the asymptotic confidence intervals of the exchange coefficients. These exchange coefficients significantly improved dFBA simulation of the labeling patterns in proteinogenic amino acids.

## Supporting Information

Dataset S1 MATLAB codes used for mathematical modeling.
(RAR)

Figure S1 Experimental observed and simulated isotopomer labeling patterns [M-57]^+^ in key proteinogenic amino acids. The standard error for GC-MS measurement was ∼0.02. Area plot: dynamic isotopomer simulation (case 1: simulation without considering reaction reversibility; case 2: simulation considering reaction reversibility). Bar plot: comparison of experimental data to simulated isotopomer labeling patterns (case 1: without considering reaction reversibility; case 2: considering reaction reversibility).
(DOC)

Figure S2 Flux ratio of malate synthase (MALS) and fumarase (FUM) in dynamic metabolism of *Shewanella oneidensis* MR-1. Blue ▪: time profiles of flux ratio using “maximizing growth rate” as the objective function in dFBA; red ▴: time profiles of flux ratio using dual-objective function in dFBA: a combination of “maximize growth rate” and “minimize overall flux”. The entire growth of MR-1 was divided into three phases. In phase I, lactate was mainly used as the carbon substrate. In phase II, lactate, acetate and pyruvate were used as the carbon substrates. In phase III, acetate was used as the carbon substrate.
(DOC)

Figure S3 Histogram of normalized Monod model residuals.
(DOC)

Table S1 Lack-of-fit test for the Monod model.
(DOC)

Text S1 Framework of integrative Flux Balance Analysis (iFBA).
(DOC)

Text S2 Reactions involved in ^13^C-labeling simulations.
(DOC)

Text S3 SBML file of the metabolic model for *Shewanella oneidensis* MR-1.
(DOC)

## References

[1] Wahl SA, Nöh K, Wiechert W (2008). ^13^C labeling experiments at metabolic nonstationary conditions: an exploratory study.. BMC Bioinformatics.

[2] Wiechert W, Nöh K (2005). From stationary to instationary metabolic flux analysis.. Adv Biochem Eng Biotechnol.

[3] Rühl M, Zamboni N, Sauer U (2010). Dynamic Flux Responses in Riboflavin Overproducing Bacillus subtilis to Increasing Glucose Limitation in Fed-Batch Culture.. Biotechnol Bioeng.

[4] Kovárová-Kovar K, Egli T (1998). Growth kinetics of suspended microbial cells: from single-substrate-controlled growth to mixed-substrate kinetics.. Microbiol Mol Biol Rev.

[5] Varma A, Palsson BO (1994). Stoichiometric flux balance models quantitatively predict growth and metabolic by-product secretion in wild-type Escherichia coli W3110.. Appl Environ Microbiol.

[6] Orth JD, Thiele I, Palsson BØ (2010). What is flux balance analysis?. Nat Biotechnol.

[7] Schuetz R, Kuepfer L, Sauer U (2007). Systematic evaluation of objective functions for predicting intracellular fluxes in *Escherichia coli*.. Mol Syst Biol.

[8] Burgard AP, Pharkya P, Maranas CD (2003). Optknock: a bilevel programming framework for identifying gene knockout strategies for microbial strain optimization.. Biotechnol Bioeng.

[9] Segrè D, Vitkup D, Church G (2002). Analysis of optimality in natural and perturbed metabolic networks.. Proc Natl Acad Sci U S A.

[10] Meadows AL, Karnika R, Lama H, Forestella S, Snedecor B (2010). Application of dynamic flux balance analysis to an industrial Escherichia coli fermentation.. Metab Eng.

[11] Mahadevan R, Edwards JS, Doyle FJ (2002). Dynamic flux balance analysis of diauxic growth in Escherichia coli.. Biophys J.

[12] Oddonea GM, Millsb DA, Block DE (2009). A dynamic, genome-scale flux model of Lactococcus lactis to increase specific recombinant protein expression.. Metab Eng.

[13] Myers CR, Nealson KH (1988). Bacterial manganese reduction and growth with manganese oxide as the sole electron acceptor.. Science.

[14] Tiedje JM (2002). *Shewanella*- the environmentally versatile genome.. Nat Biotechnol.

[15] Logan BE, Murano C, Scott K, Gray ND, Head IM (2005). Electricity generation from cysteine in a microbial fuel cell.. Water Res.

[16] Tang YJ, Meadows AL, Keasling JD (2007). A kinetic model describing *Shewanella oneidensis* MR-1 growth, substrate consumption, and product secretion.. Biotechnol Bioeng.

[17] Pinchuk GE, Hill EA, Geydebrekht OV, Ingeniis JD, Zhang X (2010). Constraint-based model of Shewanella oneidensis MR-1 metabolism: a tool for data analysis and hypothesis generation.. PLoS Comput Biol.

[18] Tang YJ, Martin HG, Dehal PS, Deutschbauer A, Llora X (2009). Metabolic flux analysis of Shewanella spp. reveals evolutionary robustness in central carbon metabolism.. Biotechnol Bioeng.

[19] Hjersted JL, Henson MA, Mahadevan R (2007). Genome-scale analysis of Saccharomyces cerevisiae metabolism and ethanol production in fed-batch culture.. Biotechnol Bioeng.

[20] Hanly T, Henson M (2011). Dynamic flux balance modeling of microbial co-cultures for efficient batch fermentation of glucose and xylose mixtures.. Biotechnol Bioeng.

[21] Lequeux G, Beauprez J, Maertens J, Horen EV, Soetaert W (2010). Dynamic metabolic flux analysis demonstrated on cultures where the limiting substrate is changed from carbon to nitrogen and vice versa.. J Biomed Biotechnol.

[22] Holzhütter HG (2004). The principle of flux minimization and its application to estimate stationary fluxes in metabolic networks.. Eur J Biochem.

[23] Zamboni N, Fendt SM, Rühl M, Sauer U (2009). ^13^C-based metabolic flux analysis.. Nat Protoc.

[24] Antoniewicz MR, Kraynie DF, Laffend LA, González-Lergier J, Kelleher JK (2007). Metabolic flux analysis in a nonstationary system: fed-batch fermentation of a high yielding strain of *E. coli* producing 1,3-propanediol.. Metab Eng.

[25] Reaves M, Rabinowitz J (2011). Metabolomics in systems microbiology.. Curr Opin Biotechnol.

[26] Tang YJ, Laidlaw D, Gani K, Keasling JD (2006). Evaluation of the effects of various culture conditions on Cr(VI) reduction by *Shewanella oneidensis* MR-1 in a novel high-throughput mini-bioreactor.. Biotechnol Bioeng.

[27] Marbach EP, Weli MH (1967). Rapid enzymatic measurement of blood lactate and pyruvate. Use and significance of metaphosphoric acid as a common precipitant.. Clin Chem.

[28] Dixon GH, Kornberg HL (1959). Assay methods for key enzymes of the glyoxylate cycle.. Biochem J.

[29] Spina J, Bright HJ, Rosenbloom J (1966). Purification and Properties of L-malic enzyme from Escherichia coli.. Biochemistry.

[30] Feng X, Banerjee A, Berla B, Page L, Wu B (2010). Mixotrophic and photoheterotrophic metabolisms in Cyanothece sp. ATCC 51142 under continuous light.. Microbiology.

[31] Feng X, Mouttaki H, Lin L, Huang R, Wu B (2009). Characterization of the Central Metabolic Pathways in Thermoanaerobacter sp. X514 via Isotopomer-Assisted Metabolite Analysis.. Appl Environ Microbiol.

[32] Feng X, Tang K-H, Blankenship RE, Tang YJ (2010). Metabolic flux analysis of the mixotrophic metabolisms in the green sulfur bacterium *Chlorobaculum tepidum*.. J Biol Chem.

[33] Wahl SA, Dauner M, Wiechert W (2004). New tools for mass isotopomer data evaluation in ^13^C flux analysis: mass isotope correction, data consistency checking, and precursor relationships.. Biotechnol Bioeng.

[34] Beck JV, Arnold KJ (1977). Parameter estimation.

[35] Stephanopoulos GN, Aristidou AA, Nielsen J (1998). Metabolic Engineering Principles and Methodologies.

[36] Burgard AP, Maranas CD (2003). Optimization-based framework for inferring and testing hypothesized metabolic objective functions.. Biotechnol Bioeng.

[37] Boyd S, Vandenberghe L (2004). Convex Optimization.

