# Supplementary material for: Integrating Flux Balance Analysis into Kinetic Models to Decipher the Dynamic Metabolism of Shewanella oneidensis MR-1
Source: PLoS Comput Biol. 2012 Feb 2;8(2):e1002376. doi: 10.1371/journal.pcbi.1002376 (PMC3271021; doi:10.1371/journal.pcbi.1002376)
Supplement: Figure S1 — Experimental observed and simulated isotopomer labeling patterns [M-57]+ in key proteinogenic amino acids. The standard error for GC-MS measurement was ∼0.02. Area plot: dynamic isotopomer simulation (case 1: simulation without considering reaction reversibility; case 2: simulation considering reaction reversibility). Bar plot: comparison of experimental data to simulated isotopomer labeling patterns (case 1: without considering reaction reversibility; case 2: considering reaction reversibility). (DOC) [file pcbi.1002376.s002.doc]

**
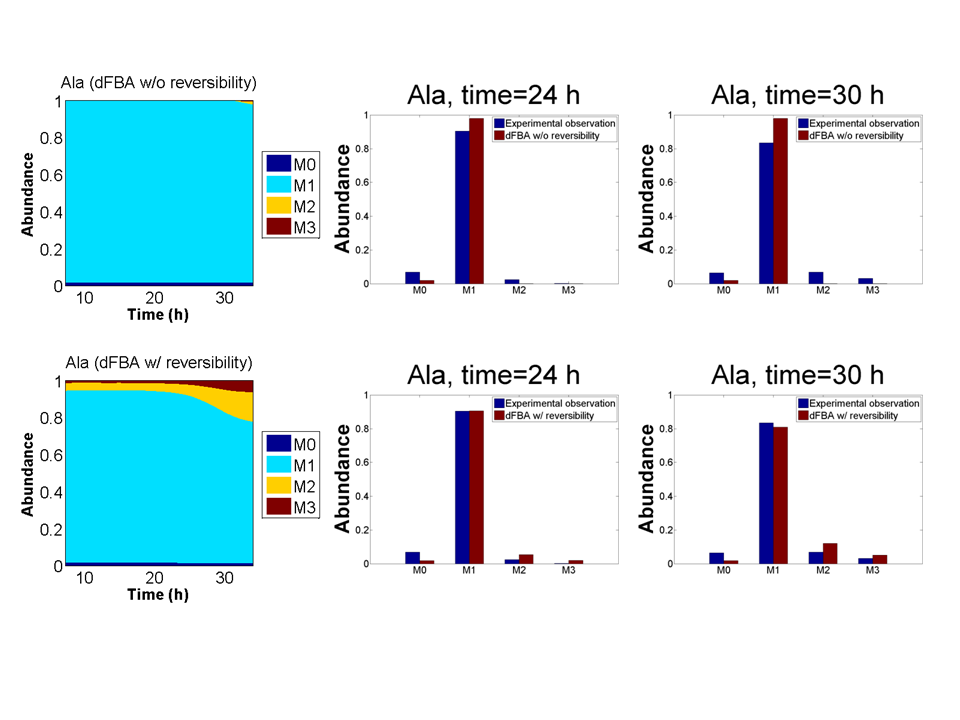
**

**
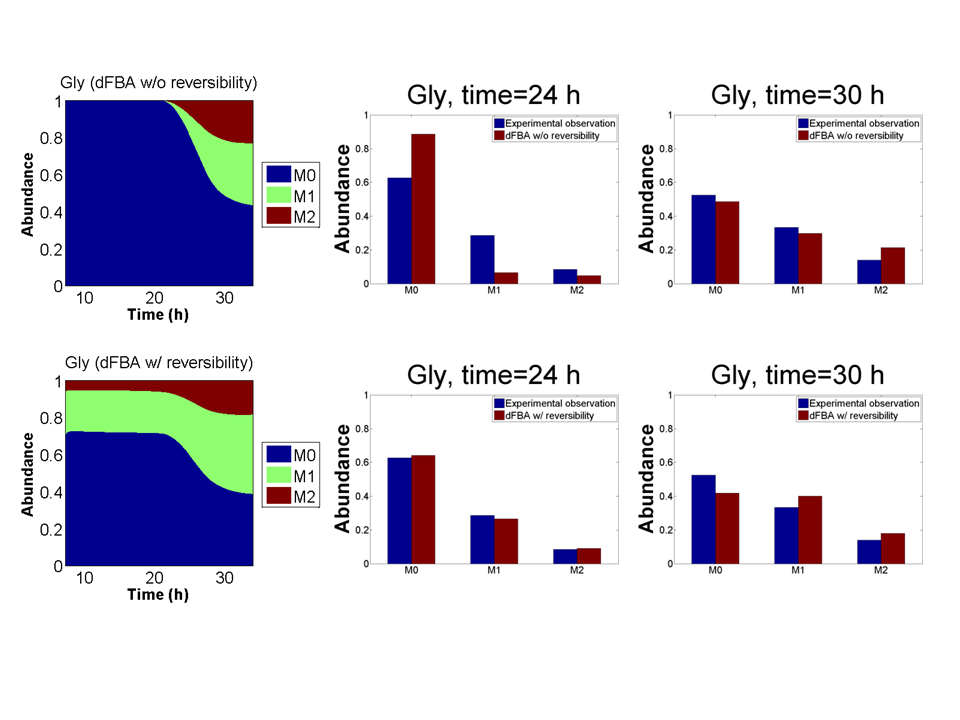
**

**
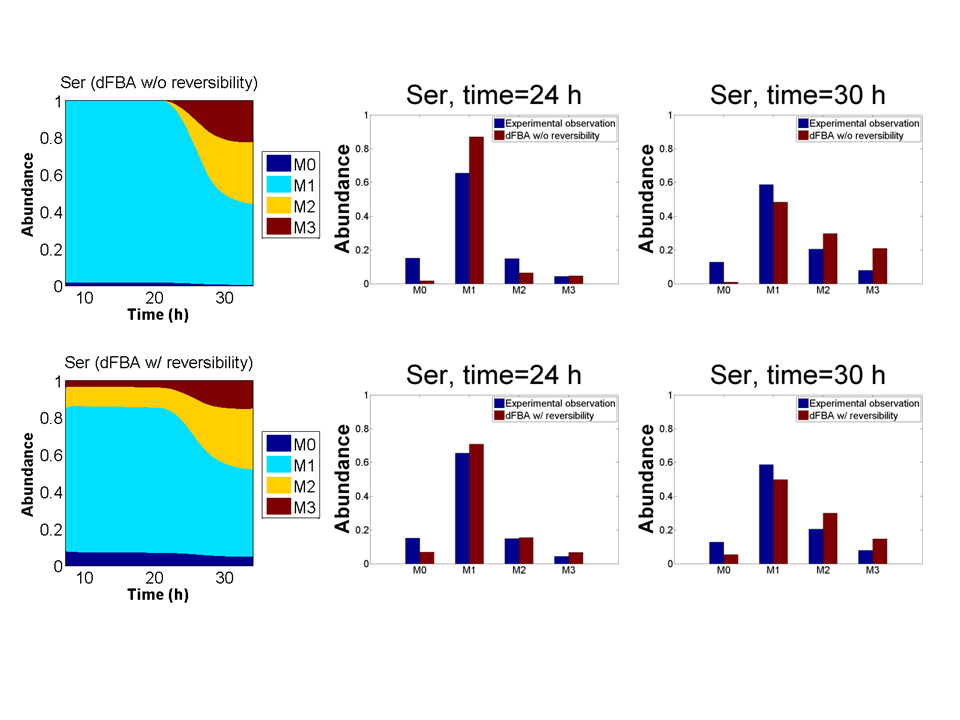
**

**
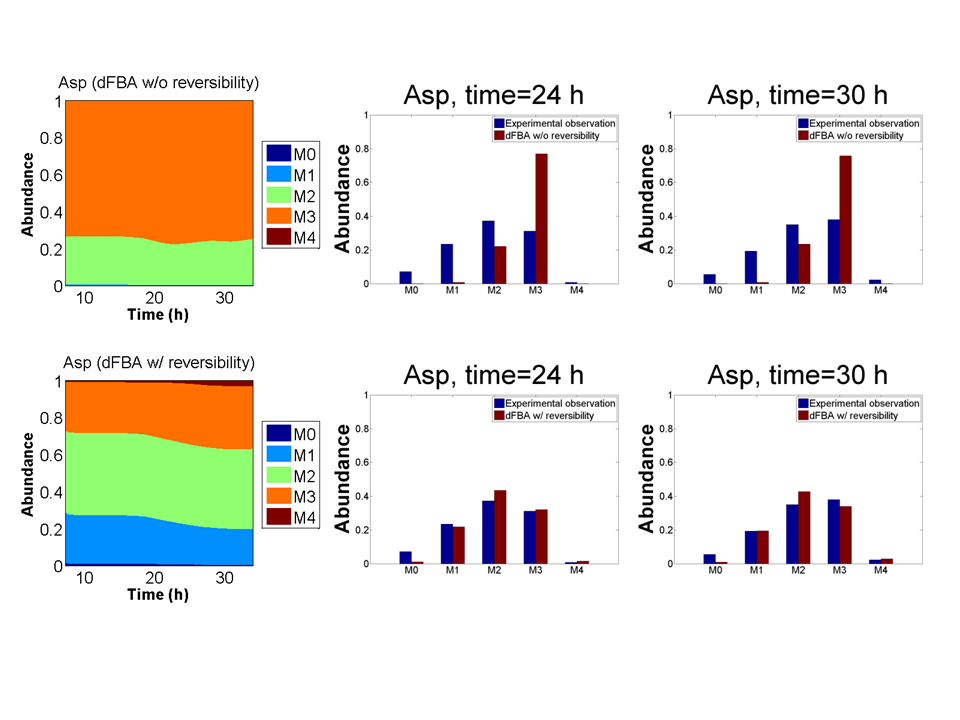
**

**Fig. S1** Experimental observed and simulated isotopomer labeling patterns [M-57]^+^ in key proteinogenic amino acids. The standard error for GC-MS measurement was ~0.02. Area plot: dynamic isotopomer simulation (case 1: simulation without considering reaction reversibility; case 2: simulation considering reaction reversibility). Bar plot: comparison of experimental data to simulated isotopomer labeling patterns (case 1: without considering reaction reversibility; case 2: considering reaction reversibility).
