# Supplementary material for: Integrating Flux Balance Analysis into Kinetic Models to Decipher the Dynamic Metabolism of Shewanella oneidensis MR-1
Source: PLoS Comput Biol. 2012 Feb 2;8(2):e1002376. doi: 10.1371/journal.pcbi.1002376 (PMC3271021; doi:10.1371/journal.pcbi.1002376)
Supplement: Figure S2 — Flux ratio of malate synthase (MALS) and fumarase (FUM) in dynamic metabolism of Shewanella oneidensis MR-1. Blue ▪: time profiles of flux ratio using “maximizing growth rate” as the objective function in dFBA; red ▴: time profiles of flux ratio using dual-objective function in dFBA: a combination of “maximize growth rate” and “minimize overall flux”. The entire growth of MR-1 was divided into three phases. In phase I, lactate was mainly used as the carbon substrate. In phase II, lactate, acetate and pyruvate were used as the carbon substrates. In phase III, acetate was used as the carbon substrate. (DOC) [file pcbi.1002376.s003.doc]

**
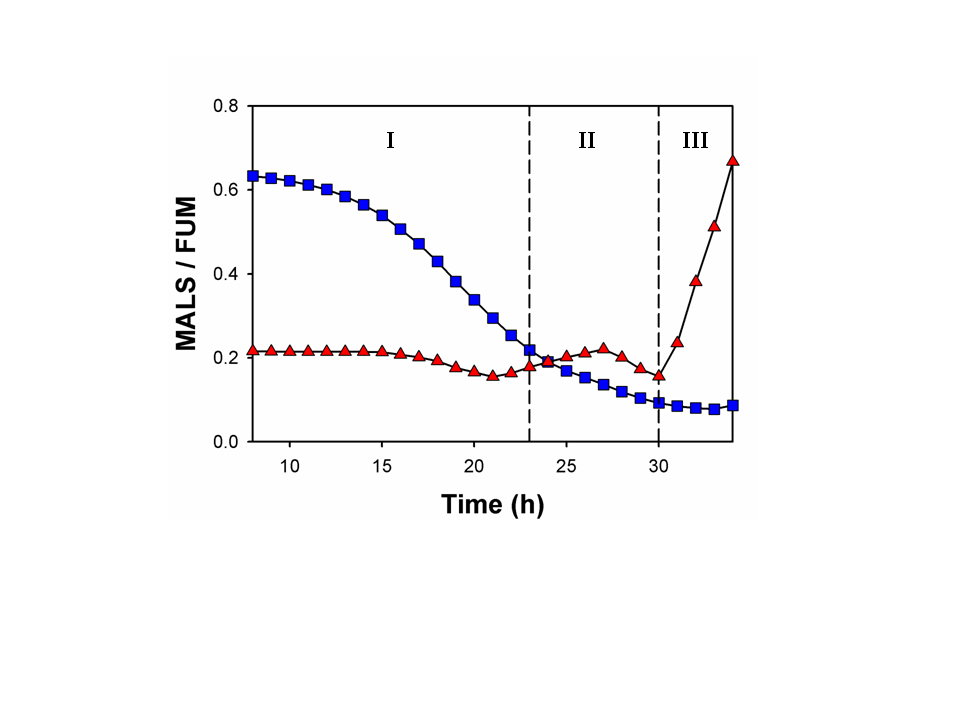
**

**Fig. S2** Flux ratio of malate synthase (MALS) and fumarase (FUM) in dynamic metabolism of *Shewanella oneidensis* MR-1. Blue ■: time profiles of flux ratio using “maximizing growth rate” as the objective function in dFBA; red ▲: time profiles of flux ratio using dual-objective function in dFBA: a combination of “maximize growth rate” and “minimize overall fluxes”. The entire growth of MR-1 was divided into three phases. In phase I, lactate was used as the carbon substrate. In phase II, lactate, acetate and pyruvate were used as the carbon substrates. In phase III, acetate was used as the carbon substrate.
