# Supplementary figures and images for: Integrating Flux Balance Analysis into Kinetic Models to Decipher the Dynamic Metabolism of Shewanella oneidensis MR-1
Source: PLoS Comput Biol. 2012 Feb 2;8(2):e1002376. doi: 10.1371/journal.pcbi.1002376 (PMC3271021; doi:10.1371/journal.pcbi.1002376)

**
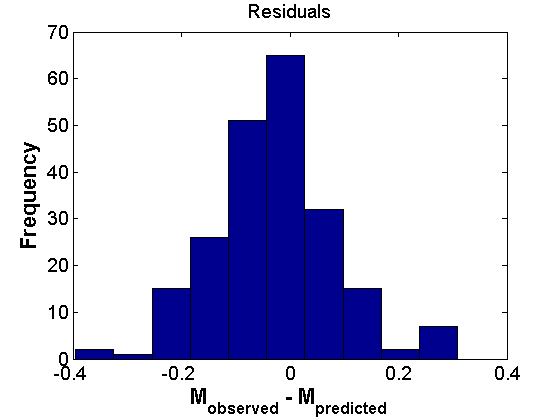
**

**Fig. S3** Histogram of normalized Monod model residuals.

Supplement: Figure S3 — Histogram of normalized Monod model residuals. (DOC) [file pcbi.1002376.s004.doc]
