# Supplementary material for: Integrating Flux Balance Analysis into Kinetic Models to Decipher the Dynamic Metabolism of Shewanella oneidensis MR-1
Source: PLoS Comput Biol. 2012 Feb 2;8(2):e1002376. doi: 10.1371/journal.pcbi.1002376 (PMC3271021; doi:10.1371/journal.pcbi.1002376)
Supplement: Table S1 — Lack-of-fit test for the Monod model. (DOC) [file pcbi.1002376.s005.doc]

**Table S1. Lack-of-fit test for the Monod model**

| **Model Name** | **Lack-of-fit sum of squares, SSLOF** | **Degree of freedom,**  ***df_1_*** | **Pure-error sum of squares, SSPE** | **Degree of freedom,**  ***df_1_*** | $\frac{SSLOF/df_{1}}{SSPE/df2}$ | ***F*(*df_1_*,*df_2_*)** |
| --- | --- | --- | --- | --- | --- | --- |
| Monod model | 2.064 | 58 | 0.813 | 144 | 6.303 | 1.390 |

**Notes:**

In order to test whether or not a model could fit the data well, we applied the lack-of-fit test. It partitioned the total sum of squares of residuals in the model (SSE) into two sources of variability: 1) the sum of squares from pure error variability (SSPE) and 2) the sum of squares from lack of fit (SSLOF), i.e. SSE=SSPE+SSLOF.

For testing the null hypothesis H0: the nonlinear model was adequate versus hypothesis Ha: the nonlinear model was inadequate, the test statistic is:$F=\frac{SSLOF/df_{1}}{SSPE/df2}$. For a specified α-level of significance, we rejected the null hypothesis if F > F_α_, where F_α_ is the α-level critical value (α=0.95 in this study) corresponding to an *F*(*df_1_*; *df_2_*) distribution. The *F*-test indicates our nonlinear model can be further improved by including new parameters. The improved kinetic model is presented in another manuscript under review.

**Reference:**

**Feng X**, Tang YJ, and Dolan K. “Construction of a parsimonious kinetic model to capture microbial dynamics via parameter estimation.” *Comput Chem Eng*. **Under review**.
