# Supplementary material for: Integrating Flux Balance Analysis into Kinetic Models to Decipher the Dynamic Metabolism of Shewanella oneidensis MR-1
Source: PLoS Comput Biol. 2012 Feb 2;8(2):e1002376. doi: 10.1371/journal.pcbi.1002376 (PMC3271021; doi:10.1371/journal.pcbi.1002376)
Supplement: Text S1 — Framework of integrative Flux Balance Analysis (iFBA). (DOC) [file pcbi.1002376.s006.doc]

**Text S1: Framework of integrative Flux Balance Analysis (iFBA)**

We have tested an alternative model framework: iFBA. The dynamic cultivation process was decomposed into numerous pseudo-steady-state time intervals. At each time interval, the inflow/outflow fluxes in the FBA were derived from the Monod equations; while the biomass increase during the time interval was predicted by mini-FBAs using proper objective functions. At the end of each time interval, the predicted biomass increase was incorporated into the Monod equations to estimate the metabolite and substrate concentrations at the next time interval. Then, we obtained the inflow/outflow fluxes of mini-FBA in the next time interval (t+∆t). To improve the model accuracy, iFBA employed a dual-objective function *w(i)*, a weighted combination of “maximizing growth rate” and “minimizing overall flux”. The time-dependent weight in the dual-objective function and the kinetic parameters in Monod equations were determined by minimizing the differences between iFBA predicted MR-1 growth kinetics and the experimentally measured data. The iFBA was formulated as below:

$$\begin{matrix} \min\text{R = [Y}^{\text{'}}\text{- η}^{\text{'}}\text{ ( t;β)]}^{\text{T}}\text{[Y}^{\text{'}}\text{- η}^{\text{'}}\text{ ( t;β)]} \\ s.t. \\ \left[\begin{matrix} X(0), LACT(0), ACT(0), PYR(0) \\ for i=1 : num\_timepo\mathrm{int} \\ t=i\cdot dt \\ v_{lac\_\inf low}(i)= f_{1}(LACT, ACT, PYR) \\ =-[\frac{\frac{\mu_{\max,L}\cdot LACT(i)}{K_{s,l}+LACT(i)}}{Y_{X/L}}-k_{pl}\cdot LACT(i)-k_{al}\cdot LACT(i)]\cdot S(t-t_{L}) \\ v_{act\_outflow}(i)= f_{2}(LACT, ACT, PYR) \\ =[-\frac{\frac{\mu_{\max,A}\cdot ACT(i)}{K_{s,a}+ACT(i)}}{Y_{X/A}}+k_{ap}\cdot PYR(i)+k_{al}\cdot LACT(i)]\cdot S(t-t_{L}) \\ v_{pyr\_outflow}(i)= f_{3}(LACT, ACT, PYR) \\ =[-\frac{\frac{\mu_{\max,A}\cdot ACT(i)}{K_{s,a}+ACT(i)}}{Y_{X/A}}+k_{ap}\cdot PYR(i)+k_{al}\cdot LACT(i)]\cdot S(t-t_{L}) \\ \left[\begin{matrix} \min[w(i)\cdot\sum_{i} v(i)_{_{i}}^{2}-(1-w(i))\cdot\mu(i)] \\ s.t. \\ S\cdot v(i)=0 \\ lb\leq v(i)\leq ub \\ v_{lac\_\inf low}(i), v_{act\_outflow}(i), v_{pyr\_outflow}(i) \\ w(i)= p_{1}\cdot\exp(p_{2}\cdot S(t-p_{3})) \end{matrix}\right] \\ X(i+1)=X(i)+\mu(i)\cdot X(i)\cdot dt \\ LACT(i+1)=LACT(i)+f_{1}\cdot X(i)\cdot dt \\ ACT(i+1)=ACT(i)+f_{2}\cdot X(i)\cdot dt \\ PYR(i+1)=PYR(i)+f_{3}\cdot X(i)\cdot dt \end{matrix}\right] \\ lb\leq\beta, p_{1}, p_{2}\leq ub \end{matrix}$$

The new symbols introduced in iFBA are as following: *num_timepoint* is the number of time intervals decomposed during the entire cultivation process, which is 408; *dt* is the time of each time interval, which is 1/12 h. *p_1_*, *p_2_* and *p_3_* are three parameters used to simulate the dynamic weighting factors in the dual objective function. The internal dFBA problem was solved using the CPLEX solver in TOMLAB optimization toolbox (TOMLAB optimization Inc, Seattle, WA) within MATLAB (R2009a). The external optimization problem (i.e. search for weight) was solved by SNOPT solver in TOMLAB optimization toolbox within MATLAB (R2009a). The MATLAB code of iFBA was attached in Dataset S1.

**Table R1. Parameters estimated in iFBA**

| **Symbols** | **Notation** | **Unit** | **iFBA** |
| --- | --- | --- | --- |
| μ_max,L_ | Maximum specific growth rate using lactate | h^-1^ | 0.53 |
| μ_max,P_ | Maximum specific growth rate using pyruvate | h^-1^ | 0.14 |
| μ_max,A_ | Maximum specific growth rate using acetate | h^-1^ | 0.14 |
| *Y*_X/L_ | Apparent biomass yield coefficient from lactate | g DCW/mol lactate | 17.5 |
| *Y*_X/P_ | Apparent biomass yield coefficient from pyruvate | g DCW/mol pyruvate | 15.5 |
| *Y*_X/A_ | Apparent biomass yield coefficient from acetate | g DCW/mol actate | 10.9 |
| *K*_s,l_ | Monod lactate saturation constant | mM | 19.4 |
| *K*_s,p_ | Monod pyruvate saturation constant | mM | 19.4 |
| *K*_s,a_ | Monod acetate saturation constant | mM | 10.1 |
| *k*_al_ | Acetate production coefficient from lactate | L∙ (h∙g DCW)^-1^ | 0.70 |
| *k*_pl_ | Pyruvate production coefficient from lactate | L∙ (h∙g DCW)^-1^ | 0.42 |
| *k*_ap_ | Acetate production coefficient from pyruvate | L∙ (h∙g DCW)^-1^ | 0.94 |
| *k*_e_ | Endogenous metabolism rate constant | h^-1^ | 0.013 |
| *t_L_* | Lag time in growth | h | 7.10 |
| *p_1_* | Parameters used in tradeoff objective function | dimensionless | 5.3×10^-6^ |
| *p_2_* | Parameters used in tradeoff objective function | h^-1^ | 0.33 |
| *p_3_* | Parameters used in tradeoff objective function | h | 26.7 |

**Table R2. Lack-of-fit test for iFBA**

| **Model Name** | **Lack-of-fit sum of squares, SSLOF** | **Degree of freedom,**  ***df_1_*** | **Pure-error sum of squares, SSPE** | **Degree of freedom,**  ***df_1_*** | $\frac{SSLOF/df_{1}}{SSPE/df2}$ | ***F*(*df_1_*,*df_2_*)** |
| --- | --- | --- | --- | --- | --- | --- |
| iFBA | 3.726 | 56 | 0.813 | 144 | 11.78 | 1.396 |

**Note:** In order to test whether or not a model could fit the data well, we applied the lack-of-fit test. The *F*-test indicates that the iFBA model should be further improved to describe the experimental data.

**
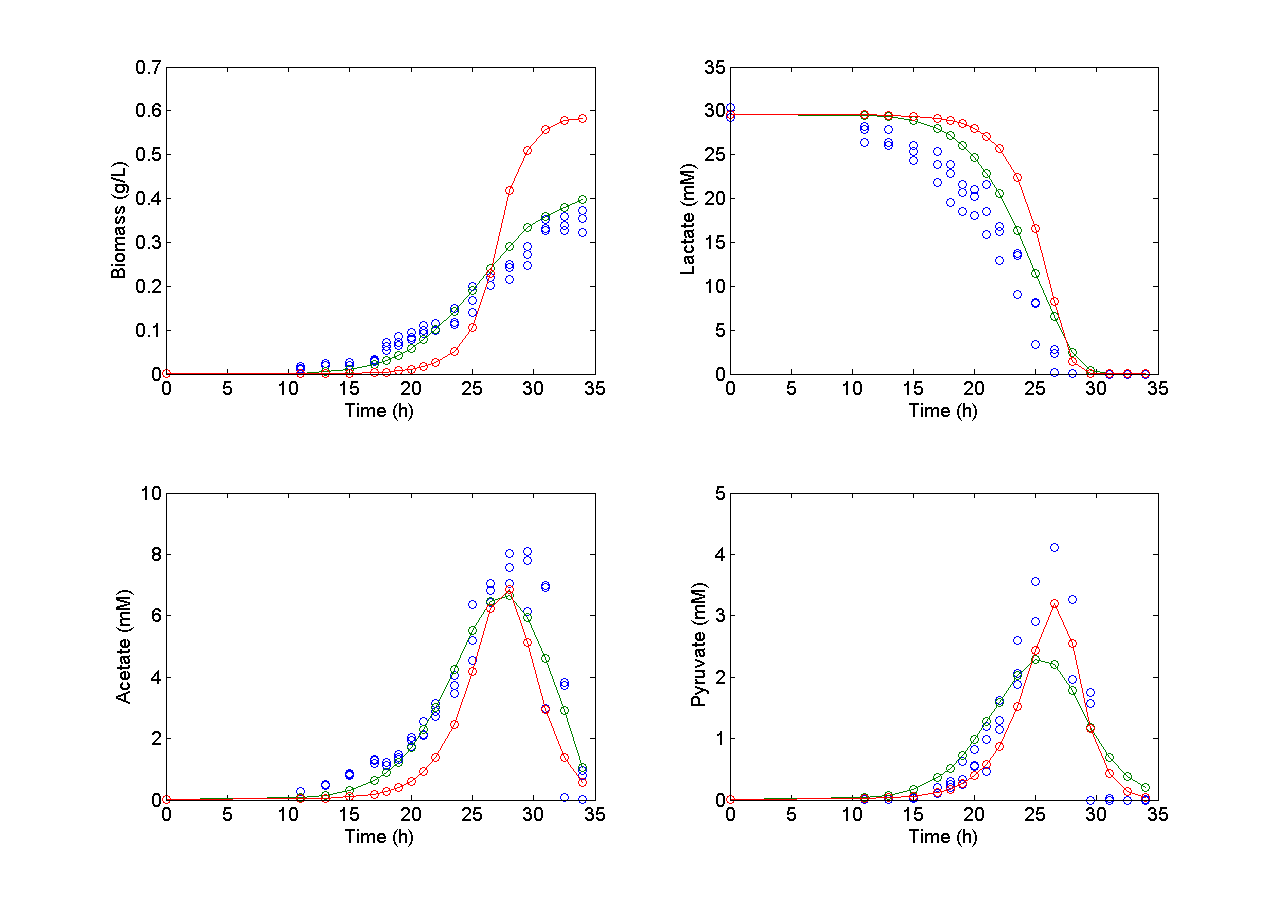
**

**Fig. R1** Growth kinetics simulated by iFBA using “maximizing growth rate” as the objective function (red line) or using the dual-objective function (green line). The blue dots are the measurements.

**
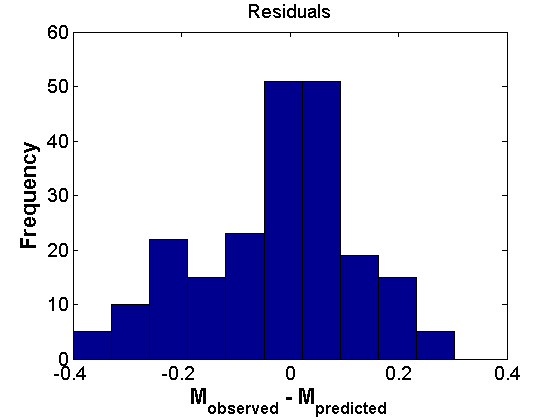
**

**Fig. R2** Histogram of normalized residuals in growth kinetics simulated by iFBA.
