# Supplementary material for: Integrating Flux Balance Analysis into Kinetic Models to Decipher the Dynamic Metabolism of Shewanella oneidensis MR-1
Source: PLoS Comput Biol. 2012 Feb 2;8(2):e1002376. doi: 10.1371/journal.pcbi.1002376 (PMC3271021; doi:10.1371/journal.pcbi.1002376)
Supplement: Text S2 — Reactions involved in 13C-labeling simulations. (DOC) [file pcbi.1002376.s007.doc]

**Text S2. Reactions involved in ^13^C-labeling simulations**

*TCA cycle and metabolites transport*

J1 (L-LACD2) LAC (abc)🡪pyr(abc)

J2 (-ACt6) accoa(ab)**↔**ACT (ab)

J3 (-PYRt2) pyr(abc)**↔**PYR(abc)

J4 (PDH) pyr(abc)🡪accoa(bc)+co2(a)

J5 (CS) oaa(abcd)+accoa(ef)🡪cit(dcbfea)

J6 (ACONT) cit(abcdef)🡪icit(abcdef)

J7 (ICDHy) icit (abcdef)🡪akg(abcde)+co2(f)

J8 (AKGD) akg(abcde)🡪succoa(bcde)+co2(a)

J9 (SUCD7) succ(1/2abcd+1/2dcba)🡪fum(1/2abcd+1/2dcba)

J10 (FUM) fum(1/2abcd+1/2dcba)🡪mal-L(abcd)

J11 (MDH) mal-L(abcd)🡪oaa(abcd)

J12 (PPC) pep(abc)+co2(d)🡪oaa(abcd)

J13 (PPCK) oaa(abcd)🡪pep(abc)+co2(d)

*Glyoxylate shunt*

J14 (ICL) icit(abcdef)🡪glx(de)+succ(1/2abcf+1/2fcba)

J15 (MALS) accoa(ab)+glx(cd)🡪mal-L(dcab)

*Reversible net fluxes and C1 metabolism*

J16 (GHMT) ser-L(abc)**↔**glx(ab)+mlthf(c)

J17 (GLYCL) glx(ab)**↔**co2(a)+mlthf(b)

J18 (ME2) mal-L(abcd)**↔**pyr(abc)+co2(d)

J19 (-SUCOAS) succoa(abcd)**↔**succ(1/2abcd+1/2dcba)

*Gluconeogenesis*

J20 (PPS) pyr(abc)🡪pep(abc)

J21 (-ENO) pep(abc)🡪2pg(abc)

J22 (-PGM) 2pg(abc)🡪3pg(abc)

*Amino acid biosynthesis*

J23 3pg(abc)🡪ser-L(abc)

J24 akg(abcde)🡪glu-L(abcde)

J25 pyr(abc)🡪ala-L(abc)

J26 oaa(abcd)🡪asp-L(abcd)

Notes:

1) All abbreviations are referred to iSO783, except LAC, PYR and ACT; which represent extracellular lactate, pyruvate and acetate, respectively.

2) The reaction IDs in iSO783 are listed with brackets. The negative sign indicates that the net flux of the pathway is in the opposite direction as set by iSO783. The amino acids biosynthesis pathways are lumped; hence no ID in iSO783 is available.
