# Supplementary material for: Integrating Flux Balance Analysis into Kinetic Models to Decipher the Dynamic Metabolism of Shewanella oneidensis MR-1
Source: PLoS Comput Biol. 2012 Feb 2;8(2):e1002376. doi: 10.1371/journal.pcbi.1002376 (PMC3271021; doi:10.1371/journal.pcbi.1002376)
Supplement: Text S3 — SBML file of the metabolic model for Shewanella oneidensis MR-1. (DOC) [file pcbi.1002376.s008.doc]

**Text S3. SBML file of the metabolic model for *Shewanella oneidensis* MR-1**

<sbml xmlns="http://www.sbml.org/sbml/level3/version1/core" level="3" version="1">

<model id="ISO">

<listOfCompartments>

<compartment id="cell" name="cell" constant="true"/>

</listOfCompartments>

<listOfSpecies>

<species id="c3hmp" name="c_3hmp" compartment="cell" hasOnlySubstanceUnits="false" boundaryCondition="false" constant="false"/>

<species id="cglygluL" name="c_gly_glu_L" compartment="cell" hasOnlySubstanceUnits="false" boundaryCondition="false" constant="false"/>

<species id="cpphn" name="c_pphn" compartment="cell" hasOnlySubstanceUnits="false" boundaryCondition="false" constant="false"/>

<species id="cthmmp" name="c_thmmp" compartment="cell" hasOnlySubstanceUnits="false" boundaryCondition="false" constant="false"/>

<species id="ctrnaasn" name="c_trnaasn" compartment="cell" hasOnlySubstanceUnits="false" boundaryCondition="false" constant="false"/>

<species id="ctrnaasp" name="c_trnaasp" compartment="cell" hasOnlySubstanceUnits="false" boundaryCondition="false" constant="false"/>

<species id="cdmpp" name="c_dmpp" compartment="cell" hasOnlySubstanceUnits="false" boundaryCondition="false" constant="false"/>

<species id="ciletrna" name="c_iletrna" compartment="cell" hasOnlySubstanceUnits="false" boundaryCondition="false" constant="false"/>

<species id="cdin" name="c_din" compartment="cell" hasOnlySubstanceUnits="false" boundaryCondition="false" constant="false"/>

<species id="ctrnaile" name="c_trnaile" compartment="cell" hasOnlySubstanceUnits="false" boundaryCondition="false" constant="false"/>

<species id="cagm" name="c_agm" compartment="cell" hasOnlySubstanceUnits="false" boundaryCondition="false" constant="false"/>

<species id="c2oph" name="c_2oph" compartment="cell" hasOnlySubstanceUnits="false" boundaryCondition="false" constant="false"/>

<species id="cpsd5p" name="c_psd5p" compartment="cell" hasOnlySubstanceUnits="false" boundaryCondition="false" constant="false"/>

<species id="ckdo8nlipid4" name="c_kdo8nlipid4" compartment="cell" hasOnlySubstanceUnits="false" boundaryCondition="false" constant="false"/>

<species id="cfdxo42" name="c_fdxo_4_2" compartment="cell" hasOnlySubstanceUnits="false" boundaryCondition="false" constant="false"/>

<species id="cgp4g" name="c_gp4g" compartment="cell" hasOnlySubstanceUnits="false" boundaryCondition="false" constant="false"/>

<species id="cgam6p" name="c_gam6p" compartment="cell" hasOnlySubstanceUnits="false" boundaryCondition="false" constant="false"/>

<species id="cglyclt" name="c_glyclt" compartment="cell" hasOnlySubstanceUnits="false" boundaryCondition="false" constant="false"/>

<species id="cdudp" name="c_dudp" compartment="cell" hasOnlySubstanceUnits="false" boundaryCondition="false" constant="false"/>

<species id="cfmn" name="c_fmn" compartment="cell" hasOnlySubstanceUnits="false" boundaryCondition="false" constant="false"/>

<species id="ctrnaleu" name="c_trnaleu" compartment="cell" hasOnlySubstanceUnits="false" boundaryCondition="false" constant="false"/>

<species id="c5678thh" name="c_5678thh" compartment="cell" hasOnlySubstanceUnits="false" boundaryCondition="false" constant="false"/>

<species id="cmthgxl" name="c_mthgxl" compartment="cell" hasOnlySubstanceUnits="false" boundaryCondition="false" constant="false"/>

<species id="cprlp" name="c_prlp" compartment="cell" hasOnlySubstanceUnits="false" boundaryCondition="false" constant="false"/>

<species id="ccamp" name="c_camp" compartment="cell" hasOnlySubstanceUnits="false" boundaryCondition="false" constant="false"/>

<species id="c25dhpp" name="c_25dhpp" compartment="cell" hasOnlySubstanceUnits="false" boundaryCondition="false" constant="false"/>

<species id="cins" name="c_ins" compartment="cell" hasOnlySubstanceUnits="false" boundaryCondition="false" constant="false"/>

<species id="chisL" name="c_his_L" compartment="cell" hasOnlySubstanceUnits="false" boundaryCondition="false" constant="false"/>

<species id="cthf" name="c_thf" compartment="cell" hasOnlySubstanceUnits="false" boundaryCondition="false" constant="false"/>

<species id="cahcys" name="c_ahcys" compartment="cell" hasOnlySubstanceUnits="false" boundaryCondition="false" constant="false"/>

<species id="cggdp" name="c_ggdp" compartment="cell" hasOnlySubstanceUnits="false" boundaryCondition="false" constant="false"/>

<species id="c2mcit" name="c_2mcit" compartment="cell" hasOnlySubstanceUnits="false" boundaryCondition="false" constant="false"/>

<species id="cpap" name="c_pap" compartment="cell" hasOnlySubstanceUnits="false" boundaryCondition="false" constant="false"/>

<species id="cargtrna" name="c_argtrna" compartment="cell" hasOnlySubstanceUnits="false" boundaryCondition="false" constant="false"/>

<species id="cbtn" name="c_btn" compartment="cell" hasOnlySubstanceUnits="false" boundaryCondition="false" constant="false"/>

<species id="ctrnatyr" name="c_trnatyr" compartment="cell" hasOnlySubstanceUnits="false" boundaryCondition="false" constant="false"/>

<species id="cdump" name="c_dump" compartment="cell" hasOnlySubstanceUnits="false" boundaryCondition="false" constant="false"/>

<species id="caspL" name="c_asp_L" compartment="cell" hasOnlySubstanceUnits="false" boundaryCondition="false" constant="false"/>

<species id="c4izp" name="c_4izp" compartment="cell" hasOnlySubstanceUnits="false" boundaryCondition="false" constant="false"/>

<species id="cssaltpp" name="c_ssaltpp" compartment="cell" hasOnlySubstanceUnits="false" boundaryCondition="false" constant="false"/>

<species id="cgua" name="c_gua" compartment="cell" hasOnlySubstanceUnits="false" boundaryCondition="false" constant="false"/>

<species id="cthymd" name="c_thymd" compartment="cell" hasOnlySubstanceUnits="false" boundaryCondition="false" constant="false"/>

<species id="cump" name="c_ump" compartment="cell" hasOnlySubstanceUnits="false" boundaryCondition="false" constant="false"/>

<species id="ccysL" name="c_cys_L" compartment="cell" hasOnlySubstanceUnits="false" boundaryCondition="false" constant="false"/>

<species id="cglu5p" name="c_glu5p" compartment="cell" hasOnlySubstanceUnits="false" boundaryCondition="false" constant="false"/>

<species id="ctrnaval" name="c_trnaval" compartment="cell" hasOnlySubstanceUnits="false" boundaryCondition="false" constant="false"/>

<species id="clipidA" name="c_lipidA" compartment="cell" hasOnlySubstanceUnits="false" boundaryCondition="false" constant="false"/>

<species id="ctrnaala" name="c_trnaala" compartment="cell" hasOnlySubstanceUnits="false" boundaryCondition="false" constant="false"/>

<species id="chmglutS" name="c_hmglut_S" compartment="cell" hasOnlySubstanceUnits="false" boundaryCondition="false" constant="false"/>

<species id="cudpgalur" name="c_udpgalur" compartment="cell" hasOnlySubstanceUnits="false" boundaryCondition="false" constant="false"/>

<species id="cgmh7p" name="c_gmh7p" compartment="cell" hasOnlySubstanceUnits="false" boundaryCondition="false" constant="false"/>

<species id="ccmac" name="c_cmac" compartment="cell" hasOnlySubstanceUnits="false" boundaryCondition="false" constant="false"/>

<species id="c4hdxbutn" name="c_4hdxbutn" compartment="cell" hasOnlySubstanceUnits="false" boundaryCondition="false" constant="false"/>

<species id="csucsal" name="c_sucsal" compartment="cell" hasOnlySubstanceUnits="false" boundaryCondition="false" constant="false"/>

<species id="cuagmda" name="c_uagmda" compartment="cell" hasOnlySubstanceUnits="false" boundaryCondition="false" constant="false"/>

<species id="cacACP" name="c_acACP" compartment="cell" hasOnlySubstanceUnits="false" boundaryCondition="false" constant="false"/>

<species id="c3dhsk" name="c_3dhsk" compartment="cell" hasOnlySubstanceUnits="false" boundaryCondition="false" constant="false"/>

<species id="capoACP" name="c_apoACP" compartment="cell" hasOnlySubstanceUnits="false" boundaryCondition="false" constant="false"/>

<species id="cocdcea" name="c_ocdcea" compartment="cell" hasOnlySubstanceUnits="false" boundaryCondition="false" constant="false"/>

<species id="cuamr" name="c_uamr" compartment="cell" hasOnlySubstanceUnits="false" boundaryCondition="false" constant="false"/>

<species id="csbzcoa" name="c_sbzcoa" compartment="cell" hasOnlySubstanceUnits="false" boundaryCondition="false" constant="false"/>

<species id="crb15bp" name="c_rb15bp" compartment="cell" hasOnlySubstanceUnits="false" boundaryCondition="false" constant="false"/>

<species id="chistd" name="c_histd" compartment="cell" hasOnlySubstanceUnits="false" boundaryCondition="false" constant="false"/>

<species id="ch2mb4p" name="c_h2mb4p" compartment="cell" hasOnlySubstanceUnits="false" boundaryCondition="false" constant="false"/>

<species id="cdcmp" name="c_dcmp" compartment="cell" hasOnlySubstanceUnits="false" boundaryCondition="false" constant="false"/>

<species id="c2ohph" name="c_2ohph" compartment="cell" hasOnlySubstanceUnits="false" boundaryCondition="false" constant="false"/>

<species id="ctrnamet" name="c_trnamet" compartment="cell" hasOnlySubstanceUnits="false" boundaryCondition="false" constant="false"/>

<species id="cdhpmp" name="c_dhpmp" compartment="cell" hasOnlySubstanceUnits="false" boundaryCondition="false" constant="false"/>

<species id="cair" name="c_air" compartment="cell" hasOnlySubstanceUnits="false" boundaryCondition="false" constant="false"/>

<species id="cmql7" name="c_mql7" compartment="cell" hasOnlySubstanceUnits="false" boundaryCondition="false" constant="false"/>

<species id="cppbng" name="c_ppbng" compartment="cell" hasOnlySubstanceUnits="false" boundaryCondition="false" constant="false"/>

<species id="cglycogen" name="c_glycogen" compartment="cell" hasOnlySubstanceUnits="false" boundaryCondition="false" constant="false"/>

<species id="cdgmp" name="c_dgmp" compartment="cell" hasOnlySubstanceUnits="false" boundaryCondition="false" constant="false"/>

<species id="cacmam" name="c_acmam" compartment="cell" hasOnlySubstanceUnits="false" boundaryCondition="false" constant="false"/>

<species id="c4fumacac" name="c_4fumacac" compartment="cell" hasOnlySubstanceUnits="false" boundaryCondition="false" constant="false"/>

<species id="c4hbutcoa" name="c_4hbutcoa" compartment="cell" hasOnlySubstanceUnits="false" boundaryCondition="false" constant="false"/>

<species id="cpaps" name="c_paps" compartment="cell" hasOnlySubstanceUnits="false" boundaryCondition="false" constant="false"/>

<species id="csertrna" name="c_sertrna" compartment="cell" hasOnlySubstanceUnits="false" boundaryCondition="false" constant="false"/>

<species id="cacgam6p" name="c_acgam6p" compartment="cell" hasOnlySubstanceUnits="false" boundaryCondition="false" constant="false"/>

<species id="c2pglyc" name="c_2pglyc" compartment="cell" hasOnlySubstanceUnits="false" boundaryCondition="false" constant="false"/>

<species id="cthym" name="c_thym" compartment="cell" hasOnlySubstanceUnits="false" boundaryCondition="false" constant="false"/>

<species id="cetha" name="c_etha" compartment="cell" hasOnlySubstanceUnits="false" boundaryCondition="false" constant="false"/>

<species id="ctrnagln" name="c_trnagln" compartment="cell" hasOnlySubstanceUnits="false" boundaryCondition="false" constant="false"/>

<species id="cdmlz" name="c_dmlz" compartment="cell" hasOnlySubstanceUnits="false" boundaryCondition="false" constant="false"/>

<species id="co2" name="c_o2" compartment="cell" hasOnlySubstanceUnits="false" boundaryCondition="false" constant="false"/>

<species id="ctrnaglu" name="c_trnaglu" compartment="cell" hasOnlySubstanceUnits="false" boundaryCondition="false" constant="false"/>

<species id="cforglu" name="c_forglu" compartment="cell" hasOnlySubstanceUnits="false" boundaryCondition="false" constant="false"/>

<species id="ctrnagly" name="c_trnagly" compartment="cell" hasOnlySubstanceUnits="false" boundaryCondition="false" constant="false"/>

<species id="cf6p" name="c_f6p" compartment="cell" hasOnlySubstanceUnits="false" boundaryCondition="false" constant="false"/>

<species id="csprm" name="c_sprm" compartment="cell" hasOnlySubstanceUnits="false" boundaryCondition="false" constant="false"/>

<species id="cmicit" name="c_micit" compartment="cell" hasOnlySubstanceUnits="false" boundaryCondition="false" constant="false"/>

<species id="c6pgl" name="c_6pgl" compartment="cell" hasOnlySubstanceUnits="false" boundaryCondition="false" constant="false"/>

<species id="cfa3ACP" name="c_fa3ACP" compartment="cell" hasOnlySubstanceUnits="false" boundaryCondition="false" constant="false"/>

<species id="c12dag3p" name="c_12dag3p" compartment="cell" hasOnlySubstanceUnits="false" boundaryCondition="false" constant="false"/>

<species id="cfa13ACP" name="c_fa13ACP" compartment="cell" hasOnlySubstanceUnits="false" boundaryCondition="false" constant="false"/>

<species id="cfrmd" name="c_frmd" compartment="cell" hasOnlySubstanceUnits="false" boundaryCondition="false" constant="false"/>

<species id="c6pgc" name="c_6pgc" compartment="cell" hasOnlySubstanceUnits="false" boundaryCondition="false" constant="false"/>

<species id="cacald" name="c_acald" compartment="cell" hasOnlySubstanceUnits="false" boundaryCondition="false" constant="false"/>

<species id="coaa" name="c_oaa" compartment="cell" hasOnlySubstanceUnits="false" boundaryCondition="false" constant="false"/>

<species id="cpppg9" name="c_pppg9" compartment="cell" hasOnlySubstanceUnits="false" boundaryCondition="false" constant="false"/>

<species id="cnicrnt" name="c_nicrnt" compartment="cell" hasOnlySubstanceUnits="false" boundaryCondition="false" constant="false"/>

<species id="chepdp" name="c_hepdp" compartment="cell" hasOnlySubstanceUnits="false" boundaryCondition="false" constant="false"/>

<species id="ch" name="c_h" compartment="cell" hasOnlySubstanceUnits="false" boundaryCondition="false" constant="false"/>

<species id="c12dgr" name="c_12dgr" compartment="cell" hasOnlySubstanceUnits="false" boundaryCondition="false" constant="false"/>

<species id="cg1p" name="c_g1p" compartment="cell" hasOnlySubstanceUnits="false" boundaryCondition="false" constant="false"/>

<species id="cphthr" name="c_phthr" compartment="cell" hasOnlySubstanceUnits="false" boundaryCondition="false" constant="false"/>

<species id="cudp" name="c_udp" compartment="cell" hasOnlySubstanceUnits="false" boundaryCondition="false" constant="false"/>

<species id="cman6p" name="c_man6p" compartment="cell" hasOnlySubstanceUnits="false" boundaryCondition="false" constant="false"/>

<species id="caps" name="c_aps" compartment="cell" hasOnlySubstanceUnits="false" boundaryCondition="false" constant="false"/>

<species id="cglyc3p" name="c_glyc3p" compartment="cell" hasOnlySubstanceUnits="false" boundaryCondition="false" constant="false"/>

<species id="cptdca" name="c_ptdca" compartment="cell" hasOnlySubstanceUnits="false" boundaryCondition="false" constant="false"/>

<species id="cfrdp" name="c_frdp" compartment="cell" hasOnlySubstanceUnits="false" boundaryCondition="false" constant="false"/>

<species id="c3ig3p" name="c_3ig3p" compartment="cell" hasOnlySubstanceUnits="false" boundaryCondition="false" constant="false"/>

<species id="cglycR" name="c_glyc_R" compartment="cell" hasOnlySubstanceUnits="false" boundaryCondition="false" constant="false"/>

<species id="cthrLA" name="c_thr_LA" compartment="cell" hasOnlySubstanceUnits="false" boundaryCondition="false" constant="false"/>

<species id="ctrnacys" name="c_trnacys" compartment="cell" hasOnlySubstanceUnits="false" boundaryCondition="false" constant="false"/>

<species id="c13dpg" name="c_13dpg" compartment="cell" hasOnlySubstanceUnits="false" boundaryCondition="false" constant="false"/>

<species id="ctrnahis" name="c_trnahis" compartment="cell" hasOnlySubstanceUnits="false" boundaryCondition="false" constant="false"/>

<species id="cuaagmda" name="c_uaagmda" compartment="cell" hasOnlySubstanceUnits="false" boundaryCondition="false" constant="false"/>

<species id="cugmd" name="c_ugmd" compartment="cell" hasOnlySubstanceUnits="false" boundaryCondition="false" constant="false"/>

<species id="c2c25dho" name="c_2c25dho" compartment="cell" hasOnlySubstanceUnits="false" boundaryCondition="false" constant="false"/>

<species id="chdcea" name="c_hdcea" compartment="cell" hasOnlySubstanceUnits="false" boundaryCondition="false" constant="false"/>

<species id="clipidAds" name="c_lipidAds" compartment="cell" hasOnlySubstanceUnits="false" boundaryCondition="false" constant="false"/>

<species id="ccdpdag" name="c_cdpdag" compartment="cell" hasOnlySubstanceUnits="false" boundaryCondition="false" constant="false"/>

<species id="c2cpr5p" name="c_2cpr5p" compartment="cell" hasOnlySubstanceUnits="false" boundaryCondition="false" constant="false"/>

<species id="cpendp" name="c_pendp" compartment="cell" hasOnlySubstanceUnits="false" boundaryCondition="false" constant="false"/>

<species id="casnL" name="c_asn_L" compartment="cell" hasOnlySubstanceUnits="false" boundaryCondition="false" constant="false"/>

<species id="c4ppcys" name="c_4ppcys" compartment="cell" hasOnlySubstanceUnits="false" boundaryCondition="false" constant="false"/>

<species id="cvaltrna" name="c_valtrna" compartment="cell" hasOnlySubstanceUnits="false" boundaryCondition="false" constant="false"/>

<species id="cmethf" name="c_methf" compartment="cell" hasOnlySubstanceUnits="false" boundaryCondition="false" constant="false"/>

<species id="c3pg" name="c_3pg" compartment="cell" hasOnlySubstanceUnits="false" boundaryCondition="false" constant="false"/>

<species id="cdmbzid" name="c_dmbzid" compartment="cell" hasOnlySubstanceUnits="false" boundaryCondition="false" constant="false"/>

<species id="cman1p" name="c_man1p" compartment="cell" hasOnlySubstanceUnits="false" boundaryCondition="false" constant="false"/>

<species id="cicit" name="c_icit" compartment="cell" hasOnlySubstanceUnits="false" boundaryCondition="false" constant="false"/>

<species id="ctrnaphe" name="c_trnaphe" compartment="cell" hasOnlySubstanceUnits="false" boundaryCondition="false" constant="false"/>

<species id="c4ppan" name="c_4ppan" compartment="cell" hasOnlySubstanceUnits="false" boundaryCondition="false" constant="false"/>

<species id="cpheme" name="c_pheme" compartment="cell" hasOnlySubstanceUnits="false" boundaryCondition="false" constant="false"/>

<species id="ctrnapro" name="c_trnapro" compartment="cell" hasOnlySubstanceUnits="false" boundaryCondition="false" constant="false"/>

<species id="camp" name="c_amp" compartment="cell" hasOnlySubstanceUnits="false" boundaryCondition="false" constant="false"/>

<species id="cleutrna" name="c_leutrna" compartment="cell" hasOnlySubstanceUnits="false" boundaryCondition="false" constant="false"/>

<species id="camet" name="c_amet" compartment="cell" hasOnlySubstanceUnits="false" boundaryCondition="false" constant="false"/>

<species id="cpntoR" name="c_pnto_R" compartment="cell" hasOnlySubstanceUnits="false" boundaryCondition="false" constant="false"/>

<species id="cconfald" name="c_confald" compartment="cell" hasOnlySubstanceUnits="false" boundaryCondition="false" constant="false"/>

<species id="cr1p" name="c_r1p" compartment="cell" hasOnlySubstanceUnits="false" boundaryCondition="false" constant="false"/>

<species id="cachms" name="c_achms" compartment="cell" hasOnlySubstanceUnits="false" boundaryCondition="false" constant="false"/>

<species id="cduri" name="c_duri" compartment="cell" hasOnlySubstanceUnits="false" boundaryCondition="false" constant="false"/>

<species id="cppi" name="c_ppi" compartment="cell" hasOnlySubstanceUnits="false" boundaryCondition="false" constant="false"/>

<species id="cdnaSon" name="c_dna_Son" compartment="cell" hasOnlySubstanceUnits="false" boundaryCondition="false" constant="false"/>

<species id="c2h3opp" name="c_2h3opp" compartment="cell" hasOnlySubstanceUnits="false" boundaryCondition="false" constant="false"/>

<species id="cchitob" name="c_chitob" compartment="cell" hasOnlySubstanceUnits="false" boundaryCondition="false" constant="false"/>

<species id="cfe2" name="c_fe2" compartment="cell" hasOnlySubstanceUnits="false" boundaryCondition="false" constant="false"/>

<species id="cargsuc" name="c_argsuc" compartment="cell" hasOnlySubstanceUnits="false" boundaryCondition="false" constant="false"/>

<species id="cppp9" name="c_ppp9" compartment="cell" hasOnlySubstanceUnits="false" boundaryCondition="false" constant="false"/>

<species id="ckdo8p" name="c_kdo8p" compartment="cell" hasOnlySubstanceUnits="false" boundaryCondition="false" constant="false"/>

<species id="cseln" name="c_seln" compartment="cell" hasOnlySubstanceUnits="false" boundaryCondition="false" constant="false"/>

<species id="cfald" name="c_fald" compartment="cell" hasOnlySubstanceUnits="false" boundaryCondition="false" constant="false"/>

<species id="co2" name="c_o2_" compartment="cell" hasOnlySubstanceUnits="false" boundaryCondition="false" constant="false"/>

<species id="cpheL" name="c_phe_L" compartment="cell" hasOnlySubstanceUnits="false" boundaryCondition="false" constant="false"/>

<species id="ch2o" name="c_h2o" compartment="cell" hasOnlySubstanceUnits="false" boundaryCondition="false" constant="false"/>

<species id="csl2a6o" name="c_sl2a6o" compartment="cell" hasOnlySubstanceUnits="false" boundaryCondition="false" constant="false"/>

<species id="ch2s" name="c_h2s" compartment="cell" hasOnlySubstanceUnits="false" boundaryCondition="false" constant="false"/>

<species id="cipdp" name="c_ipdp" compartment="cell" hasOnlySubstanceUnits="false" boundaryCondition="false" constant="false"/>

<species id="catp" name="c_atp" compartment="cell" hasOnlySubstanceUnits="false" boundaryCondition="false" constant="false"/>

<species id="cacglu" name="c_acglu" compartment="cell" hasOnlySubstanceUnits="false" boundaryCondition="false" constant="false"/>

<species id="cpppi" name="c_pppi" compartment="cell" hasOnlySubstanceUnits="false" boundaryCondition="false" constant="false"/>

<species id="cgrdp" name="c_grdp" compartment="cell" hasOnlySubstanceUnits="false" boundaryCondition="false" constant="false"/>

<species id="c3c4mop" name="c_3c4mop" compartment="cell" hasOnlySubstanceUnits="false" boundaryCondition="false" constant="false"/>

<species id="caspsa" name="c_aspsa" compartment="cell" hasOnlySubstanceUnits="false" boundaryCondition="false" constant="false"/>

<species id="cthbpt" name="c_thbpt" compartment="cell" hasOnlySubstanceUnits="false" boundaryCondition="false" constant="false"/>

<species id="cglutrna" name="c_glutrna" compartment="cell" hasOnlySubstanceUnits="false" boundaryCondition="false" constant="false"/>

<species id="cdxyl5p" name="c_dxyl5p" compartment="cell" hasOnlySubstanceUnits="false" boundaryCondition="false" constant="false"/>

<species id="csucgsa" name="c_sucgsa" compartment="cell" hasOnlySubstanceUnits="false" boundaryCondition="false" constant="false"/>

<species id="c2dr1p" name="c_2dr1p" compartment="cell" hasOnlySubstanceUnits="false" boundaryCondition="false" constant="false"/>

<species id="calatrna" name="c_alatrna" compartment="cell" hasOnlySubstanceUnits="false" boundaryCondition="false" constant="false"/>

<species id="cchor" name="c_chor" compartment="cell" hasOnlySubstanceUnits="false" boundaryCondition="false" constant="false"/>

<species id="ctcynt" name="c_tcynt" compartment="cell" hasOnlySubstanceUnits="false" boundaryCondition="false" constant="false"/>

<species id="clipidX" name="c_lipidX" compartment="cell" hasOnlySubstanceUnits="false" boundaryCondition="false" constant="false"/>

<species id="c2ahbut" name="c_2ahbut" compartment="cell" hasOnlySubstanceUnits="false" boundaryCondition="false" constant="false"/>

<species id="cprbamp" name="c_prbamp" compartment="cell" hasOnlySubstanceUnits="false" boundaryCondition="false" constant="false"/>

<species id="ccdp" name="c_cdp" compartment="cell" hasOnlySubstanceUnits="false" boundaryCondition="false" constant="false"/>

<species id="cura" name="c_ura" compartment="cell" hasOnlySubstanceUnits="false" boundaryCondition="false" constant="false"/>

<species id="cfa14ACP" name="c_fa14ACP" compartment="cell" hasOnlySubstanceUnits="false" boundaryCondition="false" constant="false"/>

<species id="curi" name="c_uri" compartment="cell" hasOnlySubstanceUnits="false" boundaryCondition="false" constant="false"/>

<species id="cxu5pD" name="c_xu5p_D" compartment="cell" hasOnlySubstanceUnits="false" boundaryCondition="false" constant="false"/>

<species id="c4abut" name="c_4abut" compartment="cell" hasOnlySubstanceUnits="false" boundaryCondition="false" constant="false"/>

<species id="csl26da" name="c_sl26da" compartment="cell" hasOnlySubstanceUnits="false" boundaryCondition="false" constant="false"/>

<species id="calacS" name="c_alac_S" compartment="cell" hasOnlySubstanceUnits="false" boundaryCondition="false" constant="false"/>

<species id="cichor" name="c_ichor" compartment="cell" hasOnlySubstanceUnits="false" boundaryCondition="false" constant="false"/>

<species id="carsna" name="c_arsna" compartment="cell" hasOnlySubstanceUnits="false" boundaryCondition="false" constant="false"/>

<species id="cametam" name="c_ametam" compartment="cell" hasOnlySubstanceUnits="false" boundaryCondition="false" constant="false"/>

<species id="c2omhmbl" name="c_2omhmbl" compartment="cell" hasOnlySubstanceUnits="false" boundaryCondition="false" constant="false"/>

<species id="cdhna" name="c_dhna" compartment="cell" hasOnlySubstanceUnits="false" boundaryCondition="false" constant="false"/>

<species id="ctrdrd" name="c_trdrd" compartment="cell" hasOnlySubstanceUnits="false" boundaryCondition="false" constant="false"/>

<species id="cu3hga" name="c_u3hga" compartment="cell" hasOnlySubstanceUnits="false" boundaryCondition="false" constant="false"/>

<species id="cohpb" name="c_ohpb" compartment="cell" hasOnlySubstanceUnits="false" boundaryCondition="false" constant="false"/>

<species id="csucc" name="c_succ" compartment="cell" hasOnlySubstanceUnits="false" boundaryCondition="false" constant="false"/>

<species id="c2ddg6p" name="c_2ddg6p" compartment="cell" hasOnlySubstanceUnits="false" boundaryCondition="false" constant="false"/>

<species id="ccmp" name="c_cmp" compartment="cell" hasOnlySubstanceUnits="false" boundaryCondition="false" constant="false"/>

<species id="cactACP" name="c_actACP" compartment="cell" hasOnlySubstanceUnits="false" boundaryCondition="false" constant="false"/>

<species id="c23dhdp" name="c_23dhdp" compartment="cell" hasOnlySubstanceUnits="false" boundaryCondition="false" constant="false"/>

<species id="cuppg3" name="c_uppg3" compartment="cell" hasOnlySubstanceUnits="false" boundaryCondition="false" constant="false"/>

<species id="cudpg" name="c_udpg" compartment="cell" hasOnlySubstanceUnits="false" boundaryCondition="false" constant="false"/>

<species id="cmmql7" name="c_mmql7" compartment="cell" hasOnlySubstanceUnits="false" boundaryCondition="false" constant="false"/>

<species id="cspmd" name="c_spmd" compartment="cell" hasOnlySubstanceUnits="false" boundaryCondition="false" constant="false"/>

<species id="cgdpdp" name="c_gdpdp" compartment="cell" hasOnlySubstanceUnits="false" boundaryCondition="false" constant="false"/>

<species id="cgmp" name="c_gmp" compartment="cell" hasOnlySubstanceUnits="false" boundaryCondition="false" constant="false"/>

<species id="cibcoa" name="c_ibcoa" compartment="cell" hasOnlySubstanceUnits="false" boundaryCondition="false" constant="false"/>

<species id="cuacgam" name="c_uacgam" compartment="cell" hasOnlySubstanceUnits="false" boundaryCondition="false" constant="false"/>

<species id="ccit" name="c_cit" compartment="cell" hasOnlySubstanceUnits="false" boundaryCondition="false" constant="false"/>

<species id="cfmnRD" name="c_fmnRD" compartment="cell" hasOnlySubstanceUnits="false" boundaryCondition="false" constant="false"/>

<species id="cmalL" name="c_mal_L" compartment="cell" hasOnlySubstanceUnits="false" boundaryCondition="false" constant="false"/>

<species id="clgtS" name="c_lgt_S" compartment="cell" hasOnlySubstanceUnits="false" boundaryCondition="false" constant="false"/>

<species id="cac" name="c_ac" compartment="cell" hasOnlySubstanceUnits="false" boundaryCondition="false" constant="false"/>

<species id="cslcys" name="c_slcys" compartment="cell" hasOnlySubstanceUnits="false" boundaryCondition="false" constant="false"/>

<species id="c4hbz" name="c_4hbz" compartment="cell" hasOnlySubstanceUnits="false" boundaryCondition="false" constant="false"/>

<species id="cr5p" name="c_r5p" compartment="cell" hasOnlySubstanceUnits="false" boundaryCondition="false" constant="false"/>

<species id="cgam1p" name="c_gam1p" compartment="cell" hasOnlySubstanceUnits="false" boundaryCondition="false" constant="false"/>

<species id="cglyc" name="c_glyc" compartment="cell" hasOnlySubstanceUnits="false" boundaryCondition="false" constant="false"/>

<species id="chmgcoa" name="c_hmgcoa" compartment="cell" hasOnlySubstanceUnits="false" boundaryCondition="false" constant="false"/>

<species id="clpsSO" name="c_lps_SO" compartment="cell" hasOnlySubstanceUnits="false" boundaryCondition="false" constant="false"/>

<species id="cunknown1" name="c_unknown1" compartment="cell" hasOnlySubstanceUnits="false" boundaryCondition="false" constant="false"/>

<species id="cdcyt" name="c_dcyt" compartment="cell" hasOnlySubstanceUnits="false" boundaryCondition="false" constant="false"/>

<species id="c4mop" name="c_4mop" compartment="cell" hasOnlySubstanceUnits="false" boundaryCondition="false" constant="false"/>

<species id="cmetL" name="c_met_L" compartment="cell" hasOnlySubstanceUnits="false" boundaryCondition="false" constant="false"/>

<species id="cribD" name="c_rib_D" compartment="cell" hasOnlySubstanceUnits="false" boundaryCondition="false" constant="false"/>

<species id="cargL" name="c_arg_L" compartment="cell" hasOnlySubstanceUnits="false" boundaryCondition="false" constant="false"/>

<species id="cudcpdp" name="c_udcpdp" compartment="cell" hasOnlySubstanceUnits="false" boundaryCondition="false" constant="false"/>

<species id="cfad" name="c_fad" compartment="cell" hasOnlySubstanceUnits="false" boundaryCondition="false" constant="false"/>

<species id="cadcobhex" name="c_adcobhex" compartment="cell" hasOnlySubstanceUnits="false" boundaryCondition="false" constant="false"/>

<species id="c3mop" name="c_3mop" compartment="cell" hasOnlySubstanceUnits="false" boundaryCondition="false" constant="false"/>

<species id="cprotrna" name="c_protrna" compartment="cell" hasOnlySubstanceUnits="false" boundaryCondition="false" constant="false"/>

<species id="ccytd" name="c_cytd" compartment="cell" hasOnlySubstanceUnits="false" boundaryCondition="false" constant="false"/>

<species id="ccbi" name="c_cbi" compartment="cell" hasOnlySubstanceUnits="false" boundaryCondition="false" constant="false"/>

<species id="chdeACP" name="c_hdeACP" compartment="cell" hasOnlySubstanceUnits="false" boundaryCondition="false" constant="false"/>

<species id="c4hba" name="c_4hba" compartment="cell" hasOnlySubstanceUnits="false" boundaryCondition="false" constant="false"/>

<species id="csucorn" name="c_sucorn" compartment="cell" hasOnlySubstanceUnits="false" boundaryCondition="false" constant="false"/>

<species id="c26dapLL" name="c_26dap_LL" compartment="cell" hasOnlySubstanceUnits="false" boundaryCondition="false" constant="false"/>

<species id="cdtdp" name="c_dtdp" compartment="cell" hasOnlySubstanceUnits="false" boundaryCondition="false" constant="false"/>

<species id="c3mob" name="c_3mob" compartment="cell" hasOnlySubstanceUnits="false" boundaryCondition="false" constant="false"/>

<species id="cmalACP" name="c_malACP" compartment="cell" hasOnlySubstanceUnits="false" boundaryCondition="false" constant="false"/>

<species id="cgtp" name="c_gtp" compartment="cell" hasOnlySubstanceUnits="false" boundaryCondition="false" constant="false"/>

<species id="c4mlacac" name="c_4mlacac" compartment="cell" hasOnlySubstanceUnits="false" boundaryCondition="false" constant="false"/>

<species id="cagpe" name="c_agpe" compartment="cell" hasOnlySubstanceUnits="false" boundaryCondition="false" constant="false"/>

<species id="cagpg" name="c_agpg" compartment="cell" hasOnlySubstanceUnits="false" boundaryCondition="false" constant="false"/>

<species id="cdtdp6dm" name="c_dtdp6dm" compartment="cell" hasOnlySubstanceUnits="false" boundaryCondition="false" constant="false"/>

<species id="csuccoa" name="c_succoa" compartment="cell" hasOnlySubstanceUnits="false" boundaryCondition="false" constant="false"/>

<species id="cphetrna" name="c_phetrna" compartment="cell" hasOnlySubstanceUnits="false" boundaryCondition="false" constant="false"/>

<species id="caacoa" name="c_aacoa" compartment="cell" hasOnlySubstanceUnits="false" boundaryCondition="false" constant="false"/>

<species id="ccbasp" name="c_cbasp" compartment="cell" hasOnlySubstanceUnits="false" boundaryCondition="false" constant="false"/>

<species id="c2dr5p" name="c_2dr5p" compartment="cell" hasOnlySubstanceUnits="false" boundaryCondition="false" constant="false"/>

<species id="cdgsn" name="c_dgsn" compartment="cell" hasOnlySubstanceUnits="false" boundaryCondition="false" constant="false"/>

<species id="curcan" name="c_urcan" compartment="cell" hasOnlySubstanceUnits="false" boundaryCondition="false" constant="false"/>

<species id="cfa3" name="c_fa3" compartment="cell" hasOnlySubstanceUnits="false" boundaryCondition="false" constant="false"/>

<species id="cfa1" name="c_fa1" compartment="cell" hasOnlySubstanceUnits="false" boundaryCondition="false" constant="false"/>

<species id="cfa6" name="c_fa6" compartment="cell" hasOnlySubstanceUnits="false" boundaryCondition="false" constant="false"/>

<species id="cdcdp" name="c_dcdp" compartment="cell" hasOnlySubstanceUnits="false" boundaryCondition="false" constant="false"/>

<species id="cara5p" name="c_ara5p" compartment="cell" hasOnlySubstanceUnits="false" boundaryCondition="false" constant="false"/>

<species id="cacgam" name="c_acgam" compartment="cell" hasOnlySubstanceUnits="false" boundaryCondition="false" constant="false"/>

<species id="cddcaACP" name="c_ddcaACP" compartment="cell" hasOnlySubstanceUnits="false" boundaryCondition="false" constant="false"/>

<species id="cgly" name="c_gly" compartment="cell" hasOnlySubstanceUnits="false" boundaryCondition="false" constant="false"/>

<species id="cileL" name="c_ile_L" compartment="cell" hasOnlySubstanceUnits="false" boundaryCondition="false" constant="false"/>

<species id="c5mta" name="c_5mta" compartment="cell" hasOnlySubstanceUnits="false" boundaryCondition="false" constant="false"/>

<species id="cfgam" name="c_fgam" compartment="cell" hasOnlySubstanceUnits="false" boundaryCondition="false" constant="false"/>

<species id="c5mtr" name="c_5mtr" compartment="cell" hasOnlySubstanceUnits="false" boundaryCondition="false" constant="false"/>

<species id="cadgcoba" name="c_adgcoba" compartment="cell" hasOnlySubstanceUnits="false" boundaryCondition="false" constant="false"/>

<species id="casntrna" name="c_asntrna" compartment="cell" hasOnlySubstanceUnits="false" boundaryCondition="false" constant="false"/>

<species id="c2mcacn" name="c_2mcacn" compartment="cell" hasOnlySubstanceUnits="false" boundaryCondition="false" constant="false"/>

<species id="cch4s" name="c_ch4s" compartment="cell" hasOnlySubstanceUnits="false" boundaryCondition="false" constant="false"/>

<species id="cglucys" name="c_glucys" compartment="cell" hasOnlySubstanceUnits="false" boundaryCondition="false" constant="false"/>

<species id="cimacp" name="c_imacp" compartment="cell" hasOnlySubstanceUnits="false" boundaryCondition="false" constant="false"/>

<species id="cproteinSonaerobic" name="c_protein_Son_aerobic" compartment="cell" hasOnlySubstanceUnits="false" boundaryCondition="false"constant="false"/>

<species id="cacac" name="c_acac" compartment="cell" hasOnlySubstanceUnits="false" boundaryCondition="false" constant="false"/>

<species id="cglu1sa" name="c_glu1sa" compartment="cell" hasOnlySubstanceUnits="false" boundaryCondition="false" constant="false"/>

<species id="csucarg" name="c_sucarg" compartment="cell" hasOnlySubstanceUnits="false" boundaryCondition="false" constant="false"/>

<species id="cppa" name="c_ppa" compartment="cell" hasOnlySubstanceUnits="false" boundaryCondition="false" constant="false"/>

<species id="ctyrL" name="c_tyr_L" compartment="cell" hasOnlySubstanceUnits="false" boundaryCondition="false" constant="false"/>

<species id="cetoh" name="c_etoh" compartment="cell" hasOnlySubstanceUnits="false" boundaryCondition="false" constant="false"/>

<species id="caccoa" name="c_accoa" compartment="cell" hasOnlySubstanceUnits="false" boundaryCondition="false" constant="false"/>

<species id="cacmalt" name="c_acmalt" compartment="cell" hasOnlySubstanceUnits="false" boundaryCondition="false" constant="false"/>

<species id="cfa11" name="c_fa11" compartment="cell" hasOnlySubstanceUnits="false" boundaryCondition="false" constant="false"/>

<species id="cfa13" name="c_fa13" compartment="cell" hasOnlySubstanceUnits="false" boundaryCondition="false" constant="false"/>

<species id="chomL" name="c_hom_L" compartment="cell" hasOnlySubstanceUnits="false" boundaryCondition="false" constant="false"/>

<species id="cidp" name="c_idp" compartment="cell" hasOnlySubstanceUnits="false" boundaryCondition="false" constant="false"/>

<species id="c2kmb" name="c_2kmb" compartment="cell" hasOnlySubstanceUnits="false" boundaryCondition="false" constant="false"/>

<species id="ctrptrna" name="c_trptrna" compartment="cell" hasOnlySubstanceUnits="false" boundaryCondition="false" constant="false"/>

<species id="c2obut" name="c_2obut" compartment="cell" hasOnlySubstanceUnits="false" boundaryCondition="false" constant="false"/>

<species id="c3dhq" name="c_3dhq" compartment="cell" hasOnlySubstanceUnits="false" boundaryCondition="false" constant="false"/>

<species id="cgal1p" name="c_gal1p" compartment="cell" hasOnlySubstanceUnits="false" boundaryCondition="false" constant="false"/>

<species id="cdhbpt" name="c_dhbpt" compartment="cell" hasOnlySubstanceUnits="false" boundaryCondition="false" constant="false"/>

<species id="cocdca" name="c_ocdca" compartment="cell" hasOnlySubstanceUnits="false" boundaryCondition="false" constant="false"/>

<species id="cthdp" name="c_thdp" compartment="cell" hasOnlySubstanceUnits="false" boundaryCondition="false" constant="false"/>

<species id="c5aizc" name="c_5aizc" compartment="cell" hasOnlySubstanceUnits="false" boundaryCondition="false" constant="false"/>

<species id="cshcl" name="c_shcl" compartment="cell" hasOnlySubstanceUnits="false" boundaryCondition="false" constant="false"/>

<species id="cindole" name="c_indole" compartment="cell" hasOnlySubstanceUnits="false" boundaryCondition="false" constant="false"/>

<species id="clacL" name="c_lac_L" compartment="cell" hasOnlySubstanceUnits="false" boundaryCondition="false" constant="false"/>

<species id="cps" name="c_ps" compartment="cell" hasOnlySubstanceUnits="false" boundaryCondition="false" constant="false"/>

<species id="clacD" name="c_lac_D" compartment="cell" hasOnlySubstanceUnits="false" boundaryCondition="false" constant="false"/>

<species id="cgmh17bp" name="c_gmh17bp" compartment="cell" hasOnlySubstanceUnits="false" boundaryCondition="false" constant="false"/>

<species id="c5odhf2a" name="c_5odhf2a" compartment="cell" hasOnlySubstanceUnits="false" boundaryCondition="false" constant="false"/>

<species id="cornL" name="c_orn_L" compartment="cell" hasOnlySubstanceUnits="false" boundaryCondition="false" constant="false"/>

<species id="cppcoa" name="c_ppcoa" compartment="cell" hasOnlySubstanceUnits="false" boundaryCondition="false" constant="false"/>

<species id="cpe" name="c_pe" compartment="cell" hasOnlySubstanceUnits="false" boundaryCondition="false" constant="false"/>

<species id="cfa11ACP" name="c_fa11ACP" compartment="cell" hasOnlySubstanceUnits="false" boundaryCondition="false" constant="false"/>

<species id="cpantR" name="c_pant_R" compartment="cell" hasOnlySubstanceUnits="false" boundaryCondition="false" constant="false"/>

<species id="cpi" name="c_pi" compartment="cell" hasOnlySubstanceUnits="false" boundaryCondition="false" constant="false"/>

<species id="ccyan" name="c_cyan" compartment="cell" hasOnlySubstanceUnits="false" boundaryCondition="false" constant="false"/>

<species id="cgmh1p" name="c_gmh1p" compartment="cell" hasOnlySubstanceUnits="false" boundaryCondition="false" constant="false"/>

<species id="cactp" name="c_actp" compartment="cell" hasOnlySubstanceUnits="false" boundaryCondition="false" constant="false"/>

<species id="cdhptd" name="c_dhptd" compartment="cell" hasOnlySubstanceUnits="false" boundaryCondition="false" constant="false"/>

<species id="csucglu" name="c_sucglu" compartment="cell" hasOnlySubstanceUnits="false" boundaryCondition="false" constant="false"/>

<species id="c5prdmbz" name="c_5prdmbz" compartment="cell" hasOnlySubstanceUnits="false" boundaryCondition="false" constant="false"/>

<species id="cnadph" name="c_nadph" compartment="cell" hasOnlySubstanceUnits="false" boundaryCondition="false" constant="false"/>

<species id="cfa8ACP" name="c_fa8ACP" compartment="cell" hasOnlySubstanceUnits="false" boundaryCondition="false" constant="false"/>

<species id="carsni2" name="c_arsni2" compartment="cell" hasOnlySubstanceUnits="false" boundaryCondition="false" constant="false"/>

<species id="c1ap2ol" name="c_1ap2ol" compartment="cell" hasOnlySubstanceUnits="false" boundaryCondition="false" constant="false"/>

<species id="cdgtp" name="c_dgtp" compartment="cell" hasOnlySubstanceUnits="false" boundaryCondition="false" constant="false"/>

<species id="cadcobdam" name="c_adcobdam" compartment="cell" hasOnlySubstanceUnits="false" boundaryCondition="false" constant="false"/>

<species id="csrch" name="c_srch" compartment="cell" hasOnlySubstanceUnits="false" boundaryCondition="false" constant="false"/>

<species id="cpmcoa" name="c_pmcoa" compartment="cell" hasOnlySubstanceUnits="false" boundaryCondition="false" constant="false"/>

<species id="cmalylcoa" name="c_malylcoa" compartment="cell" hasOnlySubstanceUnits="false" boundaryCondition="false" constant="false"/>

<species id="chpdca" name="c_hpdca" compartment="cell" hasOnlySubstanceUnits="false" boundaryCondition="false" constant="false"/>

<species id="cctp" name="c_ctp" compartment="cell" hasOnlySubstanceUnits="false" boundaryCondition="false" constant="false"/>

<species id="cpgly" name="c_pgly" compartment="cell" hasOnlySubstanceUnits="false" boundaryCondition="false" constant="false"/>

<species id="cdhnpt" name="c_dhnpt" compartment="cell" hasOnlySubstanceUnits="false" boundaryCondition="false" constant="false"/>

<species id="cfa1ACP" name="c_fa1ACP" compartment="cell" hasOnlySubstanceUnits="false" boundaryCondition="false" constant="false"/>

<species id="camob" name="c_amob" compartment="cell" hasOnlySubstanceUnits="false" boundaryCondition="false" constant="false"/>

<species id="civcoa" name="c_ivcoa" compartment="cell" hasOnlySubstanceUnits="false" boundaryCondition="false" constant="false"/>

<species id="cdutp" name="c_dutp" compartment="cell" hasOnlySubstanceUnits="false" boundaryCondition="false" constant="false"/>

<species id="casptrna" name="c_asptrna" compartment="cell" hasOnlySubstanceUnits="false" boundaryCondition="false" constant="false"/>

<species id="cgcald" name="c_gcald" compartment="cell" hasOnlySubstanceUnits="false" boundaryCondition="false" constant="false"/>

<species id="ckdo8nlipid4L" name="c_kdo8nlipid4L" compartment="cell" hasOnlySubstanceUnits="false" boundaryCondition="false" constant="false"/>

<species id="cdpcoa" name="c_dpcoa" compartment="cell" hasOnlySubstanceUnits="false" boundaryCondition="false" constant="false"/>

<species id="cdgdp" name="c_dgdp" compartment="cell" hasOnlySubstanceUnits="false" boundaryCondition="false" constant="false"/>

<species id="cudpglcur" name="c_udpglcur" compartment="cell" hasOnlySubstanceUnits="false" boundaryCondition="false" constant="false"/>

<species id="c10fthf" name="c_10fthf" compartment="cell" hasOnlySubstanceUnits="false" boundaryCondition="false" constant="false"/>

<species id="cglx" name="c_glx" compartment="cell" hasOnlySubstanceUnits="false" boundaryCondition="false" constant="false"/>

<species id="cadpglc" name="c_adpglc" compartment="cell" hasOnlySubstanceUnits="false" boundaryCondition="false" constant="false"/>

<species id="c4r5au" name="c_4r5au" compartment="cell" hasOnlySubstanceUnits="false" boundaryCondition="false" constant="false"/>

<species id="cfrlt" name="c_frlt" compartment="cell" hasOnlySubstanceUnits="false" boundaryCondition="false" constant="false"/>

<species id="cubq8h2" name="c_ubq8h2" compartment="cell" hasOnlySubstanceUnits="false" boundaryCondition="false" constant="false"/>

<species id="cglyaspL" name="c_gly_asp_L" compartment="cell" hasOnlySubstanceUnits="false" boundaryCondition="false" constant="false"/>

<species id="c5drib" name="c_5drib" compartment="cell" hasOnlySubstanceUnits="false" boundaryCondition="false" constant="false"/>

<species id="cpydx5p" name="c_pydx5p" compartment="cell" hasOnlySubstanceUnits="false" boundaryCondition="false" constant="false"/>

<species id="cnadh" name="c_nadh" compartment="cell" hasOnlySubstanceUnits="false" boundaryCondition="false" constant="false"/>

<species id="cnadp" name="c_nadp" compartment="cell" hasOnlySubstanceUnits="false" boundaryCondition="false" constant="false"/>

<species id="c2mbcoa" name="c_2mbcoa" compartment="cell" hasOnlySubstanceUnits="false" boundaryCondition="false" constant="false"/>

<species id="cdad5" name="c_dad_5" compartment="cell" hasOnlySubstanceUnits="false" boundaryCondition="false" constant="false"/>

<species id="chpdACP" name="c_hpdACP" compartment="cell" hasOnlySubstanceUnits="false" boundaryCondition="false" constant="false"/>

<species id="cglntrna" name="c_glntrna" compartment="cell" hasOnlySubstanceUnits="false" boundaryCondition="false" constant="false"/>

<species id="cg3p" name="c_g3p" compartment="cell" hasOnlySubstanceUnits="false" boundaryCondition="false" constant="false"/>

<species id="cdctp" name="c_dctp" compartment="cell" hasOnlySubstanceUnits="false" boundaryCondition="false" constant="false"/>

<species id="cmlthf" name="c_mlthf" compartment="cell" hasOnlySubstanceUnits="false" boundaryCondition="false" constant="false"/>

<species id="cfa7ACP" name="c_fa7ACP" compartment="cell" hasOnlySubstanceUnits="false" boundaryCondition="false" constant="false"/>

<species id="c2dhp" name="c_2dhp" compartment="cell" hasOnlySubstanceUnits="false" boundaryCondition="false" constant="false"/>

<species id="cdad2" name="c_dad_2" compartment="cell" hasOnlySubstanceUnits="false" boundaryCondition="false" constant="false"/>

<species id="cleuL" name="c_leu_L" compartment="cell" hasOnlySubstanceUnits="false" boundaryCondition="false" constant="false"/>

<species id="cribflv" name="c_ribflv" compartment="cell" hasOnlySubstanceUnits="false" boundaryCondition="false" constant="false"/>

<species id="calaala" name="c_alaala" compartment="cell" hasOnlySubstanceUnits="false" boundaryCondition="false" constant="false"/>

<species id="cpde" name="c_pde" compartment="cell" hasOnlySubstanceUnits="false" boundaryCondition="false" constant="false"/>

<species id="cdhap" name="c_dhap" compartment="cell" hasOnlySubstanceUnits="false" boundaryCondition="false" constant="false"/>

<species id="cfor" name="c_for" compartment="cell" hasOnlySubstanceUnits="false" boundaryCondition="false" constant="false"/>

<species id="cfprica" name="c_fprica" compartment="cell" hasOnlySubstanceUnits="false" boundaryCondition="false" constant="false"/>

<species id="cadcoba" name="c_adcoba" compartment="cell" hasOnlySubstanceUnits="false" boundaryCondition="false" constant="false"/>

<species id="c2ahhmp" name="c_2ahhmp" compartment="cell" hasOnlySubstanceUnits="false" boundaryCondition="false" constant="false"/>

<species id="cu3aga" name="c_u3aga" compartment="cell" hasOnlySubstanceUnits="false" boundaryCondition="false" constant="false"/>

<species id="cmalcoa" name="c_malcoa" compartment="cell" hasOnlySubstanceUnits="false" boundaryCondition="false" constant="false"/>

<species id="cskm5p" name="c_skm5p" compartment="cell" hasOnlySubstanceUnits="false" boundaryCondition="false" constant="false"/>

<species id="chco3" name="c_hco3" compartment="cell" hasOnlySubstanceUnits="false" boundaryCondition="false" constant="false"/>

<species id="c2ahhmd" name="c_2ahhmd" compartment="cell" hasOnlySubstanceUnits="false" boundaryCondition="false" constant="false"/>

<species id="c5aop" name="c_5aop" compartment="cell" hasOnlySubstanceUnits="false" boundaryCondition="false" constant="false"/>

<species id="cdadp" name="c_dadp" compartment="cell" hasOnlySubstanceUnits="false" boundaryCondition="false" constant="false"/>

<species id="chexdp" name="c_hexdp" compartment="cell" hasOnlySubstanceUnits="false" boundaryCondition="false" constant="false"/>

<species id="crnaSon" name="c_rna_Son" compartment="cell" hasOnlySubstanceUnits="false" boundaryCondition="false" constant="false"/>

<species id="cggluaba" name="c_ggluaba" compartment="cell" hasOnlySubstanceUnits="false" boundaryCondition="false" constant="false"/>

<species id="c5aprbu" name="c_5aprbu" compartment="cell" hasOnlySubstanceUnits="false" boundaryCondition="false" constant="false"/>

<species id="canth" name="c_anth" compartment="cell" hasOnlySubstanceUnits="false" boundaryCondition="false" constant="false"/>

<species id="chxan" name="c_hxan" compartment="cell" hasOnlySubstanceUnits="false" boundaryCondition="false" constant="false"/>

<species id="cmi1pD" name="c_mi1p_D" compartment="cell" hasOnlySubstanceUnits="false" boundaryCondition="false" constant="false"/>

<species id="corot5p" name="c_orot5p" compartment="cell" hasOnlySubstanceUnits="false" boundaryCondition="false" constant="false"/>

<species id="c5mdru1p" name="c_5mdru1p" compartment="cell" hasOnlySubstanceUnits="false" boundaryCondition="false" constant="false"/>

<species id="ccbp" name="c_cbp" compartment="cell" hasOnlySubstanceUnits="false" boundaryCondition="false" constant="false"/>

<species id="cggluabt" name="c_ggluabt" compartment="cell" hasOnlySubstanceUnits="false" boundaryCondition="false" constant="false"/>

<species id="ccbl1" name="c_cbl1" compartment="cell" hasOnlySubstanceUnits="false" boundaryCondition="false" constant="false"/>

<species id="cgsn" name="c_gsn" compartment="cell" hasOnlySubstanceUnits="false" boundaryCondition="false" constant="false"/>

<species id="cutp" name="c_utp" compartment="cell" hasOnlySubstanceUnits="false" boundaryCondition="false" constant="false"/>

<species id="c5mdr1p" name="c_5mdr1p" compartment="cell" hasOnlySubstanceUnits="false" boundaryCondition="false" constant="false"/>

<species id="ceig3p" name="c_eig3p" compartment="cell" hasOnlySubstanceUnits="false" boundaryCondition="false" constant="false"/>

<species id="ciasp" name="c_iasp" compartment="cell" hasOnlySubstanceUnits="false" boundaryCondition="false" constant="false"/>

<species id="c1pyr5c" name="c_1pyr5c" compartment="cell" hasOnlySubstanceUnits="false" boundaryCondition="false" constant="false"/>

<species id="cfpram" name="c_fpram" compartment="cell" hasOnlySubstanceUnits="false" boundaryCondition="false" constant="false"/>

<species id="c4ampm" name="c_4ampm" compartment="cell" hasOnlySubstanceUnits="false" boundaryCondition="false" constant="false"/>

<species id="cckdo8n" name="c_ckdo8n" compartment="cell" hasOnlySubstanceUnits="false" boundaryCondition="false" constant="false"/>

<species id="cdrib" name="c_drib" compartment="cell" hasOnlySubstanceUnits="false" boundaryCondition="false" constant="false"/>

<species id="cakg" name="c_akg" compartment="cell" hasOnlySubstanceUnits="false" boundaryCondition="false" constant="false"/>

<species id="cocteACP" name="c_octeACP" compartment="cell" hasOnlySubstanceUnits="false" boundaryCondition="false" constant="false"/>

<species id="cfdxr42" name="c_fdxr_4_2" compartment="cell" hasOnlySubstanceUnits="false" boundaryCondition="false" constant="false"/>

<species id="ce4p" name="c_e4p" compartment="cell" hasOnlySubstanceUnits="false" boundaryCondition="false" constant="false"/>

<species id="cmqn7" name="c_mqn7" compartment="cell" hasOnlySubstanceUnits="false" boundaryCondition="false" constant="false"/>

<species id="cskm" name="c_skm" compartment="cell" hasOnlySubstanceUnits="false" boundaryCondition="false" constant="false"/>

<species id="cthrtrna" name="c_thrtrna" compartment="cell" hasOnlySubstanceUnits="false" boundaryCondition="false" constant="false"/>

<species id="c25aics" name="c_25aics" compartment="cell" hasOnlySubstanceUnits="false" boundaryCondition="false" constant="false"/>

<species id="cubq8" name="c_ubq8" compartment="cell" hasOnlySubstanceUnits="false" boundaryCondition="false" constant="false"/>

<species id="chgentis" name="c_hgentis" compartment="cell" hasOnlySubstanceUnits="false" boundaryCondition="false" constant="false"/>

<species id="c2dmmq7" name="c_2dmmq7" compartment="cell" hasOnlySubstanceUnits="false" boundaryCondition="false" constant="false"/>

<species id="cvalL" name="c_val_L" compartment="cell" hasOnlySubstanceUnits="false" boundaryCondition="false" constant="false"/>

<species id="corot" name="c_orot" compartment="cell" hasOnlySubstanceUnits="false" boundaryCondition="false" constant="false"/>

<species id="cru5pD" name="c_ru5p_D" compartment="cell" hasOnlySubstanceUnits="false" boundaryCondition="false" constant="false"/>

<species id="cacmama" name="c_acmama" compartment="cell" hasOnlySubstanceUnits="false" boundaryCondition="false" constant="false"/>

<species id="cpyr" name="c_pyr" compartment="cell" hasOnlySubstanceUnits="false" boundaryCondition="false" constant="false"/>

<species id="cg6p" name="c_g6p" compartment="cell" hasOnlySubstanceUnits="false" boundaryCondition="false" constant="false"/>

<species id="c2dda7p" name="c_2dda7p" compartment="cell" hasOnlySubstanceUnits="false" boundaryCondition="false" constant="false"/>

<species id="c2pg" name="c_2pg" compartment="cell" hasOnlySubstanceUnits="false" boundaryCondition="false" constant="false"/>

<species id="cgal" name="c_gal" compartment="cell" hasOnlySubstanceUnits="false" boundaryCondition="false" constant="false"/>

<species id="cpan4p" name="c_pan4p" compartment="cell" hasOnlySubstanceUnits="false" boundaryCondition="false" constant="false"/>

<species id="c2p4c2me" name="c_2p4c2me" compartment="cell" hasOnlySubstanceUnits="false" boundaryCondition="false" constant="false"/>

<species id="ctrnaarg" name="c_trnaarg" compartment="cell" hasOnlySubstanceUnits="false" boundaryCondition="false" constant="false"/>

<species id="chistrna" name="c_histrna" compartment="cell" hasOnlySubstanceUnits="false" boundaryCondition="false" constant="false"/>

<species id="c4c2me" name="c_4c2me" compartment="cell" hasOnlySubstanceUnits="false" boundaryCondition="false" constant="false"/>

<species id="cgar" name="c_gar" compartment="cell" hasOnlySubstanceUnits="false" boundaryCondition="false" constant="false"/>

<species id="c5apru" name="c_5apru" compartment="cell" hasOnlySubstanceUnits="false" boundaryCondition="false" constant="false"/>

<species id="cdtbt" name="c_dtbt" compartment="cell" hasOnlySubstanceUnits="false" boundaryCondition="false" constant="false"/>

<species id="c5mthf" name="c_5mthf" compartment="cell" hasOnlySubstanceUnits="false" boundaryCondition="false" constant="false"/>

<species id="c2mecdp" name="c_2mecdp" compartment="cell" hasOnlySubstanceUnits="false" boundaryCondition="false" constant="false"/>

<species id="citp" name="c_itp" compartment="cell" hasOnlySubstanceUnits="false" boundaryCondition="false" constant="false"/>

<species id="cdhf" name="c_dhf" compartment="cell" hasOnlySubstanceUnits="false" boundaryCondition="false" constant="false"/>

<species id="cdkmpp" name="c_dkmpp" compartment="cell" hasOnlySubstanceUnits="false" boundaryCondition="false" constant="false"/>

<species id="ccobamcoa" name="c_cobamcoa" compartment="cell" hasOnlySubstanceUnits="false" boundaryCondition="false" constant="false"/>

<species id="cglnL" name="c_gln_L" compartment="cell" hasOnlySubstanceUnits="false" boundaryCondition="false" constant="false"/>

<species id="cmaltttr" name="c_maltttr" compartment="cell" hasOnlySubstanceUnits="false" boundaryCondition="false" constant="false"/>

<species id="cglu5sa" name="c_glu5sa" compartment="cell" hasOnlySubstanceUnits="false" boundaryCondition="false" constant="false"/>

<species id="cproL" name="c_pro_L" compartment="cell" hasOnlySubstanceUnits="false" boundaryCondition="false" constant="false"/>

<species id="cuama" name="c_uama" compartment="cell" hasOnlySubstanceUnits="false" boundaryCondition="false" constant="false"/>

<species id="cpram" name="c_pram" compartment="cell" hasOnlySubstanceUnits="false" boundaryCondition="false" constant="false"/>

<species id="cpran" name="c_pran" compartment="cell" hasOnlySubstanceUnits="false" boundaryCondition="false" constant="false"/>

<species id="cgluD" name="c_glu_D" compartment="cell" hasOnlySubstanceUnits="false" boundaryCondition="false" constant="false"/>

<species id="cmmalsa" name="c_mmalsa" compartment="cell" hasOnlySubstanceUnits="false" boundaryCondition="false" constant="false"/>

<species id="cmalthx" name="c_malthx" compartment="cell" hasOnlySubstanceUnits="false" boundaryCondition="false" constant="false"/>

<species id="c4hbcoa" name="c_4hbcoa" compartment="cell" hasOnlySubstanceUnits="false" boundaryCondition="false" constant="false"/>

<species id="cmalthp" name="c_malthp" compartment="cell" hasOnlySubstanceUnits="false" boundaryCondition="false" constant="false"/>

<species id="cgdptp" name="c_gdptp" compartment="cell" hasOnlySubstanceUnits="false" boundaryCondition="false" constant="false"/>

<species id="cfum" name="c_fum" compartment="cell" hasOnlySubstanceUnits="false" boundaryCondition="false" constant="false"/>

<species id="cuamag" name="c_uamag" compartment="cell" hasOnlySubstanceUnits="false" boundaryCondition="false" constant="false"/>

<species id="c23dhmb" name="c_23dhmb" compartment="cell" hasOnlySubstanceUnits="false" boundaryCondition="false" constant="false"/>

<species id="cdann" name="c_dann" compartment="cell" hasOnlySubstanceUnits="false" boundaryCondition="false" constant="false"/>

<species id="cbtcoa" name="c_btcoa" compartment="cell" hasOnlySubstanceUnits="false" boundaryCondition="false" constant="false"/>

<species name="c_adphep_D,D" compartment="cell" hasOnlySubstanceUnits="false" boundaryCondition="false" constant="false"/>

<species id="c23dhmp" name="c_23dhmp" compartment="cell" hasOnlySubstanceUnits="false" boundaryCondition="false" constant="false"/>

<species id="cap4a" name="c_ap4a" compartment="cell" hasOnlySubstanceUnits="false" boundaryCondition="false" constant="false"/>

<species id="ccoa" name="c_coa" compartment="cell" hasOnlySubstanceUnits="false" boundaryCondition="false" constant="false"/>

<species id="cso3" name="c_so3" compartment="cell" hasOnlySubstanceUnits="false" boundaryCondition="false" constant="false"/>

<species id="chmbil" name="c_hmbil" compartment="cell" hasOnlySubstanceUnits="false" boundaryCondition="false" constant="false"/>

<species id="cso4" name="c_so4" compartment="cell" hasOnlySubstanceUnits="false" boundaryCondition="false" constant="false"/>

<species id="cprbatp" name="c_prbatp" compartment="cell" hasOnlySubstanceUnits="false" boundaryCondition="false" constant="false"/>

<species id="ccpppg3" name="c_cpppg3" compartment="cell" hasOnlySubstanceUnits="false" boundaryCondition="false" constant="false"/>

<species id="cgluL" name="c_glu_L" compartment="cell" hasOnlySubstanceUnits="false" boundaryCondition="false" constant="false"/>

<species id="cACP" name="c_ACP" compartment="cell" hasOnlySubstanceUnits="false" boundaryCondition="false" constant="false"/>

<species id="csucbz" name="c_sucbz" compartment="cell" hasOnlySubstanceUnits="false" boundaryCondition="false" constant="false"/>

<species id="cacser" name="c_acser" compartment="cell" hasOnlySubstanceUnits="false" boundaryCondition="false" constant="false"/>

<species id="cphom" name="c_phom" compartment="cell" hasOnlySubstanceUnits="false" boundaryCondition="false" constant="false"/>

<species id="c4adcho" name="c_4adcho" compartment="cell" hasOnlySubstanceUnits="false" boundaryCondition="false" constant="false"/>

<species id="cmmqn7" name="c_mmqn7" compartment="cell" hasOnlySubstanceUnits="false" boundaryCondition="false" constant="false"/>

<species id="clysL" name="c_lys_L" compartment="cell" hasOnlySubstanceUnits="false" boundaryCondition="false" constant="false"/>

<species id="caicar" name="c_aicar" compartment="cell" hasOnlySubstanceUnits="false" boundaryCondition="false" constant="false"/>

<species id="c2omph" name="c_2omph" compartment="cell" hasOnlySubstanceUnits="false" boundaryCondition="false" constant="false"/>

<species id="cudpgal" name="c_udpgal" compartment="cell" hasOnlySubstanceUnits="false" boundaryCondition="false" constant="false"/>

<species id="cpglyp" name="c_pglyp" compartment="cell" hasOnlySubstanceUnits="false" boundaryCondition="false" constant="false"/>

<species id="cpdACP" name="c_pdACP" compartment="cell" hasOnlySubstanceUnits="false" boundaryCondition="false" constant="false"/>

<species id="ctrdox" name="c_trdox" compartment="cell" hasOnlySubstanceUnits="false" boundaryCondition="false" constant="false"/>

<species id="cimp" name="c_imp" compartment="cell" hasOnlySubstanceUnits="false" boundaryCondition="false" constant="false"/>

<species id="c3ophb" name="c_3ophb" compartment="cell" hasOnlySubstanceUnits="false" boundaryCondition="false" constant="false"/>

<species id="ccm" name="c_cm" compartment="cell" hasOnlySubstanceUnits="false" boundaryCondition="false" constant="false"/>

<species id="cade" name="c_ade" compartment="cell" hasOnlySubstanceUnits="false" boundaryCondition="false" constant="false"/>

<species id="cadn" name="c_adn" compartment="cell" hasOnlySubstanceUnits="false" boundaryCondition="false" constant="false"/>

<species id="chpyr" name="c_hpyr" compartment="cell" hasOnlySubstanceUnits="false" boundaryCondition="false" constant="false"/>

<species id="ctrnathr" name="c_trnathr" compartment="cell" hasOnlySubstanceUnits="false" boundaryCondition="false" constant="false"/>

<species id="cmettrna" name="c_mettrna" compartment="cell" hasOnlySubstanceUnits="false" boundaryCondition="false" constant="false"/>

<species id="c8aonn" name="c_8aonn" compartment="cell" hasOnlySubstanceUnits="false" boundaryCondition="false" constant="false"/>

<species id="cadp" name="c_adp" compartment="cell" hasOnlySubstanceUnits="false" boundaryCondition="false" constant="false"/>

<species id="cco2" name="c_co2" compartment="cell" hasOnlySubstanceUnits="false" boundaryCondition="false" constant="false"/>

<species id="chcysL" name="c_hcys_L" compartment="cell" hasOnlySubstanceUnits="false" boundaryCondition="false" constant="false"/>

<species id="cbut" name="c_but" compartment="cell" hasOnlySubstanceUnits="false" boundaryCondition="false" constant="false"/>

<species id="cmalt" name="c_malt" compartment="cell" hasOnlySubstanceUnits="false" boundaryCondition="false" constant="false"/>

<species id="c3psme" name="c_3psme" compartment="cell" hasOnlySubstanceUnits="false" boundaryCondition="false" constant="false"/>

<species id="cgthrd" name="c_gthrd" compartment="cell" hasOnlySubstanceUnits="false" boundaryCondition="false" constant="false"/>

<species id="ctyrtrna" name="c_tyrtrna" compartment="cell" hasOnlySubstanceUnits="false" boundaryCondition="false" constant="false"/>

<species id="cpep" name="c_pep" compartment="cell" hasOnlySubstanceUnits="false" boundaryCondition="false" constant="false"/>

<species id="c5caiz" name="c_5caiz" compartment="cell" hasOnlySubstanceUnits="false" boundaryCondition="false" constant="false"/>

<species id="c2me4p" name="c_2me4p" compartment="cell" hasOnlySubstanceUnits="false" boundaryCondition="false" constant="false"/>

<species id="cttdca" name="c_ttdca" compartment="cell" hasOnlySubstanceUnits="false" boundaryCondition="false" constant="false"/>

<species id="cnh4" name="c_nh4" compartment="cell" hasOnlySubstanceUnits="false" boundaryCondition="false" constant="false"/>

<species id="cuagnr" name="c_uagnr" compartment="cell" hasOnlySubstanceUnits="false" boundaryCondition="false" constant="false"/>

<species id="chemeO" name="c_hemeO" compartment="cell" hasOnlySubstanceUnits="false" boundaryCondition="false" constant="false"/>

<species id="c4pasp" name="c_4pasp" compartment="cell" hasOnlySubstanceUnits="false" boundaryCondition="false" constant="false"/>

<species id="cphpyr" name="c_phpyr" compartment="cell" hasOnlySubstanceUnits="false" boundaryCondition="false" constant="false"/>

<species id="cacgam1p" name="c_acgam1p" compartment="cell" hasOnlySubstanceUnits="false" boundaryCondition="false" constant="false"/>

<species id="cfglutS" name="c_fglut_S" compartment="cell" hasOnlySubstanceUnits="false" boundaryCondition="false" constant="false"/>

<species id="cnac" name="c_nac" compartment="cell" hasOnlySubstanceUnits="false" boundaryCondition="false" constant="false"/>

<species id="cinost" name="c_inost" compartment="cell" hasOnlySubstanceUnits="false" boundaryCondition="false" constant="false"/>

<species id="cnad" name="c_nad" compartment="cell" hasOnlySubstanceUnits="false" boundaryCondition="false" constant="false"/>

<species id="ccinnm" name="c_cinnm" compartment="cell" hasOnlySubstanceUnits="false" boundaryCondition="false" constant="false"/>

<species id="c4mpetz" name="c_4mpetz" compartment="cell" hasOnlySubstanceUnits="false" boundaryCondition="false" constant="false"/>

<species id="cap5a" name="c_ap5a" compartment="cell" hasOnlySubstanceUnits="false" boundaryCondition="false" constant="false"/>

<species id="cdcamp" name="c_dcamp" compartment="cell" hasOnlySubstanceUnits="false" boundaryCondition="false" constant="false"/>

<species id="cpalmACP" name="c_palmACP" compartment="cell" hasOnlySubstanceUnits="false" boundaryCondition="false" constant="false"/>

<species id="cprfp" name="c_prfp" compartment="cell" hasOnlySubstanceUnits="false" boundaryCondition="false" constant="false"/>

<species id="calaL" name="c_ala_L" compartment="cell" hasOnlySubstanceUnits="false" boundaryCondition="false" constant="false"/>

<species id="ctsul" name="c_tsul" compartment="cell" hasOnlySubstanceUnits="false" boundaryCondition="false" constant="false"/>

<species id="cadcobap" name="c_adcobap" compartment="cell" hasOnlySubstanceUnits="false" boundaryCondition="false" constant="false"/>

<species id="calaB" name="c_ala_B" compartment="cell" hasOnlySubstanceUnits="false" boundaryCondition="false" constant="false"/>

<species id="c2ommbl" name="c_2ommbl" compartment="cell" hasOnlySubstanceUnits="false" boundaryCondition="false" constant="false"/>

<species id="calaD" name="c_ala_D" compartment="cell" hasOnlySubstanceUnits="false" boundaryCondition="false" constant="false"/>

<species id="cserL" name="c_ser_L" compartment="cell" hasOnlySubstanceUnits="false" boundaryCondition="false" constant="false"/>

<species id="cxan" name="c_xan" compartment="cell" hasOnlySubstanceUnits="false" boundaryCondition="false" constant="false"/>

<species id="cdhpt" name="c_dhpt" compartment="cell" hasOnlySubstanceUnits="false" boundaryCondition="false" constant="false"/>

<species id="cmalttr" name="c_malttr" compartment="cell" hasOnlySubstanceUnits="false" boundaryCondition="false" constant="false"/>

<species id="cpyam5p" name="c_pyam5p" compartment="cell" hasOnlySubstanceUnits="false" boundaryCondition="false" constant="false"/>

<species id="cpserL" name="c_pser_L" compartment="cell" hasOnlySubstanceUnits="false" boundaryCondition="false" constant="false"/>

<species id="cxtsn" name="c_xtsn" compartment="cell" hasOnlySubstanceUnits="false" boundaryCondition="false" constant="false"/>

<species id="cfmettrna" name="c_fmettrna" compartment="cell" hasOnlySubstanceUnits="false" boundaryCondition="false" constant="false"/>

<species id="cprpp" name="c_prpp" compartment="cell" hasOnlySubstanceUnits="false" boundaryCondition="false" constant="false"/>

<species id="cfa6ACP" name="c_fa6ACP" compartment="cell" hasOnlySubstanceUnits="false" boundaryCondition="false" constant="false"/>

<species id="cmyrsACP" name="c_myrsACP" compartment="cell" hasOnlySubstanceUnits="false" boundaryCondition="false" constant="false"/>

<species id="c3mb2coa" name="c_3mb2coa" compartment="cell" hasOnlySubstanceUnits="false" boundaryCondition="false" constant="false"/>

<species id="c3c3hmp" name="c_3c3hmp" compartment="cell" hasOnlySubstanceUnits="false" boundaryCondition="false" constant="false"/>

<species id="cgthox" name="c_gthox" compartment="cell" hasOnlySubstanceUnits="false" boundaryCondition="false" constant="false"/>

<species id="cpdeACP" name="c_pdeACP" compartment="cell" hasOnlySubstanceUnits="false" boundaryCondition="false" constant="false"/>

<species id="cselnp" name="c_selnp" compartment="cell" hasOnlySubstanceUnits="false" boundaryCondition="false" constant="false"/>

<species id="cxmp" name="c_xmp" compartment="cell" hasOnlySubstanceUnits="false" boundaryCondition="false" constant="false"/>

<species id="crhcys" name="c_rhcys" compartment="cell" hasOnlySubstanceUnits="false" boundaryCondition="false" constant="false"/>

<species id="cocdACP" name="c_ocdACP" compartment="cell" hasOnlySubstanceUnits="false" boundaryCondition="false" constant="false"/>

<species id="c2mahmp" name="c_2mahmp" compartment="cell" hasOnlySubstanceUnits="false" boundaryCondition="false" constant="false"/>

<species id="cahdt" name="c_ahdt" compartment="cell" hasOnlySubstanceUnits="false" boundaryCondition="false" constant="false"/>

<species id="c2ippm" name="c_2ippm" compartment="cell" hasOnlySubstanceUnits="false" boundaryCondition="false" constant="false"/>

<species id="cpdx5p" name="c_pdx5p" compartment="cell" hasOnlySubstanceUnits="false" boundaryCondition="false" constant="false"/>

<species id="chpdeACP" name="c_hpdeACP" compartment="cell" hasOnlySubstanceUnits="false" boundaryCondition="false" constant="false"/>

<species id="cg3pg" name="c_g3pg" compartment="cell" hasOnlySubstanceUnits="false" boundaryCondition="false" constant="false"/>

<species id="cg3pe" name="c_g3pe" compartment="cell" hasOnlySubstanceUnits="false" boundaryCondition="false" constant="false"/>

<species id="ctrnaser" name="c_trnaser" compartment="cell" hasOnlySubstanceUnits="false" boundaryCondition="false" constant="false"/>

<species id="ccysthL" name="c_cysth_L" compartment="cell" hasOnlySubstanceUnits="false" boundaryCondition="false" constant="false"/>

<species id="ccsn" name="c_csn" compartment="cell" hasOnlySubstanceUnits="false" boundaryCondition="false" constant="false"/>

<species id="cdhorS" name="c_dhor_S" compartment="cell" hasOnlySubstanceUnits="false" boundaryCondition="false" constant="false"/>

<species id="cglcD" name="c_glc_D" compartment="cell" hasOnlySubstanceUnits="false" boundaryCondition="false" constant="false"/>

<species id="c78dhp" name="c_78dhp" compartment="cell" hasOnlySubstanceUnits="false" boundaryCondition="false" constant="false"/>

<species id="c3c2hmp" name="c_3c2hmp" compartment="cell" hasOnlySubstanceUnits="false" boundaryCondition="false" constant="false"/>

<species id="cacg5p" name="c_acg5p" compartment="cell" hasOnlySubstanceUnits="false" boundaryCondition="false" constant="false"/>

<species id="ctrnalys" name="c_trnalys" compartment="cell" hasOnlySubstanceUnits="false" boundaryCondition="false" constant="false"/>

<species id="c3php" name="c_3php" compartment="cell" hasOnlySubstanceUnits="false" boundaryCondition="false" constant="false"/>

<species id="ckdo" name="c_kdo" compartment="cell" hasOnlySubstanceUnits="false" boundaryCondition="false" constant="false"/>

<species id="c4abz" name="c_4abz" compartment="cell" hasOnlySubstanceUnits="false" boundaryCondition="false" constant="false"/>

<species id="c2ombzl" name="c_2ombzl" compartment="cell" hasOnlySubstanceUnits="false" boundaryCondition="false" constant="false"/>

<species id="cdttp" name="c_dttp" compartment="cell" hasOnlySubstanceUnits="false" boundaryCondition="false" constant="false"/>

<species id="cuacgala" name="c_uacgala" compartment="cell" hasOnlySubstanceUnits="false" boundaryCondition="false" constant="false"/>

<species id="cudcpp" name="c_udcpp" compartment="cell" hasOnlySubstanceUnits="false" boundaryCondition="false" constant="false"/>

<species id="cacorn" name="c_acorn" compartment="cell" hasOnlySubstanceUnits="false" boundaryCondition="false" constant="false"/>

<species id="ckdo8nlipa" name="c_kdo8nlipa" compartment="cell" hasOnlySubstanceUnits="false" boundaryCondition="false" constant="false"/>

<species id="cquln" name="c_quln" compartment="cell" hasOnlySubstanceUnits="false" boundaryCondition="false" constant="false"/>

<species id="cgdp" name="c_gdp" compartment="cell" hasOnlySubstanceUnits="false" boundaryCondition="false" constant="false"/>

<species id="ch2o2" name="c_h2o2" compartment="cell" hasOnlySubstanceUnits="false" boundaryCondition="false" constant="false"/>

<species id="cs7p" name="c_s7p" compartment="cell" hasOnlySubstanceUnits="false" boundaryCondition="false" constant="false"/>

<species id="cckdo" name="c_ckdo" compartment="cell" hasOnlySubstanceUnits="false" boundaryCondition="false" constant="false"/>

<species id="chdca" name="c_hdca" compartment="cell" hasOnlySubstanceUnits="false" boundaryCondition="false" constant="false"/>

<species id="cfdp" name="c_fdp" compartment="cell" hasOnlySubstanceUnits="false" boundaryCondition="false" constant="false"/>

<species id="coctdp" name="c_octdp" compartment="cell" hasOnlySubstanceUnits="false" boundaryCondition="false" constant="false"/>

<species id="cglytrna" name="c_glytrna" compartment="cell" hasOnlySubstanceUnits="false" boundaryCondition="false" constant="false"/>

<species id="ccitrL" name="c_citr_L" compartment="cell" hasOnlySubstanceUnits="false" boundaryCondition="false" constant="false"/>

<species id="cdb4p" name="c_db4p" compartment="cell" hasOnlySubstanceUnits="false" boundaryCondition="false" constant="false"/>

<species id="cugmda" name="c_ugmda" compartment="cell" hasOnlySubstanceUnits="false" boundaryCondition="false" constant="false"/>

<species id="csuchms" name="c_suchms" compartment="cell" hasOnlySubstanceUnits="false" boundaryCondition="false" constant="false"/>

<species id="ctrnatrp" name="c_trnatrp" compartment="cell" hasOnlySubstanceUnits="false" boundaryCondition="false" constant="false"/>

<species id="cnmn" name="c_nmn" compartment="cell" hasOnlySubstanceUnits="false" boundaryCondition="false" constant="false"/>

<species id="cdatp" name="c_datp" compartment="cell" hasOnlySubstanceUnits="false" boundaryCondition="false" constant="false"/>

<species id="cacg5sa" name="c_acg5sa" compartment="cell" hasOnlySubstanceUnits="false" boundaryCondition="false" constant="false"/>

<species id="chisp" name="c_hisp" compartment="cell" hasOnlySubstanceUnits="false" boundaryCondition="false" constant="false"/>

<species id="ctrpL" name="c_trp_L" compartment="cell" hasOnlySubstanceUnits="false" boundaryCondition="false" constant="false"/>

<species id="cptrc" name="c_ptrc" compartment="cell" hasOnlySubstanceUnits="false" boundaryCondition="false" constant="false"/>

<species id="cthrL" name="c_thr_L" compartment="cell" hasOnlySubstanceUnits="false" boundaryCondition="false" constant="false"/>

<species id="cu23ga" name="c_u23ga" compartment="cell" hasOnlySubstanceUnits="false" boundaryCondition="false" constant="false"/>

<species id="cuaccg" name="c_uaccg" compartment="cell" hasOnlySubstanceUnits="false" boundaryCondition="false" constant="false"/>

<species id="c3mgcoa" name="c_3mgcoa" compartment="cell" hasOnlySubstanceUnits="false" boundaryCondition="false" constant="false"/>

<species id="c2shchc" name="c_2shchc" compartment="cell" hasOnlySubstanceUnits="false" boundaryCondition="false" constant="false"/>

<species id="ccystrna" name="c_cystrna" compartment="cell" hasOnlySubstanceUnits="false" boundaryCondition="false" constant="false"/>

<species id="cdtdpglc" name="c_dtdpglc" compartment="cell" hasOnlySubstanceUnits="false" boundaryCondition="false" constant="false"/>

<species id="c26dapM" name="c_26dap_M" compartment="cell" hasOnlySubstanceUnits="false" boundaryCondition="false" constant="false"/>

<species id="cmercppyr" name="c_mercppyr" compartment="cell" hasOnlySubstanceUnits="false" boundaryCondition="false" constant="false"/>

<species id="cdnad" name="c_dnad" compartment="cell" hasOnlySubstanceUnits="false" boundaryCondition="false" constant="false"/>

<species id="cggluptrc" name="c_ggluptrc" compartment="cell" hasOnlySubstanceUnits="false" boundaryCondition="false" constant="false"/>

<species id="cmaltpt" name="c_maltpt" compartment="cell" hasOnlySubstanceUnits="false" boundaryCondition="false" constant="false"/>

<species id="c34hpp" name="c_34hpp" compartment="cell" hasOnlySubstanceUnits="false" boundaryCondition="false" constant="false"/>

<species id="cdamp" name="c_damp" compartment="cell" hasOnlySubstanceUnits="false" boundaryCondition="false" constant="false"/>

<species id="cdtmp" name="c_dtmp" compartment="cell" hasOnlySubstanceUnits="false" boundaryCondition="false" constant="false"/>

<species id="cthmpp" name="c_thmpp" compartment="cell" hasOnlySubstanceUnits="false" boundaryCondition="false" constant="false"/>

<species id="c4per" name="c_4per" compartment="cell" hasOnlySubstanceUnits="false" boundaryCondition="false" constant="false"/>

<species id="c3htdACP" name="c_3htdACP" compartment="cell" hasOnlySubstanceUnits="false" boundaryCondition="false" constant="false"/>

<species id="csheme" name="c_sheme" compartment="cell" hasOnlySubstanceUnits="false" boundaryCondition="false" constant="false"/>

<species id="cdtdpddm" name="c_dtdpddm" compartment="cell" hasOnlySubstanceUnits="false" boundaryCondition="false" constant="false"/>

<species id="clystrna" name="c_lystrna" compartment="cell" hasOnlySubstanceUnits="false" boundaryCondition="false" constant="false"/>

<species id="chpde" name="c_hpde" compartment="cell" hasOnlySubstanceUnits="false" boundaryCondition="false" constant="false"/>

<species id="cdtdpddg" name="c_dtdpddg" compartment="cell" hasOnlySubstanceUnits="false" boundaryCondition="false" constant="false"/>

<species id="c2aobut" name="c_2aobut" compartment="cell" hasOnlySubstanceUnits="false" boundaryCondition="false" constant="false"/>

<species id="crdmbzi" name="c_rdmbzi" compartment="cell" hasOnlySubstanceUnits="false" boundaryCondition="false" constant="false"/>

</listOfSpecies>

<listOfReactions>

<reaction id="CSND" name="CSND" reversible="false" fast="false" compartment="cell">

<listOfReactants>

<speciesReference species="c_csn" stoichiometry="1" constant="false"/>

<speciesReference species="c_h" stoichiometry="1" constant="false"/>

<speciesReference species="c_h2o" stoichiometry="1" constant="false"/>

</listOfReactants>

<listOfProducts>

<speciesReference species="c_nh4" stoichiometry="1" constant="false"/>

<speciesReference species="c_ura" stoichiometry="1" constant="false"/>

</listOfProducts>

</reaction>

<reaction id="PMDPHT" name="PMDPHT" reversible="false" fast="false" compartment="cell">

<listOfReactants>

<speciesReference species="c_5aprbu" stoichiometry="1" constant="false"/>

<speciesReference species="c_h2o" stoichiometry="1" constant="false"/>

</listOfReactants>

<listOfProducts>

<speciesReference species="c_4r5au" stoichiometry="1" constant="false"/>

<speciesReference species="c_pi" stoichiometry="1" constant="false"/>

</listOfProducts>

</reaction>

<reaction id="DHNPA" name="DHNPA" reversible="false" fast="false" compartment="cell">

<listOfReactants>

<speciesReference species="c_dhnpt" stoichiometry="1" constant="false"/>

</listOfReactants>

<listOfProducts>

<speciesReference species="c_2ahhmp" stoichiometry="1" constant="false"/>

<speciesReference species="c_gcald" stoichiometry="1" constant="false"/>

</listOfProducts>

</reaction>

<reaction id="DRBK" name="DRBK" reversible="false" fast="false" compartment="cell">

<listOfReactants>

<speciesReference species="c_atp" stoichiometry="1" constant="false"/>

<speciesReference species="c_drib" stoichiometry="1" constant="false"/>

</listOfReactants>

<listOfProducts>

<speciesReference species="c_2dr5p" stoichiometry="1" constant="false"/>

<speciesReference species="c_adp" stoichiometry="1" constant="false"/>

<speciesReference species="c_h" stoichiometry="1" constant="false"/>

</listOfProducts>

</reaction>

<reaction id="NTD5" name="NTD5" reversible="false" fast="false" compartment="cell">

<listOfReactants>

<speciesReference species="c_dtmp" stoichiometry="1" constant="false"/>

<speciesReference species="c_h2o" stoichiometry="1" constant="false"/>

</listOfReactants>

<listOfProducts>

<speciesReference species="c_pi" stoichiometry="1" constant="false"/>

<speciesReference species="c_thymd" stoichiometry="1" constant="false"/>

</listOfProducts>

</reaction>

<reaction id="GUAPRT" name="GUAPRT" reversible="false" fast="false" compartment="cell">

<listOfReactants>

<speciesReference species="c_gua" stoichiometry="1" constant="false"/>

<speciesReference species="c_prpp" stoichiometry="1" constant="false"/>

</listOfReactants>

<listOfProducts>

<speciesReference species="c_gmp" stoichiometry="1" constant="false"/>

<speciesReference species="c_ppi" stoichiometry="1" constant="false"/>

</listOfProducts>

</reaction>

<reaction id="NTD10" name="NTD10" reversible="false" fast="false" compartment="cell">

<listOfReactants>

<speciesReference species="c_h2o" stoichiometry="1" constant="false"/>

<speciesReference species="c_xmp" stoichiometry="1" constant="false"/>

</listOfReactants>

<listOfProducts>

<speciesReference species="c_pi" stoichiometry="1" constant="false"/>

<speciesReference species="c_xtsn" stoichiometry="1" constant="false"/>

</listOfProducts>

</reaction>

<reaction id="NTD11" name="NTD11" reversible="false" fast="false" compartment="cell">

<listOfReactants>

<speciesReference species="c_h2o" stoichiometry="1" constant="false"/>

<speciesReference species="c_imp" stoichiometry="1" constant="false"/>

</listOfReactants>

<listOfProducts>

<speciesReference species="c_ins" stoichiometry="1" constant="false"/>

<speciesReference species="c_pi" stoichiometry="1" constant="false"/>

</listOfProducts>

</reaction>

<reaction id="DHPS3" name="DHPS3" reversible="false" fast="false" compartment="cell">

<listOfReactants>

<speciesReference species="c_2ahhmd" stoichiometry="1" constant="false"/>

<speciesReference species="c_4abz" stoichiometry="1" constant="false"/>

</listOfReactants>

<listOfProducts>

<speciesReference species="c_dhpt" stoichiometry="1" constant="false"/>

<speciesReference species="c_ppi" stoichiometry="1" constant="false"/>

</listOfProducts>

</reaction>

<reaction id="P5CD" name="P5CD" reversible="false" fast="false" compartment="cell">

<listOfReactants>

<speciesReference species="c_1pyr5c" stoichiometry="1" constant="false"/>

<speciesReference species="c_h2o" stoichiometry="2" constant="false"/>

<speciesReference species="c_nad" stoichiometry="1" constant="false"/>

</listOfReactants>

<listOfProducts>

<speciesReference species="c_glu_L" stoichiometry="1" constant="false"/>

<speciesReference species="c_h" stoichiometry="1" constant="false"/>

<speciesReference species="c_nadh" stoichiometry="1" constant="false"/>

</listOfProducts>

</reaction>

<reaction id="UPP3MT" name="UPP3MT" reversible="false" fast="false" compartment="cell">

<listOfReactants>

<speciesReference species="c_amet" stoichiometry="2" constant="false"/>

<speciesReference species="c_uppg3" stoichiometry="1" constant="false"/>

</listOfReactants>

<listOfProducts>

<speciesReference species="c_ahcys" stoichiometry="2" constant="false"/>

<speciesReference species="c_h" stoichiometry="2" constant="false"/>

<speciesReference species="c_shcl" stoichiometry="1" constant="false"/>

</listOfProducts>

</reaction>

<reaction id="DHAD1" name="DHAD1" reversible="false" fast="false" compartment="cell">

<listOfReactants>

<speciesReference species="c_23dhmb" stoichiometry="1" constant="false"/>

</listOfReactants>

<listOfProducts>

<speciesReference species="c_3mob" stoichiometry="1" constant="false"/>

<speciesReference species="c_h2o" stoichiometry="1" constant="false"/>

</listOfProducts>

</reaction>

<reaction id="P5CR" name="P5CR" reversible="false" fast="false" compartment="cell">

<listOfReactants>

<speciesReference species="c_1pyr5c" stoichiometry="1" constant="false"/>

<speciesReference species="c_h" stoichiometry="2" constant="false"/>

<speciesReference species="c_nadph" stoichiometry="1" constant="false"/>

</listOfReactants>

<listOfProducts>

<speciesReference species="c_nadp" stoichiometry="1" constant="false"/>

<speciesReference species="c_pro_L" stoichiometry="1" constant="false"/>

</listOfProducts>

</reaction>

<reaction id="DHAD2" name="DHAD2" reversible="false" fast="false" compartment="cell">

<listOfReactants>

<speciesReference species="c_23dhmp" stoichiometry="1" constant="false"/>

</listOfReactants>

<listOfProducts>

<speciesReference species="c_3mop" stoichiometry="1" constant="false"/>

<speciesReference species="c_h2o" stoichiometry="1" constant="false"/>

</listOfProducts>

</reaction>

<reaction id="ATPM" name="ATPM" reversible="false" fast="false" compartment="cell">

<listOfReactants>

<speciesReference species="c_atp" stoichiometry="1" constant="false"/>

<speciesReference species="c_h2o" stoichiometry="1" constant="false"/>

</listOfReactants>

<listOfProducts>

<speciesReference species="c_adp" stoichiometry="1" constant="false"/>

<speciesReference species="c_h" stoichiometry="1" constant="false"/>

<speciesReference species="c_pi" stoichiometry="1" constant="false"/>

</listOfProducts>

</reaction>

<reaction id="TAL" name="TAL" reversible="true" fast="false" compartment="cell">

<listOfReactants>

<speciesReference species="c_g3p" stoichiometry="1" constant="false"/>

<speciesReference species="c_s7p" stoichiometry="1" constant="false"/>

</listOfReactants>

<listOfProducts>

<speciesReference species="c_e4p" stoichiometry="1" constant="false"/>

<speciesReference species="c_f6p" stoichiometry="1" constant="false"/>

</listOfProducts>

</reaction>

<reaction id="SPA" name="SPA" reversible="false" fast="false" compartment="cell">

<listOfReactants>

<speciesReference species="c_pyr" stoichiometry="1" constant="false"/>

<speciesReference species="c_ser_L" stoichiometry="1" constant="false"/>

</listOfReactants>

<listOfProducts>

<speciesReference species="c_ala_L" stoichiometry="1" constant="false"/>

<speciesReference species="c_hpyr" stoichiometry="1" constant="false"/>

</listOfProducts>

</reaction>

<reaction id="GLUTRR" name="GLUTRR" reversible="false" fast="false" compartment="cell">

<listOfReactants>

<speciesReference species="c_glutrna" stoichiometry="1" constant="false"/>

<speciesReference species="c_h" stoichiometry="1" constant="false"/>

<speciesReference species="c_nadph" stoichiometry="1" constant="false"/>

</listOfReactants>

<listOfProducts>

<speciesReference species="c_glu1sa" stoichiometry="1" constant="false"/>

<speciesReference species="c_nadp" stoichiometry="1" constant="false"/>

<speciesReference species="c_trnaglu" stoichiometry="1" constant="false"/>

</listOfProducts>

</reaction>

<reaction id="GLUTRS" name="GLUTRS" reversible="false" fast="false" compartment="cell">

<listOfReactants>

<speciesReference species="c_atp" stoichiometry="1" constant="false"/>

<speciesReference species="c_glu_L" stoichiometry="1" constant="false"/>

<speciesReference species="c_trnaglu" stoichiometry="1" constant="false"/>

</listOfReactants>

<listOfProducts>

<speciesReference species="c_amp" stoichiometry="1" constant="false"/>

<speciesReference species="c_glutrna" stoichiometry="1" constant="false"/>

<speciesReference species="c_ppi" stoichiometry="1" constant="false"/>

</listOfProducts>

</reaction>

<reaction id="UNK3" name="UNK3" reversible="false" fast="false" compartment="cell">

<listOfReactants>

<speciesReference species="c_2kmb" stoichiometry="1" constant="false"/>

<speciesReference species="c_glu_L" stoichiometry="1" constant="false"/>

</listOfReactants>

<listOfProducts>

<speciesReference species="c_akg" stoichiometry="1" constant="false"/>

<speciesReference species="c_met_L" stoichiometry="1" constant="false"/>

</listOfProducts>

</reaction>

<reaction id="XPK" name="XPK" reversible="false" fast="false" compartment="cell">

<listOfReactants>

<speciesReference species="c_pi" stoichiometry="1" constant="false"/>

<speciesReference species="c_xu5p_D" stoichiometry="1" constant="false"/>

</listOfReactants>

<listOfProducts>

<speciesReference species="c_actp" stoichiometry="1" constant="false"/>

<speciesReference species="c_g3p" stoichiometry="1" constant="false"/>

<speciesReference species="c_h2o" stoichiometry="1" constant="false"/>

</listOfProducts>

</reaction>

<reaction id="DASYN" name="DASYN" reversible="true" fast="false" compartment="cell">

<listOfReactants>

<speciesReference species="c_12dag3p" stoichiometry="1" constant="false"/>

<speciesReference species="c_ctp" stoichiometry="1" constant="false"/>

<speciesReference species="c_h" stoichiometry="1" constant="false"/>

</listOfReactants>

<listOfProducts>

<speciesReference species="c_cdpdag" stoichiometry="1" constant="false"/>

<speciesReference species="c_ppi" stoichiometry="1" constant="false"/>

</listOfProducts>

</reaction>

<reaction id="ACMAT1" name="ACMAT1" reversible="false" fast="false" compartment="cell">

<listOfReactants>

<speciesReference species="c_acACP" stoichiometry="1" constant="false"/>

<speciesReference species="c_h" stoichiometry="1" constant="false"/>

<speciesReference species="c_malACP" stoichiometry="1" constant="false"/>

</listOfReactants>

<listOfProducts>

<speciesReference species="c_ACP" stoichiometry="1" constant="false"/>

<speciesReference species="c_actACP" stoichiometry="1" constant="false"/>

<speciesReference species="c_co2" stoichiometry="1" constant="false"/>

</listOfProducts>

</reaction>

<reaction id="G1PACT" name="G1PACT" reversible="false" fast="false" compartment="cell">

<listOfReactants>

<speciesReference species="c_accoa" stoichiometry="1" constant="false"/>

<speciesReference species="c_gam1p" stoichiometry="1" constant="false"/>

</listOfReactants>

<listOfProducts>

<speciesReference species="c_acgam1p" stoichiometry="1" constant="false"/>

<speciesReference species="c_coa" stoichiometry="1" constant="false"/>

<speciesReference species="c_h" stoichiometry="1" constant="false"/>

</listOfProducts>

</reaction>

<reaction id="HMGSs" name="HMGSs" reversible="true" fast="false" compartment="cell">

<listOfReactants>

<speciesReference species="c_fald" stoichiometry="1" constant="false"/>

<speciesReference species="c_gthrd" stoichiometry="1" constant="false"/>

</listOfReactants>

<listOfProducts>

<speciesReference species="c_hmglut_S" stoichiometry="1" constant="false"/>

</listOfProducts>

</reaction>

<reaction id="KAS15" name="KAS15" reversible="false" fast="false" compartment="cell">

<listOfReactants>

<speciesReference species="c_accoa" stoichiometry="1" constant="false"/>

<speciesReference species="c_h" stoichiometry="1" constant="false"/>

<speciesReference species="c_malACP" stoichiometry="1" constant="false"/>

</listOfReactants>

<listOfProducts>

<speciesReference species="c_actACP" stoichiometry="1" constant="false"/>

<speciesReference species="c_co2" stoichiometry="1" constant="false"/>

<speciesReference species="c_coa" stoichiometry="1" constant="false"/>

</listOfProducts>

</reaction>

<reaction id="HBZOPT" name="HBZOPT" reversible="false" fast="false" compartment="cell">

<listOfReactants>

<speciesReference species="c_4hbz" stoichiometry="1" constant="false"/>

<speciesReference species="c_octdp" stoichiometry="1" constant="false"/>

</listOfReactants>

<listOfProducts>

<speciesReference species="c_3ophb" stoichiometry="1" constant="false"/>

<speciesReference species="c_ppi" stoichiometry="1" constant="false"/>

</listOfProducts>

</reaction>

<reaction id="KAS16" name="KAS16" reversible="false" fast="false" compartment="cell">

<listOfReactants>

<speciesReference species="c_ddcaACP" stoichiometry="1" constant="false"/>

<speciesReference species="c_h" stoichiometry="2" constant="false"/>

<speciesReference species="c_malACP" stoichiometry="1" constant="false"/>

<speciesReference species="c_nadph" stoichiometry="1" constant="false"/>

</listOfReactants>

<listOfProducts>

<speciesReference species="c_3htdACP" stoichiometry="1" constant="false"/>

<speciesReference species="c_ACP" stoichiometry="1" constant="false"/>

<speciesReference species="c_co2" stoichiometry="1" constant="false"/>

<speciesReference species="c_nadp" stoichiometry="1" constant="false"/>

</listOfProducts>

</reaction>

<reaction id="DHQD1" name="DHQD1" reversible="true" fast="false" compartment="cell">

<listOfReactants>

<speciesReference species="c_3dhq" stoichiometry="1" constant="false"/>

</listOfReactants>

<listOfProducts>

<speciesReference species="c_3dhsk" stoichiometry="1" constant="false"/>

<speciesReference species="c_h2o" stoichiometry="1" constant="false"/>

</listOfProducts>

</reaction>

<reaction id="C130ISN" name="C130ISN" reversible="false" fast="false" compartment="cell">

<listOfReactants>

<speciesReference species="c_fa7ACP" stoichiometry="1" constant="false"/>

<speciesReference species="c_h" stoichiometry="9" constant="false"/>

<speciesReference species="c_malACP" stoichiometry="3" constant="false"/>

<speciesReference species="c_nadph" stoichiometry="6" constant="false"/>

</listOfReactants>

<listOfProducts>

<speciesReference species="c_ACP" stoichiometry="3" constant="false"/>

<speciesReference species="c_co2" stoichiometry="3" constant="false"/>

<speciesReference species="c_fa13ACP" stoichiometry="1" constant="false"/>

<speciesReference species="c_h2o" stoichiometry="3" constant="false"/>

<speciesReference species="c_nadp" stoichiometry="6" constant="false"/>

</listOfProducts>

</reaction>

<reaction id="G5SADs" name="G5SADs" reversible="true" fast="false" compartment="cell">

<listOfReactants>

<speciesReference species="c_glu5sa" stoichiometry="1" constant="false"/>

</listOfReactants>

<listOfProducts>

<speciesReference species="c_1pyr5c" stoichiometry="1" constant="false"/>

<speciesReference species="c_h" stoichiometry="1" constant="false"/>

<speciesReference species="c_h2o" stoichiometry="1" constant="false"/>

</listOfProducts>

</reaction>

<reaction id="OMBZLM" name="OMBZLM" reversible="false" fast="false" compartment="cell">

<listOfReactants>

<speciesReference species="c_2ombzl" stoichiometry="1" constant="false"/>

<speciesReference species="c_amet" stoichiometry="1" constant="false"/>

</listOfReactants>

<listOfProducts>

<speciesReference species="c_2ommbl" stoichiometry="1" constant="false"/>

<speciesReference species="c_ahcys" stoichiometry="1" constant="false"/>

<speciesReference species="c_h" stoichiometry="1" constant="false"/>

</listOfProducts>

</reaction>

<reaction id="ACALDi" name="ACALDi" reversible="false" fast="false" compartment="cell">

<listOfReactants>

<speciesReference species="c_acald" stoichiometry="1" constant="false"/>

<speciesReference species="c_coa" stoichiometry="1" constant="false"/>

<speciesReference species="c_nad" stoichiometry="1" constant="false"/>

</listOfReactants>

<listOfProducts>

<speciesReference species="c_accoa" stoichiometry="1" constant="false"/>

<speciesReference species="c_h" stoichiometry="1" constant="false"/>

<speciesReference species="c_nadh" stoichiometry="1" constant="false"/>

</listOfProducts>

</reaction>

<reaction id="G1PTMT" name="G1PTMT" reversible="false" fast="false" compartment="cell">

<listOfReactants>

<speciesReference species="c_dttp" stoichiometry="1" constant="false"/>

<speciesReference species="c_g1p" stoichiometry="1" constant="false"/>

<speciesReference species="c_h" stoichiometry="1" constant="false"/>

</listOfReactants>

<listOfProducts>

<speciesReference species="c_dtdpglc" stoichiometry="1" constant="false"/>

<speciesReference species="c_ppi" stoichiometry="1" constant="false"/>

</listOfProducts>

</reaction>

<reaction id="NNDMBRT" name="NNDMBRT" reversible="false" fast="false" compartment="cell">

<listOfReactants>

<speciesReference species="c_dmbzid" stoichiometry="1" constant="false"/>

<speciesReference species="c_nicrnt" stoichiometry="1" constant="false"/>

</listOfReactants>

<listOfProducts>

<speciesReference species="c_5prdmbz" stoichiometry="1" constant="false"/>

<speciesReference species="c_h" stoichiometry="1" constant="false"/>

<speciesReference species="c_nac" stoichiometry="1" constant="false"/>

</listOfProducts>

</reaction>

<reaction id="OMPHHX" name="OMPHHX" reversible="false" fast="false" compartment="cell">

<listOfReactants>

<speciesReference species="c_2omph" stoichiometry="1" constant="false"/>

<speciesReference species="c_o2" stoichiometry="0.5" constant="false"/>

</listOfReactants>

<listOfProducts>

<speciesReference species="c_2ombzl" stoichiometry="1" constant="false"/>

</listOfProducts>

</reaction>

<reaction id="PANTS" name="PANTS" reversible="false" fast="false" compartment="cell">

<listOfReactants>

<speciesReference species="c_ala_B" stoichiometry="1" constant="false"/>

<speciesReference species="c_atp" stoichiometry="1" constant="false"/>

<speciesReference species="c_pant_R" stoichiometry="1" constant="false"/>

</listOfReactants>

<listOfProducts>

<speciesReference species="c_amp" stoichiometry="1" constant="false"/>

<speciesReference species="c_h" stoichiometry="1" constant="false"/>

<speciesReference species="c_pnto_R" stoichiometry="1" constant="false"/>

<speciesReference species="c_ppi" stoichiometry="1" constant="false"/>

</listOfProducts>

</reaction>

<reaction id="DMQMT" name="DMQMT" reversible="false" fast="false" compartment="cell">

<listOfReactants>

<speciesReference species="c_2omhmbl" stoichiometry="1" constant="false"/>

<speciesReference species="c_amet" stoichiometry="1" constant="false"/>

</listOfReactants>

<listOfProducts>

<speciesReference species="c_ahcys" stoichiometry="1" constant="false"/>

<speciesReference species="c_h" stoichiometry="1" constant="false"/>

<speciesReference species="c_ubq8h2" stoichiometry="1" constant="false"/>

</listOfProducts>

</reaction>

<reaction id="AACPS14" name="AACPS14" reversible="false" fast="false" compartment="cell">

<listOfReactants>

<speciesReference species="c_ACP" stoichiometry="1" constant="false"/>

<speciesReference species="c_atp" stoichiometry="1" constant="false"/>

<speciesReference species="c_pde" stoichiometry="1" constant="false"/>

</listOfReactants>

<listOfProducts>

<speciesReference species="c_amp" stoichiometry="1" constant="false"/>

<speciesReference species="c_pdeACP" stoichiometry="1" constant="false"/>

<speciesReference species="c_ppi" stoichiometry="1" constant="false"/>

</listOfProducts>

</reaction>

<reaction id="E4PD" name="E4PD" reversible="true" fast="false" compartment="cell">

<listOfReactants>

<speciesReference species="c_e4p" stoichiometry="1" constant="false"/>

<speciesReference species="c_h2o" stoichiometry="1" constant="false"/>

<speciesReference species="c_nad" stoichiometry="1" constant="false"/>

</listOfReactants>

<listOfProducts>

<speciesReference species="c_4per" stoichiometry="1" constant="false"/>

<speciesReference species="c_h" stoichiometry="2" constant="false"/>

<speciesReference species="c_nadh" stoichiometry="1" constant="false"/>

</listOfProducts>

</reaction>

<reaction id="AACPS12" name="AACPS12" reversible="false" fast="false" compartment="cell">

<listOfReactants>

<speciesReference species="c_ACP" stoichiometry="1" constant="false"/>

<speciesReference species="c_atp" stoichiometry="1" constant="false"/>

<speciesReference species="c_fa6" stoichiometry="1" constant="false"/>

</listOfReactants>

<listOfProducts>

<speciesReference species="c_amp" stoichiometry="1" constant="false"/>

<speciesReference species="c_fa6ACP" stoichiometry="1" constant="false"/>

<speciesReference species="c_ppi" stoichiometry="1" constant="false"/>

</listOfProducts>

</reaction>

<reaction id="AACPS13" name="AACPS13" reversible="false" fast="false" compartment="cell">

<listOfReactants>

<speciesReference species="c_ACP" stoichiometry="1" constant="false"/>

<speciesReference species="c_atp" stoichiometry="1" constant="false"/>

<speciesReference species="c_fa11" stoichiometry="1" constant="false"/>

</listOfReactants>

<listOfProducts>

<speciesReference species="c_amp" stoichiometry="1" constant="false"/>

<speciesReference species="c_fa11ACP" stoichiometry="1" constant="false"/>

<speciesReference species="c_ppi" stoichiometry="1" constant="false"/>

</listOfProducts>

</reaction>

<reaction id="AACPS10" name="AACPS10" reversible="false" fast="false" compartment="cell">

<listOfReactants>

<speciesReference species="c_ACP" stoichiometry="1" constant="false"/>

<speciesReference species="c_atp" stoichiometry="1" constant="false"/>

<speciesReference species="c_fa1" stoichiometry="1" constant="false"/>

</listOfReactants>

<listOfProducts>

<speciesReference species="c_amp" stoichiometry="1" constant="false"/>

<speciesReference species="c_fa1ACP" stoichiometry="1" constant="false"/>

<speciesReference species="c_ppi" stoichiometry="1" constant="false"/>

</listOfProducts>

</reaction>

<reaction id="AACPS11" name="AACPS11" reversible="false" fast="false" compartment="cell">

<listOfReactants>

<speciesReference species="c_ACP" stoichiometry="1" constant="false"/>

<speciesReference species="c_atp" stoichiometry="1" constant="false"/>

<speciesReference species="c_fa3" stoichiometry="1" constant="false"/>

</listOfReactants>

<listOfProducts>

<speciesReference species="c_amp" stoichiometry="1" constant="false"/>

<speciesReference species="c_fa3ACP" stoichiometry="1" constant="false"/>

<speciesReference species="c_ppi" stoichiometry="1" constant="false"/>

</listOfProducts>

</reaction>

<reaction id="GLCGSD" name="GLCGSD" reversible="false" fast="false" compartment="cell">

<listOfReactants>

<speciesReference species="c_glycogen" stoichiometry="1" constant="false"/>

<speciesReference species="c_h2o" stoichiometry="1" constant="false"/>

</listOfReactants>

<listOfProducts>

<speciesReference species="c_glc_D" stoichiometry="1" constant="false"/>

</listOfProducts>

</reaction>

<reaction id="PYAM5PO" name="PYAM5PO" reversible="false" fast="false" compartment="cell">

<listOfReactants>

<speciesReference species="c_h2o" stoichiometry="1" constant="false"/>

<speciesReference species="c_o2" stoichiometry="1" constant="false"/>

<speciesReference species="c_pyam5p" stoichiometry="1" constant="false"/>

</listOfReactants>

<listOfProducts>

<speciesReference species="c_h2o2" stoichiometry="1" constant="false"/>

<speciesReference species="c_nh4" stoichiometry="1" constant="false"/>

<speciesReference species="c_pydx5p" stoichiometry="1" constant="false"/>

</listOfProducts>

</reaction>

<reaction id="ACLS" name="ACLS" reversible="false" fast="false" compartment="cell">

<listOfReactants>

<speciesReference species="c_h" stoichiometry="1" constant="false"/>

<speciesReference species="c_pyr" stoichiometry="2" constant="false"/>

</listOfReactants>

<listOfProducts>

<speciesReference species="c_alac_S" stoichiometry="1" constant="false"/>

<speciesReference species="c_co2" stoichiometry="1" constant="false"/>

</listOfProducts>

</reaction>

<reaction id="C170SN" name="C170SN" reversible="false" fast="false" compartment="cell">

<listOfReactants>

<speciesReference species="c_fa14ACP" stoichiometry="1" constant="false"/>

<speciesReference species="c_h" stoichiometry="18" constant="false"/>

<speciesReference species="c_malACP" stoichiometry="6" constant="false"/>

<speciesReference species="c_nadph" stoichiometry="12" constant="false"/>

</listOfReactants>

<listOfProducts>

<speciesReference species="c_ACP" stoichiometry="6" constant="false"/>

<speciesReference species="c_co2" stoichiometry="6" constant="false"/>

<speciesReference species="c_h2o" stoichiometry="6" constant="false"/>

<speciesReference species="c_hpdACP" stoichiometry="1" constant="false"/>

<speciesReference species="c_nadp" stoichiometry="12" constant="false"/>

</listOfProducts>

</reaction>

<reaction id="NTPP8" name="NTPP8" reversible="false" fast="false" compartment="cell">

<listOfReactants>

<speciesReference species="c_h2o" stoichiometry="1" constant="false"/>

<speciesReference species="c_utp" stoichiometry="1" constant="false"/>

</listOfReactants>

<listOfProducts>

<speciesReference species="c_h" stoichiometry="1" constant="false"/>

<speciesReference species="c_ppi" stoichiometry="1" constant="false"/>

<speciesReference species="c_ump" stoichiometry="1" constant="false"/>

</listOfProducts>

</reaction>

<reaction id="NTPP7" name="NTPP7" reversible="false" fast="false" compartment="cell">

<listOfReactants>

<speciesReference species="c_dttp" stoichiometry="1" constant="false"/>

<speciesReference species="c_h2o" stoichiometry="1" constant="false"/>

</listOfReactants>

<listOfProducts>

<speciesReference species="c_dtmp" stoichiometry="1" constant="false"/>

<speciesReference species="c_h" stoichiometry="1" constant="false"/>

<speciesReference species="c_ppi" stoichiometry="1" constant="false"/>

</listOfProducts>

</reaction>

<reaction id="NTPP6" name="NTPP6" reversible="false" fast="false" compartment="cell">

<listOfReactants>

<speciesReference species="c_atp" stoichiometry="1" constant="false"/>

<speciesReference species="c_h2o" stoichiometry="1" constant="false"/>

</listOfReactants>

<listOfProducts>

<speciesReference species="c_amp" stoichiometry="1" constant="false"/>

<speciesReference species="c_h" stoichiometry="1" constant="false"/>

<speciesReference species="c_ppi" stoichiometry="1" constant="false"/>

</listOfProducts>

</reaction>

<reaction id="NTPP5" name="NTPP5" reversible="false" fast="false" compartment="cell">

<listOfReactants>

<speciesReference species="c_datp" stoichiometry="1" constant="false"/>

<speciesReference species="c_h2o" stoichiometry="1" constant="false"/>

</listOfReactants>

<listOfProducts>

<speciesReference species="c_damp" stoichiometry="1" constant="false"/>

<speciesReference species="c_h" stoichiometry="1" constant="false"/>

<speciesReference species="c_ppi" stoichiometry="1" constant="false"/>

</listOfProducts>

</reaction>

<reaction id="NTPP4" name="NTPP4" reversible="false" fast="false" compartment="cell">

<listOfReactants>

<speciesReference species="c_ctp" stoichiometry="1" constant="false"/>

<speciesReference species="c_h2o" stoichiometry="1" constant="false"/>

</listOfReactants>

<listOfProducts>

<speciesReference species="c_cmp" stoichiometry="1" constant="false"/>

<speciesReference species="c_h" stoichiometry="1" constant="false"/>

<speciesReference species="c_ppi" stoichiometry="1" constant="false"/>

</listOfProducts>

</reaction>

<reaction id="NTPP3" name="NTPP3" reversible="false" fast="false" compartment="cell">

<listOfReactants>

<speciesReference species="c_dctp" stoichiometry="1" constant="false"/>

<speciesReference species="c_h2o" stoichiometry="1" constant="false"/>

</listOfReactants>

<listOfProducts>

<speciesReference species="c_dcmp" stoichiometry="1" constant="false"/>

<speciesReference species="c_h" stoichiometry="1" constant="false"/>

<speciesReference species="c_ppi" stoichiometry="1" constant="false"/>

</listOfProducts>

</reaction>

<reaction id="C140ISN" name="C140ISN" reversible="false" fast="false" compartment="cell">

<listOfReactants>

<speciesReference species="c_fa8ACP" stoichiometry="1" constant="false"/>

<speciesReference species="c_h" stoichiometry="12" constant="false"/>

<speciesReference species="c_malACP" stoichiometry="4" constant="false"/>

<speciesReference species="c_nadph" stoichiometry="8" constant="false"/>

</listOfReactants>

<listOfProducts>

<speciesReference species="c_ACP" stoichiometry="4" constant="false"/>

<speciesReference species="c_co2" stoichiometry="4" constant="false"/>

<speciesReference species="c_fa1ACP" stoichiometry="1" constant="false"/>

<speciesReference species="c_h2o" stoichiometry="4" constant="false"/>

<speciesReference species="c_nadp" stoichiometry="8" constant="false"/>

</listOfProducts>

</reaction>

<reaction id="DHNAOT7" name="DHNAOT7" reversible="false" fast="false" compartment="cell">

<listOfReactants>

<speciesReference species="c_dhna" stoichiometry="1" constant="false"/>

<speciesReference species="c_hepdp" stoichiometry="1" constant="false"/>

<speciesReference species="c_nad" stoichiometry="1" constant="false"/>

</listOfReactants>

<listOfProducts>

<speciesReference species="c_2dmmq7" stoichiometry="1" constant="false"/>

<speciesReference species="c_co2" stoichiometry="1" constant="false"/>

<speciesReference species="c_nadh" stoichiometry="1" constant="false"/>

<speciesReference species="c_ppi" stoichiometry="1" constant="false"/>

</listOfProducts>

</reaction>

<reaction id="ACODA" name="ACODA" reversible="false" fast="false" compartment="cell">

<listOfReactants>

<speciesReference species="c_acorn" stoichiometry="1" constant="false"/>

<speciesReference species="c_h2o" stoichiometry="1" constant="false"/>

</listOfReactants>

<listOfProducts>

<speciesReference species="c_ac" stoichiometry="1" constant="false"/>

<speciesReference species="c_orn_L" stoichiometry="1" constant="false"/>

</listOfProducts>

</reaction>

<reaction id="LPSSYN_SO" name="LPSSYN_SO" reversible="false" fast="false" compartment="cell">

<listOfReactants>

<speciesReference stoichiometry="1" constant="false"/>

<speciesReference species="c_atp" stoichiometry="1" constant="false"/>

<speciesReference species="c_kdo8nlipa" stoichiometry="1" constant="false"/>

<speciesReference species="c_uacgala" stoichiometry="1" constant="false"/>

<speciesReference species="c_udpgal" stoichiometry="3" constant="false"/>

</listOfReactants>

<listOfProducts>

<speciesReference species="c_adp" stoichiometry="2" constant="false"/>

<speciesReference species="c_h" stoichiometry="6" constant="false"/>

<speciesReference species="c_lps_SO" stoichiometry="1" constant="false"/>

<speciesReference species="c_udp" stoichiometry="3" constant="false"/>

<speciesReference species="c_ump" stoichiometry="1" constant="false"/>

</listOfProducts>

</reaction>

<reaction id="PUNP7" name="PUNP7" reversible="true" fast="false" compartment="cell">

<listOfReactants>

<speciesReference species="c_pi" stoichiometry="1" constant="false"/>

<speciesReference species="c_xtsn" stoichiometry="1" constant="false"/>

</listOfReactants>

<listOfProducts>

<speciesReference species="c_r1p" stoichiometry="1" constant="false"/>

<speciesReference species="c_xan" stoichiometry="1" constant="false"/>

</listOfProducts>

</reaction>

<reaction id="RBZP" name="RBZP" reversible="false" fast="false" compartment="cell">

<listOfReactants>

<speciesReference species="c_5prdmbz" stoichiometry="1" constant="false"/>

<speciesReference species="c_h2o" stoichiometry="1" constant="false"/>

</listOfReactants>

<listOfProducts>

<speciesReference species="c_pi" stoichiometry="1" constant="false"/>

<speciesReference species="c_rdmbzi" stoichiometry="1" constant="false"/>

</listOfProducts>

</reaction>

<reaction name="4HBTE" reversible="true" fast="false" compartment="cell">

<listOfReactants>

<speciesReference species="c_4hbcoa" stoichiometry="1" constant="false"/>

<speciesReference species="c_h2o" stoichiometry="1" constant="false"/>

</listOfReactants>

<listOfProducts>

<speciesReference species="c_4hbz" stoichiometry="1" constant="false"/>

<speciesReference species="c_coa" stoichiometry="1" constant="false"/>

<speciesReference species="c_h" stoichiometry="1" constant="false"/>

</listOfProducts>

</reaction>

<reaction id="GLCS1" name="GLCS1" reversible="false" fast="false" compartment="cell">

<listOfReactants>

<speciesReference species="c_adpglc" stoichiometry="1" constant="false"/>

</listOfReactants>

<listOfProducts>

<speciesReference species="c_adp" stoichiometry="1" constant="false"/>

<speciesReference species="c_glycogen" stoichiometry="1" constant="false"/>

<speciesReference species="c_h" stoichiometry="1" constant="false"/>

</listOfProducts>

</reaction>

<reaction id="PUNP3" name="PUNP3" reversible="true" fast="false" compartment="cell">

<listOfReactants>

<speciesReference species="c_gsn" stoichiometry="1" constant="false"/>

<speciesReference species="c_pi" stoichiometry="1" constant="false"/>

</listOfReactants>

<listOfProducts>

<speciesReference species="c_gua" stoichiometry="1" constant="false"/>

<speciesReference species="c_r1p" stoichiometry="1" constant="false"/>

</listOfProducts>

</reaction>

<reaction id="GHMT" name="GHMT" reversible="true" fast="false" compartment="cell">

<listOfReactants>

<speciesReference species="c_ser_L" stoichiometry="1" constant="false"/>

<speciesReference species="c_thf" stoichiometry="1" constant="false"/>

</listOfReactants>

<listOfProducts>

<speciesReference species="c_gly" stoichiometry="1" constant="false"/>

<speciesReference species="c_h2o" stoichiometry="1" constant="false"/>

<speciesReference species="c_mlthf" stoichiometry="1" constant="false"/>

</listOfProducts>

</reaction>

<reaction id="ASNN" name="ASNN" reversible="false" fast="false" compartment="cell">

<listOfReactants>

<speciesReference species="c_asn_L" stoichiometry="1" constant="false"/>

<speciesReference species="c_h2o" stoichiometry="1" constant="false"/>

</listOfReactants>

<listOfProducts>

<speciesReference species="c_asp_L" stoichiometry="1" constant="false"/>

<speciesReference species="c_nh4" stoichiometry="1" constant="false"/>

</listOfProducts>

</reaction>

<reaction id="MCITS" name="MCITS" reversible="false" fast="false" compartment="cell">

<listOfReactants>

<speciesReference species="c_h2o" stoichiometry="1" constant="false"/>

<speciesReference species="c_oaa" stoichiometry="1" constant="false"/>

<speciesReference species="c_ppcoa" stoichiometry="1" constant="false"/>

</listOfReactants>

<listOfProducts>

<speciesReference species="c_2mcit" stoichiometry="1" constant="false"/>

<speciesReference species="c_coa" stoichiometry="1" constant="false"/>

<speciesReference species="c_h" stoichiometry="1" constant="false"/>

</listOfProducts>

</reaction>

<reaction id="APRAUR" name="APRAUR" reversible="false" fast="false" compartment="cell">

<listOfReactants>

<speciesReference species="c_5apru" stoichiometry="1" constant="false"/>

<speciesReference species="c_h" stoichiometry="1" constant="false"/>

<speciesReference species="c_nadph" stoichiometry="1" constant="false"/>

</listOfReactants>

<listOfProducts>

<speciesReference species="c_5aprbu" stoichiometry="1" constant="false"/>

<speciesReference species="c_nadp" stoichiometry="1" constant="false"/>

</listOfProducts>

</reaction>

<reaction id="MCITD" name="MCITD" reversible="false" fast="false" compartment="cell">

<listOfReactants>

<speciesReference species="c_2mcit" stoichiometry="1" constant="false"/>

</listOfReactants>

<listOfProducts>

<speciesReference species="c_2mcacn" stoichiometry="1" constant="false"/>

<speciesReference species="c_h2o" stoichiometry="1" constant="false"/>

</listOfProducts>

</reaction>

<reaction id="LDH_D2" name="LDH_D2" reversible="false" fast="false" compartment="cell">

<listOfReactants>

<speciesReference species="c_lac_D" stoichiometry="1" constant="false"/>

<speciesReference species="c_ubq8" stoichiometry="1" constant="false"/>

</listOfReactants>

<listOfProducts>

<speciesReference species="c_pyr" stoichiometry="1" constant="false"/>

<speciesReference species="c_ubq8h2" stoichiometry="1" constant="false"/>

</listOfProducts>

</reaction>

<reaction id="GTPDPK" name="GTPDPK" reversible="false" fast="false" compartment="cell">

<listOfReactants>

<speciesReference species="c_atp" stoichiometry="1" constant="false"/>

<speciesReference species="c_gtp" stoichiometry="1" constant="false"/>

</listOfReactants>

<listOfProducts>

<speciesReference species="c_amp" stoichiometry="1" constant="false"/>

<speciesReference species="c_gdptp" stoichiometry="1" constant="false"/>

<speciesReference species="c_h" stoichiometry="1" constant="false"/>

</listOfProducts>

</reaction>

<reaction id="IG3PS" name="IG3PS" reversible="false" fast="false" compartment="cell">

<listOfReactants>

<speciesReference species="c_gln_L" stoichiometry="1" constant="false"/>

<speciesReference species="c_prlp" stoichiometry="1" constant="false"/>

</listOfReactants>

<listOfProducts>

<speciesReference species="c_aicar" stoichiometry="1" constant="false"/>

<speciesReference species="c_eig3p" stoichiometry="1" constant="false"/>

<speciesReference species="c_glu_L" stoichiometry="1" constant="false"/>

<speciesReference species="c_h" stoichiometry="2" constant="false"/>

</listOfProducts>

</reaction>

<reaction id="AMMQT7_2" name="AMMQT7_2" reversible="false" fast="false" compartment="cell">

<listOfReactants>

<speciesReference species="c_2dmmq7" stoichiometry="1" constant="false"/>

<speciesReference species="c_amet" stoichiometry="1" constant="false"/>

</listOfReactants>

<listOfProducts>

<speciesReference species="c_ahcys" stoichiometry="1" constant="false"/>

<speciesReference species="c_h" stoichiometry="1" constant="false"/>

<speciesReference species="c_mqn7" stoichiometry="1" constant="false"/>

</listOfProducts>

</reaction>

<reaction id="AMMQT7_3" name="AMMQT7_3" reversible="false" fast="false" compartment="cell">

<listOfReactants>

<speciesReference species="c_amet" stoichiometry="1" constant="false"/>

<speciesReference species="c_mqn7" stoichiometry="1" constant="false"/>

</listOfReactants>

<listOfProducts>

<speciesReference species="c_ahcys" stoichiometry="1" constant="false"/>

<speciesReference species="c_h" stoichiometry="1" constant="false"/>

<speciesReference species="c_mmqn7" stoichiometry="1" constant="false"/>

</listOfProducts>

</reaction>

<reaction name="L-LACD4" reversible="false" fast="false" compartment="cell">

<listOfReactants>

<speciesReference species="c_lac_L" stoichiometry="1" constant="false"/>

<speciesReference species="c_mqn7" stoichiometry="1" constant="false"/>

</listOfReactants>

<listOfProducts>

<speciesReference species="c_mql7" stoichiometry="1" constant="false"/>

<speciesReference species="c_pyr" stoichiometry="1" constant="false"/>

</listOfProducts>

</reaction>

<reaction name="L-LACD5" reversible="false" fast="false" compartment="cell">

<listOfReactants>

<speciesReference species="c_lac_L" stoichiometry="1" constant="false"/>

<speciesReference species="c_mmqn7" stoichiometry="1" constant="false"/>

</listOfReactants>

<listOfProducts>

<speciesReference species="c_mmql7" stoichiometry="1" constant="false"/>

<speciesReference species="c_pyr" stoichiometry="1" constant="false"/>

</listOfProducts>

</reaction>

<reaction id="PPND" name="PPND" reversible="false" fast="false" compartment="cell">

<listOfReactants>

<speciesReference species="c_nad" stoichiometry="1" constant="false"/>

<speciesReference species="c_pphn" stoichiometry="1" constant="false"/>

</listOfReactants>

<listOfProducts>

<speciesReference species="c_34hpp" stoichiometry="1" constant="false"/>

<speciesReference species="c_co2" stoichiometry="1" constant="false"/>

<speciesReference species="c_nadh" stoichiometry="1" constant="false"/>

</listOfProducts>

</reaction>

<reaction id="ADSL2r" name="ADSL2r" reversible="true" fast="false" compartment="cell">

<listOfReactants>

<speciesReference species="c_25aics" stoichiometry="1" constant="false"/>

</listOfReactants>

<listOfProducts>

<speciesReference species="c_aicar" stoichiometry="1" constant="false"/>

<speciesReference species="c_fum" stoichiometry="1" constant="false"/>

</listOfProducts>

</reaction>

<reaction id="DNMPPA" name="DNMPPA" reversible="false" fast="false" compartment="cell">

<listOfReactants>

<speciesReference species="c_dhpmp" stoichiometry="1" constant="false"/>

<speciesReference species="c_h2o" stoichiometry="1" constant="false"/>

</listOfReactants>

<listOfProducts>

<speciesReference species="c_dhnpt" stoichiometry="1" constant="false"/>

<speciesReference species="c_pi" stoichiometry="1" constant="false"/>

</listOfProducts>

</reaction>

<reaction id="G1SATi" name="G1SATi" reversible="false" fast="false" compartment="cell">

<listOfReactants>

<speciesReference species="c_glu1sa" stoichiometry="1" constant="false"/>

</listOfReactants>

<listOfProducts>

<speciesReference species="c_5aop" stoichiometry="1" constant="false"/>

</listOfProducts>

</reaction>

<reaction id="GMHEPK" name="GMHEPK" reversible="false" fast="false" compartment="cell">

<listOfReactants>

<speciesReference species="c_atp" stoichiometry="1" constant="false"/>

<speciesReference species="c_gmh7p" stoichiometry="1" constant="false"/>

</listOfReactants>

<listOfProducts>

<speciesReference species="c_adp" stoichiometry="1" constant="false"/>

<speciesReference species="c_gmh17bp" stoichiometry="1" constant="false"/>

<speciesReference species="c_h" stoichiometry="1" constant="false"/>

</listOfProducts>

</reaction>

<reaction id="UAGPT3" name="UAGPT3" reversible="false" fast="false" compartment="cell">

<listOfReactants>

<speciesReference species="c_uacgam" stoichiometry="1" constant="false"/>

<speciesReference species="c_uagmda" stoichiometry="1" constant="false"/>

</listOfReactants>

<listOfProducts>

<speciesReference species="c_h" stoichiometry="1" constant="false"/>

<speciesReference species="c_uaagmda" stoichiometry="1" constant="false"/>

<speciesReference species="c_udp" stoichiometry="1" constant="false"/>

</listOfProducts>

</reaction>

<reaction id="ACHBS" name="ACHBS" reversible="false" fast="false" compartment="cell">

<listOfReactants>

<speciesReference species="c_2obut" stoichiometry="1" constant="false"/>

<speciesReference species="c_h" stoichiometry="1" constant="false"/>

<speciesReference species="c_pyr" stoichiometry="1" constant="false"/>

</listOfReactants>

<listOfProducts>

<speciesReference species="c_2ahbut" stoichiometry="1" constant="false"/>

<speciesReference species="c_co2" stoichiometry="1" constant="false"/>

</listOfProducts>

</reaction>

<reaction id="TYRTRS" name="TYRTRS" reversible="false" fast="false" compartment="cell">

<listOfReactants>

<speciesReference species="c_atp" stoichiometry="1" constant="false"/>

<speciesReference species="c_trnatyr" stoichiometry="1" constant="false"/>

<speciesReference species="c_tyr_L" stoichiometry="1" constant="false"/>

</listOfReactants>

<listOfProducts>

<speciesReference species="c_amp" stoichiometry="1" constant="false"/>

<speciesReference species="c_ppi" stoichiometry="1" constant="false"/>

<speciesReference species="c_tyrtrna" stoichiometry="1" constant="false"/>

</listOfProducts>

</reaction>

<reaction id="GART" name="GART" reversible="false" fast="false" compartment="cell">

<listOfReactants>

<speciesReference species="c_atp" stoichiometry="1" constant="false"/>

<speciesReference species="c_for" stoichiometry="1" constant="false"/>

<speciesReference species="c_gar" stoichiometry="1" constant="false"/>

</listOfReactants>

<listOfProducts>

<speciesReference species="c_adp" stoichiometry="1" constant="false"/>

<speciesReference species="c_fgam" stoichiometry="1" constant="false"/>

<speciesReference species="c_h" stoichiometry="1" constant="false"/>

<speciesReference species="c_pi" stoichiometry="1" constant="false"/>

</listOfProducts>

</reaction>

<reaction id="DRPA" name="DRPA" reversible="false" fast="false" compartment="cell">

<listOfReactants>

<speciesReference species="c_2dr5p" stoichiometry="1" constant="false"/>

</listOfReactants>

<listOfProducts>

<speciesReference species="c_acald" stoichiometry="1" constant="false"/>

<speciesReference species="c_g3p" stoichiometry="1" constant="false"/>

</listOfProducts>

</reaction>

<reaction id="AMAOT" name="AMAOT" reversible="true" fast="false" compartment="cell">

<listOfReactants>

<speciesReference species="c_8aonn" stoichiometry="1" constant="false"/>

<speciesReference species="c_amet" stoichiometry="1" constant="false"/>

</listOfReactants>

<listOfProducts>

<speciesReference species="c_amob" stoichiometry="1" constant="false"/>

<speciesReference species="c_dann" stoichiometry="1" constant="false"/>

</listOfProducts>

</reaction>

<reaction id="LEUTA" name="LEUTA" reversible="true" fast="false" compartment="cell">

<listOfReactants>

<speciesReference species="c_akg" stoichiometry="1" constant="false"/>

<speciesReference species="c_leu_L" stoichiometry="1" constant="false"/>

</listOfReactants>

<listOfProducts>

<speciesReference species="c_4mop" stoichiometry="1" constant="false"/>

<speciesReference species="c_glu_L" stoichiometry="1" constant="false"/>

</listOfProducts>

</reaction>

<reaction id="CYTDK3" name="CYTDK3" reversible="false" fast="false" compartment="cell">

<listOfReactants>

<speciesReference species="c_cytd" stoichiometry="1" constant="false"/>

<speciesReference species="c_itp" stoichiometry="1" constant="false"/>

</listOfReactants>

<listOfProducts>

<speciesReference species="c_cmp" stoichiometry="1" constant="false"/>

<speciesReference species="c_h" stoichiometry="1" constant="false"/>

<speciesReference species="c_idp" stoichiometry="1" constant="false"/>

</listOfProducts>

</reaction>

<reaction id="CYTDK2" name="CYTDK2" reversible="false" fast="false" compartment="cell">

<listOfReactants>

<speciesReference species="c_cytd" stoichiometry="1" constant="false"/>

<speciesReference species="c_gtp" stoichiometry="1" constant="false"/>

</listOfReactants>

<listOfProducts>

<speciesReference species="c_cmp" stoichiometry="1" constant="false"/>

<speciesReference species="c_gdp" stoichiometry="1" constant="false"/>

<speciesReference species="c_h" stoichiometry="1" constant="false"/>

</listOfProducts>

</reaction>

<reaction id="CYTDK1" name="CYTDK1" reversible="false" fast="false" compartment="cell">

<listOfReactants>

<speciesReference species="c_atp" stoichiometry="1" constant="false"/>

<speciesReference species="c_cytd" stoichiometry="1" constant="false"/>

</listOfReactants>

<listOfProducts>

<speciesReference species="c_adp" stoichiometry="1" constant="false"/>

<speciesReference species="c_cmp" stoichiometry="1" constant="false"/>

<speciesReference species="c_h" stoichiometry="1" constant="false"/>

</listOfProducts>

</reaction>

<reaction id="ALATRS" name="ALATRS" reversible="false" fast="false" compartment="cell">

<listOfReactants>

<speciesReference species="c_ala_L" stoichiometry="1" constant="false"/>

<speciesReference species="c_atp" stoichiometry="1" constant="false"/>

<speciesReference species="c_trnaala" stoichiometry="1" constant="false"/>

</listOfReactants>

<listOfProducts>

<speciesReference species="c_alatrna" stoichiometry="1" constant="false"/>

<speciesReference species="c_amp" stoichiometry="1" constant="false"/>

<speciesReference species="c_ppi" stoichiometry="1" constant="false"/>

</listOfProducts>

</reaction>

<reaction id="C140SN" name="C140SN" reversible="false" fast="false" compartment="cell">

<listOfReactants>

<speciesReference species="c_actACP" stoichiometry="1" constant="false"/>

<speciesReference species="c_h" stoichiometry="17" constant="false"/>

<speciesReference species="c_malACP" stoichiometry="5" constant="false"/>

<speciesReference species="c_nadph" stoichiometry="12" constant="false"/>

</listOfReactants>

<listOfProducts>

<speciesReference species="c_ACP" stoichiometry="5" constant="false"/>

<speciesReference species="c_co2" stoichiometry="5" constant="false"/>

<speciesReference species="c_h2o" stoichiometry="6" constant="false"/>

<speciesReference species="c_myrsACP" stoichiometry="1" constant="false"/>

<speciesReference species="c_nadp" stoichiometry="12" constant="false"/>

</listOfProducts>

</reaction>

<reaction id="C150SN" name="C150SN" reversible="false" fast="false" compartment="cell">

<listOfReactants>

<speciesReference species="c_fa14ACP" stoichiometry="1" constant="false"/>

<speciesReference species="c_h" stoichiometry="15" constant="false"/>

<speciesReference species="c_malACP" stoichiometry="5" constant="false"/>

<speciesReference species="c_nadph" stoichiometry="10" constant="false"/>

</listOfReactants>

<listOfProducts>

<speciesReference species="c_ACP" stoichiometry="5" constant="false"/>

<speciesReference species="c_co2" stoichiometry="5" constant="false"/>

<speciesReference species="c_h2o" stoichiometry="5" constant="false"/>

<speciesReference species="c_nadp" stoichiometry="10" constant="false"/>

<speciesReference species="c_pdACP" stoichiometry="1" constant="false"/>

</listOfProducts>

</reaction>

<reaction id="ABTA" name="ABTA" reversible="false" fast="false" compartment="cell">

<listOfReactants>

<speciesReference species="c_4abut" stoichiometry="1" constant="false"/>

<speciesReference species="c_akg" stoichiometry="1" constant="false"/>

</listOfReactants>

<listOfProducts>

<speciesReference species="c_glu_L" stoichiometry="1" constant="false"/>

<speciesReference species="c_sucsal" stoichiometry="1" constant="false"/>

</listOfProducts>

</reaction>

<reaction id="HTHBPD" name="HTHBPD" reversible="false" fast="false" compartment="cell">

<listOfReactants>

<speciesReference species="c_5678thh" stoichiometry="1" constant="false"/>

</listOfReactants>

<listOfProducts>

<speciesReference species="c_78dhp" stoichiometry="1" constant="false"/>

<speciesReference species="c_h2o" stoichiometry="1" constant="false"/>

</listOfProducts>

</reaction>

<reaction id="FGLU" name="FGLU" reversible="false" fast="false" compartment="cell">

<listOfReactants>

<speciesReference species="c_forglu" stoichiometry="1" constant="false"/>

<speciesReference species="c_h2o" stoichiometry="1" constant="false"/>

</listOfReactants>

<listOfProducts>

<speciesReference species="c_frmd" stoichiometry="1" constant="false"/>

<speciesReference species="c_glu_L" stoichiometry="1" constant="false"/>

</listOfProducts>

</reaction>

<reaction id="PMANM" name="PMANM" reversible="true" fast="false" compartment="cell">

<listOfReactants>

<speciesReference species="c_man1p" stoichiometry="1" constant="false"/>

</listOfReactants>

<listOfProducts>

<speciesReference species="c_man6p" stoichiometry="1" constant="false"/>

</listOfProducts>

</reaction>

<reaction id="DXPRI" name="DXPRI" reversible="true" fast="false" compartment="cell">

<listOfReactants>

<speciesReference species="c_dxyl5p" stoichiometry="1" constant="false"/>

<speciesReference species="c_h" stoichiometry="1" constant="false"/>

<speciesReference species="c_nadph" stoichiometry="1" constant="false"/>

</listOfReactants>

<listOfProducts>

<speciesReference species="c_2me4p" stoichiometry="1" constant="false"/>

<speciesReference species="c_nadp" stoichiometry="1" constant="false"/>

</listOfProducts>

</reaction>

<reaction id="GSHPO" name="GSHPO" reversible="false" fast="false" compartment="cell">

<listOfReactants>

<speciesReference species="c_gthrd" stoichiometry="2" constant="false"/>

<speciesReference species="c_h2o2" stoichiometry="1" constant="false"/>

</listOfReactants>

<listOfProducts>

<speciesReference species="c_gthox" stoichiometry="1" constant="false"/>

<speciesReference species="c_h2o" stoichiometry="2" constant="false"/>

</listOfProducts>

</reaction>

<reaction id="DMATT" name="DMATT" reversible="false" fast="false" compartment="cell">

<listOfReactants>

<speciesReference species="c_dmpp" stoichiometry="1" constant="false"/>

<speciesReference species="c_ipdp" stoichiometry="1" constant="false"/>

</listOfReactants>

<listOfProducts>

<speciesReference species="c_grdp" stoichiometry="1" constant="false"/>

<speciesReference species="c_ppi" stoichiometry="1" constant="false"/>

</listOfProducts>

</reaction>

<reaction id="LLEUDr" name="LLEUDr" reversible="true" fast="false" compartment="cell">

<listOfReactants>

<speciesReference species="c_h2o" stoichiometry="1" constant="false"/>

<speciesReference species="c_leu_L" stoichiometry="1" constant="false"/>

<speciesReference species="c_nad" stoichiometry="1" constant="false"/>

</listOfReactants>

<listOfProducts>

<speciesReference species="c_4mop" stoichiometry="1" constant="false"/>

<speciesReference species="c_h" stoichiometry="1" constant="false"/>

<speciesReference species="c_nadh" stoichiometry="1" constant="false"/>

<speciesReference species="c_nh4" stoichiometry="1" constant="false"/>

</listOfProducts>

</reaction>

<reaction id="GSNK" name="GSNK" reversible="false" fast="false" compartment="cell">

<listOfReactants>

<speciesReference species="c_atp" stoichiometry="1" constant="false"/>

<speciesReference species="c_gsn" stoichiometry="1" constant="false"/>

</listOfReactants>

<listOfProducts>

<speciesReference species="c_adp" stoichiometry="1" constant="false"/>

<speciesReference species="c_gmp" stoichiometry="1" constant="false"/>

<speciesReference species="c_h" stoichiometry="1" constant="false"/>

</listOfProducts>

</reaction>

<reaction id="SDPDS" name="SDPDS" reversible="false" fast="false" compartment="cell">

<listOfReactants>

<speciesReference species="c_h2o" stoichiometry="1" constant="false"/>

<speciesReference species="c_sl26da" stoichiometry="1" constant="false"/>

</listOfReactants>

<listOfProducts>

<speciesReference species="c_26dap_LL" stoichiometry="1" constant="false"/>

<speciesReference species="c_succ" stoichiometry="1" constant="false"/>

</listOfProducts>

</reaction>

<reaction id="HMBS" name="HMBS" reversible="false" fast="false" compartment="cell">

<listOfReactants>

<speciesReference species="c_h2o" stoichiometry="1" constant="false"/>

<speciesReference species="c_ppbng" stoichiometry="4" constant="false"/>

</listOfReactants>

<listOfProducts>

<speciesReference species="c_hmbil" stoichiometry="1" constant="false"/>

<speciesReference species="c_nh4" stoichiometry="4" constant="false"/>

</listOfProducts>

</reaction>

<reaction id="GTPCI" name="GTPCI" reversible="false" fast="false" compartment="cell">

<listOfReactants>

<speciesReference species="c_gtp" stoichiometry="1" constant="false"/>

<speciesReference species="c_h2o" stoichiometry="1" constant="false"/>

</listOfReactants>

<listOfProducts>

<speciesReference species="c_ahdt" stoichiometry="1" constant="false"/>

<speciesReference species="c_for" stoichiometry="1" constant="false"/>

<speciesReference species="c_h" stoichiometry="1" constant="false"/>

</listOfProducts>

</reaction>

<reaction id="PPBNGS" name="PPBNGS" reversible="false" fast="false" compartment="cell">

<listOfReactants>

<speciesReference species="c_5aop" stoichiometry="2" constant="false"/>

</listOfReactants>

<listOfProducts>

<speciesReference species="c_h" stoichiometry="1" constant="false"/>

<speciesReference species="c_h2o" stoichiometry="2" constant="false"/>

<speciesReference species="c_ppbng" stoichiometry="1" constant="false"/>

</listOfProducts>

</reaction>

<reaction id="C160ISN" name="C160ISN" reversible="false" fast="false" compartment="cell">

<listOfReactants>

<speciesReference species="c_fa8ACP" stoichiometry="1" constant="false"/>

<speciesReference species="c_h" stoichiometry="15" constant="false"/>

<speciesReference species="c_malACP" stoichiometry="5" constant="false"/>

<speciesReference species="c_nadph" stoichiometry="10" constant="false"/>

</listOfReactants>

<listOfProducts>

<speciesReference species="c_ACP" stoichiometry="5" constant="false"/>

<speciesReference species="c_co2" stoichiometry="5" constant="false"/>

<speciesReference species="c_fa6ACP" stoichiometry="1" constant="false"/>

<speciesReference species="c_h2o" stoichiometry="5" constant="false"/>

<speciesReference species="c_nadp" stoichiometry="10" constant="false"/>

</listOfProducts>

</reaction>

<reaction id="FCLT" name="FCLT" reversible="false" fast="false" compartment="cell">

<listOfReactants>

<speciesReference species="c_fe2" stoichiometry="1" constant="false"/>

<speciesReference species="c_ppp9" stoichiometry="1" constant="false"/>

</listOfReactants>

<listOfProducts>

<speciesReference species="c_h" stoichiometry="1" constant="false"/>

<speciesReference species="c_pheme" stoichiometry="1" constant="false"/>

</listOfProducts>

</reaction>

<reaction id="IPPMIb" name="IPPMIb" reversible="true" fast="false" compartment="cell">

<listOfReactants>

<speciesReference species="c_2ippm" stoichiometry="1" constant="false"/>

<speciesReference species="c_h2o" stoichiometry="1" constant="false"/>

</listOfReactants>

<listOfProducts>

<speciesReference species="c_3c3hmp" stoichiometry="1" constant="false"/>

</listOfProducts>

</reaction>

<reaction id="PTAr" name="PTAr" reversible="true" fast="false" compartment="cell">

<listOfReactants>

<speciesReference species="c_accoa" stoichiometry="1" constant="false"/>

<speciesReference species="c_pi" stoichiometry="1" constant="false"/>

</listOfReactants>

<listOfProducts>

<speciesReference species="c_actp" stoichiometry="1" constant="false"/>

<speciesReference species="c_coa" stoichiometry="1" constant="false"/>

</listOfProducts>

</reaction>

<reaction id="URIDK3" name="URIDK3" reversible="true" fast="false" compartment="cell">

<listOfReactants>

<speciesReference species="c_atp" stoichiometry="1" constant="false"/>

<speciesReference species="c_dump" stoichiometry="1" constant="false"/>

</listOfReactants>

<listOfProducts>

<speciesReference species="c_adp" stoichiometry="1" constant="false"/>

<speciesReference species="c_dudp" stoichiometry="1" constant="false"/>

</listOfProducts>

</reaction>

<reaction id="URIDK2" name="URIDK2" reversible="false" fast="false" compartment="cell">

<listOfReactants>

<speciesReference species="c_atp" stoichiometry="1" constant="false"/>

<speciesReference species="c_dump" stoichiometry="1" constant="false"/>

</listOfReactants>

<listOfProducts>

<speciesReference species="c_adp" stoichiometry="1" constant="false"/>

<speciesReference species="c_dudp" stoichiometry="1" constant="false"/>

</listOfProducts>

</reaction>

<reaction id="CLAT" name="CLAT" reversible="true" fast="false" compartment="cell">

<listOfReactants>

<speciesReference species="c_accoa" stoichiometry="1" constant="false"/>

<speciesReference species="c_cm" stoichiometry="1" constant="false"/>

</listOfReactants>

<listOfProducts>

<speciesReference species="c_cmac" stoichiometry="1" constant="false"/>

<speciesReference species="c_coa" stoichiometry="1" constant="false"/>

</listOfProducts>

</reaction>

<reaction id="HISTRS" name="HISTRS" reversible="false" fast="false" compartment="cell">

<listOfReactants>

<speciesReference species="c_atp" stoichiometry="1" constant="false"/>

<speciesReference species="c_his_L" stoichiometry="1" constant="false"/>

<speciesReference species="c_trnahis" stoichiometry="1" constant="false"/>

</listOfReactants>

<listOfProducts>

<speciesReference species="c_amp" stoichiometry="1" constant="false"/>

<speciesReference species="c_histrna" stoichiometry="1" constant="false"/>

<speciesReference species="c_ppi" stoichiometry="1" constant="false"/>

</listOfProducts>

</reaction>

<reaction id="CBPS" name="CBPS" reversible="false" fast="false" compartment="cell">

<listOfReactants>

<speciesReference species="c_atp" stoichiometry="2" constant="false"/>

<speciesReference species="c_gln_L" stoichiometry="1" constant="false"/>

<speciesReference species="c_h2o" stoichiometry="1" constant="false"/>

<speciesReference species="c_hco3" stoichiometry="1" constant="false"/>

</listOfReactants>

<listOfProducts>

<speciesReference species="c_adp" stoichiometry="2" constant="false"/>

<speciesReference species="c_cbp" stoichiometry="1" constant="false"/>

<speciesReference species="c_glu_L" stoichiometry="1" constant="false"/>

<speciesReference species="c_h" stoichiometry="2" constant="false"/>

<speciesReference species="c_pi" stoichiometry="1" constant="false"/>

</listOfProducts>

</reaction>

<reaction id="MOHMT" name="MOHMT" reversible="false" fast="false" compartment="cell">

<listOfReactants>

<speciesReference species="c_3mob" stoichiometry="1" constant="false"/>

<speciesReference species="c_h2o" stoichiometry="1" constant="false"/>

<speciesReference species="c_mlthf" stoichiometry="1" constant="false"/>

</listOfReactants>

<listOfProducts>

<speciesReference species="c_2dhp" stoichiometry="1" constant="false"/>

<speciesReference species="c_thf" stoichiometry="1" constant="false"/>

</listOfProducts>

</reaction>

<reaction id="SADT2" name="SADT2" reversible="false" fast="false" compartment="cell">

<listOfReactants>

<speciesReference species="c_atp" stoichiometry="1" constant="false"/>

<speciesReference species="c_gtp" stoichiometry="1" constant="false"/>

<speciesReference species="c_h2o" stoichiometry="1" constant="false"/>

<speciesReference species="c_so4" stoichiometry="1" constant="false"/>

</listOfReactants>

<listOfProducts>

<speciesReference species="c_aps" stoichiometry="1" constant="false"/>

<speciesReference species="c_gdp" stoichiometry="1" constant="false"/>

<speciesReference species="c_pi" stoichiometry="1" constant="false"/>

<speciesReference species="c_ppi" stoichiometry="1" constant="false"/>

</listOfProducts>

</reaction>

<reaction id="DHDPS" name="DHDPS" reversible="false" fast="false" compartment="cell">

<listOfReactants>

<speciesReference species="c_aspsa" stoichiometry="1" constant="false"/>

<speciesReference species="c_pyr" stoichiometry="1" constant="false"/>

</listOfReactants>

<listOfProducts>

<speciesReference species="c_23dhdp" stoichiometry="1" constant="false"/>

<speciesReference species="c_h" stoichiometry="1" constant="false"/>

<speciesReference species="c_h2o" stoichiometry="2" constant="false"/>

</listOfProducts>

</reaction>

<reaction id="C160SN" name="C160SN" reversible="false" fast="false" compartment="cell">

<listOfReactants>

<speciesReference species="c_actACP" stoichiometry="1" constant="false"/>

<speciesReference species="c_h" stoichiometry="20" constant="false"/>

<speciesReference species="c_malACP" stoichiometry="6" constant="false"/>

<speciesReference species="c_nadph" stoichiometry="14" constant="false"/>

</listOfReactants>

<listOfProducts>

<speciesReference species="c_ACP" stoichiometry="6" constant="false"/>

<speciesReference species="c_co2" stoichiometry="6" constant="false"/>

<speciesReference species="c_h2o" stoichiometry="7" constant="false"/>

<speciesReference species="c_nadp" stoichiometry="14" constant="false"/>

<speciesReference species="c_palmACP" stoichiometry="1" constant="false"/>

</listOfProducts>

</reaction>

<reaction id="EPPP2" name="EPPP2" reversible="true" fast="false" compartment="cell">

<listOfReactants>

<speciesReference species="c_gdptp" stoichiometry="1" constant="false"/>

<speciesReference species="c_h2o" stoichiometry="1" constant="false"/>

</listOfReactants>

<listOfProducts>

<speciesReference species="c_gdpdp" stoichiometry="1" constant="false"/>

<speciesReference species="c_h" stoichiometry="1" constant="false"/>

<speciesReference species="c_pi" stoichiometry="1" constant="false"/>

</listOfProducts>

</reaction>

<reaction id="UDCPDP" name="UDCPDP" reversible="false" fast="false" compartment="cell">

<listOfReactants>

<speciesReference species="c_h2o" stoichiometry="1" constant="false"/>

<speciesReference species="c_udcpdp" stoichiometry="1" constant="false"/>

</listOfReactants>

<listOfProducts>

<speciesReference species="c_h" stoichiometry="1" constant="false"/>

<speciesReference species="c_pi" stoichiometry="1" constant="false"/>

<speciesReference species="c_udcpp" stoichiometry="1" constant="false"/>

</listOfProducts>

</reaction>

<reaction id="GLUCYSL" name="GLUCYSL" reversible="false" fast="false" compartment="cell">

<listOfReactants>

<speciesReference species="c_atp" stoichiometry="1" constant="false"/>

<speciesReference species="c_cys_L" stoichiometry="1" constant="false"/>

<speciesReference species="c_glu_L" stoichiometry="1" constant="false"/>

</listOfReactants>

<listOfProducts>

<speciesReference species="c_adp" stoichiometry="1" constant="false"/>

<speciesReference species="c_glucys" stoichiometry="1" constant="false"/>

<speciesReference species="c_h" stoichiometry="1" constant="false"/>

<speciesReference species="c_pi" stoichiometry="1" constant="false"/>

</listOfProducts>

</reaction>

<reaction id="ALATA_D2" name="ALATA_D2" reversible="false" fast="false" compartment="cell">

<listOfReactants>

<speciesReference species="c_ala_D" stoichiometry="1" constant="false"/>

<speciesReference species="c_pydx5p" stoichiometry="1" constant="false"/>

</listOfReactants>

<listOfProducts>

<speciesReference species="c_pyam5p" stoichiometry="1" constant="false"/>

<speciesReference species="c_pyr" stoichiometry="1" constant="false"/>

</listOfProducts>

</reaction>

<reaction id="AKGD" name="AKGD" reversible="false" fast="false" compartment="cell">

<listOfReactants>

<speciesReference species="c_akg" stoichiometry="1" constant="false"/>

<speciesReference species="c_coa" stoichiometry="1" constant="false"/>

<speciesReference species="c_nad" stoichiometry="1" constant="false"/>

</listOfReactants>

<listOfProducts>

<speciesReference species="c_co2" stoichiometry="1" constant="false"/>

<speciesReference species="c_nadh" stoichiometry="1" constant="false"/>

<speciesReference species="c_succoa" stoichiometry="1" constant="false"/>

</listOfProducts>

</reaction>

<reaction id="CPPPGOAN" name="CPPPGOAN" reversible="false" fast="false" compartment="cell">

<listOfReactants>

<speciesReference species="c_amet" stoichiometry="2" constant="false"/>

<speciesReference species="c_cpppg3" stoichiometry="1" constant="false"/>

<speciesReference species="c_fdxo_4_2" stoichiometry="2" constant="false"/>

<speciesReference species="c_nadph" stoichiometry="2" constant="false"/>

</listOfReactants>

<listOfProducts>

<speciesReference species="c_co2" stoichiometry="2" constant="false"/>

<speciesReference species="c_dad_5" stoichiometry="2" constant="false"/>

<speciesReference species="c_fdxr_4_2" stoichiometry="2" constant="false"/>

<speciesReference species="c_h" stoichiometry="2" constant="false"/>

<speciesReference species="c_met_L" stoichiometry="2" constant="false"/>

<speciesReference species="c_nadp" stoichiometry="2" constant="false"/>

<speciesReference species="c_pppg9" stoichiometry="1" constant="false"/>

</listOfProducts>

</reaction>

<reaction id="ADPT" name="ADPT" reversible="false" fast="false" compartment="cell">

<listOfReactants>

<speciesReference species="c_ade" stoichiometry="1" constant="false"/>

<speciesReference species="c_prpp" stoichiometry="1" constant="false"/>

</listOfReactants>

<listOfProducts>

<speciesReference species="c_amp" stoichiometry="1" constant="false"/>

<speciesReference species="c_ppi" stoichiometry="1" constant="false"/>

</listOfProducts>

</reaction>

<reaction id="C170ISN" name="C170ISN" reversible="false" fast="false" compartment="cell">

<listOfReactants>

<speciesReference species="c_fa7ACP" stoichiometry="1" constant="false"/>

<speciesReference species="c_h" stoichiometry="15" constant="false"/>

<speciesReference species="c_malACP" stoichiometry="5" constant="false"/>

<speciesReference species="c_nadph" stoichiometry="10" constant="false"/>

</listOfReactants>

<listOfProducts>

<speciesReference species="c_ACP" stoichiometry="5" constant="false"/>

<speciesReference species="c_co2" stoichiometry="5" constant="false"/>

<speciesReference species="c_fa11ACP" stoichiometry="1" constant="false"/>

<speciesReference species="c_h2o" stoichiometry="5" constant="false"/>

<speciesReference species="c_nadp" stoichiometry="10" constant="false"/>

</listOfProducts>

</reaction>

<reaction id="EDA" name="EDA" reversible="false" fast="false" compartment="cell">

<listOfReactants>

<speciesReference species="c_2ddg6p" stoichiometry="1" constant="false"/>

</listOfReactants>

<listOfProducts>

<speciesReference species="c_g3p" stoichiometry="1" constant="false"/>

<speciesReference species="c_pyr" stoichiometry="1" constant="false"/>

</listOfProducts>

</reaction>

<reaction id="PDX5PS" name="PDX5PS" reversible="false" fast="false" compartment="cell">

<listOfReactants>

<speciesReference species="c_dxyl5p" stoichiometry="1" constant="false"/>

<speciesReference species="c_nad" stoichiometry="1" constant="false"/>

<speciesReference species="c_phthr" stoichiometry="1" constant="false"/>

</listOfReactants>

<listOfProducts>

<speciesReference species="c_co2" stoichiometry="1" constant="false"/>

<speciesReference species="c_h" stoichiometry="1" constant="false"/>

<speciesReference species="c_h2o" stoichiometry="2" constant="false"/>

<speciesReference species="c_nadh" stoichiometry="1" constant="false"/>

<speciesReference species="c_pdx5p" stoichiometry="1" constant="false"/>

<speciesReference species="c_pi" stoichiometry="1" constant="false"/>

</listOfProducts>

</reaction>

<reaction id="MICITH" name="MICITH" reversible="false" fast="false" compartment="cell">

<listOfReactants>

<speciesReference species="c_2mcacn" stoichiometry="1" constant="false"/>

<speciesReference species="c_h2o" stoichiometry="1" constant="false"/>

</listOfReactants>

<listOfProducts>

<speciesReference species="c_micit" stoichiometry="1" constant="false"/>

</listOfProducts>

</reaction>

<reaction id="UAPGR" name="UAPGR" reversible="false" fast="false" compartment="cell">

<listOfReactants>

<speciesReference species="c_h" stoichiometry="1" constant="false"/>

<speciesReference species="c_nadph" stoichiometry="1" constant="false"/>

<speciesReference species="c_uaccg" stoichiometry="1" constant="false"/>

</listOfReactants>

<listOfProducts>

<speciesReference species="c_nadp" stoichiometry="1" constant="false"/>

<speciesReference species="c_uamr" stoichiometry="1" constant="false"/>

</listOfProducts>

</reaction>

<reaction id="PHETA1" name="PHETA1" reversible="true" fast="false" compartment="cell">

<listOfReactants>

<speciesReference species="c_akg" stoichiometry="1" constant="false"/>

<speciesReference species="c_phe_L" stoichiometry="1" constant="false"/>

</listOfReactants>

<listOfProducts>

<speciesReference species="c_glu_L" stoichiometry="1" constant="false"/>

<speciesReference species="c_phpyr" stoichiometry="1" constant="false"/>

</listOfProducts>

</reaction>

<reaction id="MICITL" name="MICITL" reversible="true" fast="false" compartment="cell">

<listOfReactants>

<speciesReference species="c_micit" stoichiometry="1" constant="false"/>

</listOfReactants>

<listOfProducts>

<speciesReference species="c_pyr" stoichiometry="1" constant="false"/>

<speciesReference species="c_succ" stoichiometry="1" constant="false"/>

</listOfProducts>

</reaction>

<reaction id="AHAI" name="AHAI" reversible="false" fast="false" compartment="cell">

<listOfReactants>

<speciesReference species="c_alac_S" stoichiometry="1" constant="false"/>

<speciesReference species="c_h" stoichiometry="1" constant="false"/>

<speciesReference species="c_nadph" stoichiometry="1" constant="false"/>

</listOfReactants>

<listOfProducts>

<speciesReference species="c_23dhmb" stoichiometry="1" constant="false"/>

<speciesReference species="c_nadp" stoichiometry="1" constant="false"/>

</listOfProducts>

</reaction>

<reaction id="MEPCT" name="MEPCT" reversible="false" fast="false" compartment="cell">

<listOfReactants>

<speciesReference species="c_2me4p" stoichiometry="1" constant="false"/>

<speciesReference species="c_ctp" stoichiometry="1" constant="false"/>

<speciesReference species="c_h" stoichiometry="1" constant="false"/>

</listOfReactants>

<listOfProducts>

<speciesReference species="c_4c2me" stoichiometry="1" constant="false"/>

<speciesReference species="c_ppi" stoichiometry="1" constant="false"/>

</listOfProducts>

</reaction>

<reaction id="PDX5PO" name="PDX5PO" reversible="true" fast="false" compartment="cell">

<listOfReactants>

<speciesReference species="c_o2" stoichiometry="1" constant="false"/>

<speciesReference species="c_pdx5p" stoichiometry="1" constant="false"/>

</listOfReactants>

<listOfProducts>

<speciesReference species="c_h" stoichiometry="1" constant="false"/>

<speciesReference species="c_h2o2" stoichiometry="1" constant="false"/>

<speciesReference species="c_pydx5p" stoichiometry="1" constant="false"/>

</listOfProducts>

</reaction>

<reaction id="PRPPS" name="PRPPS" reversible="true" fast="false" compartment="cell">

<listOfReactants>

<speciesReference species="c_atp" stoichiometry="1" constant="false"/>

<speciesReference species="c_r5p" stoichiometry="1" constant="false"/>

</listOfReactants>

<listOfProducts>

<speciesReference species="c_amp" stoichiometry="1" constant="false"/>

<speciesReference species="c_h" stoichiometry="1" constant="false"/>

<speciesReference species="c_prpp" stoichiometry="1" constant="false"/>

</listOfProducts>

</reaction>

<reaction id="SHCHF" name="SHCHF" reversible="false" fast="false" compartment="cell">

<listOfReactants>

<speciesReference species="c_fe2" stoichiometry="1" constant="false"/>

<speciesReference species="c_srch" stoichiometry="1" constant="false"/>

</listOfReactants>

<listOfProducts>

<speciesReference species="c_h" stoichiometry="2" constant="false"/>

<speciesReference species="c_sheme" stoichiometry="1" constant="false"/>

</listOfProducts>

</reaction>

<reaction id="DPR" name="DPR" reversible="false" fast="false" compartment="cell">

<listOfReactants>

<speciesReference species="c_2dhp" stoichiometry="1" constant="false"/>

<speciesReference species="c_h" stoichiometry="1" constant="false"/>

<speciesReference species="c_nadph" stoichiometry="1" constant="false"/>

</listOfReactants>

<listOfProducts>

<speciesReference species="c_nadp" stoichiometry="1" constant="false"/>

<speciesReference species="c_pant_R" stoichiometry="1" constant="false"/>

</listOfProducts>

</reaction>

<reaction id="DHFR" name="DHFR" reversible="true" fast="false" compartment="cell">

<listOfReactants>

<speciesReference species="c_dhf" stoichiometry="1" constant="false"/>

<speciesReference species="c_h" stoichiometry="1" constant="false"/>

<speciesReference species="c_nadph" stoichiometry="1" constant="false"/>

</listOfReactants>

<listOfProducts>

<speciesReference species="c_nadp" stoichiometry="1" constant="false"/>

<speciesReference species="c_thf" stoichiometry="1" constant="false"/>

</listOfProducts>

</reaction>

<reaction id="DHFS" name="DHFS" reversible="false" fast="false" compartment="cell">

<listOfReactants>

<speciesReference species="c_atp" stoichiometry="1" constant="false"/>

<speciesReference species="c_dhpt" stoichiometry="1" constant="false"/>

<speciesReference species="c_glu_L" stoichiometry="1" constant="false"/>

</listOfReactants>

<listOfProducts>

<speciesReference species="c_adp" stoichiometry="1" constant="false"/>

<speciesReference species="c_dhf" stoichiometry="1" constant="false"/>

<speciesReference species="c_h" stoichiometry="1" constant="false"/>

<speciesReference species="c_pi" stoichiometry="1" constant="false"/>

</listOfProducts>

</reaction>

<reaction id="ACKr" name="ACKr" reversible="true" fast="false" compartment="cell">

<listOfReactants>

<speciesReference species="c_ac" stoichiometry="1" constant="false"/>

<speciesReference species="c_atp" stoichiometry="1" constant="false"/>

</listOfReactants>

<listOfProducts>

<speciesReference species="c_actp" stoichiometry="1" constant="false"/>

<speciesReference species="c_adp" stoichiometry="1" constant="false"/>

</listOfProducts>

</reaction>

<reaction id="C171SN" name="C171SN" reversible="false" fast="false" compartment="cell">

<listOfReactants>

<speciesReference species="c_h" stoichiometry="20" constant="false"/>

<speciesReference species="c_malACP" stoichiometry="7" constant="false"/>

<speciesReference species="c_nadph" stoichiometry="13" constant="false"/>

<speciesReference species="c_ppcoa" stoichiometry="1" constant="false"/>

</listOfReactants>

<listOfProducts>

<speciesReference species="c_ACP" stoichiometry="6" constant="false"/>

<speciesReference species="c_co2" stoichiometry="7" constant="false"/>

<speciesReference species="c_coa" stoichiometry="1" constant="false"/>

<speciesReference species="c_h2o" stoichiometry="7" constant="false"/>

<speciesReference species="c_hpdeACP" stoichiometry="1" constant="false"/>

<speciesReference species="c_nadp" stoichiometry="13" constant="false"/>

</listOfProducts>

</reaction>

<reaction id="ARGDC" name="ARGDC" reversible="false" fast="false" compartment="cell">

<listOfReactants>

<speciesReference species="c_arg_L" stoichiometry="1" constant="false"/>

<speciesReference species="c_h" stoichiometry="1" constant="false"/>

</listOfReactants>

<listOfProducts>

<speciesReference species="c_agm" stoichiometry="1" constant="false"/>

<speciesReference species="c_co2" stoichiometry="1" constant="false"/>

</listOfProducts>

</reaction>

<reaction id="CAT" name="CAT" reversible="false" fast="false" compartment="cell">

<listOfReactants>

<speciesReference species="c_h2o2" stoichiometry="2" constant="false"/>

</listOfReactants>

<listOfProducts>

<speciesReference species="c_h2o" stoichiometry="2" constant="false"/>

<speciesReference species="c_o2" stoichiometry="1" constant="false"/>

</listOfProducts>

</reaction>

<reaction id="FTHFL" name="FTHFL" reversible="false" fast="false" compartment="cell">

<listOfReactants>

<speciesReference species="c_atp" stoichiometry="1" constant="false"/>

<speciesReference species="c_for" stoichiometry="1" constant="false"/>

<speciesReference species="c_thf" stoichiometry="1" constant="false"/>

</listOfReactants>

<listOfProducts>

<speciesReference species="c_10fthf" stoichiometry="1" constant="false"/>

<speciesReference species="c_adp" stoichiometry="1" constant="false"/>

<speciesReference species="c_pi" stoichiometry="1" constant="false"/>

</listOfProducts>

</reaction>

<reaction id="VALTA" name="VALTA" reversible="true" fast="false" compartment="cell">

<listOfReactants>

<speciesReference species="c_akg" stoichiometry="1" constant="false"/>

<speciesReference species="c_val_L" stoichiometry="1" constant="false"/>

</listOfReactants>

<listOfProducts>

<speciesReference species="c_3mob" stoichiometry="1" constant="false"/>

<speciesReference species="c_glu_L" stoichiometry="1" constant="false"/>

</listOfProducts>

</reaction>

<reaction id="FTHFD" name="FTHFD" reversible="false" fast="false" compartment="cell">

<listOfReactants>

<speciesReference species="c_10fthf" stoichiometry="1" constant="false"/>

<speciesReference species="c_h2o" stoichiometry="1" constant="false"/>

</listOfReactants>

<listOfProducts>

<speciesReference species="c_for" stoichiometry="1" constant="false"/>

<speciesReference species="c_h" stoichiometry="1" constant="false"/>

<speciesReference species="c_thf" stoichiometry="1" constant="false"/>

</listOfProducts>

</reaction>

<reaction id="SGDS" name="SGDS" reversible="false" fast="false" compartment="cell">

<listOfReactants>

<speciesReference species="c_h2o" stoichiometry="1" constant="false"/>

<speciesReference species="c_sucglu" stoichiometry="1" constant="false"/>

</listOfReactants>

<listOfProducts>

<speciesReference species="c_glu_L" stoichiometry="1" constant="false"/>

<speciesReference species="c_succ" stoichiometry="1" constant="false"/>

</listOfProducts>

</reaction>

<reaction id="PPNDH" name="PPNDH" reversible="false" fast="false" compartment="cell">

<listOfReactants>

<speciesReference species="c_h" stoichiometry="1" constant="false"/>

<speciesReference species="c_pphn" stoichiometry="1" constant="false"/>

</listOfReactants>

<listOfProducts>

<speciesReference species="c_co2" stoichiometry="1" constant="false"/>

<speciesReference species="c_h2o" stoichiometry="1" constant="false"/>

<speciesReference species="c_phpyr" stoichiometry="1" constant="false"/>

</listOfProducts>

</reaction>

<reaction id="ADK4" name="ADK4" reversible="true" fast="false" compartment="cell">

<listOfReactants>

<speciesReference species="c_amp" stoichiometry="1" constant="false"/>

<speciesReference species="c_itp" stoichiometry="1" constant="false"/>

</listOfReactants>

<listOfProducts>

<speciesReference species="c_adp" stoichiometry="1" constant="false"/>

<speciesReference species="c_idp" stoichiometry="1" constant="false"/>

</listOfProducts>

</reaction>

<reaction id="PGM" name="PGM" reversible="true" fast="false" compartment="cell">

<listOfReactants>

<speciesReference species="c_3pg" stoichiometry="1" constant="false"/>

</listOfReactants>

<listOfProducts>

<speciesReference species="c_2pg" stoichiometry="1" constant="false"/>

</listOfProducts>

</reaction>

<reaction id="ADK3" name="ADK3" reversible="true" fast="false" compartment="cell">

<listOfReactants>

<speciesReference species="c_amp" stoichiometry="1" constant="false"/>

<speciesReference species="c_gtp" stoichiometry="1" constant="false"/>

</listOfReactants>

<listOfProducts>

<speciesReference species="c_adp" stoichiometry="1" constant="false"/>

<speciesReference species="c_gdp" stoichiometry="1" constant="false"/>

</listOfProducts>

</reaction>

<reaction id="ADK1" name="ADK1" reversible="true" fast="false" compartment="cell">

<listOfReactants>

<speciesReference species="c_amp" stoichiometry="1" constant="false"/>

<speciesReference species="c_atp" stoichiometry="1" constant="false"/>

</listOfReactants>

<listOfProducts>

<speciesReference species="c_adp" stoichiometry="2" constant="false"/>

</listOfProducts>

</reaction>

<reaction id="GLUPRT" name="GLUPRT" reversible="false" fast="false" compartment="cell">

<listOfReactants>

<speciesReference species="c_gln_L" stoichiometry="1" constant="false"/>

<speciesReference species="c_h2o" stoichiometry="1" constant="false"/>

<speciesReference species="c_prpp" stoichiometry="1" constant="false"/>

</listOfReactants>

<listOfProducts>

<speciesReference species="c_glu_L" stoichiometry="1" constant="false"/>

<speciesReference species="c_ppi" stoichiometry="1" constant="false"/>

<speciesReference species="c_pram" stoichiometry="1" constant="false"/>

</listOfProducts>

</reaction>

<reaction id="HXPRT" name="HXPRT" reversible="false" fast="false" compartment="cell">

<listOfReactants>

<speciesReference species="c_hxan" stoichiometry="1" constant="false"/>

<speciesReference species="c_prpp" stoichiometry="1" constant="false"/>

</listOfReactants>

<listOfProducts>

<speciesReference species="c_imp" stoichiometry="1" constant="false"/>

<speciesReference species="c_ppi" stoichiometry="1" constant="false"/>

</listOfProducts>

</reaction>

<reaction id="SUCOAS" name="SUCOAS" reversible="true" fast="false" compartment="cell">

<listOfReactants>

<speciesReference species="c_atp" stoichiometry="1" constant="false"/>

<speciesReference species="c_coa" stoichiometry="1" constant="false"/>

<speciesReference species="c_succ" stoichiometry="1" constant="false"/>

</listOfReactants>

<listOfProducts>

<speciesReference species="c_adp" stoichiometry="1" constant="false"/>

<speciesReference species="c_pi" stoichiometry="1" constant="false"/>

<speciesReference species="c_succoa" stoichiometry="1" constant="false"/>

</listOfProducts>

</reaction>

<reaction id="METGL" name="METGL" reversible="false" fast="false" compartment="cell">

<listOfReactants>

<speciesReference species="c_h2o" stoichiometry="1" constant="false"/>

<speciesReference species="c_met_L" stoichiometry="1" constant="false"/>

</listOfReactants>

<listOfProducts>

<speciesReference species="c_2obut" stoichiometry="1" constant="false"/>

<speciesReference species="c_ch4s" stoichiometry="1" constant="false"/>

<speciesReference species="c_nh4" stoichiometry="1" constant="false"/>

</listOfProducts>

</reaction>

<reaction id="ASPTRS" name="ASPTRS" reversible="false" fast="false" compartment="cell">

<listOfReactants>

<speciesReference species="c_asp_L" stoichiometry="1" constant="false"/>

<speciesReference species="c_atp" stoichiometry="1" constant="false"/>

<speciesReference species="c_trnaasp" stoichiometry="1" constant="false"/>

</listOfReactants>

<listOfProducts>

<speciesReference species="c_amp" stoichiometry="1" constant="false"/>

<speciesReference species="c_asptrna" stoichiometry="1" constant="false"/>

<speciesReference species="c_ppi" stoichiometry="1" constant="false"/>

</listOfProducts>

</reaction>

<reaction id="DHORD8" name="DHORD8" reversible="false" fast="false" compartment="cell">

<listOfReactants>

<speciesReference species="c_dhor_S" stoichiometry="1" constant="false"/>

<speciesReference species="c_mmqn7" stoichiometry="1" constant="false"/>

</listOfReactants>

<listOfProducts>

<speciesReference species="c_mmql7" stoichiometry="1" constant="false"/>

<speciesReference species="c_orot" stoichiometry="1" constant="false"/>

</listOfProducts>

</reaction>

<reaction id="ADCOBHEXS" name="ADCOBHEXS" reversible="false" fast="false" compartment="cell">

<listOfReactants>

<speciesReference species="c_adcobdam" stoichiometry="1" constant="false"/>

<speciesReference species="c_atp" stoichiometry="4" constant="false"/>

<speciesReference species="c_gln_L" stoichiometry="4" constant="false"/>

<speciesReference species="c_h2o" stoichiometry="4" constant="false"/>

</listOfReactants>

<listOfProducts>

<speciesReference species="c_adcobhex" stoichiometry="1" constant="false"/>

<speciesReference species="c_adp" stoichiometry="4" constant="false"/>

<speciesReference species="c_glu_L" stoichiometry="4" constant="false"/>

<speciesReference species="c_h" stoichiometry="4" constant="false"/>

<speciesReference species="c_pi" stoichiometry="4" constant="false"/>

</listOfProducts>

</reaction>

<reaction id="KDOPP" name="KDOPP" reversible="false" fast="false" compartment="cell">

<listOfReactants>

<speciesReference species="c_h2o" stoichiometry="1" constant="false"/>

<speciesReference species="c_kdo8p" stoichiometry="1" constant="false"/>

</listOfReactants>

<listOfProducts>

<speciesReference species="c_kdo" stoichiometry="1" constant="false"/>

<speciesReference species="c_pi" stoichiometry="1" constant="false"/>

</listOfProducts>

</reaction>

<reaction id="KDOPS" name="KDOPS" reversible="false" fast="false" compartment="cell">

<listOfReactants>

<speciesReference species="c_ara5p" stoichiometry="1" constant="false"/>

<speciesReference species="c_h2o" stoichiometry="1" constant="false"/>

<speciesReference species="c_pep" stoichiometry="1" constant="false"/>

</listOfReactants>

<listOfProducts>

<speciesReference species="c_kdo8p" stoichiometry="1" constant="false"/>

<speciesReference species="c_pi" stoichiometry="1" constant="false"/>

</listOfProducts>

</reaction>

<reaction id="GMHEPAT" name="GMHEPAT" reversible="false" fast="false" compartment="cell">

<listOfReactants>

<speciesReference species="c_atp" stoichiometry="1" constant="false"/>

<speciesReference species="c_gmh1p" stoichiometry="1" constant="false"/>

<speciesReference species="c_h" stoichiometry="1" constant="false"/>

</listOfReactants>

<listOfProducts>

<speciesReference stoichiometry="1" constant="false"/>

<speciesReference species="c_ppi" stoichiometry="1" constant="false"/>

</listOfProducts>

</reaction>

<reaction id="OCBT" name="OCBT" reversible="true" fast="false" compartment="cell">

<listOfReactants>

<speciesReference species="c_cbp" stoichiometry="1" constant="false"/>

<speciesReference species="c_orn_L" stoichiometry="1" constant="false"/>

</listOfReactants>

<listOfProducts>

<speciesReference species="c_citr_L" stoichiometry="1" constant="false"/>

<speciesReference species="c_h" stoichiometry="1" constant="false"/>

<speciesReference species="c_pi" stoichiometry="1" constant="false"/>

</listOfProducts>

</reaction>

<reaction id="DHORD2" name="DHORD2" reversible="false" fast="false" compartment="cell">

<listOfReactants>

<speciesReference species="c_dhor_S" stoichiometry="1" constant="false"/>

<speciesReference species="c_ubq8" stoichiometry="1" constant="false"/>

</listOfReactants>

<listOfProducts>

<speciesReference species="c_orot" stoichiometry="1" constant="false"/>

<speciesReference species="c_ubq8h2" stoichiometry="1" constant="false"/>

</listOfProducts>

</reaction>

<reaction id="C181SN" name="C181SN" reversible="false" fast="false" compartment="cell">

<listOfReactants>

<speciesReference species="c_actACP" stoichiometry="1" constant="false"/>

<speciesReference species="c_h" stoichiometry="22" constant="false"/>

<speciesReference species="c_malACP" stoichiometry="7" constant="false"/>

<speciesReference species="c_nadph" stoichiometry="15" constant="false"/>

</listOfReactants>

<listOfProducts>

<speciesReference species="c_ACP" stoichiometry="7" constant="false"/>

<speciesReference species="c_co2" stoichiometry="7" constant="false"/>

<speciesReference species="c_h2o" stoichiometry="8" constant="false"/>

<speciesReference species="c_nadp" stoichiometry="15" constant="false"/>

<speciesReference species="c_octeACP" stoichiometry="1" constant="false"/>

</listOfProducts>

</reaction>

<reaction id="HEPTT" name="HEPTT" reversible="false" fast="false" compartment="cell">

<listOfReactants>

<speciesReference species="c_hepdp" stoichiometry="1" constant="false"/>

<speciesReference species="c_ipdp" stoichiometry="1" constant="false"/>

</listOfReactants>

<listOfProducts>

<speciesReference species="c_octdp" stoichiometry="1" constant="false"/>

<speciesReference species="c_ppi" stoichiometry="1" constant="false"/>

</listOfProducts>

</reaction>

<reaction id="ORPT" name="ORPT" reversible="true" fast="false" compartment="cell">

<listOfReactants>

<speciesReference species="c_orot5p" stoichiometry="1" constant="false"/>

<speciesReference species="c_ppi" stoichiometry="1" constant="false"/>

</listOfReactants>

<listOfProducts>

<speciesReference species="c_orot" stoichiometry="1" constant="false"/>

<speciesReference species="c_prpp" stoichiometry="1" constant="false"/>

</listOfProducts>

</reaction>

<reaction id="ATPPRT" name="ATPPRT" reversible="false" fast="false" compartment="cell">

<listOfReactants>

<speciesReference species="c_atp" stoichiometry="1" constant="false"/>

<speciesReference species="c_prpp" stoichiometry="1" constant="false"/>

</listOfReactants>

<listOfProducts>

<speciesReference species="c_ppi" stoichiometry="1" constant="false"/>

<speciesReference species="c_prbatp" stoichiometry="1" constant="false"/>

</listOfProducts>

</reaction>

<reaction id="USHD" name="USHD" reversible="false" fast="false" compartment="cell">

<listOfReactants>

<speciesReference species="c_h2o" stoichiometry="1" constant="false"/>

<speciesReference species="c_u23ga" stoichiometry="1" constant="false"/>

</listOfReactants>

<listOfProducts>

<speciesReference species="c_h" stoichiometry="2" constant="false"/>

<speciesReference species="c_lipidX" stoichiometry="1" constant="false"/>

<speciesReference species="c_ump" stoichiometry="1" constant="false"/>

</listOfProducts>

</reaction>

<reaction id="GMPS2" name="GMPS2" reversible="false" fast="false" compartment="cell">

<listOfReactants>

<speciesReference species="c_atp" stoichiometry="1" constant="false"/>

<speciesReference species="c_gln_L" stoichiometry="1" constant="false"/>

<speciesReference species="c_h2o" stoichiometry="1" constant="false"/>

<speciesReference species="c_xmp" stoichiometry="1" constant="false"/>

</listOfReactants>

<listOfProducts>

<speciesReference species="c_amp" stoichiometry="1" constant="false"/>

<speciesReference species="c_glu_L" stoichiometry="1" constant="false"/>

<speciesReference species="c_gmp" stoichiometry="1" constant="false"/>

<speciesReference species="c_h" stoichiometry="2" constant="false"/>

<speciesReference species="c_ppi" stoichiometry="1" constant="false"/>

</listOfProducts>

</reaction>

<reaction id="UAAGDS" name="UAAGDS" reversible="false" fast="false" compartment="cell">

<listOfReactants>

<speciesReference species="c_26dap_M" stoichiometry="1" constant="false"/>

<speciesReference species="c_atp" stoichiometry="1" constant="false"/>

<speciesReference species="c_uamag" stoichiometry="1" constant="false"/>

</listOfReactants>

<listOfProducts>

<speciesReference species="c_adp" stoichiometry="1" constant="false"/>

<speciesReference species="c_h" stoichiometry="1" constant="false"/>

<speciesReference species="c_pi" stoichiometry="1" constant="false"/>

<speciesReference species="c_ugmd" stoichiometry="1" constant="false"/>

</listOfProducts>

</reaction>

<reaction id="PRAMPC" name="PRAMPC" reversible="false" fast="false" compartment="cell">

<listOfReactants>

<speciesReference species="c_h" stoichiometry="1" constant="false"/>

<speciesReference species="c_h2o" stoichiometry="1" constant="false"/>

<speciesReference species="c_prbamp" stoichiometry="1" constant="false"/>

</listOfReactants>

<listOfProducts>

<speciesReference species="c_prfp" stoichiometry="1" constant="false"/>

</listOfProducts>

</reaction>

<reaction id="TMPKr" name="TMPKr" reversible="true" fast="false" compartment="cell">

<listOfReactants>

<speciesReference species="c_atp" stoichiometry="1" constant="false"/>

<speciesReference species="c_thmmp" stoichiometry="1" constant="false"/>

</listOfReactants>

<listOfProducts>

<speciesReference species="c_adp" stoichiometry="1" constant="false"/>

<speciesReference species="c_thmpp" stoichiometry="1" constant="false"/>

</listOfProducts>

</reaction>

<reaction id="ICDHy" name="ICDHy" reversible="true" fast="false" compartment="cell">

<listOfReactants>

<speciesReference species="c_icit" stoichiometry="1" constant="false"/>

<speciesReference species="c_nadp" stoichiometry="1" constant="false"/>

</listOfReactants>

<listOfProducts>

<speciesReference species="c_akg" stoichiometry="1" constant="false"/>

<speciesReference species="c_co2" stoichiometry="1" constant="false"/>

<speciesReference species="c_nadph" stoichiometry="1" constant="false"/>

</listOfProducts>

</reaction>

<reaction id="PGL" name="PGL" reversible="false" fast="false" compartment="cell">

<listOfReactants>

<speciesReference species="c_6pgl" stoichiometry="1" constant="false"/>

<speciesReference species="c_h2o" stoichiometry="1" constant="false"/>

</listOfReactants>

<listOfProducts>

<speciesReference species="c_6pgc" stoichiometry="1" constant="false"/>

<speciesReference species="c_h" stoichiometry="1" constant="false"/>

</listOfProducts>

</reaction>

<reaction id="CYSS" name="CYSS" reversible="false" fast="false" compartment="cell">

<listOfReactants>

<speciesReference species="c_acser" stoichiometry="1" constant="false"/>

<speciesReference species="c_h2s" stoichiometry="1" constant="false"/>

</listOfReactants>

<listOfProducts>

<speciesReference species="c_ac" stoichiometry="1" constant="false"/>

<speciesReference species="c_cys_L" stoichiometry="1" constant="false"/>

</listOfProducts>

</reaction>

<reaction id="DUTPDP" name="DUTPDP" reversible="false" fast="false" compartment="cell">

<listOfReactants>

<speciesReference species="c_dutp" stoichiometry="1" constant="false"/>

<speciesReference species="c_h2o" stoichiometry="1" constant="false"/>

</listOfReactants>

<listOfProducts>

<speciesReference species="c_dump" stoichiometry="1" constant="false"/>

<speciesReference species="c_h" stoichiometry="1" constant="false"/>

<speciesReference species="c_ppi" stoichiometry="1" constant="false"/>

</listOfProducts>

</reaction>

<reaction id="IPMD" name="IPMD" reversible="false" fast="false" compartment="cell">

<listOfReactants>

<speciesReference species="c_3c2hmp" stoichiometry="1" constant="false"/>

<speciesReference species="c_nad" stoichiometry="1" constant="false"/>

</listOfReactants>

<listOfProducts>

<speciesReference species="c_3c4mop" stoichiometry="1" constant="false"/>

<speciesReference species="c_h" stoichiometry="1" constant="false"/>

<speciesReference species="c_nadh" stoichiometry="1" constant="false"/>

</listOfProducts>

</reaction>

<reaction id="PGK" name="PGK" reversible="true" fast="false" compartment="cell">

<listOfReactants>

<speciesReference species="c_13dpg" stoichiometry="1" constant="false"/>

<speciesReference species="c_adp" stoichiometry="1" constant="false"/>

</listOfReactants>

<listOfProducts>

<speciesReference species="c_3pg" stoichiometry="1" constant="false"/>

<speciesReference species="c_atp" stoichiometry="1" constant="false"/>

</listOfProducts>

</reaction>

<reaction id="PYK" name="PYK" reversible="false" fast="false" compartment="cell">

<listOfReactants>

<speciesReference species="c_adp" stoichiometry="1" constant="false"/>

<speciesReference species="c_h" stoichiometry="1" constant="false"/>

<speciesReference species="c_pep" stoichiometry="1" constant="false"/>

</listOfReactants>

<listOfProducts>

<speciesReference species="c_atp" stoichiometry="1" constant="false"/>

<speciesReference species="c_pyr" stoichiometry="1" constant="false"/>

</listOfProducts>

</reaction>

<reaction id="PRAIi" name="PRAIi" reversible="false" fast="false" compartment="cell">

<listOfReactants>

<speciesReference species="c_pran" stoichiometry="1" constant="false"/>

</listOfReactants>

<listOfProducts>

<speciesReference species="c_2cpr5p" stoichiometry="1" constant="false"/>

</listOfProducts>

</reaction>

<reaction id="PAPSR" name="PAPSR" reversible="false" fast="false" compartment="cell">

<listOfReactants>

<speciesReference species="c_paps" stoichiometry="1" constant="false"/>

<speciesReference species="c_trdrd" stoichiometry="1" constant="false"/>

</listOfReactants>

<listOfProducts>

<speciesReference species="c_h" stoichiometry="2" constant="false"/>

<speciesReference species="c_pap" stoichiometry="1" constant="false"/>

<speciesReference species="c_so3" stoichiometry="1" constant="false"/>

<speciesReference species="c_trdox" stoichiometry="1" constant="false"/>

</listOfProducts>

</reaction>

<reaction name="5DOAN" reversible="false" fast="false" compartment="cell">

<listOfReactants>

<speciesReference species="c_dad_5" stoichiometry="1" constant="false"/>

<speciesReference species="c_h2o" stoichiometry="1" constant="false"/>

</listOfReactants>

<listOfProducts>

<speciesReference species="c_5drib" stoichiometry="1" constant="false"/>

<speciesReference species="c_ade" stoichiometry="1" constant="false"/>

</listOfProducts>

</reaction>

<reaction id="PGI" name="PGI" reversible="true" fast="false" compartment="cell">

<listOfReactants>

<speciesReference species="c_g6p" stoichiometry="1" constant="false"/>

</listOfReactants>

<listOfProducts>

<speciesReference species="c_f6p" stoichiometry="1" constant="false"/>

</listOfProducts>

</reaction>

<reaction id="DHDPRy" name="DHDPRy" reversible="false" fast="false" compartment="cell">

<listOfReactants>

<speciesReference species="c_23dhdp" stoichiometry="1" constant="false"/>

<speciesReference species="c_h" stoichiometry="1" constant="false"/>

<speciesReference species="c_nadph" stoichiometry="1" constant="false"/>

</listOfReactants>

<listOfProducts>

<speciesReference species="c_nadp" stoichiometry="1" constant="false"/>

<speciesReference species="c_thdp" stoichiometry="1" constant="false"/>

</listOfProducts>

</reaction>

<reaction id="PRAIS" name="PRAIS" reversible="false" fast="false" compartment="cell">

<listOfReactants>

<speciesReference species="c_atp" stoichiometry="1" constant="false"/>

<speciesReference species="c_fpram" stoichiometry="1" constant="false"/>

</listOfReactants>

<listOfProducts>

<speciesReference species="c_adp" stoichiometry="1" constant="false"/>

<speciesReference species="c_air" stoichiometry="1" constant="false"/>

<speciesReference species="c_h" stoichiometry="1" constant="false"/>

<speciesReference species="c_pi" stoichiometry="1" constant="false"/>

</listOfProducts>

</reaction>

<reaction id="DMOCT" name="DMOCT" reversible="false" fast="false" compartment="cell">

<listOfReactants>

<speciesReference species="c_ctp" stoichiometry="1" constant="false"/>

<speciesReference species="c_kdo" stoichiometry="1" constant="false"/>

</listOfReactants>

<listOfProducts>

<speciesReference species="c_ckdo" stoichiometry="1" constant="false"/>

<speciesReference species="c_ppi" stoichiometry="1" constant="false"/>

</listOfProducts>

</reaction>

<reaction id="MECDPS" name="MECDPS" reversible="false" fast="false" compartment="cell">

<listOfReactants>

<speciesReference species="c_2p4c2me" stoichiometry="1" constant="false"/>

</listOfReactants>

<listOfProducts>

<speciesReference species="c_2mecdp" stoichiometry="1" constant="false"/>

<speciesReference species="c_cmp" stoichiometry="1" constant="false"/>

</listOfProducts>

</reaction>

<reaction id="OMMBLHX" name="OMMBLHX" reversible="false" fast="false" compartment="cell">

<listOfReactants>

<speciesReference species="c_2ommbl" stoichiometry="1" constant="false"/>

<speciesReference species="c_o2" stoichiometry="0.5" constant="false"/>

</listOfReactants>

<listOfProducts>

<speciesReference species="c_2omhmbl" stoichiometry="1" constant="false"/>

</listOfProducts>

</reaction>

<reaction id="MECDPDH" name="MECDPDH" reversible="false" fast="false" compartment="cell">

<listOfReactants>

<speciesReference species="c_2mecdp" stoichiometry="1" constant="false"/>

<speciesReference species="c_nadh" stoichiometry="1" constant="false"/>

</listOfReactants>

<listOfProducts>

<speciesReference species="c_h2mb4p" stoichiometry="1" constant="false"/>

<speciesReference species="c_h2o" stoichiometry="1" constant="false"/>

<speciesReference species="c_nad" stoichiometry="1" constant="false"/>

</listOfProducts>

</reaction>

<reaction id="IMPC" name="IMPC" reversible="true" fast="false" compartment="cell">

<listOfReactants>

<speciesReference species="c_h2o" stoichiometry="1" constant="false"/>

<speciesReference species="c_imp" stoichiometry="1" constant="false"/>

</listOfReactants>

<listOfProducts>

<speciesReference species="c_fprica" stoichiometry="1" constant="false"/>

</listOfProducts>

</reaction>

<reaction id="PGCD" name="PGCD" reversible="false" fast="false" compartment="cell">

<listOfReactants>

<speciesReference species="c_3pg" stoichiometry="1" constant="false"/>

<speciesReference species="c_nad" stoichiometry="1" constant="false"/>

</listOfReactants>

<listOfProducts>

<speciesReference species="c_3php" stoichiometry="1" constant="false"/>

<speciesReference species="c_h" stoichiometry="1" constant="false"/>

<speciesReference species="c_nadh" stoichiometry="1" constant="false"/>

</listOfProducts>

</reaction>

<reaction id="THDPS" name="THDPS" reversible="false" fast="false" compartment="cell">

<listOfReactants>

<speciesReference species="c_h2o" stoichiometry="1" constant="false"/>

<speciesReference species="c_succoa" stoichiometry="1" constant="false"/>

<speciesReference species="c_thdp" stoichiometry="1" constant="false"/>

</listOfReactants>

<listOfProducts>

<speciesReference species="c_coa" stoichiometry="1" constant="false"/>

<speciesReference species="c_sl2a6o" stoichiometry="1" constant="false"/>

</listOfProducts>

</reaction>

<reaction id="IMPD" name="IMPD" reversible="false" fast="false" compartment="cell">

<listOfReactants>

<speciesReference species="c_h2o" stoichiometry="1" constant="false"/>

<speciesReference species="c_imp" stoichiometry="1" constant="false"/>

<speciesReference species="c_nad" stoichiometry="1" constant="false"/>

</listOfReactants>

<listOfProducts>

<speciesReference species="c_h" stoichiometry="1" constant="false"/>

<speciesReference species="c_nadh" stoichiometry="1" constant="false"/>

<speciesReference species="c_xmp" stoichiometry="1" constant="false"/>

</listOfProducts>

</reaction>

<reaction id="LYSTRS" name="LYSTRS" reversible="false" fast="false" compartment="cell">

<listOfReactants>

<speciesReference species="c_atp" stoichiometry="1" constant="false"/>

<speciesReference species="c_lys_L" stoichiometry="1" constant="false"/>

<speciesReference species="c_trnalys" stoichiometry="1" constant="false"/>

</listOfReactants>

<listOfProducts>

<speciesReference species="c_amp" stoichiometry="1" constant="false"/>

<speciesReference species="c_lystrna" stoichiometry="1" constant="false"/>

<speciesReference species="c_ppi" stoichiometry="1" constant="false"/>

</listOfProducts>

</reaction>

<reaction id="ACOATA" name="ACOATA" reversible="true" fast="false" compartment="cell">

<listOfReactants>

<speciesReference species="c_ACP" stoichiometry="1" constant="false"/>

<speciesReference species="c_accoa" stoichiometry="1" constant="false"/>

</listOfReactants>

<listOfProducts>

<speciesReference species="c_acACP" stoichiometry="1" constant="false"/>

<speciesReference species="c_coa" stoichiometry="1" constant="false"/>

</listOfProducts>

</reaction>

<reaction id="FMETTRS" name="FMETTRS" reversible="false" fast="false" compartment="cell">

<listOfReactants>

<speciesReference species="c_10fthf" stoichiometry="1" constant="false"/>

<speciesReference species="c_mettrna" stoichiometry="1" constant="false"/>

</listOfReactants>

<listOfProducts>

<speciesReference species="c_fmettrna" stoichiometry="1" constant="false"/>

<speciesReference species="c_thf" stoichiometry="1" constant="false"/>

</listOfProducts>

</reaction>

<reaction id="TPI" name="TPI" reversible="true" fast="false" compartment="cell">

<listOfReactants>

<speciesReference species="c_dhap" stoichiometry="1" constant="false"/>

</listOfReactants>

<listOfProducts>

<speciesReference species="c_g3p" stoichiometry="1" constant="false"/>

</listOfProducts>

</reaction>

<reaction id="AACPS15" name="AACPS15" reversible="false" fast="false" compartment="cell">

<listOfReactants>

<speciesReference species="c_ACP" stoichiometry="1" constant="false"/>

<speciesReference species="c_atp" stoichiometry="1" constant="false"/>

<speciesReference species="c_hpde" stoichiometry="1" constant="false"/>

</listOfReactants>

<listOfProducts>

<speciesReference species="c_amp" stoichiometry="1" constant="false"/>

<speciesReference species="c_hpdeACP" stoichiometry="1" constant="false"/>

<speciesReference species="c_ppi" stoichiometry="1" constant="false"/>

</listOfProducts>

</reaction>

<reaction id="DURIPP" name="DURIPP" reversible="true" fast="false" compartment="cell">

<listOfReactants>

<speciesReference species="c_duri" stoichiometry="1" constant="false"/>

<speciesReference species="c_pi" stoichiometry="1" constant="false"/>

</listOfReactants>

<listOfProducts>

<speciesReference species="c_2dr1p" stoichiometry="1" constant="false"/>

<speciesReference species="c_ura" stoichiometry="1" constant="false"/>

</listOfProducts>

</reaction>

<reaction id="RNASYN_Son" name="RNASYN_Son" reversible="false" fast="false" compartment="cell">

<listOfReactants>

<speciesReference species="c_atp" stoichiometry="127" constant="false"/>

<speciesReference species="c_ctp" stoichiometry="23" constant="false"/>

<speciesReference species="c_gtp" stoichiometry="23" constant="false"/>

<speciesReference species="c_h2o" stoichiometry="100" constant="false"/>

<speciesReference species="c_utp" stoichiometry="27" constant="false"/>

</listOfReactants>

<listOfProducts>

<speciesReference species="c_adp" stoichiometry="100" constant="false"/>

<speciesReference species="c_h" stoichiometry="100" constant="false"/>

<speciesReference species="c_pi" stoichiometry="100" constant="false"/>

<speciesReference species="c_ppi" stoichiometry="100" constant="false"/>

<speciesReference species="c_rna_Son" stoichiometry="1" constant="false"/>

</listOfProducts>

</reaction>

<reaction id="AGDC" name="AGDC" reversible="false" fast="false" compartment="cell">

<listOfReactants>

<speciesReference species="c_acgam6p" stoichiometry="1" constant="false"/>

<speciesReference species="c_h2o" stoichiometry="1" constant="false"/>

</listOfReactants>

<listOfProducts>

<speciesReference species="c_ac" stoichiometry="1" constant="false"/>

<speciesReference species="c_gam6p" stoichiometry="1" constant="false"/>

</listOfProducts>

</reaction>

<reaction id="PRKIN" name="PRKIN" reversible="false" fast="false" compartment="cell">

<listOfReactants>

<speciesReference species="c_atp" stoichiometry="1" constant="false"/>

<speciesReference species="c_ru5p_D" stoichiometry="1" constant="false"/>

</listOfReactants>

<listOfProducts>

<speciesReference species="c_adp" stoichiometry="1" constant="false"/>

<speciesReference species="c_h" stoichiometry="1" constant="false"/>

<speciesReference species="c_rb15bp" stoichiometry="1" constant="false"/>

</listOfProducts>

</reaction>

<reaction id="PRFGS" name="PRFGS" reversible="false" fast="false" compartment="cell">

<listOfReactants>

<speciesReference species="c_atp" stoichiometry="1" constant="false"/>

<speciesReference species="c_fgam" stoichiometry="1" constant="false"/>

<speciesReference species="c_gln_L" stoichiometry="1" constant="false"/>

<speciesReference species="c_h2o" stoichiometry="1" constant="false"/>

</listOfReactants>

<listOfProducts>

<speciesReference species="c_adp" stoichiometry="1" constant="false"/>

<speciesReference species="c_fpram" stoichiometry="1" constant="false"/>

<speciesReference species="c_glu_L" stoichiometry="1" constant="false"/>

<speciesReference species="c_h" stoichiometry="2" constant="false"/>

<speciesReference species="c_pi" stoichiometry="1" constant="false"/>

</listOfProducts>

</reaction>

<reaction id="DAPDC" name="DAPDC" reversible="false" fast="false" compartment="cell">

<listOfReactants>

<speciesReference species="c_26dap_M" stoichiometry="1" constant="false"/>

<speciesReference species="c_h" stoichiometry="1" constant="false"/>

</listOfReactants>

<listOfProducts>

<speciesReference species="c_co2" stoichiometry="1" constant="false"/>

<speciesReference species="c_lys_L" stoichiometry="1" constant="false"/>

</listOfProducts>

</reaction>

<reaction id="G35DP" name="G35DP" reversible="false" fast="false" compartment="cell">

<listOfReactants>

<speciesReference species="c_gdpdp" stoichiometry="1" constant="false"/>

<speciesReference species="c_h2o" stoichiometry="1" constant="false"/>

</listOfReactants>

<listOfProducts>

<speciesReference species="c_gdp" stoichiometry="1" constant="false"/>

<speciesReference species="c_ppi" stoichiometry="1" constant="false"/>

</listOfProducts>

</reaction>

<reaction id="ORNDC" name="ORNDC" reversible="false" fast="false" compartment="cell">

<listOfReactants>

<speciesReference species="c_h" stoichiometry="1" constant="false"/>

<speciesReference species="c_orn_L" stoichiometry="1" constant="false"/>

</listOfReactants>

<listOfProducts>

<speciesReference species="c_co2" stoichiometry="1" constant="false"/>

<speciesReference species="c_ptrc" stoichiometry="1" constant="false"/>

</listOfProducts>

</reaction>

<reaction id="GLYCLTDxr" name="GLYCLTDxr" reversible="true" fast="false" compartment="cell">

<listOfReactants>

<speciesReference species="c_glyclt" stoichiometry="1" constant="false"/>

<speciesReference species="c_nad" stoichiometry="1" constant="false"/>

</listOfReactants>

<listOfProducts>

<speciesReference species="c_glx" stoichiometry="1" constant="false"/>

<speciesReference species="c_h" stoichiometry="1" constant="false"/>

<speciesReference species="c_nadh" stoichiometry="1" constant="false"/>

</listOfProducts>

</reaction>

<reaction id="PSCVT" name="PSCVT" reversible="true" fast="false" compartment="cell">

<listOfReactants>

<speciesReference species="c_pep" stoichiometry="1" constant="false"/>

<speciesReference species="c_skm5p" stoichiometry="1" constant="false"/>

</listOfReactants>

<listOfProducts>

<speciesReference species="c_3psme" stoichiometry="1" constant="false"/>

<speciesReference species="c_pi" stoichiometry="1" constant="false"/>

</listOfProducts>

</reaction>

<reaction id="MTRI" name="MTRI" reversible="true" fast="false" compartment="cell">

<listOfReactants>

<speciesReference species="c_5mdr1p" stoichiometry="1" constant="false"/>

</listOfReactants>

<listOfProducts>

<speciesReference species="c_5mdru1p" stoichiometry="1" constant="false"/>

</listOfProducts>

</reaction>

<reaction id="HACOADr" name="HACOADr" reversible="false" fast="false" compartment="cell">

<listOfReactants>

<speciesReference species="c_3hmp" stoichiometry="1" constant="false"/>

<speciesReference species="c_nad" stoichiometry="1" constant="false"/>

</listOfReactants>

<listOfProducts>

<speciesReference species="c_h" stoichiometry="1" constant="false"/>

<speciesReference species="c_mmalsa" stoichiometry="1" constant="false"/>

<speciesReference species="c_nadh" stoichiometry="1" constant="false"/>

</listOfProducts>

</reaction>

<reaction id="MTRK" name="MTRK" reversible="false" fast="false" compartment="cell">

<listOfReactants>

<speciesReference species="c_5mtr" stoichiometry="1" constant="false"/>

<speciesReference species="c_atp" stoichiometry="1" constant="false"/>

</listOfReactants>

<listOfProducts>

<speciesReference species="c_5mdr1p" stoichiometry="1" constant="false"/>

<speciesReference species="c_adp" stoichiometry="1" constant="false"/>

<speciesReference species="c_h" stoichiometry="1" constant="false"/>

</listOfProducts>

</reaction>

<reaction id="MALTAT" name="MALTAT" reversible="false" fast="false" compartment="cell">

<listOfReactants>

<speciesReference species="c_accoa" stoichiometry="1" constant="false"/>

<speciesReference species="c_malt" stoichiometry="1" constant="false"/>

</listOfReactants>

<listOfProducts>

<speciesReference species="c_acmalt" stoichiometry="1" constant="false"/>

<speciesReference species="c_coa" stoichiometry="1" constant="false"/>

</listOfProducts>

</reaction>

<reaction id="SPMS" name="SPMS" reversible="false" fast="false" compartment="cell">

<listOfReactants>

<speciesReference species="c_ametam" stoichiometry="1" constant="false"/>

<speciesReference species="c_ptrc" stoichiometry="1" constant="false"/>

</listOfReactants>

<listOfProducts>

<speciesReference species="c_5mta" stoichiometry="1" constant="false"/>

<speciesReference species="c_h" stoichiometry="1" constant="false"/>

<speciesReference species="c_spmd" stoichiometry="1" constant="false"/>

</listOfProducts>

</reaction>

<reaction id="COBPS" name="COBPS" reversible="false" fast="false" compartment="cell">

<listOfReactants>

<speciesReference species="c_adgcoba" stoichiometry="1" constant="false"/>

<speciesReference species="c_rdmbzi" stoichiometry="1" constant="false"/>

</listOfReactants>

<listOfProducts>

<speciesReference species="c_cobamcoa" stoichiometry="1" constant="false"/>

<speciesReference species="c_gmp" stoichiometry="1" constant="false"/>

<speciesReference species="c_h" stoichiometry="1" constant="false"/>

</listOfProducts>

</reaction>

<reaction id="ASPCT" name="ASPCT" reversible="false" fast="false" compartment="cell">

<listOfReactants>

<speciesReference species="c_asp_L" stoichiometry="1" constant="false"/>

<speciesReference species="c_cbp" stoichiometry="1" constant="false"/>

</listOfReactants>

<listOfProducts>

<speciesReference species="c_cbasp" stoichiometry="1" constant="false"/>

<speciesReference species="c_h" stoichiometry="1" constant="false"/>

<speciesReference species="c_pi" stoichiometry="1" constant="false"/>

</listOfProducts>

</reaction>

<reaction id="ADNK1" name="ADNK1" reversible="false" fast="false" compartment="cell">

<listOfReactants>

<speciesReference species="c_adn" stoichiometry="1" constant="false"/>

<speciesReference species="c_atp" stoichiometry="1" constant="false"/>

</listOfReactants>

<listOfProducts>

<speciesReference species="c_adp" stoichiometry="1" constant="false"/>

<speciesReference species="c_amp" stoichiometry="1" constant="false"/>

<speciesReference species="c_h" stoichiometry="1" constant="false"/>

</listOfProducts>

</reaction>

<reaction id="CYTK1" name="CYTK1" reversible="true" fast="false" compartment="cell">

<listOfReactants>

<speciesReference species="c_atp" stoichiometry="1" constant="false"/>

<speciesReference species="c_cmp" stoichiometry="1" constant="false"/>

</listOfReactants>

<listOfProducts>

<speciesReference species="c_adp" stoichiometry="1" constant="false"/>

<speciesReference species="c_cdp" stoichiometry="1" constant="false"/>

</listOfProducts>

</reaction>

<reaction id="CYTK2" name="CYTK2" reversible="true" fast="false" compartment="cell">

<listOfReactants>

<speciesReference species="c_atp" stoichiometry="1" constant="false"/>

<speciesReference species="c_dcmp" stoichiometry="1" constant="false"/>

</listOfReactants>

<listOfProducts>

<speciesReference species="c_adp" stoichiometry="1" constant="false"/>

<speciesReference species="c_dcdp" stoichiometry="1" constant="false"/>

</listOfProducts>

</reaction>

<reaction id="NCTPPRT" name="NCTPPRT" reversible="true" fast="false" compartment="cell">

<listOfReactants>

<speciesReference species="c_nicrnt" stoichiometry="1" constant="false"/>

<speciesReference species="c_ppi" stoichiometry="1" constant="false"/>

</listOfReactants>

<listOfProducts>

<speciesReference species="c_h" stoichiometry="1" constant="false"/>

<speciesReference species="c_nac" stoichiometry="1" constant="false"/>

<speciesReference species="c_prpp" stoichiometry="1" constant="false"/>

</listOfProducts>

</reaction>

<reaction id="TRPTRS" name="TRPTRS" reversible="false" fast="false" compartment="cell">

<listOfReactants>

<speciesReference species="c_atp" stoichiometry="1" constant="false"/>

<speciesReference species="c_trnatrp" stoichiometry="1" constant="false"/>

<speciesReference species="c_trp_L" stoichiometry="1" constant="false"/>

</listOfReactants>

<listOfProducts>

<speciesReference species="c_amp" stoichiometry="1" constant="false"/>

<speciesReference species="c_ppi" stoichiometry="1" constant="false"/>

<speciesReference species="c_trptrna" stoichiometry="1" constant="false"/>

</listOfProducts>

</reaction>

<reaction id="C70ISN" name="C70ISN" reversible="false" fast="false" compartment="cell">

<listOfReactants>

<speciesReference species="c_h" stoichiometry="3" constant="false"/>

<speciesReference species="c_ivcoa" stoichiometry="1" constant="false"/>

<speciesReference species="c_malACP" stoichiometry="1" constant="false"/>

<speciesReference species="c_nadph" stoichiometry="2" constant="false"/>

</listOfReactants>

<listOfProducts>

<speciesReference species="c_co2" stoichiometry="1" constant="false"/>

<speciesReference species="c_coa" stoichiometry="1" constant="false"/>

<speciesReference species="c_fa7ACP" stoichiometry="1" constant="false"/>

<speciesReference species="c_h2o" stoichiometry="1" constant="false"/>

<speciesReference species="c_nadp" stoichiometry="2" constant="false"/>

</listOfProducts>

</reaction>

<reaction id="ME2" name="ME2" reversible="false" fast="false" compartment="cell">

<listOfReactants>

<speciesReference species="c_mal_L" stoichiometry="1" constant="false"/>

<speciesReference species="c_nadp" stoichiometry="1" constant="false"/>

</listOfReactants>

<listOfProducts>

<speciesReference species="c_co2" stoichiometry="1" constant="false"/>

<speciesReference species="c_nadph" stoichiometry="1" constant="false"/>

<speciesReference species="c_pyr" stoichiometry="1" constant="false"/>

</listOfProducts>

</reaction>

<reaction id="OIVD1i" name="OIVD1i" reversible="false" fast="false" compartment="cell">

<listOfReactants>

<speciesReference species="c_4mop" stoichiometry="1" constant="false"/>

<speciesReference species="c_coa" stoichiometry="1" constant="false"/>

<speciesReference species="c_nad" stoichiometry="1" constant="false"/>

</listOfReactants>

<listOfProducts>

<speciesReference species="c_co2" stoichiometry="1" constant="false"/>

<speciesReference species="c_ivcoa" stoichiometry="1" constant="false"/>

<speciesReference species="c_nadh" stoichiometry="1" constant="false"/>

</listOfProducts>

</reaction>

<reaction id="ACPSc" name="ACPSc" reversible="false" fast="false" compartment="cell">

<listOfReactants>

<speciesReference species="c_ACP" stoichiometry="1" constant="false"/>

<speciesReference species="c_h2o" stoichiometry="1" constant="false"/>

</listOfReactants>

<listOfProducts>

<speciesReference species="c_apoACP" stoichiometry="1" constant="false"/>

<speciesReference species="c_h" stoichiometry="1" constant="false"/>

<speciesReference species="c_pan4p" stoichiometry="1" constant="false"/>

</listOfProducts>

</reaction>

<reaction id="FAO6" name="FAO6" reversible="false" fast="false" compartment="cell">

<listOfReactants>

<speciesReference species="c_atp" stoichiometry="1" constant="false"/>

<speciesReference species="c_coa" stoichiometry="8" constant="false"/>

<speciesReference species="c_h2o" stoichiometry="7" constant="false"/>

<speciesReference species="c_hdca" stoichiometry="1" constant="false"/>

<speciesReference species="c_nad" stoichiometry="7" constant="false"/>

<speciesReference species="c_ubq8" stoichiometry="7" constant="false"/>

</listOfReactants>

<listOfProducts>

<speciesReference species="c_accoa" stoichiometry="8" constant="false"/>

<speciesReference species="c_amp" stoichiometry="1" constant="false"/>

<speciesReference species="c_h" stoichiometry="7" constant="false"/>

<speciesReference species="c_nadh" stoichiometry="7" constant="false"/>

<speciesReference species="c_ppi" stoichiometry="1" constant="false"/>

<speciesReference species="c_ubq8h2" stoichiometry="7" constant="false"/>

</listOfProducts>

</reaction>

<reaction id="FAO7" name="FAO7" reversible="false" fast="false" compartment="cell">

<listOfReactants>

<speciesReference species="c_atp" stoichiometry="1" constant="false"/>

<speciesReference species="c_coa" stoichiometry="9" constant="false"/>

<speciesReference species="c_h2o" stoichiometry="8" constant="false"/>

<speciesReference species="c_nad" stoichiometry="8" constant="false"/>

<speciesReference species="c_ocdca" stoichiometry="1" constant="false"/>

<speciesReference species="c_ubq8" stoichiometry="8" constant="false"/>

</listOfReactants>

<listOfProducts>

<speciesReference species="c_accoa" stoichiometry="9" constant="false"/>

<speciesReference species="c_amp" stoichiometry="1" constant="false"/>

<speciesReference species="c_h" stoichiometry="8" constant="false"/>

<speciesReference species="c_nadh" stoichiometry="8" constant="false"/>

<speciesReference species="c_ppi" stoichiometry="1" constant="false"/>

<speciesReference species="c_ubq8h2" stoichiometry="8" constant="false"/>

</listOfProducts>

</reaction>

<reaction id="DXPS" name="DXPS" reversible="false" fast="false" compartment="cell">

<listOfReactants>

<speciesReference species="c_g3p" stoichiometry="1" constant="false"/>

<speciesReference species="c_h" stoichiometry="1" constant="false"/>

<speciesReference species="c_pyr" stoichiometry="1" constant="false"/>

</listOfReactants>

<listOfProducts>

<speciesReference species="c_co2" stoichiometry="1" constant="false"/>

<speciesReference species="c_dxyl5p" stoichiometry="1" constant="false"/>

</listOfProducts>

</reaction>

<reaction id="MALTK" name="MALTK" reversible="false" fast="false" compartment="cell">

<listOfReactants>

<speciesReference species="c_atp" stoichiometry="1" constant="false"/>

<speciesReference species="c_coa" stoichiometry="1" constant="false"/>

<speciesReference species="c_mal_L" stoichiometry="1" constant="false"/>

</listOfReactants>

<listOfProducts>

<speciesReference species="c_adp" stoichiometry="1" constant="false"/>

<speciesReference species="c_malylcoa" stoichiometry="1" constant="false"/>

<speciesReference species="c_pi" stoichiometry="1" constant="false"/>

</listOfProducts>

</reaction>

<reaction id="FUM" name="FUM" reversible="true" fast="false" compartment="cell">

<listOfReactants>

<speciesReference species="c_fum" stoichiometry="1" constant="false"/>

<speciesReference species="c_h2o" stoichiometry="1" constant="false"/>

</listOfReactants>

<listOfProducts>

<speciesReference species="c_mal_L" stoichiometry="1" constant="false"/>

</listOfProducts>

</reaction>

<reaction id="PGDHY" name="PGDHY" reversible="false" fast="false" compartment="cell">

<listOfReactants>

<speciesReference species="c_6pgc" stoichiometry="1" constant="false"/>

</listOfReactants>

<listOfProducts>

<speciesReference species="c_2ddg6p" stoichiometry="1" constant="false"/>

<speciesReference species="c_h2o" stoichiometry="1" constant="false"/>

</listOfProducts>

</reaction>

<reaction id="DAPE" name="DAPE" reversible="true" fast="false" compartment="cell">

<listOfReactants>

<speciesReference species="c_26dap_LL" stoichiometry="1" constant="false"/>

</listOfReactants>

<listOfProducts>

<speciesReference species="c_26dap_M" stoichiometry="1" constant="false"/>

</listOfProducts>

</reaction>

<reaction id="HOXPRx" name="HOXPRx" reversible="true" fast="false" compartment="cell">

<listOfReactants>

<speciesReference species="c_glyc_R" stoichiometry="1" constant="false"/>

<speciesReference species="c_nad" stoichiometry="1" constant="false"/>

</listOfReactants>

<listOfProducts>

<speciesReference species="c_2h3opp" stoichiometry="1" constant="false"/>

<speciesReference species="c_h" stoichiometry="1" constant="false"/>

<speciesReference species="c_nadh" stoichiometry="1" constant="false"/>

</listOfProducts>

</reaction>

<reaction id="DCTPD" name="DCTPD" reversible="false" fast="false" compartment="cell">

<listOfReactants>

<speciesReference species="c_dctp" stoichiometry="1" constant="false"/>

<speciesReference species="c_h" stoichiometry="1" constant="false"/>

<speciesReference species="c_h2o" stoichiometry="1" constant="false"/>

</listOfReactants>

<listOfProducts>

<speciesReference species="c_dutp" stoichiometry="1" constant="false"/>

<speciesReference species="c_nh4" stoichiometry="1" constant="false"/>

</listOfProducts>

</reaction>

<reaction id="SADH" name="SADH" reversible="false" fast="false" compartment="cell">

<listOfReactants>

<speciesReference species="c_h" stoichiometry="2" constant="false"/>

<speciesReference species="c_h2o" stoichiometry="2" constant="false"/>

<speciesReference species="c_sucarg" stoichiometry="1" constant="false"/>

</listOfReactants>

<listOfProducts>

<speciesReference species="c_co2" stoichiometry="1" constant="false"/>

<speciesReference species="c_nh4" stoichiometry="2" constant="false"/>

<speciesReference species="c_sucorn" stoichiometry="1" constant="false"/>

</listOfProducts>

</reaction>

<reaction id="ILETA" name="ILETA" reversible="true" fast="false" compartment="cell">

<listOfReactants>

<speciesReference species="c_akg" stoichiometry="1" constant="false"/>

<speciesReference species="c_ile_L" stoichiometry="1" constant="false"/>

</listOfReactants>

<listOfProducts>

<speciesReference species="c_3mop" stoichiometry="1" constant="false"/>

<speciesReference species="c_glu_L" stoichiometry="1" constant="false"/>

</listOfProducts>

</reaction>

<reaction id="FMNAT" name="FMNAT" reversible="false" fast="false" compartment="cell">

<listOfReactants>

<speciesReference species="c_atp" stoichiometry="1" constant="false"/>

<speciesReference species="c_fmn" stoichiometry="1" constant="false"/>

<speciesReference species="c_h" stoichiometry="1" constant="false"/>

</listOfReactants>

<listOfProducts>

<speciesReference species="c_fad" stoichiometry="1" constant="false"/>

<speciesReference species="c_ppi" stoichiometry="1" constant="false"/>

</listOfProducts>

</reaction>

<reaction id="LDH_D5" name="LDH_D5" reversible="false" fast="false" compartment="cell">

<listOfReactants>

<speciesReference species="c_lac_D" stoichiometry="1" constant="false"/>

<speciesReference species="c_mmqn7" stoichiometry="1" constant="false"/>

</listOfReactants>

<listOfProducts>

<speciesReference species="c_mmql7" stoichiometry="1" constant="false"/>

<speciesReference species="c_pyr" stoichiometry="1" constant="false"/>

</listOfProducts>

</reaction>

<reaction id="LDH_D4" name="LDH_D4" reversible="false" fast="false" compartment="cell">

<listOfReactants>

<speciesReference species="c_lac_D" stoichiometry="1" constant="false"/>

<speciesReference species="c_mqn7" stoichiometry="1" constant="false"/>

</listOfReactants>

<listOfProducts>

<speciesReference species="c_mql7" stoichiometry="1" constant="false"/>

<speciesReference species="c_pyr" stoichiometry="1" constant="false"/>

</listOfProducts>

</reaction>

<reaction id="ENO" name="ENO" reversible="true" fast="false" compartment="cell">

<listOfReactants>

<speciesReference species="c_2pg" stoichiometry="1" constant="false"/>

</listOfReactants>

<listOfProducts>

<speciesReference species="c_h2o" stoichiometry="1" constant="false"/>

<speciesReference species="c_pep" stoichiometry="1" constant="false"/>

</listOfProducts>

</reaction>

<reaction id="PROTSYN_Son_aerobic" name="PROTSYN_Son_aerobic" reversible="false" fast="false" compartment="cell">

<listOfReactants>

<speciesReference species="c_alatrna" stoichiometry="1083" constant="false"/>

<speciesReference species="c_argtrna" stoichiometry="449" constant="false"/>

<speciesReference species="c_asntrna" stoichiometry="507" constant="false"/>

<speciesReference species="c_asptrna" stoichiometry="660" constant="false"/>

<speciesReference species="c_cystrna" stoichiometry="107" constant="false"/>

<speciesReference species="c_glntrna" stoichiometry="524" constant="false"/>

<speciesReference species="c_glutrna" stoichiometry="612" constant="false"/>

<speciesReference species="c_glytrna" stoichiometry="934" constant="false"/>

<speciesReference species="c_gtp" stoichiometry="19944" constant="false"/>

<speciesReference species="c_h2o" stoichiometry="19974" constant="false"/>

<speciesReference species="c_histrna" stoichiometry="177" constant="false"/>

<speciesReference species="c_iletrna" stoichiometry="506" constant="false"/>

<speciesReference species="c_leutrna" stoichiometry="777" constant="false"/>

<speciesReference species="c_lystrna" stoichiometry="505" constant="false"/>

<speciesReference species="c_mettrna" stoichiometry="257" constant="false"/>

<speciesReference species="c_phetrna" stoichiometry="330" constant="false"/>

<speciesReference species="c_protrna" stoichiometry="436" constant="false"/>

<speciesReference species="c_sertrna" stoichiometry="598" constant="false"/>

<speciesReference species="c_thrtrna" stoichiometry="566" constant="false"/>

<speciesReference species="c_trptrna" stoichiometry="125" constant="false"/>

<speciesReference species="c_tyrtrna" stoichiometry="253" constant="false"/>

<speciesReference species="c_valtrna" stoichiometry="596" constant="false"/>

</listOfReactants>

<listOfProducts>

<speciesReference species="c_gdp" stoichiometry="19944" constant="false"/>

<speciesReference species="c_h" stoichiometry="29946" constant="false"/>

<speciesReference species="c_pi" stoichiometry="19944" constant="false"/>

<speciesReference species="c_protein_Son_aerobic" stoichiometry="1" constant="false"/>

<speciesReference species="c_trnaala" stoichiometry="1083" constant="false"/>

<speciesReference species="c_trnaarg" stoichiometry="449" constant="false"/>

<speciesReference species="c_trnaasn" stoichiometry="507" constant="false"/>

<speciesReference species="c_trnaasp" stoichiometry="660" constant="false"/>

<speciesReference species="c_trnacys" stoichiometry="107" constant="false"/>

<speciesReference species="c_trnagln" stoichiometry="524" constant="false"/>

<speciesReference species="c_trnaglu" stoichiometry="612" constant="false"/>

<speciesReference species="c_trnagly" stoichiometry="934" constant="false"/>

<speciesReference species="c_trnahis" stoichiometry="177" constant="false"/>

<speciesReference species="c_trnaile" stoichiometry="506" constant="false"/>

<speciesReference species="c_trnaleu" stoichiometry="777" constant="false"/>

<speciesReference species="c_trnalys" stoichiometry="505" constant="false"/>

<speciesReference species="c_trnamet" stoichiometry="257" constant="false"/>

<speciesReference species="c_trnaphe" stoichiometry="330" constant="false"/>

<speciesReference species="c_trnapro" stoichiometry="436" constant="false"/>

<speciesReference species="c_trnaser" stoichiometry="598" constant="false"/>

<speciesReference species="c_trnathr" stoichiometry="566" constant="false"/>

<speciesReference species="c_trnatrp" stoichiometry="125" constant="false"/>

<speciesReference species="c_trnatyr" stoichiometry="253" constant="false"/>

<speciesReference species="c_trnaval" stoichiometry="596" constant="false"/>

</listOfProducts>

</reaction>

<reaction id="ACPS1" name="ACPS1" reversible="false" fast="false" compartment="cell">

<listOfReactants>

<speciesReference species="c_apoACP" stoichiometry="1" constant="false"/>

<speciesReference species="c_coa" stoichiometry="1" constant="false"/>

</listOfReactants>

<listOfProducts>

<speciesReference species="c_ACP" stoichiometry="1" constant="false"/>

<speciesReference species="c_h" stoichiometry="1" constant="false"/>

<speciesReference species="c_pap" stoichiometry="1" constant="false"/>

</listOfProducts>

</reaction>

<reaction id="IPPMIa" name="IPPMIa" reversible="true" fast="false" compartment="cell">

<listOfReactants>

<speciesReference species="c_3c2hmp" stoichiometry="1" constant="false"/>

</listOfReactants>

<listOfProducts>

<speciesReference species="c_2ippm" stoichiometry="1" constant="false"/>

<speciesReference species="c_h2o" stoichiometry="1" constant="false"/>

</listOfProducts>

</reaction>

<reaction id="MDRPD" name="MDRPD" reversible="false" fast="false" compartment="cell">

<listOfReactants>

<speciesReference species="c_5mdru1p" stoichiometry="1" constant="false"/>

</listOfReactants>

<listOfProducts>

<speciesReference species="c_dkmpp" stoichiometry="1" constant="false"/>

<speciesReference species="c_h2o" stoichiometry="1" constant="false"/>

</listOfProducts>

</reaction>

<reaction id="DNASYN_Son" name="DNASYN_Son" reversible="false" fast="false" compartment="cell">

<listOfReactants>

<speciesReference species="c_atp" stoichiometry="100" constant="false"/>

<speciesReference species="c_datp" stoichiometry="27" constant="false"/>

<speciesReference species="c_dctp" stoichiometry="23" constant="false"/>

<speciesReference species="c_dgtp" stoichiometry="23" constant="false"/>

<speciesReference species="c_dttp" stoichiometry="27" constant="false"/>

<speciesReference species="c_h2o" stoichiometry="100" constant="false"/>

</listOfReactants>

<listOfProducts>

<speciesReference species="c_adp" stoichiometry="100" constant="false"/>

<speciesReference species="c_dna_Son" stoichiometry="1" constant="false"/>

<speciesReference species="c_h" stoichiometry="100" constant="false"/>

<speciesReference species="c_pi" stoichiometry="100" constant="false"/>

<speciesReference species="c_ppi" stoichiometry="100" constant="false"/>

</listOfProducts>

</reaction>

<reaction id="SOTA" name="SOTA" reversible="false" fast="false" compartment="cell">

<listOfReactants>

<speciesReference species="c_akg" stoichiometry="1" constant="false"/>

<speciesReference species="c_sucorn" stoichiometry="1" constant="false"/>

</listOfReactants>

<listOfProducts>

<speciesReference species="c_glu_L" stoichiometry="1" constant="false"/>

<speciesReference species="c_sucgsa" stoichiometry="1" constant="false"/>

</listOfProducts>

</reaction>

<reaction id="AMALT4" name="AMALT4" reversible="false" fast="false" compartment="cell">

<listOfReactants>

<speciesReference species="c_malt" stoichiometry="1" constant="false"/>

<speciesReference species="c_malthx" stoichiometry="1" constant="false"/>

</listOfReactants>

<listOfProducts>

<speciesReference species="c_glc_D" stoichiometry="1" constant="false"/>

<speciesReference species="c_malthp" stoichiometry="1" constant="false"/>

</listOfProducts>

</reaction>

<reaction id="AMALT3" name="AMALT3" reversible="false" fast="false" compartment="cell">

<listOfReactants>

<speciesReference species="c_malt" stoichiometry="1" constant="false"/>

<speciesReference species="c_maltpt" stoichiometry="1" constant="false"/>

</listOfReactants>

<listOfProducts>

<speciesReference species="c_glc_D" stoichiometry="1" constant="false"/>

<speciesReference species="c_malthx" stoichiometry="1" constant="false"/>

</listOfProducts>

</reaction>

<reaction id="AMALT2" name="AMALT2" reversible="false" fast="false" compartment="cell">

<listOfReactants>

<speciesReference species="c_malt" stoichiometry="1" constant="false"/>

<speciesReference species="c_maltttr" stoichiometry="1" constant="false"/>

</listOfReactants>

<listOfProducts>

<speciesReference species="c_glc_D" stoichiometry="1" constant="false"/>

<speciesReference species="c_maltpt" stoichiometry="1" constant="false"/>

</listOfProducts>

</reaction>

<reaction id="AMALT1" name="AMALT1" reversible="false" fast="false" compartment="cell">

<listOfReactants>

<speciesReference species="c_malt" stoichiometry="1" constant="false"/>

<speciesReference species="c_malttr" stoichiometry="1" constant="false"/>

</listOfReactants>

<listOfProducts>

<speciesReference species="c_glc_D" stoichiometry="1" constant="false"/>

<speciesReference species="c_maltttr" stoichiometry="1" constant="false"/>

</listOfProducts>

</reaction>

<reaction id="NTPP2" name="NTPP2" reversible="false" fast="false" compartment="cell">

<listOfReactants>

<speciesReference species="c_gtp" stoichiometry="1" constant="false"/>

<speciesReference species="c_h2o" stoichiometry="1" constant="false"/>

</listOfReactants>

<listOfProducts>

<speciesReference species="c_gmp" stoichiometry="1" constant="false"/>

<speciesReference species="c_h" stoichiometry="1" constant="false"/>

<speciesReference species="c_ppi" stoichiometry="1" constant="false"/>

</listOfProducts>

</reaction>

<reaction id="DHPPDA2" name="DHPPDA2" reversible="false" fast="false" compartment="cell">

<listOfReactants>

<speciesReference species="c_25dhpp" stoichiometry="1" constant="false"/>

<speciesReference species="c_h" stoichiometry="1" constant="false"/>

<speciesReference species="c_h2o" stoichiometry="1" constant="false"/>

</listOfReactants>

<listOfProducts>

<speciesReference species="c_5apru" stoichiometry="1" constant="false"/>

<speciesReference species="c_nh4" stoichiometry="1" constant="false"/>

</listOfProducts>

</reaction>

<reaction id="GTHS" name="GTHS" reversible="false" fast="false" compartment="cell">

<listOfReactants>

<speciesReference species="c_atp" stoichiometry="1" constant="false"/>

<speciesReference species="c_glucys" stoichiometry="1" constant="false"/>

<speciesReference species="c_gly" stoichiometry="1" constant="false"/>

</listOfReactants>

<listOfProducts>

<speciesReference species="c_adp" stoichiometry="1" constant="false"/>

<speciesReference species="c_gthrd" stoichiometry="1" constant="false"/>

<speciesReference species="c_h" stoichiometry="1" constant="false"/>

<speciesReference species="c_pi" stoichiometry="1" constant="false"/>

</listOfProducts>

</reaction>

<reaction id="ACONT" name="ACONT" reversible="true" fast="false" compartment="cell">

<listOfReactants>

<speciesReference species="c_cit" stoichiometry="1" constant="false"/>

</listOfReactants>

<listOfProducts>

<speciesReference species="c_icit" stoichiometry="1" constant="false"/>

</listOfProducts>

</reaction>

<reaction id="UPP3S" name="UPP3S" reversible="false" fast="false" compartment="cell">

<listOfReactants>

<speciesReference species="c_hmbil" stoichiometry="1" constant="false"/>

</listOfReactants>

<listOfProducts>

<speciesReference species="c_h2o" stoichiometry="1" constant="false"/>

<speciesReference species="c_uppg3" stoichiometry="1" constant="false"/>

</listOfProducts>

</reaction>

<reaction id="ADSS" name="ADSS" reversible="false" fast="false" compartment="cell">

<listOfReactants>

<speciesReference species="c_asp_L" stoichiometry="1" constant="false"/>

<speciesReference species="c_gtp" stoichiometry="1" constant="false"/>

<speciesReference species="c_imp" stoichiometry="1" constant="false"/>

</listOfReactants>

<listOfProducts>

<speciesReference species="c_dcamp" stoichiometry="1" constant="false"/>

<speciesReference species="c_gdp" stoichiometry="1" constant="false"/>

<speciesReference species="c_h" stoichiometry="2" constant="false"/>

<speciesReference species="c_pi" stoichiometry="1" constant="false"/>

</listOfProducts>

</reaction>

<reaction id="SUCBZS" name="SUCBZS" reversible="false" fast="false" compartment="cell">

<listOfReactants>

<speciesReference species="c_2shchc" stoichiometry="1" constant="false"/>

</listOfReactants>

<listOfProducts>

<speciesReference species="c_h2o" stoichiometry="1" constant="false"/>

<speciesReference species="c_sucbz" stoichiometry="1" constant="false"/>

</listOfProducts>

</reaction>

<reaction id="HGENDO" name="HGENDO" reversible="false" fast="false" compartment="cell">

<listOfReactants>

<speciesReference species="c_hgentis" stoichiometry="1" constant="false"/>

<speciesReference species="c_o2" stoichiometry="1" constant="false"/>

</listOfReactants>

<listOfProducts>

<speciesReference species="c_4mlacac" stoichiometry="1" constant="false"/>

<speciesReference species="c_h" stoichiometry="1" constant="false"/>

</listOfProducts>

</reaction>

<reaction id="SHCHCS2" name="SHCHCS2" reversible="false" fast="false" compartment="cell">

<listOfReactants>

<speciesReference species="c_ichor" stoichiometry="1" constant="false"/>

<speciesReference species="c_ssaltpp" stoichiometry="1" constant="false"/>

</listOfReactants>

<listOfProducts>

<speciesReference species="c_2shchc" stoichiometry="1" constant="false"/>

<speciesReference species="c_h" stoichiometry="1" constant="false"/>

<speciesReference species="c_pyr" stoichiometry="1" constant="false"/>

<speciesReference species="c_thmpp" stoichiometry="1" constant="false"/>

</listOfProducts>

</reaction>

<reaction id="SHSL1" name="SHSL1" reversible="false" fast="false" compartment="cell">

<listOfReactants>

<speciesReference species="c_cys_L" stoichiometry="1" constant="false"/>

<speciesReference species="c_suchms" stoichiometry="1" constant="false"/>

</listOfReactants>

<listOfProducts>

<speciesReference species="c_cysth_L" stoichiometry="1" constant="false"/>

<speciesReference species="c_h" stoichiometry="1" constant="false"/>

<speciesReference species="c_succ" stoichiometry="1" constant="false"/>

</listOfProducts>

</reaction>

<reaction id="MTHFR2" name="MTHFR2" reversible="false" fast="false" compartment="cell">

<listOfReactants>

<speciesReference species="c_h" stoichiometry="2" constant="false"/>

<speciesReference species="c_mlthf" stoichiometry="1" constant="false"/>

<speciesReference species="c_nadh" stoichiometry="1" constant="false"/>

</listOfReactants>

<listOfProducts>

<speciesReference species="c_5mthf" stoichiometry="1" constant="false"/>

<speciesReference species="c_nad" stoichiometry="1" constant="false"/>

</listOfProducts>

</reaction>

<reaction id="PSP_L" name="PSP_L" reversible="false" fast="false" compartment="cell">

<listOfReactants>

<speciesReference species="c_h2o" stoichiometry="1" constant="false"/>

<speciesReference species="c_pser_L" stoichiometry="1" constant="false"/>

</listOfReactants>

<listOfProducts>

<speciesReference species="c_pi" stoichiometry="1" constant="false"/>

<speciesReference species="c_ser_L" stoichiometry="1" constant="false"/>

</listOfProducts>

</reaction>

<reaction id="HPPK" name="HPPK" reversible="false" fast="false" compartment="cell">

<listOfReactants>

<speciesReference species="c_2ahhmp" stoichiometry="1" constant="false"/>

<speciesReference species="c_atp" stoichiometry="1" constant="false"/>

</listOfReactants>

<listOfProducts>

<speciesReference species="c_2ahhmd" stoichiometry="1" constant="false"/>

<speciesReference species="c_amp" stoichiometry="1" constant="false"/>

<speciesReference species="c_h" stoichiometry="1" constant="false"/>

</listOfProducts>

</reaction>

<reaction id="G5SD" name="G5SD" reversible="false" fast="false" compartment="cell">

<listOfReactants>

<speciesReference species="c_glu5p" stoichiometry="1" constant="false"/>

<speciesReference species="c_h" stoichiometry="1" constant="false"/>

<speciesReference species="c_nadph" stoichiometry="1" constant="false"/>

</listOfReactants>

<listOfProducts>

<speciesReference species="c_glu5sa" stoichiometry="1" constant="false"/>

<speciesReference species="c_nadp" stoichiometry="1" constant="false"/>

<speciesReference species="c_pi" stoichiometry="1" constant="false"/>

</listOfProducts>

</reaction>

<reaction id="IGPS" name="IGPS" reversible="false" fast="false" compartment="cell">

<listOfReactants>

<speciesReference species="c_2cpr5p" stoichiometry="1" constant="false"/>

<speciesReference species="c_h" stoichiometry="1" constant="false"/>

</listOfReactants>

<listOfProducts>

<speciesReference species="c_3ig3p" stoichiometry="1" constant="false"/>

<speciesReference species="c_co2" stoichiometry="1" constant="false"/>

<speciesReference species="c_h2o" stoichiometry="1" constant="false"/>

</listOfProducts>

</reaction>

<reaction id="VALTRS" name="VALTRS" reversible="false" fast="false" compartment="cell">

<listOfReactants>

<speciesReference species="c_atp" stoichiometry="1" constant="false"/>

<speciesReference species="c_trnaval" stoichiometry="1" constant="false"/>

<speciesReference species="c_val_L" stoichiometry="1" constant="false"/>

</listOfReactants>

<listOfProducts>

<speciesReference species="c_amp" stoichiometry="1" constant="false"/>

<speciesReference species="c_ppi" stoichiometry="1" constant="false"/>

<speciesReference species="c_valtrna" stoichiometry="1" constant="false"/>

</listOfProducts>

</reaction>

<reaction id="ADSK" name="ADSK" reversible="false" fast="false" compartment="cell">

<listOfReactants>

<speciesReference species="c_aps" stoichiometry="1" constant="false"/>

<speciesReference species="c_atp" stoichiometry="1" constant="false"/>

</listOfReactants>

<listOfProducts>

<speciesReference species="c_adp" stoichiometry="1" constant="false"/>

<speciesReference species="c_h" stoichiometry="1" constant="false"/>

<speciesReference species="c_paps" stoichiometry="1" constant="false"/>

</listOfProducts>

</reaction>

<reaction id="DGTPH" name="DGTPH" reversible="false" fast="false" compartment="cell">

<listOfReactants>

<speciesReference species="c_dgtp" stoichiometry="1" constant="false"/>

<speciesReference species="c_h2o" stoichiometry="1" constant="false"/>

</listOfReactants>

<listOfProducts>

<speciesReference species="c_dgsn" stoichiometry="1" constant="false"/>

<speciesReference species="c_pppi" stoichiometry="1" constant="false"/>

</listOfProducts>

</reaction>

<reaction id="DNTPPA" name="DNTPPA" reversible="false" fast="false" compartment="cell">

<listOfReactants>

<speciesReference species="c_ahdt" stoichiometry="1" constant="false"/>

<speciesReference species="c_h2o" stoichiometry="1" constant="false"/>

</listOfReactants>

<listOfProducts>

<speciesReference species="c_dhpmp" stoichiometry="1" constant="false"/>

<speciesReference species="c_h" stoichiometry="1" constant="false"/>

<speciesReference species="c_ppi" stoichiometry="1" constant="false"/>

</listOfProducts>

</reaction>

<reaction id="S7PI" name="S7PI" reversible="true" fast="false" compartment="cell">

<listOfReactants>

<speciesReference species="c_s7p" stoichiometry="1" constant="false"/>

</listOfReactants>

<listOfProducts>

<speciesReference species="c_gmh7p" stoichiometry="1" constant="false"/>

</listOfProducts>

</reaction>

<reaction id="AST" name="AST" reversible="false" fast="false" compartment="cell">

<listOfReactants>

<speciesReference species="c_arg_L" stoichiometry="1" constant="false"/>

<speciesReference species="c_succoa" stoichiometry="1" constant="false"/>

</listOfReactants>

<listOfProducts>

<speciesReference species="c_coa" stoichiometry="1" constant="false"/>

<speciesReference species="c_h" stoichiometry="1" constant="false"/>

<speciesReference species="c_sucarg" stoichiometry="1" constant="false"/>

</listOfProducts>

</reaction>

<reaction id="ALATA_L" name="ALATA_L" reversible="true" fast="false" compartment="cell">

<listOfReactants>

<speciesReference species="c_akg" stoichiometry="1" constant="false"/>

<speciesReference species="c_ala_L" stoichiometry="1" constant="false"/>

</listOfReactants>

<listOfProducts>

<speciesReference species="c_glu_L" stoichiometry="1" constant="false"/>

<speciesReference species="c_pyr" stoichiometry="1" constant="false"/>

</listOfProducts>

</reaction>

<reaction id="SERTRS" name="SERTRS" reversible="false" fast="false" compartment="cell">

<listOfReactants>

<speciesReference species="c_atp" stoichiometry="1" constant="false"/>

<speciesReference species="c_ser_L" stoichiometry="1" constant="false"/>

<speciesReference species="c_trnaser" stoichiometry="1" constant="false"/>

</listOfReactants>

<listOfProducts>

<speciesReference species="c_amp" stoichiometry="1" constant="false"/>

<speciesReference species="c_ppi" stoichiometry="1" constant="false"/>

<speciesReference species="c_sertrna" stoichiometry="1" constant="false"/>

</listOfProducts>

</reaction>

<reaction id="THD5" name="THD5" reversible="false" fast="false" compartment="cell">

<listOfReactants>

<speciesReference species="c_nad" stoichiometry="1" constant="false"/>

<speciesReference species="c_nadph" stoichiometry="1" constant="false"/>

</listOfReactants>

<listOfProducts>

<speciesReference species="c_nadh" stoichiometry="1" constant="false"/>

<speciesReference species="c_nadp" stoichiometry="1" constant="false"/>

</listOfProducts>

</reaction>

<reaction id="UAGAAT" name="UAGAAT" reversible="true" fast="false" compartment="cell">

<listOfReactants>

<speciesReference species="c_3htdACP" stoichiometry="1" constant="false"/>

<speciesReference species="c_uacgam" stoichiometry="1" constant="false"/>

</listOfReactants>

<listOfProducts>

<speciesReference species="c_ACP" stoichiometry="1" constant="false"/>

<speciesReference species="c_u3aga" stoichiometry="1" constant="false"/>

</listOfProducts>

</reaction>

<reaction id="C161SN" name="C161SN" reversible="false" fast="false" compartment="cell">

<listOfReactants>

<speciesReference species="c_actACP" stoichiometry="1" constant="false"/>

<speciesReference species="c_h" stoichiometry="19" constant="false"/>

<speciesReference species="c_malACP" stoichiometry="6" constant="false"/>

<speciesReference species="c_nadph" stoichiometry="13" constant="false"/>

</listOfReactants>

<listOfProducts>

<speciesReference species="c_ACP" stoichiometry="6" constant="false"/>

<speciesReference species="c_co2" stoichiometry="6" constant="false"/>

<speciesReference species="c_h2o" stoichiometry="7" constant="false"/>

<speciesReference species="c_hdeACP" stoichiometry="1" constant="false"/>

<speciesReference species="c_nadp" stoichiometry="13" constant="false"/>

</listOfProducts>

</reaction>

<reaction id="AMPMS" name="AMPMS" reversible="false" fast="false" compartment="cell">

<listOfReactants>

<speciesReference species="c_air" stoichiometry="1" constant="false"/>

<speciesReference species="c_h2o" stoichiometry="1" constant="false"/>

</listOfReactants>

<listOfProducts>

<speciesReference species="c_4ampm" stoichiometry="1" constant="false"/>

<speciesReference species="c_for" stoichiometry="2" constant="false"/>

<speciesReference species="c_h" stoichiometry="4" constant="false"/>

</listOfProducts>

</reaction>

<reaction id="DAHPS" name="DAHPS" reversible="false" fast="false" compartment="cell">

<listOfReactants>

<speciesReference species="c_e4p" stoichiometry="1" constant="false"/>

<speciesReference species="c_h2o" stoichiometry="1" constant="false"/>

<speciesReference species="c_pep" stoichiometry="1" constant="false"/>

</listOfReactants>

<listOfProducts>

<speciesReference species="c_2dda7p" stoichiometry="1" constant="false"/>

<speciesReference species="c_pi" stoichiometry="1" constant="false"/>

</listOfProducts>

</reaction>

<reaction id="ALATA_L2" name="ALATA_L2" reversible="false" fast="false" compartment="cell">

<listOfReactants>

<speciesReference species="c_ala_L" stoichiometry="1" constant="false"/>

<speciesReference species="c_pydx5p" stoichiometry="1" constant="false"/>

</listOfReactants>

<listOfProducts>

<speciesReference species="c_pyam5p" stoichiometry="1" constant="false"/>

<speciesReference species="c_pyr" stoichiometry="1" constant="false"/>

</listOfProducts>

</reaction>

<reaction id="AIRC3" name="AIRC3" reversible="true" fast="false" compartment="cell">

<listOfReactants>

<speciesReference species="c_5aizc" stoichiometry="1" constant="false"/>

</listOfReactants>

<listOfProducts>

<speciesReference species="c_5caiz" stoichiometry="1" constant="false"/>

</listOfProducts>

</reaction>

<reaction id="FNOR" name="FNOR" reversible="true" fast="false" compartment="cell">

<listOfReactants>

<speciesReference species="c_fdxr_4_2" stoichiometry="1" constant="false"/>

<speciesReference species="c_h" stoichiometry="1" constant="false"/>

<speciesReference species="c_nadp" stoichiometry="1" constant="false"/>

</listOfReactants>

<listOfProducts>

<speciesReference species="c_fdxo_4_2" stoichiometry="1" constant="false"/>

<speciesReference species="c_nadph" stoichiometry="1" constant="false"/>

</listOfProducts>

</reaction>

<reaction id="DB4PS" name="DB4PS" reversible="false" fast="false" compartment="cell">

<listOfReactants>

<speciesReference species="c_ru5p_D" stoichiometry="1" constant="false"/>

</listOfReactants>

<listOfProducts>

<speciesReference species="c_db4p" stoichiometry="1" constant="false"/>

<speciesReference species="c_for" stoichiometry="1" constant="false"/>

<speciesReference species="c_h" stoichiometry="1" constant="false"/>

</listOfProducts>

</reaction>

<reaction id="HISTP" name="HISTP" reversible="false" fast="false" compartment="cell">

<listOfReactants>

<speciesReference species="c_h2o" stoichiometry="1" constant="false"/>

<speciesReference species="c_hisp" stoichiometry="1" constant="false"/>

</listOfReactants>

<listOfProducts>

<speciesReference species="c_histd" stoichiometry="1" constant="false"/>

<speciesReference species="c_pi" stoichiometry="1" constant="false"/>

</listOfProducts>

</reaction>

<reaction id="TMDS" name="TMDS" reversible="false" fast="false" compartment="cell">

<listOfReactants>

<speciesReference species="c_dump" stoichiometry="1" constant="false"/>

<speciesReference species="c_mlthf" stoichiometry="1" constant="false"/>

</listOfReactants>

<listOfProducts>

<speciesReference species="c_dhf" stoichiometry="1" constant="false"/>

<speciesReference species="c_dtmp" stoichiometry="1" constant="false"/>

</listOfProducts>

</reaction>

<reaction id="PHETRS" name="PHETRS" reversible="false" fast="false" compartment="cell">

<listOfReactants>

<speciesReference species="c_atp" stoichiometry="1" constant="false"/>

<speciesReference species="c_phe_L" stoichiometry="1" constant="false"/>

<speciesReference species="c_trnaphe" stoichiometry="1" constant="false"/>

</listOfReactants>

<listOfProducts>

<speciesReference species="c_amp" stoichiometry="1" constant="false"/>

<speciesReference species="c_phetrna" stoichiometry="1" constant="false"/>

<speciesReference species="c_ppi" stoichiometry="1" constant="false"/>

</listOfProducts>

</reaction>

<reaction id="IGPDH" name="IGPDH" reversible="false" fast="false" compartment="cell">

<listOfReactants>

<speciesReference species="c_eig3p" stoichiometry="1" constant="false"/>

<speciesReference species="c_h" stoichiometry="1" constant="false"/>

</listOfReactants>

<listOfProducts>

<speciesReference species="c_h2o" stoichiometry="1" constant="false"/>

<speciesReference species="c_imacp" stoichiometry="1" constant="false"/>

</listOfProducts>

</reaction>

<reaction id="GLYCL" name="GLYCL" reversible="false" fast="false" compartment="cell">

<listOfReactants>

<speciesReference species="c_gly" stoichiometry="1" constant="false"/>

<speciesReference species="c_nad" stoichiometry="1" constant="false"/>

<speciesReference species="c_thf" stoichiometry="1" constant="false"/>

</listOfReactants>

<listOfProducts>

<speciesReference species="c_co2" stoichiometry="1" constant="false"/>

<speciesReference species="c_mlthf" stoichiometry="1" constant="false"/>

<speciesReference species="c_nadh" stoichiometry="1" constant="false"/>

<speciesReference species="c_nh4" stoichiometry="1" constant="false"/>

</listOfProducts>

</reaction>

<reaction id="PDH" name="PDH" reversible="false" fast="false" compartment="cell">

<listOfReactants>

<speciesReference species="c_coa" stoichiometry="1" constant="false"/>

<speciesReference species="c_nad" stoichiometry="1" constant="false"/>

<speciesReference species="c_pyr" stoichiometry="1" constant="false"/>

</listOfReactants>

<listOfProducts>

<speciesReference species="c_accoa" stoichiometry="1" constant="false"/>

<speciesReference species="c_co2" stoichiometry="1" constant="false"/>

<speciesReference species="c_nadh" stoichiometry="1" constant="false"/>

</listOfProducts>

</reaction>

<reaction id="HISTD" name="HISTD" reversible="false" fast="false" compartment="cell">

<listOfReactants>

<speciesReference species="c_h2o" stoichiometry="1" constant="false"/>

<speciesReference species="c_histd" stoichiometry="1" constant="false"/>

<speciesReference species="c_nad" stoichiometry="2" constant="false"/>

</listOfReactants>

<listOfProducts>

<speciesReference species="c_h" stoichiometry="3" constant="false"/>

<speciesReference species="c_his_L" stoichiometry="1" constant="false"/>

<speciesReference species="c_nadh" stoichiometry="2" constant="false"/>

</listOfProducts>

</reaction>

<reaction id="ASP1DC" name="ASP1DC" reversible="false" fast="false" compartment="cell">

<listOfReactants>

<speciesReference species="c_asp_L" stoichiometry="1" constant="false"/>

<speciesReference species="c_h" stoichiometry="1" constant="false"/>

</listOfReactants>

<listOfProducts>

<speciesReference species="c_ala_B" stoichiometry="1" constant="false"/>

<speciesReference species="c_co2" stoichiometry="1" constant="false"/>

</listOfProducts>

</reaction>

<reaction id="OHPBAT" name="OHPBAT" reversible="true" fast="false" compartment="cell">

<listOfReactants>

<speciesReference species="c_glu_L" stoichiometry="1" constant="false"/>

<speciesReference species="c_ohpb" stoichiometry="1" constant="false"/>

</listOfReactants>

<listOfProducts>

<speciesReference species="c_akg" stoichiometry="1" constant="false"/>

<speciesReference species="c_phthr" stoichiometry="1" constant="false"/>

</listOfProducts>

</reaction>

<reaction id="THRTRS" name="THRTRS" reversible="false" fast="false" compartment="cell">

<listOfReactants>

<speciesReference species="c_atp" stoichiometry="1" constant="false"/>

<speciesReference species="c_thr_L" stoichiometry="1" constant="false"/>

<speciesReference species="c_trnathr" stoichiometry="1" constant="false"/>

</listOfReactants>

<listOfProducts>

<speciesReference species="c_amp" stoichiometry="1" constant="false"/>

<speciesReference species="c_ppi" stoichiometry="1" constant="false"/>

<speciesReference species="c_thrtrna" stoichiometry="1" constant="false"/>

</listOfProducts>

</reaction>

<reaction id="SSALx" name="SSALx" reversible="false" fast="false" compartment="cell">

<listOfReactants>

<speciesReference species="c_h2o" stoichiometry="1" constant="false"/>

<speciesReference species="c_nad" stoichiometry="1" constant="false"/>

<speciesReference species="c_sucsal" stoichiometry="1" constant="false"/>

</listOfReactants>

<listOfProducts>

<speciesReference species="c_h" stoichiometry="2" constant="false"/>

<speciesReference species="c_nadh" stoichiometry="1" constant="false"/>

<speciesReference species="c_succ" stoichiometry="1" constant="false"/>

</listOfProducts>

</reaction>

<reaction id="PGAMT" name="PGAMT" reversible="true" fast="false" compartment="cell">

<listOfReactants>

<speciesReference species="c_gam1p" stoichiometry="1" constant="false"/>

</listOfReactants>

<listOfProducts>

<speciesReference species="c_gam6p" stoichiometry="1" constant="false"/>

</listOfProducts>

</reaction>

<reaction id="GTPCII" name="GTPCII" reversible="false" fast="false" compartment="cell">

<listOfReactants>

<speciesReference species="c_gtp" stoichiometry="1" constant="false"/>

<speciesReference species="c_h2o" stoichiometry="3" constant="false"/>

</listOfReactants>

<listOfProducts>

<speciesReference species="c_25dhpp" stoichiometry="1" constant="false"/>

<speciesReference species="c_for" stoichiometry="1" constant="false"/>

<speciesReference species="c_h" stoichiometry="2" constant="false"/>

<speciesReference species="c_ppi" stoichiometry="1" constant="false"/>

</listOfProducts>

</reaction>

<reaction id="SERD_L" name="SERD_L" reversible="false" fast="false" compartment="cell">

<listOfReactants>

<speciesReference species="c_ser_L" stoichiometry="1" constant="false"/>

</listOfReactants>

<listOfProducts>

<speciesReference species="c_nh4" stoichiometry="1" constant="false"/>

<speciesReference species="c_pyr" stoichiometry="1" constant="false"/>

</listOfProducts>

</reaction>

<reaction id="PRAGS" name="PRAGS" reversible="false" fast="false" compartment="cell">

<listOfReactants>

<speciesReference species="c_atp" stoichiometry="1" constant="false"/>

<speciesReference species="c_gly" stoichiometry="1" constant="false"/>

<speciesReference species="c_pram" stoichiometry="1" constant="false"/>

</listOfReactants>

<listOfProducts>

<speciesReference species="c_adp" stoichiometry="1" constant="false"/>

<speciesReference species="c_gar" stoichiometry="1" constant="false"/>

<speciesReference species="c_h" stoichiometry="1" constant="false"/>

<speciesReference species="c_pi" stoichiometry="1" constant="false"/>

</listOfProducts>

</reaction>

<reaction id="ACBIPGT" name="ACBIPGT" reversible="false" fast="false" compartment="cell">

<listOfReactants>

<speciesReference species="c_adcobap" stoichiometry="1" constant="false"/>

<speciesReference species="c_gtp" stoichiometry="1" constant="false"/>

<speciesReference species="c_h" stoichiometry="1" constant="false"/>

</listOfReactants>

<listOfProducts>

<speciesReference species="c_adgcoba" stoichiometry="1" constant="false"/>

<speciesReference species="c_ppi" stoichiometry="1" constant="false"/>

</listOfProducts>

</reaction>

<reaction id="PASYN_SO_Aerobic" name="PASYN_SO_Aerobic" reversible="false" fast="false" compartment="cell">

<listOfReactants>

<speciesReference species="c_fa11ACP" stoichiometry="0.13" constant="false"/>

<speciesReference species="c_fa13ACP" stoichiometry="0.07" constant="false"/>

<speciesReference species="c_fa1ACP" stoichiometry="0.12" constant="false"/>

<speciesReference species="c_fa3ACP" stoichiometry="0.33" constant="false"/>

<speciesReference species="c_fa6ACP" stoichiometry="0.15" constant="false"/>

<speciesReference species="c_glyc3p" stoichiometry="1" constant="false"/>

<speciesReference species="c_hdeACP" stoichiometry="0.38" constant="false"/>

<speciesReference species="c_hpdACP" stoichiometry="0.03" constant="false"/>

<speciesReference species="c_hpdeACP" stoichiometry="0.25" constant="false"/>

<speciesReference species="c_ocdACP" stoichiometry="0.02" constant="false"/>

<speciesReference species="c_octeACP" stoichiometry="0.25" constant="false"/>

<speciesReference species="c_palmACP" stoichiometry="0.13" constant="false"/>

<speciesReference species="c_pdACP" stoichiometry="0.02" constant="false"/>

<speciesReference species="c_pdeACP" stoichiometry="0.12" constant="false"/>

</listOfReactants>

<listOfProducts>

<speciesReference species="c_12dag3p" stoichiometry="1" constant="false"/>

<speciesReference species="c_ACP" stoichiometry="2" constant="false"/>

</listOfProducts>

</reaction>

<reaction id="TDPDRE" name="TDPDRE" reversible="true" fast="false" compartment="cell">

<listOfReactants>

<speciesReference species="c_dtdpddg" stoichiometry="1" constant="false"/>

</listOfReactants>

<listOfProducts>

<speciesReference species="c_dtdpddm" stoichiometry="1" constant="false"/>

</listOfProducts>

</reaction>

<reaction id="KARA2i" name="KARA2i" reversible="false" fast="false" compartment="cell">

<listOfReactants>

<speciesReference species="c_2ahbut" stoichiometry="1" constant="false"/>

<speciesReference species="c_h" stoichiometry="1" constant="false"/>

<speciesReference species="c_nadph" stoichiometry="1" constant="false"/>

</listOfReactants>

<listOfProducts>

<speciesReference species="c_23dhmp" stoichiometry="1" constant="false"/>

<speciesReference species="c_nadp" stoichiometry="1" constant="false"/>

</listOfProducts>

</reaction>

<reaction id="AIRC2" name="AIRC2" reversible="false" fast="false" compartment="cell">

<listOfReactants>

<speciesReference species="c_air" stoichiometry="1" constant="false"/>

<speciesReference species="c_atp" stoichiometry="1" constant="false"/>

<speciesReference species="c_hco3" stoichiometry="1" constant="false"/>

</listOfReactants>

<listOfProducts>

<speciesReference species="c_5caiz" stoichiometry="1" constant="false"/>

<speciesReference species="c_adp" stoichiometry="1" constant="false"/>

<speciesReference species="c_h" stoichiometry="1" constant="false"/>

<speciesReference species="c_pi" stoichiometry="1" constant="false"/>

</listOfProducts>

</reaction>

<reaction id="FUMACA" name="FUMACA" reversible="false" fast="false" compartment="cell">

<listOfReactants>

<speciesReference species="c_4fumacac" stoichiometry="1" constant="false"/>

<speciesReference species="c_h2o" stoichiometry="1" constant="false"/>

</listOfReactants>

<listOfProducts>

<speciesReference species="c_acac" stoichiometry="1" constant="false"/>

<speciesReference species="c_fum" stoichiometry="1" constant="false"/>

<speciesReference species="c_h" stoichiometry="1" constant="false"/>

</listOfProducts>

</reaction>

<reaction id="UPPRT" name="UPPRT" reversible="false" fast="false" compartment="cell">

<listOfReactants>

<speciesReference species="c_prpp" stoichiometry="1" constant="false"/>

<speciesReference species="c_ura" stoichiometry="1" constant="false"/>

</listOfReactants>

<listOfProducts>

<speciesReference species="c_ppi" stoichiometry="1" constant="false"/>

<speciesReference species="c_ump" stoichiometry="1" constant="false"/>

</listOfProducts>

</reaction>

<reaction id="HEMEOS" name="HEMEOS" reversible="false" fast="false" compartment="cell">

<listOfReactants>

<speciesReference species="c_frdp" stoichiometry="1" constant="false"/>

<speciesReference species="c_h2o" stoichiometry="1" constant="false"/>

<speciesReference species="c_pheme" stoichiometry="1" constant="false"/>

</listOfReactants>

<listOfProducts>

<speciesReference species="c_h" stoichiometry="1" constant="false"/>

<speciesReference species="c_hemeO" stoichiometry="1" constant="false"/>

<speciesReference species="c_ppi" stoichiometry="1" constant="false"/>

</listOfProducts>

</reaction>

<reaction id="LDH_Dir" name="LDH_Dir" reversible="false" fast="false" compartment="cell">

<listOfReactants>

<speciesReference species="c_h" stoichiometry="1" constant="false"/>

<speciesReference species="c_nadh" stoichiometry="1" constant="false"/>

<speciesReference species="c_pyr" stoichiometry="1" constant="false"/>

</listOfReactants>

<listOfProducts>

<speciesReference species="c_lac_D" stoichiometry="1" constant="false"/>

<speciesReference species="c_nad" stoichiometry="1" constant="false"/>

</listOfProducts>

</reaction>

<reaction id="CYTD" name="CYTD" reversible="false" fast="false" compartment="cell">

<listOfReactants>

<speciesReference species="c_cytd" stoichiometry="1" constant="false"/>

<speciesReference species="c_h" stoichiometry="1" constant="false"/>

<speciesReference species="c_h2o" stoichiometry="1" constant="false"/>

</listOfReactants>

<listOfProducts>

<speciesReference species="c_nh4" stoichiometry="1" constant="false"/>

<speciesReference species="c_uri" stoichiometry="1" constant="false"/>

</listOfProducts>

</reaction>

<reaction id="ADNCYC" name="ADNCYC" reversible="false" fast="false" compartment="cell">

<listOfReactants>

<speciesReference species="c_atp" stoichiometry="1" constant="false"/>

</listOfReactants>

<listOfProducts>

<speciesReference species="c_camp" stoichiometry="1" constant="false"/>

<speciesReference species="c_ppi" stoichiometry="1" constant="false"/>

</listOfProducts>

</reaction>

<reaction id="NDPK8" name="NDPK8" reversible="true" fast="false" compartment="cell">

<listOfReactants>

<speciesReference species="c_atp" stoichiometry="1" constant="false"/>

<speciesReference species="c_dadp" stoichiometry="1" constant="false"/>

</listOfReactants>

<listOfProducts>

<speciesReference species="c_adp" stoichiometry="1" constant="false"/>

<speciesReference species="c_datp" stoichiometry="1" constant="false"/>

</listOfProducts>

</reaction>

<reaction id="RPE" name="RPE" reversible="true" fast="false" compartment="cell">

<listOfReactants>

<speciesReference species="c_ru5p_D" stoichiometry="1" constant="false"/>

</listOfReactants>

<listOfProducts>

<speciesReference species="c_xu5p_D" stoichiometry="1" constant="false"/>

</listOfProducts>

</reaction>

<reaction id="NDPK2" name="NDPK2" reversible="true" fast="false" compartment="cell">

<listOfReactants>

<speciesReference species="c_atp" stoichiometry="1" constant="false"/>

<speciesReference species="c_udp" stoichiometry="1" constant="false"/>

</listOfReactants>

<listOfProducts>

<speciesReference species="c_adp" stoichiometry="1" constant="false"/>

<speciesReference species="c_utp" stoichiometry="1" constant="false"/>

</listOfProducts>

</reaction>

<reaction id="NDPK3" name="NDPK3" reversible="true" fast="false" compartment="cell">

<listOfReactants>

<speciesReference species="c_atp" stoichiometry="1" constant="false"/>

<speciesReference species="c_cdp" stoichiometry="1" constant="false"/>

</listOfReactants>

<listOfProducts>

<speciesReference species="c_adp" stoichiometry="1" constant="false"/>

<speciesReference species="c_ctp" stoichiometry="1" constant="false"/>

</listOfProducts>

</reaction>

<reaction id="NDPK1" name="NDPK1" reversible="true" fast="false" compartment="cell">

<listOfReactants>

<speciesReference species="c_atp" stoichiometry="1" constant="false"/>

<speciesReference species="c_gdp" stoichiometry="1" constant="false"/>

</listOfReactants>

<listOfProducts>

<speciesReference species="c_adp" stoichiometry="1" constant="false"/>

<speciesReference species="c_gtp" stoichiometry="1" constant="false"/>

</listOfProducts>

</reaction>

<reaction id="NDPK6" name="NDPK6" reversible="true" fast="false" compartment="cell">

<listOfReactants>

<speciesReference species="c_atp" stoichiometry="1" constant="false"/>

<speciesReference species="c_dudp" stoichiometry="1" constant="false"/>

</listOfReactants>

<listOfProducts>

<speciesReference species="c_adp" stoichiometry="1" constant="false"/>

<speciesReference species="c_dutp" stoichiometry="1" constant="false"/>

</listOfProducts>

</reaction>

<reaction id="NDPK7" name="NDPK7" reversible="true" fast="false" compartment="cell">

<listOfReactants>

<speciesReference species="c_atp" stoichiometry="1" constant="false"/>

<speciesReference species="c_dcdp" stoichiometry="1" constant="false"/>

</listOfReactants>

<listOfProducts>

<speciesReference species="c_adp" stoichiometry="1" constant="false"/>

<speciesReference species="c_dctp" stoichiometry="1" constant="false"/>

</listOfProducts>

</reaction>

<reaction id="NDPK4" name="NDPK4" reversible="true" fast="false" compartment="cell">

<listOfReactants>

<speciesReference species="c_atp" stoichiometry="1" constant="false"/>

<speciesReference species="c_dtdp" stoichiometry="1" constant="false"/>

</listOfReactants>

<listOfProducts>

<speciesReference species="c_adp" stoichiometry="1" constant="false"/>

<speciesReference species="c_dttp" stoichiometry="1" constant="false"/>

</listOfProducts>

</reaction>

<reaction id="NDPK5" name="NDPK5" reversible="true" fast="false" compartment="cell">

<listOfReactants>

<speciesReference species="c_atp" stoichiometry="1" constant="false"/>

<speciesReference species="c_dgdp" stoichiometry="1" constant="false"/>

</listOfReactants>

<listOfProducts>

<speciesReference species="c_adp" stoichiometry="1" constant="false"/>

<speciesReference species="c_dgtp" stoichiometry="1" constant="false"/>

</listOfProducts>

</reaction>

<reaction id="SUCD7" name="SUCD7" reversible="false" fast="false" compartment="cell">

<listOfReactants>

<speciesReference species="c_succ" stoichiometry="1" constant="false"/>

<speciesReference species="c_ubq8" stoichiometry="1" constant="false"/>

</listOfReactants>

<listOfProducts>

<speciesReference species="c_fum" stoichiometry="1" constant="false"/>

<speciesReference species="c_ubq8h2" stoichiometry="1" constant="false"/>

</listOfProducts>

</reaction>

<reaction id="MCOATA" name="MCOATA" reversible="true" fast="false" compartment="cell">

<listOfReactants>

<speciesReference species="c_ACP" stoichiometry="1" constant="false"/>

<speciesReference species="c_malcoa" stoichiometry="1" constant="false"/>

</listOfReactants>

<listOfProducts>

<speciesReference species="c_coa" stoichiometry="1" constant="false"/>

<speciesReference species="c_malACP" stoichiometry="1" constant="false"/>

</listOfProducts>

</reaction>

<reaction id="G3PD2" name="G3PD2" reversible="true" fast="false" compartment="cell">

<listOfReactants>

<speciesReference species="c_glyc3p" stoichiometry="1" constant="false"/>

<speciesReference species="c_nadp" stoichiometry="1" constant="false"/>

</listOfReactants>

<listOfProducts>

<speciesReference species="c_dhap" stoichiometry="1" constant="false"/>

<speciesReference species="c_h" stoichiometry="1" constant="false"/>

<speciesReference species="c_nadph" stoichiometry="1" constant="false"/>

</listOfProducts>

</reaction>

<reaction id="DTMPK" name="DTMPK" reversible="true" fast="false" compartment="cell">

<listOfReactants>

<speciesReference species="c_atp" stoichiometry="1" constant="false"/>

<speciesReference species="c_dtmp" stoichiometry="1" constant="false"/>

</listOfReactants>

<listOfProducts>

<speciesReference species="c_adp" stoichiometry="1" constant="false"/>

<speciesReference species="c_dtdp" stoichiometry="1" constant="false"/>

</listOfProducts>

</reaction>

<reaction id="AACPS8" name="AACPS8" reversible="false" fast="false" compartment="cell">

<listOfReactants>

<speciesReference species="c_ACP" stoichiometry="1" constant="false"/>

<speciesReference species="c_atp" stoichiometry="1" constant="false"/>

<speciesReference species="c_ocdca" stoichiometry="1" constant="false"/>

</listOfReactants>

<listOfProducts>

<speciesReference species="c_amp" stoichiometry="1" constant="false"/>

<speciesReference species="c_ocdACP" stoichiometry="1" constant="false"/>

<speciesReference species="c_ppi" stoichiometry="1" constant="false"/>

</listOfProducts>

</reaction>

<reaction id="AACPS9" name="AACPS9" reversible="false" fast="false" compartment="cell">

<listOfReactants>

<speciesReference species="c_ACP" stoichiometry="1" constant="false"/>

<speciesReference species="c_atp" stoichiometry="1" constant="false"/>

<speciesReference species="c_fa13" stoichiometry="1" constant="false"/>

</listOfReactants>

<listOfProducts>

<speciesReference species="c_amp" stoichiometry="1" constant="false"/>

<speciesReference species="c_fa13ACP" stoichiometry="1" constant="false"/>

<speciesReference species="c_ppi" stoichiometry="1" constant="false"/>

</listOfProducts>

</reaction>

<reaction id="G3PD4" name="G3PD4" reversible="false" fast="false" compartment="cell">

<listOfReactants>

<speciesReference species="c_glyc3p" stoichiometry="1" constant="false"/>

<speciesReference species="c_mqn7" stoichiometry="1" constant="false"/>

</listOfReactants>

<listOfProducts>

<speciesReference species="c_dhap" stoichiometry="1" constant="false"/>

<speciesReference species="c_mql7" stoichiometry="1" constant="false"/>

</listOfProducts>

</reaction>

<reaction id="GTHRD" name="GTHRD" reversible="true" fast="false" compartment="cell">

<listOfReactants>

<speciesReference species="c_gthrd" stoichiometry="2" constant="false"/>

<speciesReference species="c_nadp" stoichiometry="1" constant="false"/>

</listOfReactants>

<listOfProducts>

<speciesReference species="c_gthox" stoichiometry="1" constant="false"/>

<speciesReference species="c_h" stoichiometry="1" constant="false"/>

<speciesReference species="c_nadph" stoichiometry="1" constant="false"/>

</listOfProducts>

</reaction>

<reaction id="BTS2" name="BTS2" reversible="true" fast="false" compartment="cell">

<listOfReactants>

<speciesReference species="c_cys_L" stoichiometry="1" constant="false"/>

<speciesReference species="c_dtbt" stoichiometry="1" constant="false"/>

</listOfReactants>

<listOfProducts>

<speciesReference species="c_ala_L" stoichiometry="1" constant="false"/>

<speciesReference species="c_btn" stoichiometry="1" constant="false"/>

<speciesReference species="c_h" stoichiometry="2" constant="false"/>

</listOfProducts>

</reaction>

<reaction id="AACPS6" name="AACPS6" reversible="false" fast="false" compartment="cell">

<listOfReactants>

<speciesReference species="c_ACP" stoichiometry="1" constant="false"/>

<speciesReference species="c_atp" stoichiometry="1" constant="false"/>

<speciesReference species="c_ptdca" stoichiometry="1" constant="false"/>

</listOfReactants>

<listOfProducts>

<speciesReference species="c_amp" stoichiometry="1" constant="false"/>

<speciesReference species="c_pdACP" stoichiometry="1" constant="false"/>

<speciesReference species="c_ppi" stoichiometry="1" constant="false"/>

</listOfProducts>

</reaction>

<reaction id="G3PD8" name="G3PD8" reversible="false" fast="false" compartment="cell">

<listOfReactants>

<speciesReference species="c_glyc3p" stoichiometry="1" constant="false"/>

<speciesReference species="c_mmqn7" stoichiometry="1" constant="false"/>

</listOfReactants>

<listOfProducts>

<speciesReference species="c_dhap" stoichiometry="1" constant="false"/>

<speciesReference species="c_mmql7" stoichiometry="1" constant="false"/>

</listOfProducts>

</reaction>

<reaction id="AACPS3" name="AACPS3" reversible="false" fast="false" compartment="cell">

<listOfReactants>

<speciesReference species="c_ACP" stoichiometry="1" constant="false"/>

<speciesReference species="c_atp" stoichiometry="1" constant="false"/>

<speciesReference species="c_hdca" stoichiometry="1" constant="false"/>

</listOfReactants>

<listOfProducts>

<speciesReference species="c_amp" stoichiometry="1" constant="false"/>

<speciesReference species="c_palmACP" stoichiometry="1" constant="false"/>

<speciesReference species="c_ppi" stoichiometry="1" constant="false"/>

</listOfProducts>

</reaction>

<reaction id="ASPTA1" name="ASPTA1" reversible="true" fast="false" compartment="cell">

<listOfReactants>

<speciesReference species="c_akg" stoichiometry="1" constant="false"/>

<speciesReference species="c_asp_L" stoichiometry="1" constant="false"/>

</listOfReactants>

<listOfProducts>

<speciesReference species="c_glu_L" stoichiometry="1" constant="false"/>

<speciesReference species="c_oaa" stoichiometry="1" constant="false"/>

</listOfProducts>

</reaction>

<reaction id="ASPTA4" name="ASPTA4" reversible="true" fast="false" compartment="cell">

<listOfReactants>

<speciesReference species="c_akg" stoichiometry="1" constant="false"/>

<speciesReference species="c_cys_L" stoichiometry="1" constant="false"/>

</listOfReactants>

<listOfProducts>

<speciesReference species="c_glu_L" stoichiometry="1" constant="false"/>

<speciesReference species="c_mercppyr" stoichiometry="1" constant="false"/>

</listOfProducts>

</reaction>

<reaction id="ANS1" name="ANS1" reversible="false" fast="false" compartment="cell">

<listOfReactants>

<speciesReference species="c_chor" stoichiometry="1" constant="false"/>

<speciesReference species="c_gln_L" stoichiometry="1" constant="false"/>

</listOfReactants>

<listOfProducts>

<speciesReference species="c_anth" stoichiometry="1" constant="false"/>

<speciesReference species="c_glu_L" stoichiometry="1" constant="false"/>

<speciesReference species="c_h" stoichiometry="1" constant="false"/>

<speciesReference species="c_pyr" stoichiometry="1" constant="false"/>

</listOfProducts>

</reaction>

<reaction id="SHCHD2" name="SHCHD2" reversible="false" fast="false" compartment="cell">

<listOfReactants>

<speciesReference species="c_nad" stoichiometry="1" constant="false"/>

<speciesReference species="c_shcl" stoichiometry="1" constant="false"/>

</listOfReactants>

<listOfProducts>

<speciesReference species="c_h" stoichiometry="1" constant="false"/>

<speciesReference species="c_nadh" stoichiometry="1" constant="false"/>

<speciesReference species="c_srch" stoichiometry="1" constant="false"/>

</listOfProducts>

</reaction>

<reaction id="ACCOAL" name="ACCOAL" reversible="false" fast="false" compartment="cell">

<listOfReactants>

<speciesReference species="c_atp" stoichiometry="1" constant="false"/>

<speciesReference species="c_coa" stoichiometry="1" constant="false"/>

<speciesReference species="c_ppa" stoichiometry="1" constant="false"/>

</listOfReactants>

<listOfProducts>

<speciesReference species="c_adp" stoichiometry="1" constant="false"/>

<speciesReference species="c_pi" stoichiometry="1" constant="false"/>

<speciesReference species="c_ppcoa" stoichiometry="1" constant="false"/>

</listOfProducts>

</reaction>

<reaction id="GLYD" name="GLYD" reversible="true" fast="false" compartment="cell">

<listOfReactants>

<speciesReference species="c_h" stoichiometry="1" constant="false"/>

<speciesReference species="c_hpyr" stoichiometry="1" constant="false"/>

<speciesReference species="c_nadh" stoichiometry="1" constant="false"/>

</listOfReactants>

<listOfProducts>

<speciesReference species="c_glyc_R" stoichiometry="1" constant="false"/>

<speciesReference species="c_nad" stoichiometry="1" constant="false"/>

</listOfProducts>

</reaction>

<reaction id="GLYK" name="GLYK" reversible="false" fast="false" compartment="cell">

<listOfReactants>

<speciesReference species="c_atp" stoichiometry="1" constant="false"/>

<speciesReference species="c_glyc" stoichiometry="1" constant="false"/>

</listOfReactants>

<listOfProducts>

<speciesReference species="c_adp" stoichiometry="1" constant="false"/>

<speciesReference species="c_glyc3p" stoichiometry="1" constant="false"/>

<speciesReference species="c_h" stoichiometry="1" constant="false"/>

</listOfProducts>

</reaction>

<reaction id="ACCOAC" name="ACCOAC" reversible="false" fast="false" compartment="cell">

<listOfReactants>

<speciesReference species="c_accoa" stoichiometry="1" constant="false"/>

<speciesReference species="c_atp" stoichiometry="1" constant="false"/>

<speciesReference species="c_hco3" stoichiometry="1" constant="false"/>

</listOfReactants>

<listOfProducts>

<speciesReference species="c_adp" stoichiometry="1" constant="false"/>

<speciesReference species="c_h" stoichiometry="1" constant="false"/>

<speciesReference species="c_malcoa" stoichiometry="1" constant="false"/>

<speciesReference species="c_pi" stoichiometry="1" constant="false"/>

</listOfProducts>

</reaction>

<reaction id="PAP" name="PAP" reversible="false" fast="false" compartment="cell">

<listOfReactants>

<speciesReference species="c_12dag3p" stoichiometry="1" constant="false"/>

<speciesReference species="c_h2o" stoichiometry="1" constant="false"/>

</listOfReactants>

<listOfProducts>

<speciesReference species="c_12dgr" stoichiometry="1" constant="false"/>

<speciesReference species="c_pi" stoichiometry="1" constant="false"/>

</listOfProducts>

</reaction>

<reaction id="NMNAT" name="NMNAT" reversible="false" fast="false" compartment="cell">

<listOfReactants>

<speciesReference species="c_atp" stoichiometry="1" constant="false"/>

<speciesReference species="c_h" stoichiometry="1" constant="false"/>

<speciesReference species="c_nmn" stoichiometry="1" constant="false"/>

</listOfReactants>

<listOfProducts>

<speciesReference species="c_nad" stoichiometry="1" constant="false"/>

<speciesReference species="c_ppi" stoichiometry="1" constant="false"/>

</listOfProducts>

</reaction>

<reaction id="TDPDRR" name="TDPDRR" reversible="true" fast="false" compartment="cell">

<listOfReactants>

<speciesReference species="c_dtdp6dm" stoichiometry="1" constant="false"/>

<speciesReference species="c_nadp" stoichiometry="1" constant="false"/>

</listOfReactants>

<listOfProducts>

<speciesReference species="c_dtdpddm" stoichiometry="1" constant="false"/>

<speciesReference species="c_h" stoichiometry="1" constant="false"/>

<speciesReference species="c_nadph" stoichiometry="1" constant="false"/>

</listOfProducts>

</reaction>

<reaction id="DKMPPD" name="DKMPPD" reversible="false" fast="false" compartment="cell">

<listOfReactants>

<speciesReference species="c_dkmpp" stoichiometry="1" constant="false"/>

<speciesReference species="c_h2o" stoichiometry="1" constant="false"/>

<speciesReference species="c_o2" stoichiometry="1" constant="false"/>

</listOfReactants>

<listOfProducts>

<speciesReference species="c_2kmb" stoichiometry="1" constant="false"/>

<speciesReference species="c_for" stoichiometry="1" constant="false"/>

<speciesReference species="c_h" stoichiometry="2" constant="false"/>

<speciesReference species="c_pi" stoichiometry="1" constant="false"/>

</listOfProducts>

</reaction>

<reaction id="MOAT3" name="MOAT3" reversible="false" fast="false" compartment="cell">

<listOfReactants>

<speciesReference species="c_ckdo8n" stoichiometry="1" constant="false"/>

<speciesReference species="c_lipidA" stoichiometry="1" constant="false"/>

</listOfReactants>

<listOfProducts>

<speciesReference species="c_cmp" stoichiometry="1" constant="false"/>

<speciesReference species="c_h" stoichiometry="1" constant="false"/>

<speciesReference species="c_kdo8nlipid4" stoichiometry="1" constant="false"/>

</listOfProducts>

</reaction>

<reaction id="C50SN" name="C50SN" reversible="false" fast="false" compartment="cell">

<listOfReactants>

<speciesReference species="c_h" stoichiometry="3" constant="false"/>

<speciesReference species="c_malACP" stoichiometry="1" constant="false"/>

<speciesReference species="c_nadph" stoichiometry="2" constant="false"/>

<speciesReference species="c_ppcoa" stoichiometry="1" constant="false"/>

</listOfReactants>

<listOfProducts>

<speciesReference species="c_co2" stoichiometry="1" constant="false"/>

<speciesReference species="c_coa" stoichiometry="1" constant="false"/>

<speciesReference species="c_fa14ACP" stoichiometry="1" constant="false"/>

<speciesReference species="c_h2o" stoichiometry="1" constant="false"/>

<speciesReference species="c_nadp" stoichiometry="2" constant="false"/>

</listOfProducts>

</reaction>

<reaction id="UPPDC1" name="UPPDC1" reversible="false" fast="false" compartment="cell">

<listOfReactants>

<speciesReference species="c_h" stoichiometry="4" constant="false"/>

<speciesReference species="c_uppg3" stoichiometry="1" constant="false"/>

</listOfReactants>

<listOfProducts>

<speciesReference species="c_co2" stoichiometry="4" constant="false"/>

<speciesReference species="c_cpppg3" stoichiometry="1" constant="false"/>

</listOfProducts>

</reaction>

<reaction id="SHK3D" name="SHK3D" reversible="true" fast="false" compartment="cell">

<listOfReactants>

<speciesReference species="c_3dhsk" stoichiometry="1" constant="false"/>

<speciesReference species="c_h" stoichiometry="1" constant="false"/>

<speciesReference species="c_nadph" stoichiometry="1" constant="false"/>

</listOfReactants>

<listOfProducts>

<speciesReference species="c_nadp" stoichiometry="1" constant="false"/>

<speciesReference species="c_skm" stoichiometry="1" constant="false"/>

</listOfProducts>

</reaction>

<reaction id="UDCPDPS" name="UDCPDPS" reversible="false" fast="false" compartment="cell">

<listOfReactants>

<speciesReference species="c_frdp" stoichiometry="1" constant="false"/>

<speciesReference species="c_ipdp" stoichiometry="8" constant="false"/>

</listOfReactants>

<listOfProducts>

<speciesReference species="c_ppi" stoichiometry="8" constant="false"/>

<speciesReference species="c_udcpdp" stoichiometry="1" constant="false"/>

</listOfProducts>

</reaction>

<reaction id="PERD" name="PERD" reversible="true" fast="false" compartment="cell">

<listOfReactants>

<speciesReference species="c_4per" stoichiometry="1" constant="false"/>

<speciesReference species="c_nad" stoichiometry="1" constant="false"/>

</listOfReactants>

<listOfProducts>

<speciesReference species="c_h" stoichiometry="1" constant="false"/>

<speciesReference species="c_nadh" stoichiometry="1" constant="false"/>

<speciesReference species="c_ohpb" stoichiometry="1" constant="false"/>

</listOfProducts>

</reaction>

<reaction id="HSK" name="HSK" reversible="false" fast="false" compartment="cell">

<listOfReactants>

<speciesReference species="c_atp" stoichiometry="1" constant="false"/>

<speciesReference species="c_hom_L" stoichiometry="1" constant="false"/>

</listOfReactants>

<listOfProducts>

<speciesReference species="c_adp" stoichiometry="1" constant="false"/>

<speciesReference species="c_h" stoichiometry="1" constant="false"/>

<speciesReference species="c_phom" stoichiometry="1" constant="false"/>

</listOfProducts>

</reaction>

<reaction id="THRD" name="THRD" reversible="false" fast="false" compartment="cell">

<listOfReactants>

<speciesReference species="c_nad" stoichiometry="1" constant="false"/>

<speciesReference species="c_thr_L" stoichiometry="1" constant="false"/>

</listOfReactants>

<listOfProducts>

<speciesReference species="c_2aobut" stoichiometry="1" constant="false"/>

<speciesReference species="c_h" stoichiometry="1" constant="false"/>

<speciesReference species="c_nadh" stoichiometry="1" constant="false"/>

</listOfProducts>

</reaction>

<reaction id="IPDPS" name="IPDPS" reversible="false" fast="false" compartment="cell">

<listOfReactants>

<speciesReference species="c_h" stoichiometry="1" constant="false"/>

<speciesReference species="c_h2mb4p" stoichiometry="1" constant="false"/>

<speciesReference species="c_nadh" stoichiometry="1" constant="false"/>

</listOfReactants>

<listOfProducts>

<speciesReference species="c_h2o" stoichiometry="1" constant="false"/>

<speciesReference species="c_ipdp" stoichiometry="1" constant="false"/>

<speciesReference species="c_nad" stoichiometry="1" constant="false"/>

</listOfProducts>

</reaction>

<reaction id="ACOTA" name="ACOTA" reversible="true" fast="false" compartment="cell">

<listOfReactants>

<speciesReference species="c_acorn" stoichiometry="1" constant="false"/>

<speciesReference species="c_akg" stoichiometry="1" constant="false"/>

</listOfReactants>

<listOfProducts>

<speciesReference species="c_acg5sa" stoichiometry="1" constant="false"/>

<speciesReference species="c_glu_L" stoichiometry="1" constant="false"/>

</listOfProducts>

</reaction>

<reaction id="RBFSb" name="RBFSb" reversible="false" fast="false" compartment="cell">

<listOfReactants>

<speciesReference species="c_dmlz" stoichiometry="2" constant="false"/>

</listOfReactants>

<listOfProducts>

<speciesReference species="c_4r5au" stoichiometry="1" constant="false"/>

<speciesReference species="c_ribflv" stoichiometry="1" constant="false"/>

</listOfProducts>

</reaction>

<reaction id="RBFSa" name="RBFSa" reversible="false" fast="false" compartment="cell">

<listOfReactants>

<speciesReference species="c_4r5au" stoichiometry="1" constant="false"/>

<speciesReference species="c_db4p" stoichiometry="1" constant="false"/>

</listOfReactants>

<listOfProducts>

<speciesReference species="c_dmlz" stoichiometry="1" constant="false"/>

<speciesReference species="c_h2o" stoichiometry="2" constant="false"/>

<speciesReference species="c_pi" stoichiometry="1" constant="false"/>

</listOfProducts>

</reaction>

<reaction id="MTHFD" name="MTHFD" reversible="true" fast="false" compartment="cell">

<listOfReactants>

<speciesReference species="c_mlthf" stoichiometry="1" constant="false"/>

<speciesReference species="c_nadp" stoichiometry="1" constant="false"/>

</listOfReactants>

<listOfProducts>

<speciesReference species="c_methf" stoichiometry="1" constant="false"/>

<speciesReference species="c_nadph" stoichiometry="1" constant="false"/>

</listOfProducts>

</reaction>

<reaction id="ALCD2x" name="ALCD2x" reversible="true" fast="false" compartment="cell">

<listOfReactants>

<speciesReference species="c_etoh" stoichiometry="1" constant="false"/>

<speciesReference species="c_nad" stoichiometry="1" constant="false"/>

</listOfReactants>

<listOfProducts>

<speciesReference species="c_acald" stoichiometry="1" constant="false"/>

<speciesReference species="c_h" stoichiometry="1" constant="false"/>

<speciesReference species="c_nadh" stoichiometry="1" constant="false"/>

</listOfProducts>

</reaction>

<reaction id="HSTPT" name="HSTPT" reversible="false" fast="false" compartment="cell">

<listOfReactants>

<speciesReference species="c_glu_L" stoichiometry="1" constant="false"/>

<speciesReference species="c_imacp" stoichiometry="1" constant="false"/>

</listOfReactants>

<listOfProducts>

<speciesReference species="c_akg" stoichiometry="1" constant="false"/>

<speciesReference species="c_h" stoichiometry="1" constant="false"/>

<speciesReference species="c_hisp" stoichiometry="1" constant="false"/>

</listOfProducts>

</reaction>

<reaction id="THRS" name="THRS" reversible="false" fast="false" compartment="cell">

<listOfReactants>

<speciesReference species="c_h2o" stoichiometry="1" constant="false"/>

<speciesReference species="c_phom" stoichiometry="1" constant="false"/>

</listOfReactants>

<listOfProducts>

<speciesReference species="c_pi" stoichiometry="1" constant="false"/>

<speciesReference species="c_thr_L" stoichiometry="1" constant="false"/>

</listOfProducts>

</reaction>

<reaction id="GAPD" name="GAPD" reversible="true" fast="false" compartment="cell">

<listOfReactants>

<speciesReference species="c_g3p" stoichiometry="1" constant="false"/>

<speciesReference species="c_nad" stoichiometry="1" constant="false"/>

<speciesReference species="c_pi" stoichiometry="1" constant="false"/>

</listOfReactants>

<listOfProducts>

<speciesReference species="c_13dpg" stoichiometry="1" constant="false"/>

<speciesReference species="c_h" stoichiometry="1" constant="false"/>

<speciesReference species="c_nadh" stoichiometry="1" constant="false"/>

</listOfProducts>

</reaction>

<reaction id="TRPS3" name="TRPS3" reversible="false" fast="false" compartment="cell">

<listOfReactants>

<speciesReference species="c_3ig3p" stoichiometry="1" constant="false"/>

</listOfReactants>

<listOfProducts>

<speciesReference species="c_g3p" stoichiometry="1" constant="false"/>

<speciesReference species="c_indole" stoichiometry="1" constant="false"/>

</listOfProducts>

</reaction>

<reaction id="UAG4E" name="UAG4E" reversible="false" fast="false" compartment="cell">

<listOfReactants>

<speciesReference species="c_uacgam" stoichiometry="1" constant="false"/>

</listOfReactants>

<listOfProducts>

<speciesReference species="c_uacgala" stoichiometry="1" constant="false"/>

</listOfProducts>

</reaction>

<reaction id="QULNS" name="QULNS" reversible="false" fast="false" compartment="cell">

<listOfReactants>

<speciesReference species="c_dhap" stoichiometry="1" constant="false"/>

<speciesReference species="c_iasp" stoichiometry="1" constant="false"/>

</listOfReactants>

<listOfProducts>

<speciesReference species="c_h2o" stoichiometry="2" constant="false"/>

<speciesReference species="c_pi" stoichiometry="1" constant="false"/>

<speciesReference species="c_quln" stoichiometry="1" constant="false"/>

</listOfProducts>

</reaction>

<reaction id="DBTSr" name="DBTSr" reversible="true" fast="false" compartment="cell">

<listOfReactants>

<speciesReference species="c_atp" stoichiometry="1" constant="false"/>

<speciesReference species="c_co2" stoichiometry="1" constant="false"/>

<speciesReference species="c_dann" stoichiometry="1" constant="false"/>

</listOfReactants>

<listOfProducts>

<speciesReference species="c_adp" stoichiometry="1" constant="false"/>

<speciesReference species="c_dtbt" stoichiometry="1" constant="false"/>

<speciesReference species="c_h" stoichiometry="3" constant="false"/>

<speciesReference species="c_pi" stoichiometry="1" constant="false"/>

</listOfProducts>

</reaction>

<reaction id="ASNS1" name="ASNS1" reversible="false" fast="false" compartment="cell">

<listOfReactants>

<speciesReference species="c_asp_L" stoichiometry="1" constant="false"/>

<speciesReference species="c_atp" stoichiometry="1" constant="false"/>

<speciesReference species="c_gln_L" stoichiometry="1" constant="false"/>

<speciesReference species="c_h2o" stoichiometry="1" constant="false"/>

</listOfReactants>

<listOfProducts>

<speciesReference species="c_amp" stoichiometry="1" constant="false"/>

<speciesReference species="c_asn_L" stoichiometry="1" constant="false"/>

<speciesReference species="c_glu_L" stoichiometry="1" constant="false"/>

<speciesReference species="c_h" stoichiometry="1" constant="false"/>

<speciesReference species="c_ppi" stoichiometry="1" constant="false"/>

</listOfProducts>

</reaction>

<reaction id="TMDK1" name="TMDK1" reversible="false" fast="false" compartment="cell">

<listOfReactants>

<speciesReference species="c_atp" stoichiometry="1" constant="false"/>

<speciesReference species="c_thymd" stoichiometry="1" constant="false"/>

</listOfReactants>

<listOfProducts>

<speciesReference species="c_adp" stoichiometry="1" constant="false"/>

<speciesReference species="c_dtmp" stoichiometry="1" constant="false"/>

<speciesReference species="c_h" stoichiometry="1" constant="false"/>

</listOfProducts>

</reaction>

<reaction id="PRATPP" name="PRATPP" reversible="false" fast="false" compartment="cell">

<listOfReactants>

<speciesReference species="c_h2o" stoichiometry="1" constant="false"/>

<speciesReference species="c_prbatp" stoichiometry="1" constant="false"/>

</listOfReactants>

<listOfProducts>

<speciesReference species="c_h" stoichiometry="1" constant="false"/>

<speciesReference species="c_ppi" stoichiometry="1" constant="false"/>

<speciesReference species="c_prbamp" stoichiometry="1" constant="false"/>

</listOfProducts>

</reaction>

<reaction id="C180SN" name="C180SN" reversible="false" fast="false" compartment="cell">

<listOfReactants>

<speciesReference species="c_actACP" stoichiometry="1" constant="false"/>

<speciesReference species="c_h" stoichiometry="23" constant="false"/>

<speciesReference species="c_malACP" stoichiometry="7" constant="false"/>

<speciesReference species="c_nadph" stoichiometry="16" constant="false"/>

</listOfReactants>

<listOfProducts>

<speciesReference species="c_ACP" stoichiometry="7" constant="false"/>

<speciesReference species="c_co2" stoichiometry="7" constant="false"/>

<speciesReference species="c_h2o" stoichiometry="8" constant="false"/>

<speciesReference species="c_nadp" stoichiometry="16" constant="false"/>

<speciesReference species="c_ocdACP" stoichiometry="1" constant="false"/>

</listOfProducts>

</reaction>

<reaction id="THRD_L" name="THRD_L" reversible="false" fast="false" compartment="cell">

<listOfReactants>

<speciesReference species="c_thr_L" stoichiometry="1" constant="false"/>

</listOfReactants>

<listOfProducts>

<speciesReference species="c_2obut" stoichiometry="1" constant="false"/>

<speciesReference species="c_nh4" stoichiometry="1" constant="false"/>

</listOfProducts>

</reaction>

<reaction id="GLYOX" name="GLYOX" reversible="false" fast="false" compartment="cell">

<listOfReactants>

<speciesReference species="c_h2o" stoichiometry="1" constant="false"/>

<speciesReference species="c_lgt_S" stoichiometry="1" constant="false"/>

</listOfReactants>

<listOfProducts>

<speciesReference species="c_gthrd" stoichiometry="1" constant="false"/>

<speciesReference species="c_h" stoichiometry="1" constant="false"/>

<speciesReference species="c_lac_D" stoichiometry="1" constant="false"/>

</listOfProducts>

</reaction>

<reaction id="DHQS" name="DHQS" reversible="false" fast="false" compartment="cell">

<listOfReactants>

<speciesReference species="c_2dda7p" stoichiometry="1" constant="false"/>

</listOfReactants>

<listOfProducts>

<speciesReference species="c_3dhq" stoichiometry="1" constant="false"/>

<speciesReference species="c_pi" stoichiometry="1" constant="false"/>

</listOfProducts>

</reaction>

<reaction id="ACGS" name="ACGS" reversible="false" fast="false" compartment="cell">

<listOfReactants>

<speciesReference species="c_accoa" stoichiometry="1" constant="false"/>

<speciesReference species="c_glu_L" stoichiometry="1" constant="false"/>

</listOfReactants>

<listOfProducts>

<speciesReference species="c_acglu" stoichiometry="1" constant="false"/>

<speciesReference species="c_coa" stoichiometry="1" constant="false"/>

<speciesReference species="c_h" stoichiometry="1" constant="false"/>

</listOfProducts>

</reaction>

<reaction id="AMAA" name="AMAA" reversible="false" fast="false" compartment="cell">

<listOfReactants>

<speciesReference species="c_acmama" stoichiometry="1" constant="false"/>

<speciesReference species="c_h2o" stoichiometry="1" constant="false"/>

</listOfReactants>

<listOfProducts>

<speciesReference species="c_acmam" stoichiometry="1" constant="false"/>

<speciesReference species="c_ala_L" stoichiometry="1" constant="false"/>

</listOfProducts>

</reaction>

<reaction id="URIK2" name="URIK2" reversible="false" fast="false" compartment="cell">

<listOfReactants>

<speciesReference species="c_gtp" stoichiometry="1" constant="false"/>

<speciesReference species="c_uri" stoichiometry="1" constant="false"/>

</listOfReactants>

<listOfProducts>

<speciesReference species="c_gdp" stoichiometry="1" constant="false"/>

<speciesReference species="c_h" stoichiometry="1" constant="false"/>

<speciesReference species="c_ump" stoichiometry="1" constant="false"/>

</listOfProducts>

</reaction>

<reaction id="URIK3" name="URIK3" reversible="false" fast="false" compartment="cell">

<listOfReactants>

<speciesReference species="c_itp" stoichiometry="1" constant="false"/>

<speciesReference species="c_uri" stoichiometry="1" constant="false"/>

</listOfReactants>

<listOfProducts>

<speciesReference species="c_h" stoichiometry="1" constant="false"/>

<speciesReference species="c_idp" stoichiometry="1" constant="false"/>

<speciesReference species="c_ump" stoichiometry="1" constant="false"/>

</listOfProducts>

</reaction>

<reaction id="URIK1" name="URIK1" reversible="false" fast="false" compartment="cell">

<listOfReactants>

<speciesReference species="c_atp" stoichiometry="1" constant="false"/>

<speciesReference species="c_uri" stoichiometry="1" constant="false"/>

</listOfReactants>

<listOfProducts>

<speciesReference species="c_adp" stoichiometry="1" constant="false"/>

<speciesReference species="c_h" stoichiometry="1" constant="false"/>

<speciesReference species="c_ump" stoichiometry="1" constant="false"/>

</listOfProducts>

</reaction>

<reaction id="ACGK" name="ACGK" reversible="false" fast="false" compartment="cell">

<listOfReactants>

<speciesReference species="c_acglu" stoichiometry="1" constant="false"/>

<speciesReference species="c_atp" stoichiometry="1" constant="false"/>

</listOfReactants>

<listOfProducts>

<speciesReference species="c_acg5p" stoichiometry="1" constant="false"/>

<speciesReference species="c_adp" stoichiometry="1" constant="false"/>

</listOfProducts>

</reaction>

<reaction id="ICDHxi" name="ICDHxi" reversible="false" fast="false" compartment="cell">

<listOfReactants>

<speciesReference species="c_icit" stoichiometry="1" constant="false"/>

<speciesReference species="c_nad" stoichiometry="1" constant="false"/>

</listOfReactants>

<listOfProducts>

<speciesReference species="c_akg" stoichiometry="1" constant="false"/>

<speciesReference species="c_co2" stoichiometry="1" constant="false"/>

<speciesReference species="c_nadh" stoichiometry="1" constant="false"/>

</listOfProducts>

</reaction>

<reaction id="ASNTRS" name="ASNTRS" reversible="false" fast="false" compartment="cell">

<listOfReactants>

<speciesReference species="c_asn_L" stoichiometry="1" constant="false"/>

<speciesReference species="c_atp" stoichiometry="1" constant="false"/>

<speciesReference species="c_trnaasn" stoichiometry="1" constant="false"/>

</listOfReactants>

<listOfProducts>

<speciesReference species="c_amp" stoichiometry="1" constant="false"/>

<speciesReference species="c_asntrna" stoichiometry="1" constant="false"/>

<speciesReference species="c_ppi" stoichiometry="1" constant="false"/>

</listOfProducts>

</reaction>

<reaction id="GALKr" name="GALKr" reversible="true" fast="false" compartment="cell">

<listOfReactants>

<speciesReference species="c_atp" stoichiometry="1" constant="false"/>

<speciesReference species="c_gal" stoichiometry="1" constant="false"/>

</listOfReactants>

<listOfProducts>

<speciesReference species="c_adp" stoichiometry="1" constant="false"/>

<speciesReference species="c_gal1p" stoichiometry="1" constant="false"/>

<speciesReference species="c_h" stoichiometry="1" constant="false"/>

</listOfProducts>

</reaction>

<reaction id="GK1" name="GK1" reversible="true" fast="false" compartment="cell">

<listOfReactants>

<speciesReference species="c_atp" stoichiometry="1" constant="false"/>

<speciesReference species="c_gmp" stoichiometry="1" constant="false"/>

</listOfReactants>

<listOfProducts>

<speciesReference species="c_adp" stoichiometry="1" constant="false"/>

<speciesReference species="c_gdp" stoichiometry="1" constant="false"/>

</listOfProducts>

</reaction>

<reaction id="MALS" name="MALS" reversible="false" fast="false" compartment="cell">

<listOfReactants>

<speciesReference species="c_accoa" stoichiometry="1" constant="false"/>

<speciesReference species="c_glx" stoichiometry="1" constant="false"/>

<speciesReference species="c_h2o" stoichiometry="1" constant="false"/>

</listOfReactants>

<listOfProducts>

<speciesReference species="c_coa" stoichiometry="1" constant="false"/>

<speciesReference species="c_h" stoichiometry="1" constant="false"/>

<speciesReference species="c_mal_L" stoichiometry="1" constant="false"/>

</listOfProducts>

</reaction>

<reaction id="SGSAD" name="SGSAD" reversible="false" fast="false" compartment="cell">

<listOfReactants>

<speciesReference species="c_h2o" stoichiometry="1" constant="false"/>

<speciesReference species="c_nad" stoichiometry="1" constant="false"/>

<speciesReference species="c_sucgsa" stoichiometry="1" constant="false"/>

</listOfReactants>

<listOfProducts>

<speciesReference species="c_h" stoichiometry="2" constant="false"/>

<speciesReference species="c_nadh" stoichiometry="1" constant="false"/>

<speciesReference species="c_sucglu" stoichiometry="1" constant="false"/>

</listOfProducts>

</reaction>

<reaction name="MEAMP1(gly-glu)" reversible="true" fast="false" compartment="cell">

<listOfReactants>

<speciesReference species="c_gly_glu_L" stoichiometry="1" constant="false"/>

<speciesReference species="c_h2o" stoichiometry="1" constant="false"/>

</listOfReactants>

<listOfProducts>

<speciesReference species="c_glu_L" stoichiometry="1" constant="false"/>

<speciesReference species="c_gly" stoichiometry="1" constant="false"/>

</listOfProducts>

</reaction>

<reaction id="ILEDH2" name="ILEDH2" reversible="true" fast="false" compartment="cell">

<listOfReactants>

<speciesReference species="c_h2o" stoichiometry="1" constant="false"/>

<speciesReference species="c_ile_L" stoichiometry="1" constant="false"/>

<speciesReference species="c_nad" stoichiometry="1" constant="false"/>

</listOfReactants>

<listOfProducts>

<speciesReference species="c_3mop" stoichiometry="1" constant="false"/>

<speciesReference species="c_h" stoichiometry="1" constant="false"/>

<speciesReference species="c_nadh" stoichiometry="1" constant="false"/>

<speciesReference species="c_nh4" stoichiometry="1" constant="false"/>

</listOfProducts>

</reaction>

<reaction id="ADCOBAK" name="ADCOBAK" reversible="false" fast="false" compartment="cell">

<listOfReactants>

<speciesReference species="c_adcoba" stoichiometry="1" constant="false"/>

<speciesReference species="c_atp" stoichiometry="1" constant="false"/>

</listOfReactants>

<listOfProducts>

<speciesReference species="c_adcobap" stoichiometry="1" constant="false"/>

<speciesReference species="c_adp" stoichiometry="1" constant="false"/>

<speciesReference species="c_h" stoichiometry="1" constant="false"/>

</listOfProducts>

</reaction>

<reaction id="MMSDHir" name="MMSDHir" reversible="false" fast="false" compartment="cell">

<listOfReactants>

<speciesReference species="c_coa" stoichiometry="1" constant="false"/>

<speciesReference species="c_mmalsa" stoichiometry="1" constant="false"/>

<speciesReference species="c_nad" stoichiometry="1" constant="false"/>

</listOfReactants>

<listOfProducts>

<speciesReference species="c_co2" stoichiometry="1" constant="false"/>

<speciesReference species="c_nadh" stoichiometry="1" constant="false"/>

<speciesReference species="c_ppcoa" stoichiometry="1" constant="false"/>

</listOfProducts>

</reaction>

<reaction id="GGLUGABDH" name="GGLUGABDH" reversible="false" fast="false" compartment="cell">

<listOfReactants>

<speciesReference species="c_ggluaba" stoichiometry="1" constant="false"/>

<speciesReference species="c_h2o" stoichiometry="1" constant="false"/>

<speciesReference species="c_nadp" stoichiometry="1" constant="false"/>

</listOfReactants>

<listOfProducts>

<speciesReference species="c_ggluabt" stoichiometry="1" constant="false"/>

<speciesReference species="c_h" stoichiometry="2" constant="false"/>

<speciesReference species="c_nadph" stoichiometry="1" constant="false"/>

</listOfProducts>

</reaction>

<reaction id="ALDD2x" name="ALDD2x" reversible="false" fast="false" compartment="cell">

<listOfReactants>

<speciesReference species="c_acald" stoichiometry="1" constant="false"/>

<speciesReference species="c_h2o" stoichiometry="1" constant="false"/>

<speciesReference species="c_nad" stoichiometry="1" constant="false"/>

</listOfReactants>

<listOfProducts>

<speciesReference species="c_ac" stoichiometry="1" constant="false"/>

<speciesReference species="c_h" stoichiometry="2" constant="false"/>

<speciesReference species="c_nadh" stoichiometry="1" constant="false"/>

</listOfProducts>

</reaction>

<reaction name="L-LACD2" reversible="false" fast="false" compartment="cell">

<listOfReactants>

<speciesReference species="c_lac_L" stoichiometry="1" constant="false"/>

<speciesReference species="c_ubq8" stoichiometry="1" constant="false"/>

</listOfReactants>

<listOfProducts>

<speciesReference species="c_pyr" stoichiometry="1" constant="false"/>

<speciesReference species="c_ubq8h2" stoichiometry="1" constant="false"/>

</listOfProducts>

</reaction>

<reaction id="PLIPPE_SO" name="PLIPPE_SO" reversible="false" fast="false" compartment="cell">

<listOfReactants>

<speciesReference species="c_h2o" stoichiometry="1" constant="false"/>

<speciesReference species="c_pe" stoichiometry="1" constant="false"/>

</listOfReactants>

<listOfProducts>

<speciesReference species="c_agpe" stoichiometry="1" constant="false"/>

<speciesReference species="c_fa1" stoichiometry="0.06" constant="false"/>

<speciesReference species="c_fa11" stoichiometry="0.065" constant="false"/>

<speciesReference species="c_fa13" stoichiometry="0.035" constant="false"/>

<speciesReference species="c_fa3" stoichiometry="0.165" constant="false"/>

<speciesReference species="c_fa6" stoichiometry="0.075" constant="false"/>

<speciesReference species="c_h" stoichiometry="1" constant="false"/>

<speciesReference species="c_hdca" stoichiometry="0.065" constant="false"/>

<speciesReference species="c_hdcea" stoichiometry="0.19" constant="false"/>

<speciesReference species="c_hpdca" stoichiometry="0.015" constant="false"/>

<speciesReference species="c_hpde" stoichiometry="0.125" constant="false"/>

<speciesReference species="c_ocdca" stoichiometry="0.01" constant="false"/>

<speciesReference species="c_ocdcea" stoichiometry="0.125" constant="false"/>

<speciesReference species="c_pde" stoichiometry="0.06" constant="false"/>

<speciesReference species="c_ptdca" stoichiometry="0.01" constant="false"/>

</listOfProducts>

</reaction>

<reaction id="GLNTRS" name="GLNTRS" reversible="false" fast="false" compartment="cell">

<listOfReactants>

<speciesReference species="c_atp" stoichiometry="1" constant="false"/>

<speciesReference species="c_gln_L" stoichiometry="1" constant="false"/>

<speciesReference species="c_trnagln" stoichiometry="1" constant="false"/>

</listOfReactants>

<listOfProducts>

<speciesReference species="c_amp" stoichiometry="1" constant="false"/>

<speciesReference species="c_glntrna" stoichiometry="1" constant="false"/>

<speciesReference species="c_ppi" stoichiometry="1" constant="false"/>

</listOfProducts>

</reaction>

<reaction id="OMPDC" name="OMPDC" reversible="false" fast="false" compartment="cell">

<listOfReactants>

<speciesReference species="c_h" stoichiometry="1" constant="false"/>

<speciesReference species="c_orot5p" stoichiometry="1" constant="false"/>

</listOfReactants>

<listOfProducts>

<speciesReference species="c_co2" stoichiometry="1" constant="false"/>

<speciesReference species="c_ump" stoichiometry="1" constant="false"/>

</listOfProducts>

</reaction>

<reaction id="ADCOBAS" name="ADCOBAS" reversible="false" fast="false" compartment="cell">

<listOfReactants>

<speciesReference species="c_1ap2ol" stoichiometry="1" constant="false"/>

<speciesReference species="c_adcobhex" stoichiometry="1" constant="false"/>

</listOfReactants>

<listOfProducts>

<speciesReference species="c_adcoba" stoichiometry="1" constant="false"/>

<speciesReference species="c_h2o" stoichiometry="1" constant="false"/>

</listOfProducts>

</reaction>

<reaction id="GCALDD" name="GCALDD" reversible="false" fast="false" compartment="cell">

<listOfReactants>

<speciesReference species="c_gcald" stoichiometry="1" constant="false"/>

<speciesReference species="c_h2o" stoichiometry="1" constant="false"/>

<speciesReference species="c_nad" stoichiometry="1" constant="false"/>

</listOfReactants>

<listOfProducts>

<speciesReference species="c_glyclt" stoichiometry="1" constant="false"/>

<speciesReference species="c_h" stoichiometry="2" constant="false"/>

<speciesReference species="c_nadh" stoichiometry="1" constant="false"/>

</listOfProducts>

</reaction>

<reaction id="ICHORSi" name="ICHORSi" reversible="false" fast="false" compartment="cell">

<listOfReactants>

<speciesReference species="c_chor" stoichiometry="1" constant="false"/>

</listOfReactants>

<listOfProducts>

<speciesReference species="c_ichor" stoichiometry="1" constant="false"/>

</listOfProducts>

</reaction>

<reaction id="SPRS" name="SPRS" reversible="false" fast="false" compartment="cell">

<listOfReactants>

<speciesReference species="c_ametam" stoichiometry="1" constant="false"/>

<speciesReference species="c_spmd" stoichiometry="1" constant="false"/>

</listOfReactants>

<listOfProducts>

<speciesReference species="c_5mta" stoichiometry="1" constant="false"/>

<speciesReference species="c_h" stoichiometry="1" constant="false"/>

<speciesReference species="c_sprm" stoichiometry="1" constant="false"/>

</listOfProducts>

</reaction>

<reaction id="DMPPS" name="DMPPS" reversible="false" fast="false" compartment="cell">

<listOfReactants>

<speciesReference species="c_h" stoichiometry="1" constant="false"/>

<speciesReference species="c_h2mb4p" stoichiometry="1" constant="false"/>

<speciesReference species="c_nadh" stoichiometry="1" constant="false"/>

</listOfReactants>

<listOfProducts>

<speciesReference species="c_dmpp" stoichiometry="1" constant="false"/>

<speciesReference species="c_h2o" stoichiometry="1" constant="false"/>

<speciesReference species="c_nad" stoichiometry="1" constant="false"/>

</listOfProducts>

</reaction>

<reaction id="PSD" name="PSD" reversible="false" fast="false" compartment="cell">

<listOfReactants>

<speciesReference species="c_h" stoichiometry="1" constant="false"/>

<speciesReference species="c_ps" stoichiometry="1" constant="false"/>

</listOfReactants>

<listOfProducts>

<speciesReference species="c_co2" stoichiometry="1" constant="false"/>

<speciesReference species="c_pe" stoichiometry="1" constant="false"/>

</listOfProducts>

</reaction>

<reaction id="GRTT" name="GRTT" reversible="false" fast="false" compartment="cell">

<listOfReactants>

<speciesReference species="c_grdp" stoichiometry="1" constant="false"/>

<speciesReference species="c_ipdp" stoichiometry="1" constant="false"/>

</listOfReactants>

<listOfProducts>

<speciesReference species="c_frdp" stoichiometry="1" constant="false"/>

<speciesReference species="c_ppi" stoichiometry="1" constant="false"/>

</listOfProducts>

</reaction>

<reaction id="ANPRT" name="ANPRT" reversible="false" fast="false" compartment="cell">

<listOfReactants>

<speciesReference species="c_anth" stoichiometry="1" constant="false"/>

<speciesReference species="c_prpp" stoichiometry="1" constant="false"/>

</listOfReactants>

<listOfProducts>

<speciesReference species="c_ppi" stoichiometry="1" constant="false"/>

<speciesReference species="c_pran" stoichiometry="1" constant="false"/>

</listOfProducts>

</reaction>

<reaction id="PHE4MO" name="PHE4MO" reversible="false" fast="false" compartment="cell">

<listOfReactants>

<speciesReference species="c_o2" stoichiometry="1" constant="false"/>

<speciesReference species="c_phe_L" stoichiometry="1" constant="false"/>

<speciesReference species="c_thbpt" stoichiometry="1" constant="false"/>

</listOfReactants>

<listOfProducts>

<speciesReference species="c_dhbpt" stoichiometry="1" constant="false"/>

<speciesReference species="c_h2o" stoichiometry="1" constant="false"/>

<speciesReference species="c_tyr_L" stoichiometry="1" constant="false"/>

</listOfProducts>

</reaction>

<reaction id="NADK" name="NADK" reversible="false" fast="false" compartment="cell">

<listOfReactants>

<speciesReference species="c_atp" stoichiometry="1" constant="false"/>

<speciesReference species="c_nad" stoichiometry="1" constant="false"/>

</listOfReactants>

<listOfProducts>

<speciesReference species="c_adp" stoichiometry="1" constant="false"/>

<speciesReference species="c_h" stoichiometry="1" constant="false"/>

<speciesReference species="c_nadp" stoichiometry="1" constant="false"/>

</listOfProducts>

</reaction>

<reaction id="OXGDC2" name="OXGDC2" reversible="false" fast="false" compartment="cell">

<listOfReactants>

<speciesReference species="c_akg" stoichiometry="1" constant="false"/>

<speciesReference species="c_h" stoichiometry="2" constant="false"/>

<speciesReference species="c_thmpp" stoichiometry="1" constant="false"/>

</listOfReactants>

<listOfProducts>

<speciesReference species="c_co2" stoichiometry="1" constant="false"/>

<speciesReference species="c_ssaltpp" stoichiometry="1" constant="false"/>

</listOfProducts>

</reaction>

<reaction id="PNTK" name="PNTK" reversible="false" fast="false" compartment="cell">

<listOfReactants>

<speciesReference species="c_atp" stoichiometry="1" constant="false"/>

<speciesReference species="c_pnto_R" stoichiometry="1" constant="false"/>

</listOfReactants>

<listOfProducts>

<speciesReference species="c_4ppan" stoichiometry="1" constant="false"/>

<speciesReference species="c_adp" stoichiometry="1" constant="false"/>

<speciesReference species="c_h" stoichiometry="1" constant="false"/>

</listOfProducts>

</reaction>

<reaction id="UAGCVT" name="UAGCVT" reversible="false" fast="false" compartment="cell">

<listOfReactants>

<speciesReference species="c_pep" stoichiometry="1" constant="false"/>

<speciesReference species="c_uacgam" stoichiometry="1" constant="false"/>

</listOfReactants>

<listOfProducts>

<speciesReference species="c_pi" stoichiometry="1" constant="false"/>

<speciesReference species="c_uaccg" stoichiometry="1" constant="false"/>

</listOfProducts>

</reaction>

<reaction id="TSULST" name="TSULST" reversible="false" fast="false" compartment="cell">

<listOfReactants>

<speciesReference species="c_cyan" stoichiometry="1" constant="false"/>

<speciesReference species="c_tsul" stoichiometry="1" constant="false"/>

</listOfReactants>

<listOfProducts>

<speciesReference species="c_h" stoichiometry="1" constant="false"/>

<speciesReference species="c_so3" stoichiometry="1" constant="false"/>

<speciesReference species="c_tcynt" stoichiometry="1" constant="false"/>

</listOfProducts>

</reaction>

<reaction id="MLACI" name="MLACI" reversible="false" fast="false" compartment="cell">

<listOfReactants>

<speciesReference species="c_4mlacac" stoichiometry="1" constant="false"/>

</listOfReactants>

<listOfProducts>

<speciesReference species="c_4fumacac" stoichiometry="1" constant="false"/>

</listOfProducts>

</reaction>

<reaction id="A5PISO" name="A5PISO" reversible="true" fast="false" compartment="cell">

<listOfReactants>

<speciesReference species="c_ru5p_D" stoichiometry="1" constant="false"/>

</listOfReactants>

<listOfProducts>

<speciesReference species="c_ara5p" stoichiometry="1" constant="false"/>

</listOfProducts>

</reaction>

<reaction id="ARGSL" name="ARGSL" reversible="true" fast="false" compartment="cell">

<listOfReactants>

<speciesReference species="c_argsuc" stoichiometry="1" constant="false"/>

</listOfReactants>

<listOfProducts>

<speciesReference species="c_arg_L" stoichiometry="1" constant="false"/>

<speciesReference species="c_fum" stoichiometry="1" constant="false"/>

</listOfProducts>

</reaction>

<reaction id="CTPS2" name="CTPS2" reversible="false" fast="false" compartment="cell">

<listOfReactants>

<speciesReference species="c_atp" stoichiometry="1" constant="false"/>

<speciesReference species="c_gln_L" stoichiometry="1" constant="false"/>

<speciesReference species="c_h2o" stoichiometry="1" constant="false"/>

<speciesReference species="c_utp" stoichiometry="1" constant="false"/>

</listOfReactants>

<listOfProducts>

<speciesReference species="c_adp" stoichiometry="1" constant="false"/>

<speciesReference species="c_ctp" stoichiometry="1" constant="false"/>

<speciesReference species="c_glu_L" stoichiometry="1" constant="false"/>

<speciesReference species="c_h" stoichiometry="2" constant="false"/>

<speciesReference species="c_pi" stoichiometry="1" constant="false"/>

</listOfProducts>

</reaction>

<reaction id="HPPDO1" name="HPPDO1" reversible="false" fast="false" compartment="cell">

<listOfReactants>

<speciesReference species="c_34hpp" stoichiometry="1" constant="false"/>

<speciesReference species="c_o2" stoichiometry="1" constant="false"/>

</listOfReactants>

<listOfProducts>

<speciesReference species="c_co2" stoichiometry="1" constant="false"/>

<speciesReference species="c_hgentis" stoichiometry="1" constant="false"/>

</listOfProducts>

</reaction>

<reaction id="GGLUPTO" name="GGLUPTO" reversible="false" fast="false" compartment="cell">

<listOfReactants>

<speciesReference species="c_ggluptrc" stoichiometry="1" constant="false"/>

<speciesReference species="c_h2o" stoichiometry="1" constant="false"/>

<speciesReference species="c_o2" stoichiometry="1" constant="false"/>

</listOfReactants>

<listOfProducts>

<speciesReference species="c_ggluaba" stoichiometry="1" constant="false"/>

<speciesReference species="c_h2o2" stoichiometry="1" constant="false"/>

<speciesReference species="c_nh4" stoichiometry="1" constant="false"/>

</listOfProducts>

</reaction>

<reaction id="OBTFL" name="OBTFL" reversible="false" fast="false" compartment="cell">

<listOfReactants>

<speciesReference species="c_2obut" stoichiometry="1" constant="false"/>

<speciesReference species="c_coa" stoichiometry="1" constant="false"/>

</listOfReactants>

<listOfProducts>

<speciesReference species="c_for" stoichiometry="1" constant="false"/>

<speciesReference species="c_ppcoa" stoichiometry="1" constant="false"/>

</listOfProducts>

</reaction>

<reaction id="UMPK" name="UMPK" reversible="true" fast="false" compartment="cell">

<listOfReactants>

<speciesReference species="c_atp" stoichiometry="1" constant="false"/>

<speciesReference species="c_ump" stoichiometry="1" constant="false"/>

</listOfReactants>

<listOfProducts>

<speciesReference species="c_adp" stoichiometry="1" constant="false"/>

<speciesReference species="c_udp" stoichiometry="1" constant="false"/>

</listOfProducts>

</reaction>

<reaction id="GGLUPTS" name="GGLUPTS" reversible="false" fast="false" compartment="cell">

<listOfReactants>

<speciesReference species="c_atp" stoichiometry="1" constant="false"/>

<speciesReference species="c_glu_L" stoichiometry="1" constant="false"/>

<speciesReference species="c_ptrc" stoichiometry="1" constant="false"/>

</listOfReactants>

<listOfProducts>

<speciesReference species="c_adp" stoichiometry="1" constant="false"/>

<speciesReference species="c_ggluptrc" stoichiometry="1" constant="false"/>

<speciesReference species="c_h" stoichiometry="1" constant="false"/>

<speciesReference species="c_pi" stoichiometry="1" constant="false"/>

</listOfProducts>

</reaction>

<reaction id="GARFT" name="GARFT" reversible="true" fast="false" compartment="cell">

<listOfReactants>

<speciesReference species="c_10fthf" stoichiometry="1" constant="false"/>

<speciesReference species="c_gar" stoichiometry="1" constant="false"/>

</listOfReactants>

<listOfProducts>

<speciesReference species="c_fgam" stoichiometry="1" constant="false"/>

<speciesReference species="c_h" stoichiometry="1" constant="false"/>

<speciesReference species="c_thf" stoichiometry="1" constant="false"/>

</listOfProducts>

</reaction>

<reaction id="UAMAS" name="UAMAS" reversible="false" fast="false" compartment="cell">

<listOfReactants>

<speciesReference species="c_ala_L" stoichiometry="1" constant="false"/>

<speciesReference species="c_atp" stoichiometry="1" constant="false"/>

<speciesReference species="c_uamr" stoichiometry="1" constant="false"/>

</listOfReactants>

<listOfProducts>

<speciesReference species="c_adp" stoichiometry="1" constant="false"/>

<speciesReference species="c_h" stoichiometry="1" constant="false"/>

<speciesReference species="c_pi" stoichiometry="1" constant="false"/>

<speciesReference species="c_uama" stoichiometry="1" constant="false"/>

</listOfProducts>

</reaction>

<reaction id="ARGSS" name="ARGSS" reversible="false" fast="false" compartment="cell">

<listOfReactants>

<speciesReference species="c_asp_L" stoichiometry="1" constant="false"/>

<speciesReference species="c_atp" stoichiometry="1" constant="false"/>

<speciesReference species="c_citr_L" stoichiometry="1" constant="false"/>

</listOfReactants>

<listOfProducts>

<speciesReference species="c_amp" stoichiometry="1" constant="false"/>

<speciesReference species="c_argsuc" stoichiometry="1" constant="false"/>

<speciesReference species="c_h" stoichiometry="1" constant="false"/>

<speciesReference species="c_ppi" stoichiometry="1" constant="false"/>

</listOfProducts>

</reaction>

<reaction name="MEAMP1(gly-asp)" reversible="true" fast="false" compartment="cell">

<listOfReactants>

<speciesReference species="c_gly_asp_L" stoichiometry="1" constant="false"/>

<speciesReference species="c_h2o" stoichiometry="1" constant="false"/>

</listOfReactants>

<listOfProducts>

<speciesReference species="c_asp_L" stoichiometry="1" constant="false"/>

<speciesReference species="c_gly" stoichiometry="1" constant="false"/>

</listOfProducts>

</reaction>

<reaction id="GALU" name="GALU" reversible="true" fast="false" compartment="cell">

<listOfReactants>

<speciesReference species="c_g1p" stoichiometry="1" constant="false"/>

<speciesReference species="c_h" stoichiometry="1" constant="false"/>

<speciesReference species="c_utp" stoichiometry="1" constant="false"/>

</listOfReactants>

<listOfProducts>

<speciesReference species="c_ppi" stoichiometry="1" constant="false"/>

<speciesReference species="c_udpg" stoichiometry="1" constant="false"/>

</listOfProducts>

</reaction>

<reaction id="HSST" name="HSST" reversible="false" fast="false" compartment="cell">

<listOfReactants>

<speciesReference species="c_hom_L" stoichiometry="1" constant="false"/>

<speciesReference species="c_succoa" stoichiometry="1" constant="false"/>

</listOfReactants>

<listOfProducts>

<speciesReference species="c_coa" stoichiometry="1" constant="false"/>

<speciesReference species="c_suchms" stoichiometry="1" constant="false"/>

</listOfProducts>

</reaction>

<reaction id="OIVD3" name="OIVD3" reversible="false" fast="false" compartment="cell">

<listOfReactants>

<speciesReference species="c_3mop" stoichiometry="1" constant="false"/>

<speciesReference species="c_coa" stoichiometry="1" constant="false"/>

<speciesReference species="c_nad" stoichiometry="1" constant="false"/>

</listOfReactants>

<listOfProducts>

<speciesReference species="c_2mbcoa" stoichiometry="1" constant="false"/>

<speciesReference species="c_co2" stoichiometry="1" constant="false"/>

<speciesReference species="c_nadh" stoichiometry="1" constant="false"/>

</listOfProducts>

</reaction>

<reaction id="OIVD2" name="OIVD2" reversible="false" fast="false" compartment="cell">

<listOfReactants>

<speciesReference species="c_3mob" stoichiometry="1" constant="false"/>

<speciesReference species="c_coa" stoichiometry="1" constant="false"/>

<speciesReference species="c_nad" stoichiometry="1" constant="false"/>

</listOfReactants>

<listOfProducts>

<speciesReference species="c_co2" stoichiometry="1" constant="false"/>

<speciesReference species="c_ibcoa" stoichiometry="1" constant="false"/>

<speciesReference species="c_nadh" stoichiometry="1" constant="false"/>

</listOfProducts>

</reaction>

<reaction id="GLCP" name="GLCP" reversible="false" fast="false" compartment="cell">

<listOfReactants>

<speciesReference species="c_glycogen" stoichiometry="1" constant="false"/>

<speciesReference species="c_pi" stoichiometry="1" constant="false"/>

</listOfReactants>

<listOfProducts>

<speciesReference species="c_g1p" stoichiometry="1" constant="false"/>

</listOfProducts>

</reaction>

<reaction id="CS" name="CS" reversible="false" fast="false" compartment="cell">

<listOfReactants>

<speciesReference species="c_accoa" stoichiometry="1" constant="false"/>

<speciesReference species="c_h2o" stoichiometry="1" constant="false"/>

<speciesReference species="c_oaa" stoichiometry="1" constant="false"/>

</listOfReactants>

<listOfProducts>

<speciesReference species="c_cit" stoichiometry="1" constant="false"/>

<speciesReference species="c_coa" stoichiometry="1" constant="false"/>

<speciesReference species="c_h" stoichiometry="1" constant="false"/>

</listOfProducts>

</reaction>

<reaction id="UGMDDS" name="UGMDDS" reversible="false" fast="false" compartment="cell">

<listOfReactants>

<speciesReference species="c_alaala" stoichiometry="1" constant="false"/>

<speciesReference species="c_atp" stoichiometry="1" constant="false"/>

<speciesReference species="c_ugmd" stoichiometry="1" constant="false"/>

</listOfReactants>

<listOfProducts>

<speciesReference species="c_adp" stoichiometry="1" constant="false"/>

<speciesReference species="c_h" stoichiometry="1" constant="false"/>

<speciesReference species="c_pi" stoichiometry="1" constant="false"/>

<speciesReference species="c_ugmda" stoichiometry="1" constant="false"/>

</listOfProducts>

</reaction>

<reaction id="PPPGO" name="PPPGO" reversible="false" fast="false" compartment="cell">

<listOfReactants>

<speciesReference species="c_o2" stoichiometry="1.5" constant="false"/>

<speciesReference species="c_pppg9" stoichiometry="1" constant="false"/>

</listOfReactants>

<listOfProducts>

<speciesReference species="c_h2o" stoichiometry="3" constant="false"/>

<speciesReference species="c_ppp9" stoichiometry="1" constant="false"/>

</listOfProducts>

</reaction>

<reaction id="PUNP4" name="PUNP4" reversible="true" fast="false" compartment="cell">

<listOfReactants>

<speciesReference species="c_dgsn" stoichiometry="1" constant="false"/>

<speciesReference species="c_pi" stoichiometry="1" constant="false"/>

</listOfReactants>

<listOfProducts>

<speciesReference species="c_2dr1p" stoichiometry="1" constant="false"/>

<speciesReference species="c_gua" stoichiometry="1" constant="false"/>

</listOfProducts>

</reaction>

<reaction id="PUNP5" name="PUNP5" reversible="true" fast="false" compartment="cell">

<listOfReactants>

<speciesReference species="c_ins" stoichiometry="1" constant="false"/>

<speciesReference species="c_pi" stoichiometry="1" constant="false"/>

</listOfReactants>

<listOfProducts>

<speciesReference species="c_hxan" stoichiometry="1" constant="false"/>

<speciesReference species="c_r1p" stoichiometry="1" constant="false"/>

</listOfProducts>

</reaction>

<reaction id="PUNP6" name="PUNP6" reversible="true" fast="false" compartment="cell">

<listOfReactants>

<speciesReference species="c_din" stoichiometry="1" constant="false"/>

<speciesReference species="c_pi" stoichiometry="1" constant="false"/>

</listOfReactants>

<listOfProducts>

<speciesReference species="c_2dr1p" stoichiometry="1" constant="false"/>

<speciesReference species="c_hxan" stoichiometry="1" constant="false"/>

</listOfProducts>

</reaction>

<reaction id="C151SN" name="C151SN" reversible="false" fast="false" compartment="cell">

<listOfReactants>

<speciesReference species="c_h" stoichiometry="17" constant="false"/>

<speciesReference species="c_malACP" stoichiometry="6" constant="false"/>

<speciesReference species="c_nadph" stoichiometry="11" constant="false"/>

<speciesReference species="c_ppcoa" stoichiometry="1" constant="false"/>

</listOfReactants>

<listOfProducts>

<speciesReference species="c_ACP" stoichiometry="5" constant="false"/>

<speciesReference species="c_co2" stoichiometry="6" constant="false"/>

<speciesReference species="c_coa" stoichiometry="1" constant="false"/>

<speciesReference species="c_h2o" stoichiometry="6" constant="false"/>

<speciesReference species="c_nadp" stoichiometry="11" constant="false"/>

<speciesReference species="c_pdeACP" stoichiometry="1" constant="false"/>

</listOfProducts>

</reaction>

<reaction id="PUNP1" name="PUNP1" reversible="true" fast="false" compartment="cell">

<listOfReactants>

<speciesReference species="c_adn" stoichiometry="1" constant="false"/>

<speciesReference species="c_pi" stoichiometry="1" constant="false"/>

</listOfReactants>

<listOfProducts>

<speciesReference species="c_ade" stoichiometry="1" constant="false"/>

<speciesReference species="c_r1p" stoichiometry="1" constant="false"/>

</listOfProducts>

</reaction>

<reaction id="PUNP2" name="PUNP2" reversible="true" fast="false" compartment="cell">

<listOfReactants>

<speciesReference species="c_dad_2" stoichiometry="1" constant="false"/>

<speciesReference species="c_pi" stoichiometry="1" constant="false"/>

</listOfReactants>

<listOfProducts>

<speciesReference species="c_2dr1p" stoichiometry="1" constant="false"/>

<speciesReference species="c_ade" stoichiometry="1" constant="false"/>

</listOfProducts>

</reaction>

<reaction id="GP4GH" name="GP4GH" reversible="false" fast="false" compartment="cell">

<listOfReactants>

<speciesReference species="c_gp4g" stoichiometry="1" constant="false"/>

<speciesReference species="c_h2o" stoichiometry="1" constant="false"/>

</listOfReactants>

<listOfProducts>

<speciesReference species="c_gdp" stoichiometry="2" constant="false"/>

<speciesReference species="c_h" stoichiometry="2" constant="false"/>

</listOfProducts>

</reaction>

<reaction id="ASPO8" name="ASPO8" reversible="false" fast="false" compartment="cell">

<listOfReactants>

<speciesReference species="c_asp_L" stoichiometry="1" constant="false"/>

<speciesReference species="c_mqn7" stoichiometry="1" constant="false"/>

</listOfReactants>

<listOfProducts>

<speciesReference species="c_h" stoichiometry="1" constant="false"/>

<speciesReference species="c_iasp" stoichiometry="1" constant="false"/>

<speciesReference species="c_mql7" stoichiometry="1" constant="false"/>

</listOfProducts>

</reaction>

<reaction id="ASPO9" name="ASPO9" reversible="false" fast="false" compartment="cell">

<listOfReactants>

<speciesReference species="c_asp_L" stoichiometry="1" constant="false"/>

<speciesReference species="c_mmqn7" stoichiometry="1" constant="false"/>

</listOfReactants>

<listOfProducts>

<speciesReference species="c_h" stoichiometry="1" constant="false"/>

<speciesReference species="c_iasp" stoichiometry="1" constant="false"/>

<speciesReference species="c_mmql7" stoichiometry="1" constant="false"/>

</listOfProducts>

</reaction>

<reaction id="C120SN" name="C120SN" reversible="false" fast="false" compartment="cell">

<listOfReactants>

<speciesReference species="c_actACP" stoichiometry="1" constant="false"/>

<speciesReference species="c_h" stoichiometry="14" constant="false"/>

<speciesReference species="c_malACP" stoichiometry="4" constant="false"/>

<speciesReference species="c_nadph" stoichiometry="10" constant="false"/>

</listOfReactants>

<listOfProducts>

<speciesReference species="c_ACP" stoichiometry="4" constant="false"/>

<speciesReference species="c_co2" stoichiometry="4" constant="false"/>

<speciesReference species="c_ddcaACP" stoichiometry="1" constant="false"/>

<speciesReference species="c_h2o" stoichiometry="5" constant="false"/>

<speciesReference species="c_nadp" stoichiometry="10" constant="false"/>

</listOfProducts>

</reaction>

<reaction id="OHPHM" name="OHPHM" reversible="false" fast="false" compartment="cell">

<listOfReactants>

<speciesReference species="c_2ohph" stoichiometry="1" constant="false"/>

<speciesReference species="c_amet" stoichiometry="1" constant="false"/>

</listOfReactants>

<listOfProducts>

<speciesReference species="c_2omph" stoichiometry="1" constant="false"/>

<speciesReference species="c_ahcys" stoichiometry="1" constant="false"/>

<speciesReference species="c_h" stoichiometry="1" constant="false"/>

</listOfProducts>

</reaction>

<reaction id="PMPK" name="PMPK" reversible="false" fast="false" compartment="cell">

<listOfReactants>

<speciesReference species="c_4ampm" stoichiometry="1" constant="false"/>

<speciesReference species="c_atp" stoichiometry="1" constant="false"/>

</listOfReactants>

<listOfProducts>

<speciesReference species="c_2mahmp" stoichiometry="1" constant="false"/>

<speciesReference species="c_adp" stoichiometry="1" constant="false"/>

</listOfProducts>

</reaction>

<reaction id="ASPO3" name="ASPO3" reversible="false" fast="false" compartment="cell">

<listOfReactants>

<speciesReference species="c_asp_L" stoichiometry="1" constant="false"/>

<speciesReference species="c_ubq8" stoichiometry="1" constant="false"/>

</listOfReactants>

<listOfProducts>

<speciesReference species="c_h" stoichiometry="1" constant="false"/>

<speciesReference species="c_iasp" stoichiometry="1" constant="false"/>

<speciesReference species="c_ubq8h2" stoichiometry="1" constant="false"/>

</listOfProducts>

</reaction>

<reaction id="ASPO5" name="ASPO5" reversible="false" fast="false" compartment="cell">

<listOfReactants>

<speciesReference species="c_asp_L" stoichiometry="1" constant="false"/>

<speciesReference species="c_fum" stoichiometry="1" constant="false"/>

</listOfReactants>

<listOfProducts>

<speciesReference species="c_h" stoichiometry="1" constant="false"/>

<speciesReference species="c_iasp" stoichiometry="1" constant="false"/>

<speciesReference species="c_succ" stoichiometry="1" constant="false"/>

</listOfProducts>

</reaction>

<reaction id="ASPO6" name="ASPO6" reversible="false" fast="false" compartment="cell">

<listOfReactants>

<speciesReference species="c_asp_L" stoichiometry="1" constant="false"/>

<speciesReference species="c_o2" stoichiometry="1" constant="false"/>

</listOfReactants>

<listOfProducts>

<speciesReference species="c_h" stoichiometry="1" constant="false"/>

<speciesReference species="c_h2o2" stoichiometry="1" constant="false"/>

<speciesReference species="c_iasp" stoichiometry="1" constant="false"/>

</listOfProducts>

</reaction>

<reaction id="RBFK" name="RBFK" reversible="false" fast="false" compartment="cell">

<listOfReactants>

<speciesReference species="c_atp" stoichiometry="1" constant="false"/>

<speciesReference species="c_ribflv" stoichiometry="1" constant="false"/>

</listOfReactants>

<listOfProducts>

<speciesReference species="c_adp" stoichiometry="1" constant="false"/>

<speciesReference species="c_fmn" stoichiometry="1" constant="false"/>

<speciesReference species="c_h" stoichiometry="1" constant="false"/>

</listOfProducts>

</reaction>

<reaction id="INSK" name="INSK" reversible="false" fast="false" compartment="cell">

<listOfReactants>

<speciesReference species="c_atp" stoichiometry="1" constant="false"/>

<speciesReference species="c_ins" stoichiometry="1" constant="false"/>

</listOfReactants>

<listOfProducts>

<speciesReference species="c_adp" stoichiometry="1" constant="false"/>

<speciesReference species="c_h" stoichiometry="1" constant="false"/>

<speciesReference species="c_imp" stoichiometry="1" constant="false"/>

</listOfProducts>

</reaction>

<reaction id="VALDHr" name="VALDHr" reversible="true" fast="false" compartment="cell">

<listOfReactants>

<speciesReference species="c_h2o" stoichiometry="1" constant="false"/>

<speciesReference species="c_nad" stoichiometry="1" constant="false"/>

<speciesReference species="c_val_L" stoichiometry="1" constant="false"/>

</listOfReactants>

<listOfProducts>

<speciesReference species="c_3mob" stoichiometry="1" constant="false"/>

<speciesReference species="c_h" stoichiometry="1" constant="false"/>

<speciesReference species="c_nadh" stoichiometry="1" constant="false"/>

<speciesReference species="c_nh4" stoichiometry="1" constant="false"/>

</listOfProducts>

</reaction>

<reaction id="DURIK1" name="DURIK1" reversible="false" fast="false" compartment="cell">

<listOfReactants>

<speciesReference species="c_atp" stoichiometry="1" constant="false"/>

<speciesReference species="c_duri" stoichiometry="1" constant="false"/>

</listOfReactants>

<listOfProducts>

<speciesReference species="c_adp" stoichiometry="1" constant="false"/>

<speciesReference species="c_dump" stoichiometry="1" constant="false"/>

<speciesReference species="c_h" stoichiometry="1" constant="false"/>

</listOfProducts>

</reaction>

<reaction id="ALAD_L" name="ALAD_L" reversible="false" fast="false" compartment="cell">

<listOfReactants>

<speciesReference species="c_ala_L" stoichiometry="1" constant="false"/>

<speciesReference species="c_h2o" stoichiometry="1" constant="false"/>

<speciesReference species="c_nad" stoichiometry="1" constant="false"/>

</listOfReactants>

<listOfProducts>

<speciesReference species="c_h" stoichiometry="1" constant="false"/>

<speciesReference species="c_nadh" stoichiometry="1" constant="false"/>

<speciesReference species="c_nh4" stoichiometry="1" constant="false"/>

<speciesReference species="c_pyr" stoichiometry="1" constant="false"/>

</listOfProducts>

</reaction>

<reaction id="NADH12" name="NADH12" reversible="false" fast="false" compartment="cell">

<listOfReactants>

<speciesReference species="c_h" stoichiometry="1" constant="false"/>

<speciesReference species="c_nadh" stoichiometry="1" constant="false"/>

<speciesReference species="c_ubq8" stoichiometry="1" constant="false"/>

</listOfReactants>

<listOfProducts>

<speciesReference species="c_nad" stoichiometry="1" constant="false"/>

<speciesReference species="c_ubq8h2" stoichiometry="1" constant="false"/>

</listOfProducts>

</reaction>

<reaction id="DHORTS" name="DHORTS" reversible="true" fast="false" compartment="cell">

<listOfReactants>

<speciesReference species="c_dhor_S" stoichiometry="1" constant="false"/>

<speciesReference species="c_h2o" stoichiometry="1" constant="false"/>

</listOfReactants>

<listOfProducts>

<speciesReference species="c_cbasp" stoichiometry="1" constant="false"/>

<speciesReference species="c_h" stoichiometry="1" constant="false"/>

</listOfProducts>

</reaction>

<reaction id="PHEAL" name="PHEAL" reversible="false" fast="false" compartment="cell">

<listOfReactants>

<speciesReference species="c_phe_L" stoichiometry="1" constant="false"/>

</listOfReactants>

<listOfProducts>

<speciesReference species="c_cinnm" stoichiometry="1" constant="false"/>

<speciesReference species="c_nh4" stoichiometry="1" constant="false"/>

</listOfProducts>

</reaction>

<reaction id="NADH14" name="NADH14" reversible="false" fast="false" compartment="cell">

<listOfReactants>

<speciesReference species="c_h" stoichiometry="1" constant="false"/>

<speciesReference species="c_mmqn7" stoichiometry="1" constant="false"/>

<speciesReference species="c_nadh" stoichiometry="1" constant="false"/>

</listOfReactants>

<listOfProducts>

<speciesReference species="c_mmql7" stoichiometry="1" constant="false"/>

<speciesReference species="c_nad" stoichiometry="1" constant="false"/>

</listOfProducts>

</reaction>

<reaction id="PGPPH" name="PGPPH" reversible="false" fast="false" compartment="cell">

<listOfReactants>

<speciesReference species="c_h2o" stoichiometry="1" constant="false"/>

<speciesReference species="c_pglyp" stoichiometry="1" constant="false"/>

</listOfReactants>

<listOfProducts>

<speciesReference species="c_pgly" stoichiometry="1" constant="false"/>

<speciesReference species="c_pi" stoichiometry="1" constant="false"/>

</listOfProducts>

</reaction>

<reaction id="PPA" name="PPA" reversible="false" fast="false" compartment="cell">

<listOfReactants>

<speciesReference species="c_h2o" stoichiometry="1" constant="false"/>

<speciesReference species="c_ppi" stoichiometry="1" constant="false"/>

</listOfReactants>

<listOfProducts>

<speciesReference species="c_h" stoichiometry="1" constant="false"/>

<speciesReference species="c_pi" stoichiometry="2" constant="false"/>

</listOfProducts>

</reaction>

<reaction id="SOD" name="SOD" reversible="false" fast="false" compartment="cell">

<listOfReactants>

<speciesReference species="c_h" stoichiometry="2" constant="false"/>

<speciesReference species="c_o2_" stoichiometry="2" constant="false"/>

</listOfReactants>

<listOfProducts>

<speciesReference species="c_h2o2" stoichiometry="1" constant="false"/>

<speciesReference species="c_o2" stoichiometry="1" constant="false"/>

</listOfProducts>

</reaction>

<reaction id="PRMICIi" name="PRMICIi" reversible="false" fast="false" compartment="cell">

<listOfReactants>

<speciesReference species="c_prfp" stoichiometry="1" constant="false"/>

</listOfReactants>

<listOfProducts>

<speciesReference species="c_prlp" stoichiometry="1" constant="false"/>

</listOfProducts>

</reaction>

<reaction id="METTRS" name="METTRS" reversible="false" fast="false" compartment="cell">

<listOfReactants>

<speciesReference species="c_atp" stoichiometry="1" constant="false"/>

<speciesReference species="c_met_L" stoichiometry="1" constant="false"/>

<speciesReference species="c_trnamet" stoichiometry="1" constant="false"/>

</listOfReactants>

<listOfProducts>

<speciesReference species="c_amp" stoichiometry="1" constant="false"/>

<speciesReference species="c_mettrna" stoichiometry="1" constant="false"/>

<speciesReference species="c_ppi" stoichiometry="1" constant="false"/>

</listOfProducts>

</reaction>

<reaction id="PPM" name="PPM" reversible="true" fast="false" compartment="cell">

<listOfReactants>

<speciesReference species="c_r1p" stoichiometry="1" constant="false"/>

</listOfReactants>

<listOfProducts>

<speciesReference species="c_r5p" stoichiometry="1" constant="false"/>

</listOfProducts>

</reaction>

<reaction id="DHPRx" name="DHPRx" reversible="true" fast="false" compartment="cell">

<listOfReactants>

<speciesReference species="c_dhbpt" stoichiometry="1" constant="false"/>

<speciesReference species="c_h" stoichiometry="1" constant="false"/>

<speciesReference species="c_nadh" stoichiometry="1" constant="false"/>

</listOfReactants>

<listOfProducts>

<speciesReference species="c_nad" stoichiometry="1" constant="false"/>

<speciesReference species="c_thbpt" stoichiometry="1" constant="false"/>

</listOfProducts>

</reaction>

<reaction id="PPS" name="PPS" reversible="false" fast="false" compartment="cell">

<listOfReactants>

<speciesReference species="c_atp" stoichiometry="1" constant="false"/>

<speciesReference species="c_h2o" stoichiometry="1" constant="false"/>

<speciesReference species="c_pyr" stoichiometry="1" constant="false"/>

</listOfReactants>

<listOfProducts>

<speciesReference species="c_amp" stoichiometry="1" constant="false"/>

<speciesReference species="c_h" stoichiometry="2" constant="false"/>

<speciesReference species="c_pep" stoichiometry="1" constant="false"/>

<speciesReference species="c_pi" stoichiometry="1" constant="false"/>

</listOfProducts>

</reaction>

<reaction id="CONFALDD" name="CONFALDD" reversible="false" fast="false" compartment="cell">

<listOfReactants>

<speciesReference species="c_confald" stoichiometry="1" constant="false"/>

<speciesReference species="c_h2o" stoichiometry="1" constant="false"/>

<speciesReference species="c_nad" stoichiometry="1" constant="false"/>

</listOfReactants>

<listOfProducts>

<speciesReference species="c_frlt" stoichiometry="1" constant="false"/>

<speciesReference species="c_h" stoichiometry="2" constant="false"/>

<speciesReference species="c_nadh" stoichiometry="1" constant="false"/>

</listOfProducts>

</reaction>

<reaction id="SELNPS" name="SELNPS" reversible="false" fast="false" compartment="cell">

<listOfReactants>

<speciesReference species="c_atp" stoichiometry="1" constant="false"/>

<speciesReference species="c_h2o" stoichiometry="1" constant="false"/>

<speciesReference species="c_seln" stoichiometry="1" constant="false"/>

</listOfReactants>

<listOfProducts>

<speciesReference species="c_amp" stoichiometry="1" constant="false"/>

<speciesReference species="c_pi" stoichiometry="1" constant="false"/>

<speciesReference species="c_selnp" stoichiometry="1" constant="false"/>

</listOfProducts>

</reaction>

<reaction id="PLIPPG_SO" name="PLIPPG_SO" reversible="false" fast="false" compartment="cell">

<listOfReactants>

<speciesReference species="c_h2o" stoichiometry="1" constant="false"/>

<speciesReference species="c_pgly" stoichiometry="1" constant="false"/>

</listOfReactants>

<listOfProducts>

<speciesReference species="c_agpg" stoichiometry="1" constant="false"/>

<speciesReference species="c_fa1" stoichiometry="0.06" constant="false"/>

<speciesReference species="c_fa11" stoichiometry="0.065" constant="false"/>

<speciesReference species="c_fa13" stoichiometry="0.035" constant="false"/>

<speciesReference species="c_fa3" stoichiometry="0.165" constant="false"/>

<speciesReference species="c_fa6" stoichiometry="0.075" constant="false"/>

<speciesReference species="c_h" stoichiometry="1" constant="false"/>

<speciesReference species="c_hdca" stoichiometry="0.065" constant="false"/>

<speciesReference species="c_hdcea" stoichiometry="0.19" constant="false"/>

<speciesReference species="c_hpdca" stoichiometry="0.015" constant="false"/>

<speciesReference species="c_hpde" stoichiometry="0.125" constant="false"/>

<speciesReference species="c_ocdca" stoichiometry="0.01" constant="false"/>

<speciesReference species="c_ocdcea" stoichiometry="0.125" constant="false"/>

<speciesReference species="c_pde" stoichiometry="0.06" constant="false"/>

<speciesReference species="c_ptdca" stoichiometry="0.01" constant="false"/>

</listOfProducts>

</reaction>

<reaction id="GPDDA4" name="GPDDA4" reversible="false" fast="false" compartment="cell">

<listOfReactants>

<speciesReference species="c_g3pg" stoichiometry="1" constant="false"/>

<speciesReference species="c_h2o" stoichiometry="1" constant="false"/>

</listOfReactants>

<listOfProducts>

<speciesReference species="c_glyc" stoichiometry="1" constant="false"/>

<speciesReference species="c_glyc3p" stoichiometry="1" constant="false"/>

<speciesReference species="c_h" stoichiometry="1" constant="false"/>

</listOfProducts>

</reaction>

<reaction id="PGLYCP" name="PGLYCP" reversible="false" fast="false" compartment="cell">

<listOfReactants>

<speciesReference species="c_2pglyc" stoichiometry="1" constant="false"/>

<speciesReference species="c_h2o" stoichiometry="1" constant="false"/>

</listOfReactants>

<listOfProducts>

<speciesReference species="c_glyclt" stoichiometry="1" constant="false"/>

<speciesReference species="c_pi" stoichiometry="1" constant="false"/>

</listOfProducts>

</reaction>

<reaction id="GPDDA2" name="GPDDA2" reversible="false" fast="false" compartment="cell">

<listOfReactants>

<speciesReference species="c_g3pe" stoichiometry="1" constant="false"/>

<speciesReference species="c_h2o" stoichiometry="1" constant="false"/>

</listOfReactants>

<listOfProducts>

<speciesReference species="c_etha" stoichiometry="1" constant="false"/>

<speciesReference species="c_glyc3p" stoichiometry="1" constant="false"/>

<speciesReference species="c_h" stoichiometry="1" constant="false"/>

</listOfProducts>

</reaction>

<reaction id="GGLUGABH" name="GGLUGABH" reversible="false" fast="false" compartment="cell">

<listOfReactants>

<speciesReference species="c_ggluabt" stoichiometry="1" constant="false"/>

<speciesReference species="c_h2o" stoichiometry="1" constant="false"/>

</listOfReactants>

<listOfProducts>

<speciesReference species="c_4abut" stoichiometry="1" constant="false"/>

<speciesReference species="c_glu_L" stoichiometry="1" constant="false"/>

</listOfProducts>

</reaction>

<reaction id="HXAD" name="HXAD" reversible="false" fast="false" compartment="cell">

<listOfReactants>

<speciesReference species="c_chitob" stoichiometry="1" constant="false"/>

<speciesReference species="c_h2o" stoichiometry="1" constant="false"/>

</listOfReactants>

<listOfProducts>

<speciesReference species="c_acgam" stoichiometry="2" constant="false"/>

</listOfProducts>

</reaction>

<reaction id="IPPS" name="IPPS" reversible="false" fast="false" compartment="cell">

<listOfReactants>

<speciesReference species="c_3mob" stoichiometry="1" constant="false"/>

<speciesReference species="c_accoa" stoichiometry="1" constant="false"/>

<speciesReference species="c_h2o" stoichiometry="1" constant="false"/>

</listOfReactants>

<listOfProducts>

<speciesReference species="c_3c3hmp" stoichiometry="1" constant="false"/>

<speciesReference species="c_coa" stoichiometry="1" constant="false"/>

<speciesReference species="c_h" stoichiometry="1" constant="false"/>

</listOfProducts>

</reaction>

<reaction id="MCCC" name="MCCC" reversible="false" fast="false" compartment="cell">

<listOfReactants>

<speciesReference species="c_3mb2coa" stoichiometry="1" constant="false"/>

<speciesReference species="c_atp" stoichiometry="1" constant="false"/>

<speciesReference species="c_hco3" stoichiometry="1" constant="false"/>

</listOfReactants>

<listOfProducts>

<speciesReference species="c_3mgcoa" stoichiometry="1" constant="false"/>

<speciesReference species="c_adp" stoichiometry="1" constant="false"/>

<speciesReference species="c_h" stoichiometry="1" constant="false"/>

<speciesReference species="c_pi" stoichiometry="1" constant="false"/>

</listOfProducts>

</reaction>

<reaction id="UDPG4E" name="UDPG4E" reversible="true" fast="false" compartment="cell">

<listOfReactants>

<speciesReference species="c_udpg" stoichiometry="1" constant="false"/>

</listOfReactants>

<listOfProducts>

<speciesReference species="c_udpgal" stoichiometry="1" constant="false"/>

</listOfProducts>

</reaction>

<reaction id="GBEZ" name="GBEZ" reversible="false" fast="false" compartment="cell">

<listOfReactants>

<speciesReference species="c_atp" stoichiometry="1" constant="false"/>

<speciesReference species="c_glc_D" stoichiometry="1" constant="false"/>

</listOfReactants>

<listOfProducts>

<speciesReference species="c_adp" stoichiometry="1" constant="false"/>

<speciesReference species="c_glycogen" stoichiometry="1" constant="false"/>

<speciesReference species="c_h" stoichiometry="1" constant="false"/>

<speciesReference species="c_pi" stoichiometry="1" constant="false"/>

</listOfProducts>

</reaction>

<reaction id="AOXS" name="AOXS" reversible="true" fast="false" compartment="cell">

<listOfReactants>

<speciesReference species="c_ala_L" stoichiometry="1" constant="false"/>

<speciesReference species="c_h" stoichiometry="1" constant="false"/>

<speciesReference species="c_pmcoa" stoichiometry="1" constant="false"/>

</listOfReactants>

<listOfProducts>

<speciesReference species="c_8aonn" stoichiometry="1" constant="false"/>

<speciesReference species="c_co2" stoichiometry="1" constant="false"/>

<speciesReference species="c_coa" stoichiometry="1" constant="false"/>

</listOfProducts>

</reaction>

<reaction id="DADK" name="DADK" reversible="true" fast="false" compartment="cell">

<listOfReactants>

<speciesReference species="c_atp" stoichiometry="1" constant="false"/>

<speciesReference species="c_damp" stoichiometry="1" constant="false"/>

</listOfReactants>

<listOfProducts>

<speciesReference species="c_adp" stoichiometry="1" constant="false"/>

<speciesReference species="c_dadp" stoichiometry="1" constant="false"/>

</listOfProducts>

</reaction>

<reaction id="SDPTA" name="SDPTA" reversible="true" fast="false" compartment="cell">

<listOfReactants>

<speciesReference species="c_akg" stoichiometry="1" constant="false"/>

<speciesReference species="c_sl26da" stoichiometry="1" constant="false"/>

</listOfReactants>

<listOfProducts>

<speciesReference species="c_glu_L" stoichiometry="1" constant="false"/>

<speciesReference species="c_sl2a6o" stoichiometry="1" constant="false"/>

</listOfProducts>

</reaction>

<reaction id="NNDMBRT2" name="NNDMBRT2" reversible="false" fast="false" compartment="cell">

<listOfReactants>

<speciesReference species="c_ribflv" stoichiometry="1" constant="false"/>

</listOfReactants>

<listOfProducts>

<speciesReference species="c_dmbzid" stoichiometry="1" constant="false"/>

<speciesReference species="c_unknown1" stoichiometry="1" constant="false"/>

</listOfProducts>

</reaction>

<reaction id="DADA" name="DADA" reversible="false" fast="false" compartment="cell">

<listOfReactants>

<speciesReference species="c_dad_2" stoichiometry="1" constant="false"/>

<speciesReference species="c_h" stoichiometry="1" constant="false"/>

<speciesReference species="c_h2o" stoichiometry="1" constant="false"/>

</listOfReactants>

<listOfProducts>

<speciesReference species="c_din" stoichiometry="1" constant="false"/>

<speciesReference species="c_nh4" stoichiometry="1" constant="false"/>

</listOfProducts>

</reaction>

<reaction id="BPNT" name="BPNT" reversible="false" fast="false" compartment="cell">

<listOfReactants>

<speciesReference species="c_h2o" stoichiometry="1" constant="false"/>

<speciesReference species="c_pap" stoichiometry="1" constant="false"/>

</listOfReactants>

<listOfProducts>

<speciesReference species="c_amp" stoichiometry="1" constant="false"/>

<speciesReference species="c_pi" stoichiometry="1" constant="false"/>

</listOfProducts>

</reaction>

<reaction id="TDSK" name="TDSK" reversible="false" fast="false" compartment="cell">

<listOfReactants>

<speciesReference species="c_atp" stoichiometry="1" constant="false"/>

<speciesReference species="c_lipidAds" stoichiometry="1" constant="false"/>

</listOfReactants>

<listOfProducts>

<speciesReference species="c_adp" stoichiometry="1" constant="false"/>

<speciesReference species="c_h" stoichiometry="1" constant="false"/>

<speciesReference species="c_lipidA" stoichiometry="1" constant="false"/>

</listOfProducts>

</reaction>

<reaction id="FMNRx" name="FMNRx" reversible="false" fast="false" compartment="cell">

<listOfReactants>

<speciesReference species="c_fmn" stoichiometry="1" constant="false"/>

<speciesReference species="c_h" stoichiometry="1" constant="false"/>

<speciesReference species="c_nadh" stoichiometry="1" constant="false"/>

</listOfReactants>

<listOfProducts>

<speciesReference species="c_fmnRD" stoichiometry="1" constant="false"/>

<speciesReference species="c_nad" stoichiometry="1" constant="false"/>

</listOfProducts>

</reaction>

<reaction id="ACOAD8" name="ACOAD8" reversible="false" fast="false" compartment="cell">

<listOfReactants>

<speciesReference species="c_ivcoa" stoichiometry="1" constant="false"/>

<speciesReference species="c_nad" stoichiometry="1" constant="false"/>

</listOfReactants>

<listOfProducts>

<speciesReference species="c_3mb2coa" stoichiometry="1" constant="false"/>

<speciesReference species="c_h" stoichiometry="1" constant="false"/>

<speciesReference species="c_nadh" stoichiometry="1" constant="false"/>

</listOfProducts>

</reaction>

<reaction id="TMDPP" name="TMDPP" reversible="true" fast="false" compartment="cell">

<listOfReactants>

<speciesReference species="c_pi" stoichiometry="1" constant="false"/>

<speciesReference species="c_thymd" stoichiometry="1" constant="false"/>

</listOfReactants>

<listOfProducts>

<speciesReference species="c_2dr1p" stoichiometry="1" constant="false"/>

<speciesReference species="c_thym" stoichiometry="1" constant="false"/>

</listOfProducts>

</reaction>

<reaction id="CBLAT" name="CBLAT" reversible="true" fast="false" compartment="cell">

<listOfReactants>

<speciesReference species="c_atp" stoichiometry="1" constant="false"/>

<speciesReference species="c_cbl1" stoichiometry="1" constant="false"/>

<speciesReference species="c_h2o" stoichiometry="1" constant="false"/>

</listOfReactants>

<listOfProducts>

<speciesReference species="c_cobamcoa" stoichiometry="1" constant="false"/>

<speciesReference species="c_pi" stoichiometry="1" constant="false"/>

<speciesReference species="c_ppi" stoichiometry="1" constant="false"/>

</listOfProducts>

</reaction>

<reaction id="PROTRS" name="PROTRS" reversible="false" fast="false" compartment="cell">

<listOfReactants>

<speciesReference species="c_atp" stoichiometry="1" constant="false"/>

<speciesReference species="c_pro_L" stoichiometry="1" constant="false"/>

<speciesReference species="c_trnapro" stoichiometry="1" constant="false"/>

</listOfReactants>

<listOfProducts>

<speciesReference species="c_amp" stoichiometry="1" constant="false"/>

<speciesReference species="c_ppi" stoichiometry="1" constant="false"/>

<speciesReference species="c_protrna" stoichiometry="1" constant="false"/>

</listOfProducts>

</reaction>

<reaction id="GLYTRS" name="GLYTRS" reversible="false" fast="false" compartment="cell">

<listOfReactants>

<speciesReference species="c_atp" stoichiometry="1" constant="false"/>

<speciesReference species="c_gly" stoichiometry="1" constant="false"/>

<speciesReference species="c_trnagly" stoichiometry="1" constant="false"/>

</listOfReactants>

<listOfProducts>

<speciesReference species="c_amp" stoichiometry="1" constant="false"/>

<speciesReference species="c_glytrna" stoichiometry="1" constant="false"/>

<speciesReference species="c_ppi" stoichiometry="1" constant="false"/>

</listOfProducts>

</reaction>

<reaction id="CMLDC" name="CMLDC" reversible="false" fast="false" compartment="cell">

<listOfReactants>

<speciesReference species="c_2c25dho" stoichiometry="1" constant="false"/>

<speciesReference species="c_h" stoichiometry="1" constant="false"/>

</listOfReactants>

<listOfProducts>

<speciesReference species="c_5odhf2a" stoichiometry="1" constant="false"/>

<speciesReference species="c_co2" stoichiometry="1" constant="false"/>

</listOfProducts>

</reaction>

<reaction id="AHSERL" name="AHSERL" reversible="false" fast="false" compartment="cell">

<listOfReactants>

<speciesReference species="c_achms" stoichiometry="1" constant="false"/>

<speciesReference species="c_ch4s" stoichiometry="1" constant="false"/>

</listOfReactants>

<listOfProducts>

<speciesReference species="c_ac" stoichiometry="1" constant="false"/>

<speciesReference species="c_h" stoichiometry="1" constant="false"/>

<speciesReference species="c_met_L" stoichiometry="1" constant="false"/>

</listOfProducts>

</reaction>

<reaction id="NTPP1" name="NTPP1" reversible="false" fast="false" compartment="cell">

<listOfReactants>

<speciesReference species="c_dgtp" stoichiometry="1" constant="false"/>

<speciesReference species="c_h2o" stoichiometry="1" constant="false"/>

</listOfReactants>

<listOfProducts>

<speciesReference species="c_dgmp" stoichiometry="1" constant="false"/>

<speciesReference species="c_h" stoichiometry="1" constant="false"/>

<speciesReference species="c_ppi" stoichiometry="1" constant="false"/>

</listOfProducts>

</reaction>

<reaction id="PSERT" name="PSERT" reversible="false" fast="false" compartment="cell">

<listOfReactants>

<speciesReference species="c_3php" stoichiometry="1" constant="false"/>

<speciesReference species="c_glu_L" stoichiometry="1" constant="false"/>

</listOfReactants>

<listOfProducts>

<speciesReference species="c_akg" stoichiometry="1" constant="false"/>

<speciesReference species="c_pser_L" stoichiometry="1" constant="false"/>

</listOfProducts>

</reaction>

<reaction id="GLNS" name="GLNS" reversible="false" fast="false" compartment="cell">

<listOfReactants>

<speciesReference species="c_atp" stoichiometry="1" constant="false"/>

<speciesReference species="c_glu_L" stoichiometry="1" constant="false"/>

<speciesReference species="c_nh4" stoichiometry="1" constant="false"/>

</listOfReactants>

<listOfProducts>

<speciesReference species="c_adp" stoichiometry="1" constant="false"/>

<speciesReference species="c_gln_L" stoichiometry="1" constant="false"/>

<speciesReference species="c_h" stoichiometry="1" constant="false"/>

<speciesReference species="c_pi" stoichiometry="1" constant="false"/>

</listOfProducts>

</reaction>

<reaction id="PYNP2" name="PYNP2" reversible="true" fast="false" compartment="cell">

<listOfReactants>

<speciesReference species="c_pi" stoichiometry="1" constant="false"/>

<speciesReference species="c_uri" stoichiometry="1" constant="false"/>

</listOfReactants>

<listOfProducts>

<speciesReference species="c_r1p" stoichiometry="1" constant="false"/>

<speciesReference species="c_ura" stoichiometry="1" constant="false"/>

</listOfProducts>

</reaction>

<reaction id="AICART" name="AICART" reversible="true" fast="false" compartment="cell">

<listOfReactants>

<speciesReference species="c_10fthf" stoichiometry="1" constant="false"/>

<speciesReference species="c_aicar" stoichiometry="1" constant="false"/>

</listOfReactants>

<listOfProducts>

<speciesReference species="c_fprica" stoichiometry="1" constant="false"/>

<speciesReference species="c_thf" stoichiometry="1" constant="false"/>

</listOfProducts>

</reaction>

<reaction id="LPADSS" name="LPADSS" reversible="false" fast="false" compartment="cell">

<listOfReactants>

<speciesReference species="c_lipidX" stoichiometry="1" constant="false"/>

<speciesReference species="c_u23ga" stoichiometry="1" constant="false"/>

</listOfReactants>

<listOfProducts>

<speciesReference species="c_h" stoichiometry="1" constant="false"/>

<speciesReference species="c_lipidAds" stoichiometry="1" constant="false"/>

<speciesReference species="c_udp" stoichiometry="1" constant="false"/>

</listOfProducts>

</reaction>

<reaction id="NNDPR" name="NNDPR" reversible="false" fast="false" compartment="cell">

<listOfReactants>

<speciesReference species="c_h" stoichiometry="2" constant="false"/>

<speciesReference species="c_prpp" stoichiometry="1" constant="false"/>

<speciesReference species="c_quln" stoichiometry="1" constant="false"/>

</listOfReactants>

<listOfProducts>

<speciesReference species="c_co2" stoichiometry="1" constant="false"/>

<speciesReference species="c_nicrnt" stoichiometry="1" constant="false"/>

<speciesReference species="c_ppi" stoichiometry="1" constant="false"/>

</listOfProducts>

</reaction>

<reaction id="HMGDx" name="HMGDx" reversible="false" fast="false" compartment="cell">

<listOfReactants>

<speciesReference species="c_hmglut_S" stoichiometry="1" constant="false"/>

<speciesReference species="c_nad" stoichiometry="1" constant="false"/>

</listOfReactants>

<listOfProducts>

<speciesReference species="c_fglut_S" stoichiometry="1" constant="false"/>

<speciesReference species="c_h" stoichiometry="1" constant="false"/>

<speciesReference species="c_nadh" stoichiometry="1" constant="false"/>

</listOfProducts>

</reaction>

<reaction id="NADS1" name="NADS1" reversible="false" fast="false" compartment="cell">

<listOfReactants>

<speciesReference species="c_atp" stoichiometry="1" constant="false"/>

<speciesReference species="c_dnad" stoichiometry="1" constant="false"/>

<speciesReference species="c_nh4" stoichiometry="1" constant="false"/>

</listOfReactants>

<listOfProducts>

<speciesReference species="c_amp" stoichiometry="1" constant="false"/>

<speciesReference species="c_h" stoichiometry="1" constant="false"/>

<speciesReference species="c_nad" stoichiometry="1" constant="false"/>

<speciesReference species="c_ppi" stoichiometry="1" constant="false"/>

</listOfProducts>

</reaction>

<reaction id="OPHHX" name="OPHHX" reversible="false" fast="false" compartment="cell">

<listOfReactants>

<speciesReference species="c_2oph" stoichiometry="1" constant="false"/>

<speciesReference species="c_o2" stoichiometry="0.5" constant="false"/>

</listOfReactants>

<listOfProducts>

<speciesReference species="c_2ohph" stoichiometry="1" constant="false"/>

</listOfProducts>

</reaction>

<reaction id="RNTR4" name="RNTR4" reversible="false" fast="false" compartment="cell">

<listOfReactants>

<speciesReference species="c_trdrd" stoichiometry="1" constant="false"/>

<speciesReference species="c_utp" stoichiometry="1" constant="false"/>

</listOfReactants>

<listOfProducts>

<speciesReference species="c_dutp" stoichiometry="1" constant="false"/>

<speciesReference species="c_h2o" stoichiometry="1" constant="false"/>

<speciesReference species="c_trdox" stoichiometry="1" constant="false"/>

</listOfProducts>

</reaction>

<reaction id="RNTR3" name="RNTR3" reversible="false" fast="false" compartment="cell">

<listOfReactants>

<speciesReference species="c_ctp" stoichiometry="1" constant="false"/>

<speciesReference species="c_trdrd" stoichiometry="1" constant="false"/>

</listOfReactants>

<listOfProducts>

<speciesReference species="c_dctp" stoichiometry="1" constant="false"/>

<speciesReference species="c_h2o" stoichiometry="1" constant="false"/>

<speciesReference species="c_trdox" stoichiometry="1" constant="false"/>

</listOfProducts>

</reaction>

<reaction id="RNTR2" name="RNTR2" reversible="false" fast="false" compartment="cell">

<listOfReactants>

<speciesReference species="c_gtp" stoichiometry="1" constant="false"/>

<speciesReference species="c_trdrd" stoichiometry="1" constant="false"/>

</listOfReactants>

<listOfProducts>

<speciesReference species="c_dgtp" stoichiometry="1" constant="false"/>

<speciesReference species="c_h2o" stoichiometry="1" constant="false"/>

<speciesReference species="c_trdox" stoichiometry="1" constant="false"/>

</listOfProducts>

</reaction>

<reaction id="RNTR1" name="RNTR1" reversible="false" fast="false" compartment="cell">

<listOfReactants>

<speciesReference species="c_atp" stoichiometry="1" constant="false"/>

<speciesReference species="c_trdrd" stoichiometry="1" constant="false"/>

</listOfReactants>

<listOfProducts>

<speciesReference species="c_datp" stoichiometry="1" constant="false"/>

<speciesReference species="c_h2o" stoichiometry="1" constant="false"/>

<speciesReference species="c_trdox" stoichiometry="1" constant="false"/>

</listOfProducts>

</reaction>

<reaction id="MACPD" name="MACPD" reversible="false" fast="false" compartment="cell">

<listOfReactants>

<speciesReference species="c_h" stoichiometry="1" constant="false"/>

<speciesReference species="c_malACP" stoichiometry="1" constant="false"/>

</listOfReactants>

<listOfProducts>

<speciesReference species="c_acACP" stoichiometry="1" constant="false"/>

<speciesReference species="c_co2" stoichiometry="1" constant="false"/>

</listOfProducts>

</reaction>

<reaction id="PPTT" name="PPTT" reversible="false" fast="false" compartment="cell">

<listOfReactants>

<speciesReference species="c_ipdp" stoichiometry="1" constant="false"/>

<speciesReference species="c_pendp" stoichiometry="1" constant="false"/>

</listOfReactants>

<listOfProducts>

<speciesReference species="c_hexdp" stoichiometry="1" constant="false"/>

<speciesReference species="c_ppi" stoichiometry="1" constant="false"/>

</listOfProducts>

</reaction>

<reaction id="PTPATi" name="PTPATi" reversible="false" fast="false" compartment="cell">

<listOfReactants>

<speciesReference species="c_atp" stoichiometry="1" constant="false"/>

<speciesReference species="c_h" stoichiometry="1" constant="false"/>

<speciesReference species="c_pan4p" stoichiometry="1" constant="false"/>

</listOfReactants>

<listOfProducts>

<speciesReference species="c_dpcoa" stoichiometry="1" constant="false"/>

<speciesReference species="c_ppi" stoichiometry="1" constant="false"/>

</listOfProducts>

</reaction>

<reaction id="DHORD4i" name="DHORD4i" reversible="false" fast="false" compartment="cell">

<listOfReactants>

<speciesReference species="c_dhor_S" stoichiometry="1" constant="false"/>

<speciesReference species="c_mqn7" stoichiometry="1" constant="false"/>

</listOfReactants>

<listOfProducts>

<speciesReference species="c_mql7" stoichiometry="1" constant="false"/>

<speciesReference species="c_orot" stoichiometry="1" constant="false"/>

</listOfProducts>

</reaction>

<reaction id="RPI" name="RPI" reversible="true" fast="false" compartment="cell">

<listOfReactants>

<speciesReference species="c_r5p" stoichiometry="1" constant="false"/>

</listOfReactants>

<listOfProducts>

<speciesReference species="c_ru5p_D" stoichiometry="1" constant="false"/>

</listOfProducts>

</reaction>

<reaction id="AHCYSNS" name="AHCYSNS" reversible="false" fast="false" compartment="cell">

<listOfReactants>

<speciesReference species="c_ahcys" stoichiometry="1" constant="false"/>

<speciesReference species="c_h2o" stoichiometry="1" constant="false"/>

</listOfReactants>

<listOfProducts>

<speciesReference species="c_ade" stoichiometry="1" constant="false"/>

<speciesReference species="c_rhcys" stoichiometry="1" constant="false"/>

</listOfProducts>

</reaction>

<reaction id="HMGL" name="HMGL" reversible="false" fast="false" compartment="cell">

<listOfReactants>

<speciesReference species="c_hmgcoa" stoichiometry="1" constant="false"/>

</listOfReactants>

<listOfProducts>

<speciesReference species="c_acac" stoichiometry="1" constant="false"/>

<speciesReference species="c_accoa" stoichiometry="1" constant="false"/>

</listOfProducts>

</reaction>

<reaction id="GLGC" name="GLGC" reversible="false" fast="false" compartment="cell">

<listOfReactants>

<speciesReference species="c_atp" stoichiometry="1" constant="false"/>

<speciesReference species="c_g1p" stoichiometry="1" constant="false"/>

<speciesReference species="c_h" stoichiometry="1" constant="false"/>

</listOfReactants>

<listOfProducts>

<speciesReference species="c_adpglc" stoichiometry="1" constant="false"/>

<speciesReference species="c_ppi" stoichiometry="1" constant="false"/>

</listOfProducts>

</reaction>

<reaction id="TKT2" name="TKT2" reversible="true" fast="false" compartment="cell">

<listOfReactants>

<speciesReference species="c_e4p" stoichiometry="1" constant="false"/>

<speciesReference species="c_xu5p_D" stoichiometry="1" constant="false"/>

</listOfReactants>

<listOfProducts>

<speciesReference species="c_f6p" stoichiometry="1" constant="false"/>

<speciesReference species="c_g3p" stoichiometry="1" constant="false"/>

</listOfProducts>

</reaction>

<reaction id="TKT1" name="TKT1" reversible="true" fast="false" compartment="cell">

<listOfReactants>

<speciesReference species="c_r5p" stoichiometry="1" constant="false"/>

<speciesReference species="c_xu5p_D" stoichiometry="1" constant="false"/>

</listOfReactants>

<listOfProducts>

<speciesReference species="c_g3p" stoichiometry="1" constant="false"/>

<speciesReference species="c_s7p" stoichiometry="1" constant="false"/>

</listOfProducts>

</reaction>

<reaction id="TRDR" name="TRDR" reversible="false" fast="false" compartment="cell">

<listOfReactants>

<speciesReference species="c_h" stoichiometry="1" constant="false"/>

<speciesReference species="c_nadph" stoichiometry="1" constant="false"/>

<speciesReference species="c_trdox" stoichiometry="1" constant="false"/>

</listOfReactants>

<listOfProducts>

<speciesReference species="c_nadp" stoichiometry="1" constant="false"/>

<speciesReference species="c_trdrd" stoichiometry="1" constant="false"/>

</listOfProducts>

</reaction>

<reaction id="PRO1q" name="PRO1q" reversible="false" fast="false" compartment="cell">

<listOfReactants>

<speciesReference species="c_pro_L" stoichiometry="1" constant="false"/>

<speciesReference species="c_ubq8" stoichiometry="1" constant="false"/>

</listOfReactants>

<listOfProducts>

<speciesReference species="c_1pyr5c" stoichiometry="1" constant="false"/>

<speciesReference species="c_h" stoichiometry="1" constant="false"/>

<speciesReference species="c_ubq8h2" stoichiometry="1" constant="false"/>

</listOfProducts>

</reaction>

<reaction id="PPM2" name="PPM2" reversible="true" fast="false" compartment="cell">

<listOfReactants>

<speciesReference species="c_2dr1p" stoichiometry="1" constant="false"/>

</listOfReactants>

<listOfProducts>

<speciesReference species="c_2dr5p" stoichiometry="1" constant="false"/>

</listOfProducts>

</reaction>

<reaction id="IZPN" name="IZPN" reversible="false" fast="false" compartment="cell">

<listOfReactants>

<speciesReference species="c_4izp" stoichiometry="1" constant="false"/>

<speciesReference species="c_h2o" stoichiometry="1" constant="false"/>

</listOfReactants>

<listOfProducts>

<speciesReference species="c_forglu" stoichiometry="1" constant="false"/>

</listOfProducts>

</reaction>

<reaction id="LGTHL" name="LGTHL" reversible="false" fast="false" compartment="cell">

<listOfReactants>

<speciesReference species="c_gthrd" stoichiometry="1" constant="false"/>

<speciesReference species="c_mthgxl" stoichiometry="1" constant="false"/>

</listOfReactants>

<listOfProducts>

<speciesReference species="c_lgt_S" stoichiometry="1" constant="false"/>

</listOfProducts>

</reaction>

<reaction id="RNDR2" name="RNDR2" reversible="false" fast="false" compartment="cell">

<listOfReactants>

<speciesReference species="c_gdp" stoichiometry="1" constant="false"/>

<speciesReference species="c_trdrd" stoichiometry="1" constant="false"/>

</listOfReactants>

<listOfProducts>

<speciesReference species="c_dgdp" stoichiometry="1" constant="false"/>

<speciesReference species="c_h2o" stoichiometry="1" constant="false"/>

<speciesReference species="c_trdox" stoichiometry="1" constant="false"/>

</listOfProducts>

</reaction>

<reaction id="RNDR1" name="RNDR1" reversible="false" fast="false" compartment="cell">

<listOfReactants>

<speciesReference species="c_adp" stoichiometry="1" constant="false"/>

<speciesReference species="c_trdrd" stoichiometry="1" constant="false"/>

</listOfReactants>

<listOfProducts>

<speciesReference species="c_dadp" stoichiometry="1" constant="false"/>

<speciesReference species="c_h2o" stoichiometry="1" constant="false"/>

<speciesReference species="c_trdox" stoichiometry="1" constant="false"/>

</listOfProducts>

</reaction>

<reaction id="HEXTT" name="HEXTT" reversible="false" fast="false" compartment="cell">

<listOfReactants>

<speciesReference species="c_hexdp" stoichiometry="1" constant="false"/>

<speciesReference species="c_ipdp" stoichiometry="1" constant="false"/>

</listOfReactants>

<listOfProducts>

<speciesReference species="c_hepdp" stoichiometry="1" constant="false"/>

<speciesReference species="c_ppi" stoichiometry="1" constant="false"/>

</listOfProducts>

</reaction>

<reaction id="C150ISN" name="C150ISN" reversible="false" fast="false" compartment="cell">

<listOfReactants>

<speciesReference species="c_fa7ACP" stoichiometry="1" constant="false"/>

<speciesReference species="c_h" stoichiometry="12" constant="false"/>

<speciesReference species="c_malACP" stoichiometry="4" constant="false"/>

<speciesReference species="c_nadph" stoichiometry="8" constant="false"/>

</listOfReactants>

<listOfProducts>

<speciesReference species="c_ACP" stoichiometry="4" constant="false"/>

<speciesReference species="c_co2" stoichiometry="4" constant="false"/>

<speciesReference species="c_fa3ACP" stoichiometry="1" constant="false"/>

<speciesReference species="c_h2o" stoichiometry="4" constant="false"/>

<speciesReference species="c_nadp" stoichiometry="8" constant="false"/>

</listOfProducts>

</reaction>

<reaction id="METS" name="METS" reversible="false" fast="false" compartment="cell">

<listOfReactants>

<speciesReference species="c_5mthf" stoichiometry="1" constant="false"/>

<speciesReference species="c_hcys_L" stoichiometry="1" constant="false"/>

</listOfReactants>

<listOfProducts>

<speciesReference species="c_h" stoichiometry="1" constant="false"/>

<speciesReference species="c_met_L" stoichiometry="1" constant="false"/>

<speciesReference species="c_thf" stoichiometry="1" constant="false"/>

</listOfProducts>

</reaction>

<reaction id="LEUTRS" name="LEUTRS" reversible="false" fast="false" compartment="cell">

<listOfReactants>

<speciesReference species="c_atp" stoichiometry="1" constant="false"/>

<speciesReference species="c_leu_L" stoichiometry="1" constant="false"/>

<speciesReference species="c_trnaleu" stoichiometry="1" constant="false"/>

</listOfReactants>

<listOfProducts>

<speciesReference species="c_amp" stoichiometry="1" constant="false"/>

<speciesReference species="c_leutrna" stoichiometry="1" constant="false"/>

<speciesReference species="c_ppi" stoichiometry="1" constant="false"/>

</listOfProducts>

</reaction>

<reaction id="SERAT" name="SERAT" reversible="true" fast="false" compartment="cell">

<listOfReactants>

<speciesReference species="c_accoa" stoichiometry="1" constant="false"/>

<speciesReference species="c_ser_L" stoichiometry="1" constant="false"/>

</listOfReactants>

<listOfProducts>

<speciesReference species="c_acser" stoichiometry="1" constant="false"/>

<speciesReference species="c_coa" stoichiometry="1" constant="false"/>

</listOfProducts>

</reaction>

<reaction id="SLCYSS" name="SLCYSS" reversible="false" fast="false" compartment="cell">

<listOfReactants>

<speciesReference species="c_acser" stoichiometry="1" constant="false"/>

<speciesReference species="c_tsul" stoichiometry="1" constant="false"/>

</listOfReactants>

<listOfProducts>

<speciesReference species="c_ac" stoichiometry="1" constant="false"/>

<speciesReference species="c_slcys" stoichiometry="1" constant="false"/>

</listOfProducts>

</reaction>

<reaction id="GLUR" name="GLUR" reversible="true" fast="false" compartment="cell">

<listOfReactants>

<speciesReference species="c_glu_D" stoichiometry="1" constant="false"/>

</listOfReactants>

<listOfProducts>

<speciesReference species="c_glu_L" stoichiometry="1" constant="false"/>

</listOfProducts>

</reaction>

<reaction id="ALAALA" name="ALAALA" reversible="true" fast="false" compartment="cell">

<listOfReactants>

<speciesReference species="c_ala_D" stoichiometry="2" constant="false"/>

<speciesReference species="c_atp" stoichiometry="1" constant="false"/>

</listOfReactants>

<listOfProducts>

<speciesReference species="c_adp" stoichiometry="1" constant="false"/>

<speciesReference species="c_alaala" stoichiometry="1" constant="false"/>

<speciesReference species="c_h" stoichiometry="1" constant="false"/>

<speciesReference species="c_pi" stoichiometry="1" constant="false"/>

</listOfProducts>

</reaction>

<reaction id="GLUN" name="GLUN" reversible="false" fast="false" compartment="cell">

<listOfReactants>

<speciesReference species="c_gln_L" stoichiometry="1" constant="false"/>

<speciesReference species="c_h2o" stoichiometry="1" constant="false"/>

</listOfReactants>

<listOfProducts>

<speciesReference species="c_glu_L" stoichiometry="1" constant="false"/>

<speciesReference species="c_nh4" stoichiometry="1" constant="false"/>

</listOfProducts>

</reaction>

<reaction id="GLYCKb" name="GLYCKb" reversible="false" fast="false" compartment="cell">

<listOfReactants>

<speciesReference species="c_atp" stoichiometry="1" constant="false"/>

<speciesReference species="c_glyc_R" stoichiometry="1" constant="false"/>

</listOfReactants>

<listOfProducts>

<speciesReference species="c_2pg" stoichiometry="1" constant="false"/>

<speciesReference species="c_adp" stoichiometry="1" constant="false"/>

<speciesReference species="c_h" stoichiometry="1" constant="false"/>

</listOfProducts>

</reaction>

<reaction id="CHRPL" name="CHRPL" reversible="false" fast="false" compartment="cell">

<listOfReactants>

<speciesReference species="c_chor" stoichiometry="1" constant="false"/>

</listOfReactants>

<listOfProducts>

<speciesReference species="c_4hbz" stoichiometry="1" constant="false"/>

<speciesReference species="c_pyr" stoichiometry="1" constant="false"/>

</listOfProducts>

</reaction>

<reaction id="PGMT" name="PGMT" reversible="true" fast="false" compartment="cell">

<listOfReactants>

<speciesReference species="c_g1p" stoichiometry="1" constant="false"/>

</listOfReactants>

<listOfProducts>

<speciesReference species="c_g6p" stoichiometry="1" constant="false"/>

</listOfProducts>

</reaction>

<reaction id="THRA" name="THRA" reversible="true" fast="false" compartment="cell">

<listOfReactants>

<speciesReference species="c_thr_L" stoichiometry="1" constant="false"/>

</listOfReactants>

<listOfProducts>

<speciesReference species="c_acald" stoichiometry="1" constant="false"/>

<speciesReference species="c_gly" stoichiometry="1" constant="false"/>

</listOfProducts>

</reaction>

<reaction id="NTD9" name="NTD9" reversible="false" fast="false" compartment="cell">

<listOfReactants>

<speciesReference species="c_gmp" stoichiometry="1" constant="false"/>

<speciesReference species="c_h2o" stoichiometry="1" constant="false"/>

</listOfReactants>

<listOfProducts>

<speciesReference species="c_gsn" stoichiometry="1" constant="false"/>

<speciesReference species="c_pi" stoichiometry="1" constant="false"/>

</listOfProducts>

</reaction>

<reaction id="FRTT" name="FRTT" reversible="false" fast="false" compartment="cell">

<listOfReactants>

<speciesReference species="c_frdp" stoichiometry="1" constant="false"/>

<speciesReference species="c_ipdp" stoichiometry="1" constant="false"/>

</listOfReactants>

<listOfProducts>

<speciesReference species="c_ggdp" stoichiometry="1" constant="false"/>

<speciesReference species="c_ppi" stoichiometry="1" constant="false"/>

</listOfProducts>

</reaction>

<reaction id="DAGK" name="DAGK" reversible="false" fast="false" compartment="cell">

<listOfReactants>

<speciesReference species="c_12dgr" stoichiometry="1" constant="false"/>

<speciesReference species="c_atp" stoichiometry="1" constant="false"/>

</listOfReactants>

<listOfProducts>

<speciesReference species="c_12dag3p" stoichiometry="1" constant="false"/>

<speciesReference species="c_adp" stoichiometry="1" constant="false"/>

<speciesReference species="c_h" stoichiometry="1" constant="false"/>

</listOfProducts>

</reaction>

<reaction id="PPC" name="PPC" reversible="false" fast="false" compartment="cell">

<listOfReactants>

<speciesReference species="c_co2" stoichiometry="1" constant="false"/>

<speciesReference species="c_h2o" stoichiometry="1" constant="false"/>

<speciesReference species="c_pep" stoichiometry="1" constant="false"/>

</listOfReactants>

<listOfProducts>

<speciesReference species="c_h" stoichiometry="1" constant="false"/>

<speciesReference species="c_oaa" stoichiometry="1" constant="false"/>

<speciesReference species="c_pi" stoichiometry="1" constant="false"/>

</listOfProducts>

</reaction>

<reaction id="AACPS4" name="AACPS4" reversible="false" fast="false" compartment="cell">

<listOfReactants>

<speciesReference species="c_ACP" stoichiometry="1" constant="false"/>

<speciesReference species="c_atp" stoichiometry="1" constant="false"/>

<speciesReference species="c_hdcea" stoichiometry="1" constant="false"/>

</listOfReactants>

<listOfProducts>

<speciesReference species="c_amp" stoichiometry="1" constant="false"/>

<speciesReference species="c_hdeACP" stoichiometry="1" constant="false"/>

<speciesReference species="c_ppi" stoichiometry="1" constant="false"/>

</listOfProducts>

</reaction>

<reaction id="HBUTCT" name="HBUTCT" reversible="false" fast="false" compartment="cell">

<listOfReactants>

<speciesReference species="c_4hdxbutn" stoichiometry="1" constant="false"/>

<speciesReference species="c_btcoa" stoichiometry="1" constant="false"/>

</listOfReactants>

<listOfProducts>

<speciesReference species="c_4hbutcoa" stoichiometry="1" constant="false"/>

<speciesReference species="c_but" stoichiometry="1" constant="false"/>

</listOfProducts>

</reaction>

<reaction id="RNDR3" name="RNDR3" reversible="false" fast="false" compartment="cell">

<listOfReactants>

<speciesReference species="c_cdp" stoichiometry="1" constant="false"/>

<speciesReference species="c_trdrd" stoichiometry="1" constant="false"/>

</listOfReactants>

<listOfProducts>

<speciesReference species="c_dcdp" stoichiometry="1" constant="false"/>

<speciesReference species="c_h2o" stoichiometry="1" constant="false"/>

<speciesReference species="c_trdox" stoichiometry="1" constant="false"/>

</listOfProducts>

</reaction>

<reaction id="NTD4" name="NTD4" reversible="false" fast="false" compartment="cell">

<listOfReactants>

<speciesReference species="c_cmp" stoichiometry="1" constant="false"/>

<speciesReference species="c_h2o" stoichiometry="1" constant="false"/>

</listOfReactants>

<listOfProducts>

<speciesReference species="c_cytd" stoichiometry="1" constant="false"/>

<speciesReference species="c_pi" stoichiometry="1" constant="false"/>

</listOfProducts>

</reaction>

<reaction id="AP4AH" name="AP4AH" reversible="false" fast="false" compartment="cell">

<listOfReactants>

<speciesReference species="c_ap4a" stoichiometry="1" constant="false"/>

<speciesReference species="c_h2o" stoichiometry="1" constant="false"/>

</listOfReactants>

<listOfProducts>

<speciesReference species="c_adp" stoichiometry="2" constant="false"/>

<speciesReference species="c_h" stoichiometry="2" constant="false"/>

</listOfProducts>

</reaction>

<reaction id="GLU5K" name="GLU5K" reversible="false" fast="false" compartment="cell">

<listOfReactants>

<speciesReference species="c_atp" stoichiometry="1" constant="false"/>

<speciesReference species="c_glu_L" stoichiometry="1" constant="false"/>

</listOfReactants>

<listOfProducts>

<speciesReference species="c_adp" stoichiometry="1" constant="false"/>

<speciesReference species="c_glu5p" stoichiometry="1" constant="false"/>

</listOfProducts>

</reaction>

<reaction id="SHKK" name="SHKK" reversible="false" fast="false" compartment="cell">

<listOfReactants>

<speciesReference species="c_atp" stoichiometry="1" constant="false"/>

<speciesReference species="c_skm" stoichiometry="1" constant="false"/>

</listOfReactants>

<listOfProducts>

<speciesReference species="c_adp" stoichiometry="1" constant="false"/>

<speciesReference species="c_h" stoichiometry="1" constant="false"/>

<speciesReference species="c_skm5p" stoichiometry="1" constant="false"/>

</listOfProducts>

</reaction>

<reaction id="G6PDA" name="G6PDA" reversible="false" fast="false" compartment="cell">

<listOfReactants>

<speciesReference species="c_gam6p" stoichiometry="1" constant="false"/>

<speciesReference species="c_h2o" stoichiometry="1" constant="false"/>

</listOfReactants>

<listOfProducts>

<speciesReference species="c_f6p" stoichiometry="1" constant="false"/>

<speciesReference species="c_nh4" stoichiometry="1" constant="false"/>

</listOfProducts>

</reaction>

<reaction id="MDH" name="MDH" reversible="true" fast="false" compartment="cell">

<listOfReactants>

<speciesReference species="c_mal_L" stoichiometry="1" constant="false"/>

<speciesReference species="c_nad" stoichiometry="1" constant="false"/>

</listOfReactants>

<listOfProducts>

<speciesReference species="c_h" stoichiometry="1" constant="false"/>

<speciesReference species="c_nadh" stoichiometry="1" constant="false"/>

<speciesReference species="c_oaa" stoichiometry="1" constant="false"/>

</listOfProducts>

</reaction>

<reaction id="PPNCL" name="PPNCL" reversible="false" fast="false" compartment="cell">

<listOfReactants>

<speciesReference species="c_4ppan" stoichiometry="1" constant="false"/>

<speciesReference species="c_ctp" stoichiometry="1" constant="false"/>

<speciesReference species="c_cys_L" stoichiometry="1" constant="false"/>

</listOfReactants>

<listOfProducts>

<speciesReference species="c_4ppcys" stoichiometry="1" constant="false"/>

<speciesReference species="c_cdp" stoichiometry="1" constant="false"/>

<speciesReference species="c_h" stoichiometry="1" constant="false"/>

<speciesReference species="c_pi" stoichiometry="1" constant="false"/>

</listOfProducts>

</reaction>

<reaction id="HISD1" name="HISD1" reversible="false" fast="false" compartment="cell">

<listOfReactants>

<speciesReference species="c_his_L" stoichiometry="1" constant="false"/>

</listOfReactants>

<listOfProducts>

<speciesReference species="c_nh4" stoichiometry="1" constant="false"/>

<speciesReference species="c_urcan" stoichiometry="1" constant="false"/>

</listOfProducts>

</reaction>

<reaction id="ACACCT" name="ACACCT" reversible="false" fast="false" compartment="cell">

<listOfReactants>

<speciesReference species="c_acac" stoichiometry="1" constant="false"/>

<speciesReference species="c_accoa" stoichiometry="1" constant="false"/>

</listOfReactants>

<listOfProducts>

<speciesReference species="c_aacoa" stoichiometry="1" constant="false"/>

<speciesReference species="c_ac" stoichiometry="1" constant="false"/>

</listOfProducts>

</reaction>

<reaction id="U23GAAT" name="U23GAAT" reversible="false" fast="false" compartment="cell">

<listOfReactants>

<speciesReference species="c_3htdACP" stoichiometry="1" constant="false"/>

<speciesReference species="c_u3hga" stoichiometry="1" constant="false"/>

</listOfReactants>

<listOfProducts>

<speciesReference species="c_ACP" stoichiometry="1" constant="false"/>

<speciesReference species="c_h" stoichiometry="1" constant="false"/>

<speciesReference species="c_u23ga" stoichiometry="1" constant="false"/>

</listOfProducts>

</reaction>

<reaction id="NADH4" name="NADH4" reversible="false" fast="false" compartment="cell">

<listOfReactants>

<speciesReference species="c_h" stoichiometry="1" constant="false"/>

<speciesReference species="c_mqn7" stoichiometry="1" constant="false"/>

<speciesReference species="c_nadh" stoichiometry="1" constant="false"/>

</listOfReactants>

<listOfProducts>

<speciesReference species="c_mql7" stoichiometry="1" constant="false"/>

<speciesReference species="c_nad" stoichiometry="1" constant="false"/>

</listOfProducts>

</reaction>

<reaction id="NPHS" name="NPHS" reversible="false" fast="false" compartment="cell">

<listOfReactants>

<speciesReference species="c_sbzcoa" stoichiometry="1" constant="false"/>

</listOfReactants>

<listOfProducts>

<speciesReference species="c_coa" stoichiometry="1" constant="false"/>

<speciesReference species="c_dhna" stoichiometry="1" constant="false"/>

</listOfProducts>

</reaction>

<reaction id="PGSA" name="PGSA" reversible="true" fast="false" compartment="cell">

<listOfReactants>

<speciesReference species="c_cdpdag" stoichiometry="1" constant="false"/>

<speciesReference species="c_glyc3p" stoichiometry="1" constant="false"/>

</listOfReactants>

<listOfProducts>

<speciesReference species="c_cmp" stoichiometry="1" constant="false"/>

<speciesReference species="c_h" stoichiometry="1" constant="false"/>

<speciesReference species="c_pglyp" stoichiometry="1" constant="false"/>

</listOfProducts>

</reaction>

<reaction id="PAPPT3" name="PAPPT3" reversible="false" fast="false" compartment="cell">

<listOfReactants>

<speciesReference species="c_udcpp" stoichiometry="1" constant="false"/>

<speciesReference species="c_ugmda" stoichiometry="1" constant="false"/>

</listOfReactants>

<listOfProducts>

<speciesReference species="c_uagmda" stoichiometry="1" constant="false"/>

<speciesReference species="c_ump" stoichiometry="1" constant="false"/>

</listOfProducts>

</reaction>

<reaction id="CHORM" name="CHORM" reversible="false" fast="false" compartment="cell">

<listOfReactants>

<speciesReference species="c_chor" stoichiometry="1" constant="false"/>

</listOfReactants>

<listOfProducts>

<speciesReference species="c_pphn" stoichiometry="1" constant="false"/>

</listOfProducts>

</reaction>

<reaction id="RNDR4" name="RNDR4" reversible="false" fast="false" compartment="cell">

<listOfReactants>

<speciesReference species="c_trdrd" stoichiometry="1" constant="false"/>

<speciesReference species="c_udp" stoichiometry="1" constant="false"/>

</listOfReactants>

<listOfProducts>

<speciesReference species="c_dudp" stoichiometry="1" constant="false"/>

<speciesReference species="c_h2o" stoichiometry="1" constant="false"/>

<speciesReference species="c_trdox" stoichiometry="1" constant="false"/>

</listOfProducts>

</reaction>

<reaction id="CHORS" name="CHORS" reversible="false" fast="false" compartment="cell">

<listOfReactants>

<speciesReference species="c_3psme" stoichiometry="1" constant="false"/>

</listOfReactants>

<listOfProducts>

<speciesReference species="c_chor" stoichiometry="1" constant="false"/>

<speciesReference species="c_pi" stoichiometry="1" constant="false"/>

</listOfProducts>

</reaction>

<reaction id="RBK" name="RBK" reversible="false" fast="false" compartment="cell">

<listOfReactants>

<speciesReference species="c_atp" stoichiometry="1" constant="false"/>

<speciesReference species="c_rib_D" stoichiometry="1" constant="false"/>

</listOfReactants>

<listOfProducts>

<speciesReference species="c_adp" stoichiometry="1" constant="false"/>

<speciesReference species="c_h" stoichiometry="1" constant="false"/>

<speciesReference species="c_r5p" stoichiometry="1" constant="false"/>

</listOfProducts>

</reaction>

<reaction id="HCO3E" name="HCO3E" reversible="true" fast="false" compartment="cell">

<listOfReactants>

<speciesReference species="c_co2" stoichiometry="1" constant="false"/>

<speciesReference species="c_h2o" stoichiometry="1" constant="false"/>

</listOfReactants>

<listOfProducts>

<speciesReference species="c_h" stoichiometry="1" constant="false"/>

<speciesReference species="c_hco3" stoichiometry="1" constant="false"/>

</listOfProducts>

</reaction>

<reaction id="OMCDC" name="OMCDC" reversible="false" fast="false" compartment="cell">

<listOfReactants>

<speciesReference species="c_3c4mop" stoichiometry="1" constant="false"/>

<speciesReference species="c_h" stoichiometry="1" constant="false"/>

</listOfReactants>

<listOfProducts>

<speciesReference species="c_4mop" stoichiometry="1" constant="false"/>

<speciesReference species="c_co2" stoichiometry="1" constant="false"/>

</listOfProducts>

</reaction>

<reaction id="AGPR" name="AGPR" reversible="true" fast="false" compartment="cell">

<listOfReactants>

<speciesReference species="c_acg5sa" stoichiometry="1" constant="false"/>

<speciesReference species="c_nadp" stoichiometry="1" constant="false"/>

<speciesReference species="c_pi" stoichiometry="1" constant="false"/>

</listOfReactants>

<listOfProducts>

<speciesReference species="c_acg5p" stoichiometry="1" constant="false"/>

<speciesReference species="c_h" stoichiometry="1" constant="false"/>

<speciesReference species="c_nadph" stoichiometry="1" constant="false"/>

</listOfProducts>

</reaction>

<reaction id="PSSA" name="PSSA" reversible="true" fast="false" compartment="cell">

<listOfReactants>

<speciesReference species="c_cdpdag" stoichiometry="1" constant="false"/>

<speciesReference species="c_ser_L" stoichiometry="1" constant="false"/>

</listOfReactants>

<listOfProducts>

<speciesReference species="c_cmp" stoichiometry="1" constant="false"/>

<speciesReference species="c_h" stoichiometry="1" constant="false"/>

<speciesReference species="c_ps" stoichiometry="1" constant="false"/>

</listOfProducts>

</reaction>

<reaction id="ARSRD2" name="ARSRD2" reversible="false" fast="false" compartment="cell">

<listOfReactants>

<speciesReference species="c_arsna" stoichiometry="1" constant="false"/>

<speciesReference species="c_gthrd" stoichiometry="2" constant="false"/>

<speciesReference species="c_h" stoichiometry="2" constant="false"/>

</listOfReactants>

<listOfProducts>

<speciesReference species="c_arsni2" stoichiometry="1" constant="false"/>

<speciesReference species="c_gthox" stoichiometry="1" constant="false"/>

<speciesReference species="c_h2o" stoichiometry="1" constant="false"/>

</listOfProducts>

</reaction>

<reaction id="TYRTA" name="TYRTA" reversible="true" fast="false" compartment="cell">

<listOfReactants>

<speciesReference species="c_akg" stoichiometry="1" constant="false"/>

<speciesReference species="c_tyr_L" stoichiometry="1" constant="false"/>

</listOfReactants>

<listOfProducts>

<speciesReference species="c_34hpp" stoichiometry="1" constant="false"/>

<speciesReference species="c_glu_L" stoichiometry="1" constant="false"/>

</listOfProducts>

</reaction>

<reaction id="ACS" name="ACS" reversible="false" fast="false" compartment="cell">

<listOfReactants>

<speciesReference species="c_ac" stoichiometry="1" constant="false"/>

<speciesReference species="c_atp" stoichiometry="1" constant="false"/>

<speciesReference species="c_coa" stoichiometry="1" constant="false"/>

</listOfReactants>

<listOfProducts>

<speciesReference species="c_accoa" stoichiometry="1" constant="false"/>

<speciesReference species="c_amp" stoichiometry="1" constant="false"/>

<speciesReference species="c_ppi" stoichiometry="1" constant="false"/>

</listOfProducts>

</reaction>

<reaction id="DKMPPD2" name="DKMPPD2" reversible="false" fast="false" compartment="cell">

<listOfReactants>

<speciesReference species="c_dkmpp" stoichiometry="1" constant="false"/>

<speciesReference species="c_h2o" stoichiometry="3" constant="false"/>

</listOfReactants>

<listOfProducts>

<speciesReference species="c_2kmb" stoichiometry="1" constant="false"/>

<speciesReference species="c_for" stoichiometry="1" constant="false"/>

<speciesReference species="c_h" stoichiometry="6" constant="false"/>

<speciesReference species="c_pi" stoichiometry="1" constant="false"/>

</listOfProducts>

</reaction>

<reaction id="ALAR" name="ALAR" reversible="true" fast="false" compartment="cell">

<listOfReactants>

<speciesReference species="c_ala_L" stoichiometry="1" constant="false"/>

</listOfReactants>

<listOfProducts>

<speciesReference species="c_ala_D" stoichiometry="1" constant="false"/>

</listOfProducts>

</reaction>

<reaction id="UHGADA" name="UHGADA" reversible="false" fast="false" compartment="cell">

<listOfReactants>

<speciesReference species="c_h2o" stoichiometry="1" constant="false"/>

<speciesReference species="c_u3aga" stoichiometry="1" constant="false"/>

</listOfReactants>

<listOfProducts>

<speciesReference species="c_ac" stoichiometry="1" constant="false"/>

<speciesReference species="c_u3hga" stoichiometry="1" constant="false"/>

</listOfProducts>

</reaction>

<reaction id="AACPS5" name="AACPS5" reversible="false" fast="false" compartment="cell">

<listOfReactants>

<speciesReference species="c_ACP" stoichiometry="1" constant="false"/>

<speciesReference species="c_atp" stoichiometry="1" constant="false"/>

<speciesReference species="c_ocdcea" stoichiometry="1" constant="false"/>

</listOfReactants>

<listOfProducts>

<speciesReference species="c_amp" stoichiometry="1" constant="false"/>

<speciesReference species="c_octeACP" stoichiometry="1" constant="false"/>

<speciesReference species="c_ppi" stoichiometry="1" constant="false"/>

</listOfProducts>

</reaction>

<reaction id="DCYTD" name="DCYTD" reversible="false" fast="false" compartment="cell">

<listOfReactants>

<speciesReference species="c_dcyt" stoichiometry="1" constant="false"/>

<speciesReference species="c_h" stoichiometry="1" constant="false"/>

<speciesReference species="c_h2o" stoichiometry="1" constant="false"/>

</listOfReactants>

<listOfProducts>

<speciesReference species="c_duri" stoichiometry="1" constant="false"/>

<speciesReference species="c_nh4" stoichiometry="1" constant="false"/>

</listOfProducts>

</reaction>

<reaction id="ASPK" name="ASPK" reversible="true" fast="false" compartment="cell">

<listOfReactants>

<speciesReference species="c_asp_L" stoichiometry="1" constant="false"/>

<speciesReference species="c_atp" stoichiometry="1" constant="false"/>

</listOfReactants>

<listOfProducts>

<speciesReference species="c_4pasp" stoichiometry="1" constant="false"/>

<speciesReference species="c_adp" stoichiometry="1" constant="false"/>

</listOfProducts>

</reaction>

<reaction id="UAMAGS" name="UAMAGS" reversible="false" fast="false" compartment="cell">

<listOfReactants>

<speciesReference species="c_atp" stoichiometry="1" constant="false"/>

<speciesReference species="c_glu_D" stoichiometry="1" constant="false"/>

<speciesReference species="c_uama" stoichiometry="1" constant="false"/>

</listOfReactants>

<listOfProducts>

<speciesReference species="c_adp" stoichiometry="1" constant="false"/>

<speciesReference species="c_h" stoichiometry="1" constant="false"/>

<speciesReference species="c_pi" stoichiometry="1" constant="false"/>

<speciesReference species="c_uamag" stoichiometry="1" constant="false"/>

</listOfProducts>

</reaction>

<reaction id="AP5AH" name="AP5AH" reversible="false" fast="false" compartment="cell">

<listOfReactants>

<speciesReference species="c_ap5a" stoichiometry="1" constant="false"/>

<speciesReference species="c_h2o" stoichiometry="1" constant="false"/>

</listOfReactants>

<listOfProducts>

<speciesReference species="c_adp" stoichiometry="1" constant="false"/>

<speciesReference species="c_atp" stoichiometry="1" constant="false"/>

<speciesReference species="c_h" stoichiometry="2" constant="false"/>

</listOfProducts>

</reaction>

<reaction id="AACPS7" name="AACPS7" reversible="false" fast="false" compartment="cell">

<listOfReactants>

<speciesReference species="c_ACP" stoichiometry="1" constant="false"/>

<speciesReference species="c_atp" stoichiometry="1" constant="false"/>

<speciesReference species="c_hpdca" stoichiometry="1" constant="false"/>

</listOfReactants>

<listOfProducts>

<speciesReference species="c_amp" stoichiometry="1" constant="false"/>

<speciesReference species="c_hpdACP" stoichiometry="1" constant="false"/>

<speciesReference species="c_ppi" stoichiometry="1" constant="false"/>

</listOfProducts>

</reaction>

<reaction id="PSUDS" name="PSUDS" reversible="false" fast="false" compartment="cell">

<listOfReactants>

<speciesReference species="c_r5p" stoichiometry="1" constant="false"/>

<speciesReference species="c_ura" stoichiometry="1" constant="false"/>

</listOfReactants>

<listOfProducts>

<speciesReference species="c_h2o" stoichiometry="1" constant="false"/>

<speciesReference species="c_psd5p" stoichiometry="1" constant="false"/>

</listOfProducts>

</reaction>

<reaction id="MGCH" name="MGCH" reversible="false" fast="false" compartment="cell">

<listOfReactants>

<speciesReference species="c_3mgcoa" stoichiometry="1" constant="false"/>

<speciesReference species="c_h2o" stoichiometry="1" constant="false"/>

</listOfReactants>

<listOfProducts>

<speciesReference species="c_hmgcoa" stoichiometry="1" constant="false"/>

</listOfProducts>

</reaction>

<reaction id="GLUDx" name="GLUDx" reversible="true" fast="false" compartment="cell">

<listOfReactants>

<speciesReference species="c_glu_L" stoichiometry="1" constant="false"/>

<speciesReference species="c_h2o" stoichiometry="1" constant="false"/>

<speciesReference species="c_nad" stoichiometry="1" constant="false"/>

</listOfReactants>

<listOfProducts>

<speciesReference species="c_akg" stoichiometry="1" constant="false"/>

<speciesReference species="c_h" stoichiometry="1" constant="false"/>

<speciesReference species="c_nadh" stoichiometry="1" constant="false"/>

<speciesReference species="c_nh4" stoichiometry="1" constant="false"/>

</listOfProducts>

</reaction>

<reaction id="MI1PP" name="MI1PP" reversible="false" fast="false" compartment="cell">

<listOfReactants>

<speciesReference species="c_h2o" stoichiometry="1" constant="false"/>

<speciesReference species="c_mi1p_D" stoichiometry="1" constant="false"/>

</listOfReactants>

<listOfProducts>

<speciesReference species="c_inost" stoichiometry="1" constant="false"/>

<speciesReference species="c_pi" stoichiometry="1" constant="false"/>

</listOfProducts>

</reaction>

<reaction id="C60ISN" name="C60ISN" reversible="false" fast="false" compartment="cell">

<listOfReactants>

<speciesReference species="c_h" stoichiometry="3" constant="false"/>

<speciesReference species="c_ibcoa" stoichiometry="1" constant="false"/>

<speciesReference species="c_malACP" stoichiometry="1" constant="false"/>

<speciesReference species="c_nadph" stoichiometry="2" constant="false"/>

</listOfReactants>

<listOfProducts>

<speciesReference species="c_co2" stoichiometry="1" constant="false"/>

<speciesReference species="c_coa" stoichiometry="1" constant="false"/>

<speciesReference species="c_fa8ACP" stoichiometry="1" constant="false"/>

<speciesReference species="c_h2o" stoichiometry="1" constant="false"/>

<speciesReference species="c_nadp" stoichiometry="2" constant="false"/>

</listOfProducts>

</reaction>

<reaction id="SULR" name="SULR" reversible="true" fast="false" compartment="cell">

<listOfReactants>

<speciesReference species="c_h2o" stoichiometry="3" constant="false"/>

<speciesReference species="c_h2s" stoichiometry="1" constant="false"/>

<speciesReference species="c_nadp" stoichiometry="3" constant="false"/>

</listOfReactants>

<listOfProducts>

<speciesReference species="c_h" stoichiometry="4" constant="false"/>

<speciesReference species="c_nadph" stoichiometry="3" constant="false"/>

<speciesReference species="c_so3" stoichiometry="1" constant="false"/>

</listOfProducts>

</reaction>

<reaction id="AHSERL2" name="AHSERL2" reversible="false" fast="false" compartment="cell">

<listOfReactants>

<speciesReference species="c_achms" stoichiometry="1" constant="false"/>

<speciesReference species="c_h2s" stoichiometry="1" constant="false"/>

</listOfReactants>

<listOfProducts>

<speciesReference species="c_ac" stoichiometry="1" constant="false"/>

<speciesReference species="c_hcys_L" stoichiometry="1" constant="false"/>

</listOfProducts>

</reaction>

<reaction id="TMPPP" name="TMPPP" reversible="false" fast="false" compartment="cell">

<listOfReactants>

<speciesReference species="c_2mahmp" stoichiometry="1" constant="false"/>

<speciesReference species="c_4mpetz" stoichiometry="1" constant="false"/>

<speciesReference species="c_h" stoichiometry="1" constant="false"/>

</listOfReactants>

<listOfProducts>

<speciesReference species="c_ppi" stoichiometry="1" constant="false"/>

<speciesReference species="c_thmmp" stoichiometry="1" constant="false"/>

</listOfProducts>

</reaction>

<reaction id="ADA" name="ADA" reversible="false" fast="false" compartment="cell">

<listOfReactants>

<speciesReference species="c_adn" stoichiometry="1" constant="false"/>

<speciesReference species="c_h" stoichiometry="1" constant="false"/>

<speciesReference species="c_h2o" stoichiometry="1" constant="false"/>

</listOfReactants>

<listOfProducts>

<speciesReference species="c_ins" stoichiometry="1" constant="false"/>

<speciesReference species="c_nh4" stoichiometry="1" constant="false"/>

</listOfProducts>

</reaction>

<reaction id="MTHFC" name="MTHFC" reversible="true" fast="false" compartment="cell">

<listOfReactants>

<speciesReference species="c_h2o" stoichiometry="1" constant="false"/>

<speciesReference species="c_methf" stoichiometry="1" constant="false"/>

</listOfReactants>

<listOfProducts>

<speciesReference species="c_10fthf" stoichiometry="1" constant="false"/>

<speciesReference species="c_h" stoichiometry="1" constant="false"/>

</listOfProducts>

</reaction>

<reaction id="CKDOAS" name="CKDOAS" reversible="false" fast="false" compartment="cell">

<listOfReactants>

<speciesReference species="c_ckdo" stoichiometry="1" constant="false"/>

<speciesReference species="c_glu_L" stoichiometry="1" constant="false"/>

<speciesReference species="c_nad" stoichiometry="1" constant="false"/>

</listOfReactants>

<listOfProducts>

<speciesReference species="c_akg" stoichiometry="1" constant="false"/>

<speciesReference species="c_ckdo8n" stoichiometry="1" constant="false"/>

<speciesReference species="c_h" stoichiometry="1" constant="false"/>

<speciesReference species="c_nadh" stoichiometry="1" constant="false"/>

</listOfProducts>

</reaction>

<reaction id="CYSTRS" name="CYSTRS" reversible="false" fast="false" compartment="cell">

<listOfReactants>

<speciesReference species="c_atp" stoichiometry="1" constant="false"/>

<speciesReference species="c_cys_L" stoichiometry="1" constant="false"/>

<speciesReference species="c_trnacys" stoichiometry="1" constant="false"/>

</listOfReactants>

<listOfProducts>

<speciesReference species="c_amp" stoichiometry="1" constant="false"/>

<speciesReference species="c_cystrna" stoichiometry="1" constant="false"/>

<speciesReference species="c_ppi" stoichiometry="1" constant="false"/>

</listOfProducts>

</reaction>

<reaction id="ACGAMK" name="ACGAMK" reversible="false" fast="false" compartment="cell">

<listOfReactants>

<speciesReference species="c_acgam" stoichiometry="1" constant="false"/>

<speciesReference species="c_atp" stoichiometry="1" constant="false"/>

</listOfReactants>

<listOfProducts>

<speciesReference species="c_acgam6p" stoichiometry="1" constant="false"/>

<speciesReference species="c_adp" stoichiometry="1" constant="false"/>

<speciesReference species="c_h" stoichiometry="1" constant="false"/>

</listOfProducts>

</reaction>

<reaction id="SUCBZL" name="SUCBZL" reversible="false" fast="false" compartment="cell">

<listOfReactants>

<speciesReference species="c_atp" stoichiometry="1" constant="false"/>

<speciesReference species="c_coa" stoichiometry="1" constant="false"/>

<speciesReference species="c_sucbz" stoichiometry="1" constant="false"/>

</listOfReactants>

<listOfProducts>

<speciesReference species="c_amp" stoichiometry="1" constant="false"/>

<speciesReference species="c_ppi" stoichiometry="1" constant="false"/>

<speciesReference species="c_sbzcoa" stoichiometry="1" constant="false"/>

</listOfProducts>

</reaction>

<reaction id="TRPS2" name="TRPS2" reversible="false" fast="false" compartment="cell">

<listOfReactants>

<speciesReference species="c_indole" stoichiometry="1" constant="false"/>

<speciesReference species="c_ser_L" stoichiometry="1" constant="false"/>

</listOfReactants>

<listOfProducts>

<speciesReference species="c_h2o" stoichiometry="1" constant="false"/>

<speciesReference species="c_trp_L" stoichiometry="1" constant="false"/>

</listOfProducts>

</reaction>

<reaction id="EDTXS5" name="EDTXS5" reversible="false" fast="false" compartment="cell">

<listOfReactants>

<speciesReference species="c_ddcaACP" stoichiometry="1" constant="false"/>

<speciesReference species="c_kdo8nlipid4" stoichiometry="1" constant="false"/>

</listOfReactants>

<listOfProducts>

<speciesReference species="c_ACP" stoichiometry="1" constant="false"/>

<speciesReference species="c_kdo8nlipid4L" stoichiometry="1" constant="false"/>

</listOfProducts>

</reaction>

<reaction id="EDTXS6" name="EDTXS6" reversible="false" fast="false" compartment="cell">

<listOfReactants>

<speciesReference species="c_kdo8nlipid4L" stoichiometry="1" constant="false"/>

<speciesReference species="c_myrsACP" stoichiometry="1" constant="false"/>

</listOfReactants>

<listOfProducts>

<speciesReference species="c_ACP" stoichiometry="1" constant="false"/>

<speciesReference species="c_kdo8nlipa" stoichiometry="1" constant="false"/>

</listOfProducts>

</reaction>

<reaction id="FBP" name="FBP" reversible="false" fast="false" compartment="cell">

<listOfReactants>

<speciesReference species="c_fdp" stoichiometry="1" constant="false"/>

<speciesReference species="c_h2o" stoichiometry="1" constant="false"/>

</listOfReactants>

<listOfProducts>

<speciesReference species="c_f6p" stoichiometry="1" constant="false"/>

<speciesReference species="c_pi" stoichiometry="1" constant="false"/>

</listOfProducts>

</reaction>

<reaction id="OPHBDC" name="OPHBDC" reversible="false" fast="false" compartment="cell">

<listOfReactants>

<speciesReference species="c_3ophb" stoichiometry="1" constant="false"/>

<speciesReference species="c_h" stoichiometry="1" constant="false"/>

</listOfReactants>

<listOfProducts>

<speciesReference species="c_2oph" stoichiometry="1" constant="false"/>

<speciesReference species="c_co2" stoichiometry="1" constant="false"/>

</listOfProducts>

</reaction>

<reaction id="PGDH" name="PGDH" reversible="false" fast="false" compartment="cell">

<listOfReactants>

<speciesReference species="c_6pgc" stoichiometry="1" constant="false"/>

<speciesReference species="c_nadp" stoichiometry="1" constant="false"/>

</listOfReactants>

<listOfProducts>

<speciesReference species="c_co2" stoichiometry="1" constant="false"/>

<speciesReference species="c_nadph" stoichiometry="1" constant="false"/>

<speciesReference species="c_ru5p_D" stoichiometry="1" constant="false"/>

</listOfProducts>

</reaction>

<reaction id="FBA" name="FBA" reversible="true" fast="false" compartment="cell">

<listOfReactants>

<speciesReference species="c_fdp" stoichiometry="1" constant="false"/>

</listOfReactants>

<listOfProducts>

<speciesReference species="c_dhap" stoichiometry="1" constant="false"/>

<speciesReference species="c_g3p" stoichiometry="1" constant="false"/>
[truncated: 24,705 more chars]
